# Integrated Analysis of Gene Expression and Methylation Data to Identify Potential Biomarkers Related to Atherosclerosis Onset

**DOI:** 10.1155/2022/5493051

**Published:** 2022-07-22

**Authors:** Xiaoming Li, Xiaoqian Dong, Weidong Lu, Ke Yang, Xiao Li

**Affiliations:** ^1^Health Management Center, Shandong Provincial Hospital Affiliated to Shandong First Medical University, Jinan, 250021 Shandong, China; ^2^Department of Geriatric Medicine, Qilu Hospital of Shandong University, Jinan, 250012 Shandong, China; ^3^Key Laboratory of Cardiovascular Proteomics of Shandong Province, Qilu Hospital of Shandong University, Jinan, 250012 Shandong, China

## Abstract

Atherosclerosis is a kind of chronic inflammatory cardiovascular disease. Epigenetic regulation plays a crucial role in atherosclerosis. Our study was aimed at finding potential biomarkers associated with the occurrence of atherosclerosis. Two datasets were downloaded from the Gene Expression Omnibus (GEO) database. The epigenome-wide association study (EWAS) analysis was performed on methylation data using CpGassoc package. The differential expression analysis was conducted on mRNA data using limma package. The GO (Gene Ontology) and KEGG (Kyoto Encyclopedia of Genes and Genomes) functional enrichment was done in clusterProfiler package. Finally, the logistic regression model was constructed using generalized linear model (glm) function. Between atherosclerotic vs. nonatherosclerotic samples, totally 4980 cytosine-phosphate-guanine (CpG) sites (annotated to 2860 genes) and 132 differentially expressed genes (DEGs) related to atherosclerosis were identified. The annotated 2860 genes and 132 DEGs were significantly enriched in 9 and 4 KEGG pathways and 289 and 132 GO terms, respectively. After cross-analysis, 6 crucial CpG sites were screened to build the model, including cg01187920, cg03422911, cg08018825, cg10967350, cg14473924, and cg25313204. The diagnostic model could reliably separate the atherosclerosis samples from nonatherosclerotic samples. In conclusion, the 6 CpG sites are probably potential diagnostic biomarkers for atherosclerosis, including cg01187920, cg03422911, cg08018825, cg10967350, cg14473924, and cg25313204.

## 1. Introduction

Atherosclerosis is a common chronic inflammatory cardiovascular disease [1], and its consequent clinical manifestations are still the leading causes of death worldwide, especially in the elderly [2–4]. It usually originates from foam cells and fatty streaks in arterial walls, eventually leading to vessel-occluding plaques after several complex stages [1]. Besides, many cardiovascular diseases (CVDs) resulted from atherosclerosis are also important risk factors for patients, such as coronary heart disease and stroke [5]. Atherosclerosis cannot be diagnosed until a clinical feature emerges in many cases, which would increase the morbidity and mortality of the complications indirectly [6]. Consequently, despite new atheroprotective drugs and therapies have been applied, further exploration of early detection method of atherosclerotic lesion would be conducive to prompt intervention for patients. Currently, the crucial roles of genes and methylation variations in atherosclerosis have been demonstrated [7, 8], which indicates the atherosclerotic molecular heterogeneity and pathogenic complexity. Nevertheless, it is still necessary to identify novel potential biomarkers associated with the occurrence of atherosclerosis to provide more possible alternatives for atherosclerosis-related clinical decision-making.

DNA methylation is a common and important epigenetic mechanism in multiple biological processes, involving many cellular phenomena [9]. It is also a relatively stable marker during gene transcription [10]. Recently, aberrant DNA methylation has been increasingly reported to be related to various diseases, including atherosclerosis [11]. For instance, the ABCG1 methylation status has been evidenced to be negatively correlated with high-density lipoprotein cholesterol level, which might then contribute to the progression of atherosclerosis [12]. Moreover, multiple crucial factors involved in atherosclerosis, such as inflammatory response [13] and oxidative stress [14], have been influenced by the methylation status of the relative genes directly or indirectly, indicating that DNA methylation indeed involves in atherosclerosis. However, the role of DNA methylation in atherosclerosis is rarely explored in most studies. To find more novel possible biomarkers for atherosclerosis, we have integrated the gene expression and methylation data in the present study.

Gene Expression Omnibus (GEO) database (https://www.ncbi.nlm.nih.gov/geo/) is a public genomics data repository, involving gene expression and methylation data. Moreover, EWAS (epigenome-wide association studies) is a useful tool to explore the methylation variations and related diseases. Herein, via an integrated analysis of the gene expression and methylation data obtained from the GEO database, we expected to find potential biomarkers associated with the occurrence of atherosclerosis and build a diagnostic model, in order to provide more reference information for clinical decision-making of atherosclerosis in the future.

## 2. Materials and Methods

### 2.1. Research Objects

In this study, three datasets were downloaded from the GEO database, which included methylation data GSE46394 and the mRNA expression profile GSE43292 and GSE20129. In GSE46394 dataset, there were totally 49 samples' methylation data, comprising 15 atherosclerotic lesion samples and 34 aortic tissue samples. All samples' detailed information is shown in Table S1. In another dataset GSE43292, totally 64 samples' mRNA expression data were collected, which included 32 atherosclerotic lesion samples and 32 nonatherosclerotic tissue samples. There were 135 samples in GSE20129 dataset, comprising 57 atherosclerotic samples and 78 nonatherosclerotic samples. Among them, 119 samples were processed on Illumina HumanRef-8 V2.0 expression beadchip platform, and the rest 16 samples were processed on Illumina HumanHT-12 V4.0 expression beadchip platform.

### 2.2. EWAS (Epigenome-Wide Association Study) Analysis

To find the atherosclerosis-related cytosine-phosphate-guanine (CpG) sites, we used the CpGassoc package (https://cran.r-project.org/web/packages/cpgassoc/index.html) of R language (version 3.5.2) to analyze the data in GSE46394 dataset. The FDR < 0.000001 was used to select CpG sites in the promoter region (TSS200 (transcription start sites (TSS)), TSS1500, and 1st Exon).

### 2.3. Differentially Expressed Gene Analysis

Regarding the mRNA data in GSE43292 dataset, the differentially expressed genes (DEGs) between atherosclerosis specimens and nonatherosclerotic specimens were identified using “limma” function package of R [15]. The DEGs with |log_2_FC| > 1 and *P*.adjust < 0.05 were considered significant.

### 2.4. Functional Enrichment Analyses

Subsequently, the GO (Gene Ontology) and KEGG (Kyoto Encyclopedia of Genes and Genomes) enrichment analyses were conducted on the DEGs as well as the corresponding genes of differential CpG sites, using the clusterProfiler package [16] of R. The *P*.adjust < 0.05 was applied to screen the significantly enriched functional terms.

### 2.5. Protein-Protein Interaction (PPI) Network Analysis

STRING database is a useful tool for analyzing and predicting the functional interactions of proteins. We used STRING (version 11.0) [17] (https://string-db.org/) to analyze the functional interactions of proteins, and the PPI network was visualized using Cytoscape (version 3.7.2) [18].

### 2.6. Logistic Regression Prediction Model Construction

Logistic regression is a common method in classification, referring to predicting the classifications basing on a group of variables [19]. In our present work, the *β* value of various CpG sites were used for the prediction of sample type (atherosclerosis or nonatherosclerotic). Firstly, we selected the overlapped genes between the DEGs (based on mRNA profile) and the identified CpG sites' corresponding genes, and then, the CpG sites of the corresponding overlapped genes were obtained. Among which, the CpG sites located on CpG island would be used for logistic regression model construction. Based on two types of samples (atherosclerosis or nonatherosclerotic samples), the *β* value of various CpG sites were included as a continuous independent variable, and the sample type was included as a dichotomous response value. The logistic regression model was constructed using generalized linear model (glm) function of R.

## 3. Results

### 3.1. Results of EWAS Analysis in Atherosclerotic Samples

**Figure 1:**
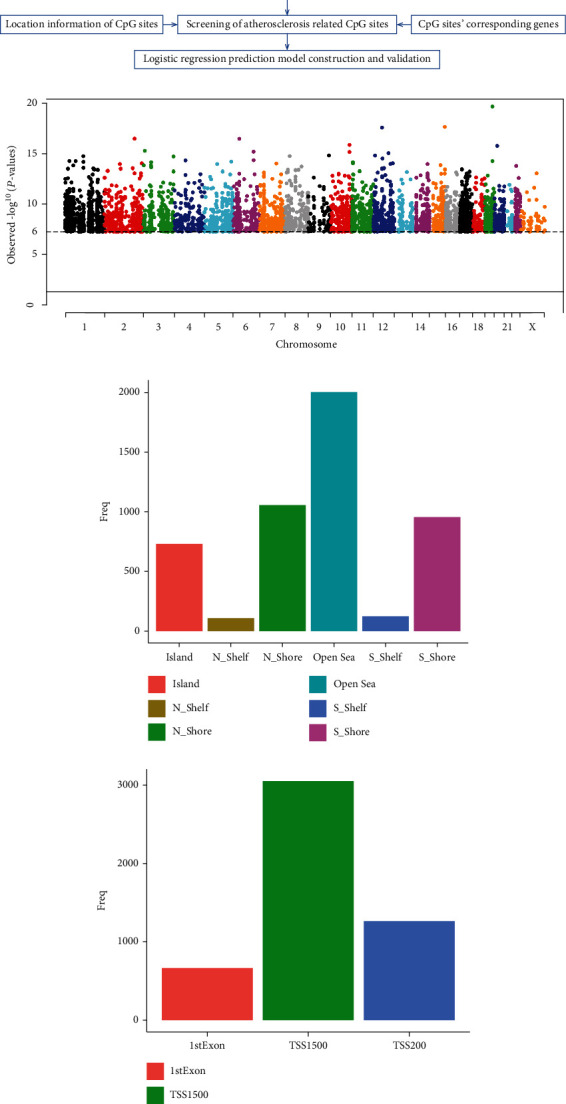
The atherosclerosis-related CpG sites and their distribution. (a) The flowchart of atherosclerosis-related CpG sites screening. (b) Manhattan map of CpG sites on all chromosomes. Horizontal axis: chromosome; vertical axis: -log10 (*P* value). (c) The distribution of the CpG sites on CpG island. (d) The distribution of the CpG sites on exact promoter region.

The screening process of atherosclerosis-related CpG sites has been displayed in Figure 1(a). Firstly, to obtain atherosclerosis-related CpG sites, we have analyzed the methylation data in GSE46394 dataset. After conducting EWAS analysis, we got the Manhattan map of CpG sites on various chromosomes, and each point represents a CpG site in Figure 1(b). The horizontal dashed line refers to log10(FDR), and the CpG sites above the dashed line met FDR < 0.000001. Then, a total of 4980 CpG sites located on promoter region were successfully identified to associate with the occurrence of atherosclerosis, which were annotated to 2860 mRNAs (Figure 1(b)). The genomic distribution of the CpG sites is shown in Figure 1(c), among which most CpG sites located on the open sea. Moreover, we also analyzed the exact promoter region distribution of the CpG sites, and most CpG sites located on TSS1500 region (Figure 1(d)). The detailed CpG sites are summarized in Table S2.

### 3.2. The Atherosclerosis-Related Differentially Expressed Genes

**Figure 2:**
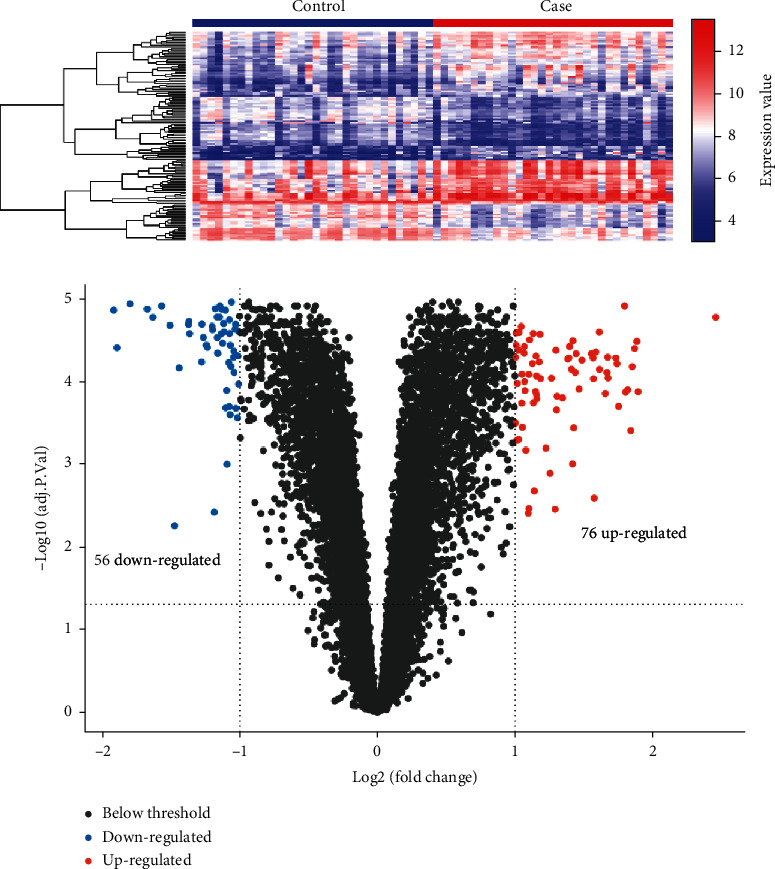
The atherosclerosis-related DEGs in GSE43292 dataset. (a) The expression level heat map of the DEGs. (b) The significant DEGs between atherosclerotic samples and nonatherosclerotic samples. Blue: downregulated genes; red: upregulated genes.

Subsequently, based on the mRNA data in GSE43292 dataset, significant atherosclerosis-related DEGs were screened. Compared with the nonatherosclerotic samples, a total of 132 DEGs were identified in atherosclerotic samples, among which 56 DEGs were downregulated and 76 DEGs were upregulated (Figure 2(b)). The expression levels of the DEGs were significantly differential between atherosclerotic samples and nonatherosclerotic samples (Figure 2(a)). All 132 DEGs are summarized in Table S3.

### 3.3. Construction of PPI Network

The above 132 DEGs were then subjected to a PPI analysis using STRING database. Those interaction pairs with minimum required interaction score > 0.4 were screened, and PPI network was visualized in Cytoscape software. The node and edge represented gene and interaction, respectively. There were a total of 100 nodes and 102 edges in the PPI network (Fig S1).

### 3.4. Functional Enrichment Results

To preliminarily obtain the functional information of the atherosclerosis-related genes, we have performed enrichment analyses on the CpG annotated genes and DEGs, respectively.

**Figure 3:**
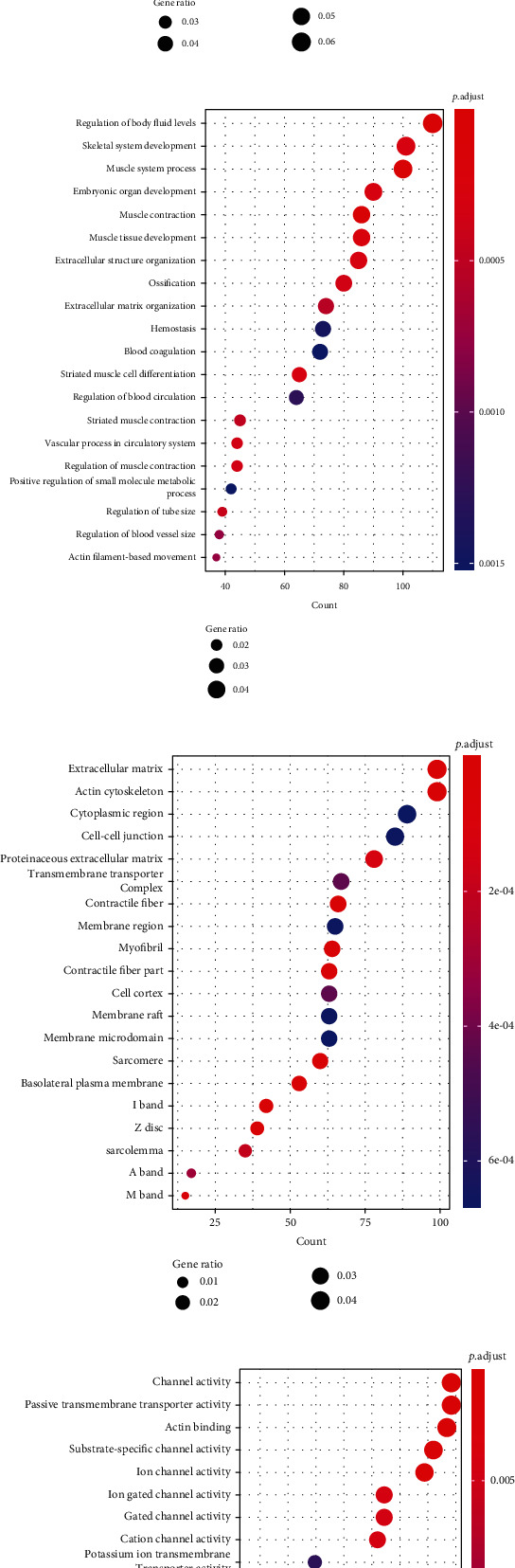
The results of enrichment analyses of the 2860 genes annotated by atherosclerosis-related CpG sites. (a) The significantly enriched 9 KEGG pathways. (b) The top 20 significantly enriched BP terms. (c) The top 20 significantly enriched CC terms. (d) The top 20 significantly enriched MF terms.

For the 2860 genes annotated by atherosclerosis-related CpG sites, they were significantly enriched in 9 KEGG pathways (Figure 3(a)), 197 Biological Process (BP) terms, 60 Cellular Component (CC) terms, and 32 Molecular Function (MF) terms (*P*.adjust < 0.05). Among which, the top 20 BP, CC, and MF terms are displayed in Figures 3(b)–3(d), separately. The detailed results are shown in Table S4.

**Figure 4:**
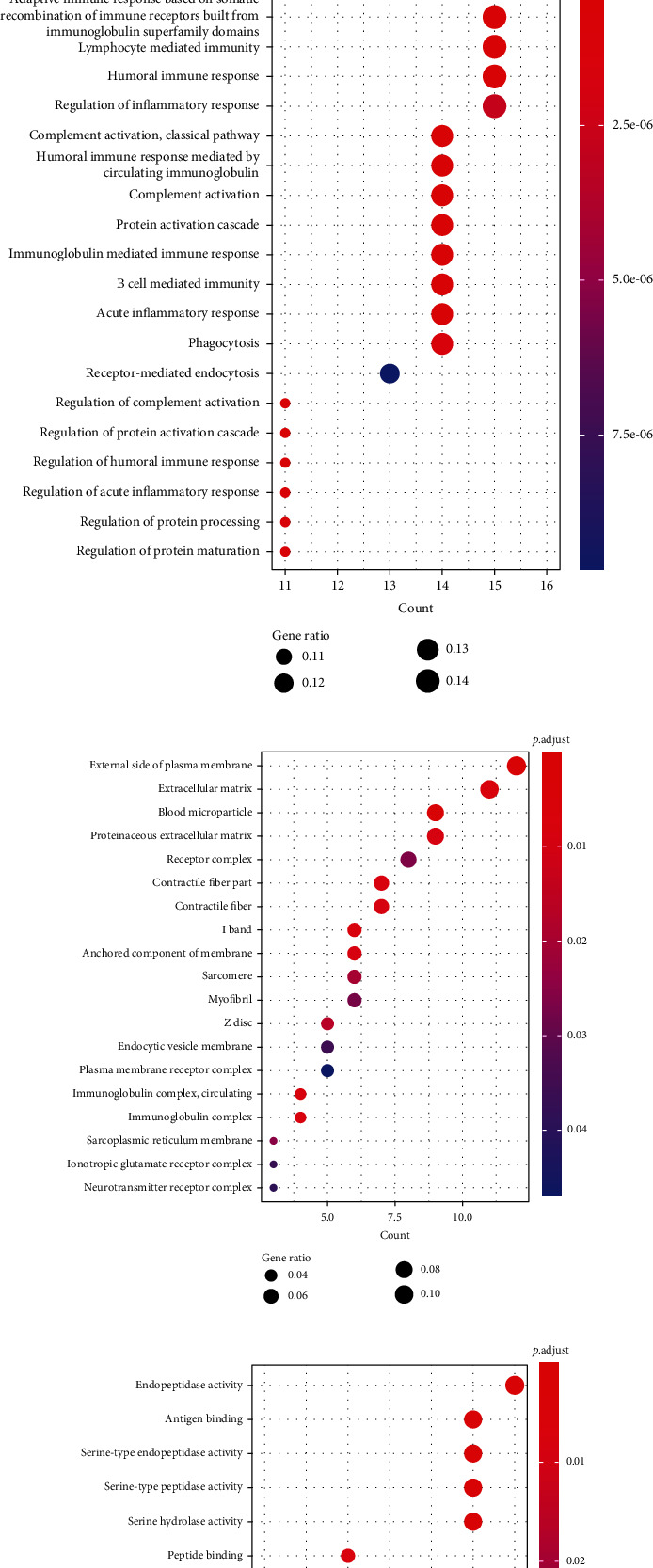
The results of enrichment analyses of the 131 DEGs. (a) The significantly enriched 4 KEGG pathways. (b) The top 20 significantly enriched BP terms. (c) The 19 significantly enriched CC terms. (d) The 13 significantly enriched MF terms.

Additionally, functional enrichment analyses were also conducted on the 131 DEGs associated with atherosclerosis. Our results showed that 131 DEGs were significantly enriched in 4 KEGG pathways (Figure 4(a)), 100 BP terms (the top 20 terms are displayed in Figure 4(b)), 19 CC terms (Figure 4(c)), and 13 MF terms (Figure 4(d)) (*P*.adjust < 0.05). Detailed information of the terms is listed in Table S5.

### 3.5. Logistic Regression Prediction Model Construction

Furthermore, to find more important CpG sites related to atherosclerosis, the cross-analysis was conducted on 2860 genes (annotated by 4980 CpG sites) and 131 DEGs; finally, we identified 32 overlapped genes. Then, the original corresponding CpG sites of these 32 overlapped genes were found, a total of 56 CpG sites. Only the CpG sites located on CpG island were selected to construct the logistic regression model. Finally, 6 CpG sites were screened to build the model, including cg01187920, cg03422911, cg08018825, cg10967350, cg14473924, and cg25313204, and the corresponding annotated genes are listed in Table 1. These 6 crucial CpG sites were all located on the TSS or 1st Exon regions, indicating their important epigenetic regulation on annotated genes. Accordingly, the expression levels of these 6 vital annotated genes in validation dataset were then evaluated. In GSE20129 dataset, CARTPT, RYR2, CNTN4, PDZRN3, and SLC22A3 all showed differential expression levels in atherosclerosis vs. nonatherosclerotic samples (Fig S2A–S2E). Among them, PDZRN3 was significantly highly expressed in atherosclerosis samples compared with nonatherosclerotic samples (Fig S2E).

**Figure 5:**
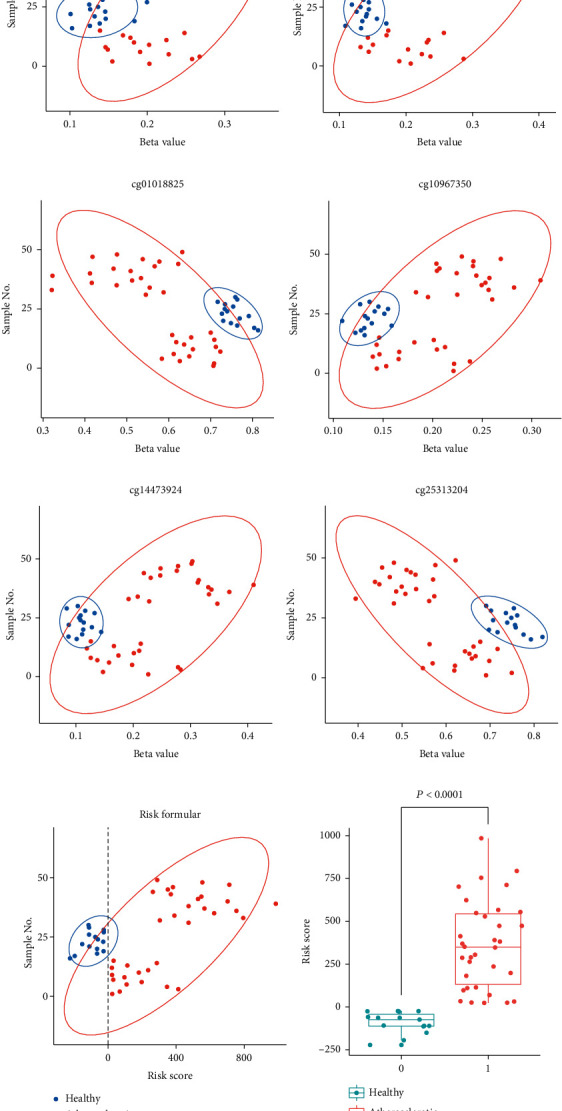
The results of logistic regression model construction. (a–f) The significantly differential *β* values of 6 CpG sites could be observed between atherosclerosis samples and nonatherosclerotic samples. (g and h) There was a significant difference of the risk score between atherosclerosis samples and nonatherosclerotic samples.

Subsequently, atherosclerosis or nonatherosclerotic sample type was taken as the dependent variable, a regression model based on the 6 CpG sites was established using glm function, and the atherosclerosis risk formula was displayed below. Risk = 518.9722∗cg01187920 + 634.4440∗cg03422911 + (−2139.4961)∗cg08018825 + (−1645.4335)∗cg10967350 + 715.9397∗cg14473924 + 213.9591∗cg25313204. The risk score > 0 represented that atherosclerosis will happen, while the risk score < 0 represented that atherosclerosis will not occur. There was a significant risk score difference between atherosclerosis samples and nonatherosclerotic samples (*P* < 0.0001) (Figure 5(h)). Our results suggested that the logistic regression model we built could reliably separate the atherosclerosis specimens from the nonatherosclerotic specimens (Figure 5(g)). Besides, the significantly differential *β* value of each CpG site could be observed between atherosclerosis samples and nonatherosclerotic samples (Figures 5(a)–5(f)), which further evidenced the crucial role of the 6 CpG sites in the occurrence of atherosclerosis. Additionally, the area under curve (AUC) value of our logistic regression model was 1 (Fig S3A), indicating the accuracy of our model. There was no significant outlier in the model (Fig S3B), and independent variables and dependent variables showed a great linear correlation (Fig S3C). Collectively, our findings indicated that our logistic regression model was able to distinguish the atherosclerosis samples from nonatherosclerotic samples accurately.

## 4. Discussion

Emerging studies indicate the important role of methylation in atherosclerosis [20]; thus, we herein explored potential CpG sites associated with the onset of atherosclerosis in this study. Based on the integrated analysis of atherosclerotic gene expression and methylation data from three GEO datasets, 6 CpG sites were found to be closely related to the occurrence of atherosclerosis. The diagnostic model constructed based on them further validated their crucial roles in the onset of atherosclerosis.

As a pivotal epigenetic modification, DNA methylation often regulates cell function via gene silence [11]. Given the high morbidity and mortality of atherosclerosis and its complications, many studies have revealed possible associations between methylation and atherosclerosis pathology [21, 22]. Moreover, atherosclerosis has even been considered as a kind of epigenetic disorder [23]. Accumulating evidence indicates the influence of aberrant DNA methylation on inflammatory response and endothelial injury [9]. Thus, we aimed to further explore the potential CpG sites as diagnostic biomarkers for atherosclerosis. After conducting EWAS analysis, 4980 CpG sites were associated with atherosclerosis, corresponding to 2860 genes. Besides, we have also identified 132 DEGs between atherosclerotic and nonatherosclerotic samples in another dataset GSE43292, which were probably associated with the onset of atherosclerosis. Then, there were totally 32 overlapped genes between the two datasets, corresponding to 56 CpG sites. As the importance of methylation of CpG islands is widely known [24], 6 CpG sites on CpG islands were subsequently selected to build the logistic regression model. The model could reliably separate atherosclerosis samples from the nonatherosclerotic ones.

These 6 CpG sites were annotated to 6 genes, CARTPT (CART Prepropeptide), RYR2 (Ryanodine receptor 2), SEL1L3 (SEL1L family member 3), CNTN4 (Contactin 4), PDZRN3 (PDZ domain containing ring finger 3), and SLC22A3 (Solute carrier family 22 member 3). Some evidence supporting our findings can be found from several previous studies. For example, SEL1L3 has been suggested to probably involve in cardiovascular mechanisms and blood pressure regulation [25], which reminded us that cg08018825 methylation might influence the SEL1L3 expression and affect the atherosclerosis status indirectly. The CNTN4 genotypes were indicated to be correlated with an excess of cardiovascular events [26], but how CNTN4 influenced atherosclerosis was not clarified in our study. Additionally, a recent study reported that SLC22A3 polymorphisms might decrease the risk of coronary heart disease by against inflammatory response [27], which implied that the inflammation response may be affected by cg25313204 methylation on SLC22A3 in atherosclerosis. Although PDZRN3 has been rarely reported in atherosclerosis as far as we know, PDZRN3 has been evidenced to involve in multiple developmental processes, such as vascular morphogenesis [28], differentiation of myoblasts [28], and endothelial intercellular junctions [28]. Nevertheless, the role of CARTPT and PDZRN3 methylation in atherosclerosis has been firstly revealed in our study. Collectively, the epigenetic regulation of these 6 genes potentially exerted crucial roles in the onset of atherosclerosis, and further exploration of them will be probably helpful to better understand atherosclerosis.

Furthermore, we have also preliminarily studied the functional information of atherosclerosis-related genes based on the GO and KEGG enrichment results. The 2860 genes annotated by atherosclerosis-related CpG sites in GSE46394 were significantly enriched in 9 KEGG pathways and 289 GO terms. The 132 DEGs in GSE43292 were significantly enriched in 4 KEGG pathways and 132 GO terms. Among which, there was one overlap pathway between the two datasets, cAMP signaling pathway. It has been recently evidenced that cAMP signaling pathway was activated in atherosclerosis; besides, aspirin could inactivate cAMP pathway to suppress atherosclerosis progression via downregulating the vascular smooth muscle cells (VSMCs) proliferation rate [29]. Notably, the vascular smooth muscle contraction pathway was also significantly enriched in our study, whose related endothelial dysfunction has been considered as one of the important atherosclerotic initiators [30, 31]. Moreover, another research revealed that the stimulation of cAMP pathway-induced autophagy was probably involved in antiatherosclerosis and anti-inflammation [32]. Endothelial inflammation was reported to be attenuated through cAMP pathway induced autophagy [33]. The vital role of inflammation in various stages of atherosclerosis has been widely studied [3]. Additionally, the platelet activation and inflammatory environment have been considered promising therapeutic targets preventing atherosclerosis [34]. For all above, our findings are consistent with the previous studies.

## 5. Conclusions

In summary, an integrative analysis has been performed on the atherosclerotic gene expression and methylation data, and a diagnostic signature based on 6 CpG sites has been revealed. After accuracy evaluation, our diagnostic model can reliably distinguish atherosclerotic samples from nonatherosclerotic ones. Among the 6 regulated genes by these crucial CpG sites, PDZRN3 exhibits significantly higher expression in atherosclerotic samples, deserving further investigation.

## Figures and Tables

**Table 1:** The corresponding annotated genes of 6 core CpG sites.

ID	FDR	UCSC_RefGene_Name	Location
cg01187920	5.79E-07	CARTPT	1st Exon
cg03422911	9.56E-07	RYR2	TSS1500
cg08018825	9.20E-07	SEL1L3	TSS1500
cg10967350	8.04E-07	CNTN4	TSS1500
cg14473924	6.03E-07	PDZRN3	TSS200
cg25313204	1.63E-07	SLC22A3	TSS1500

TSS: transcription start sites.

## Data Availability

The data used to support the findings of this study are included within the Supplementary Table 1.

## Supplementary Materials

Supplementary 1 Figure S1: construction of PPI network. The nodes and edges represented genes and interactions, respectively.

Supplementary 2 Figure S2: expression levels of 5 annotated genes in GSE20129 dataset. (A–E) The expressions of RYR2, SLC22A3, CNTN4, CARTPT, and PDZRN3, separately.

Supplementary 3 Figure S3: evaluation of logistic regression model. (A) The ROC curve. *X*-axis: false positive rate (FPR), *Y*-axis: true positive rate (TPR). (B) There was no significant outlier in the model (COOK distance > 0.5 refers to influential points). (C) The component plus residual plot of the 6 CpG sites included in the model.

Supplementary 4 Table S1: detailed characterization of all samples.

Supplementary 5 Table S2: the detailed CpG sites list.

Supplementary 6 Table S3: detailed 132 DEGs.

Supplementary 7 Table S4: the detailed functional enrichment results of the 2860 genes.

Supplementary 8 Table S5: the detailed functional enrichment results of the 132 DEGs.
