# Supplementary material for: Integrated Analysis of Gene Expression and Methylation Data to Identify Potential Biomarkers Related to Atherosclerosis Onset
Source: Oxid Med Cell Longev. 2022 Jul 22;2022:5493051. doi: 10.1155/2022/5493051 (PMC9338736; doi:10.1155/2022/5493051)
Supplement: Supplementary 1 — Figure S1: construction of PPI network. The nodes and edges represented genes and interactions, respectively. [file 5493051.f1.docx]

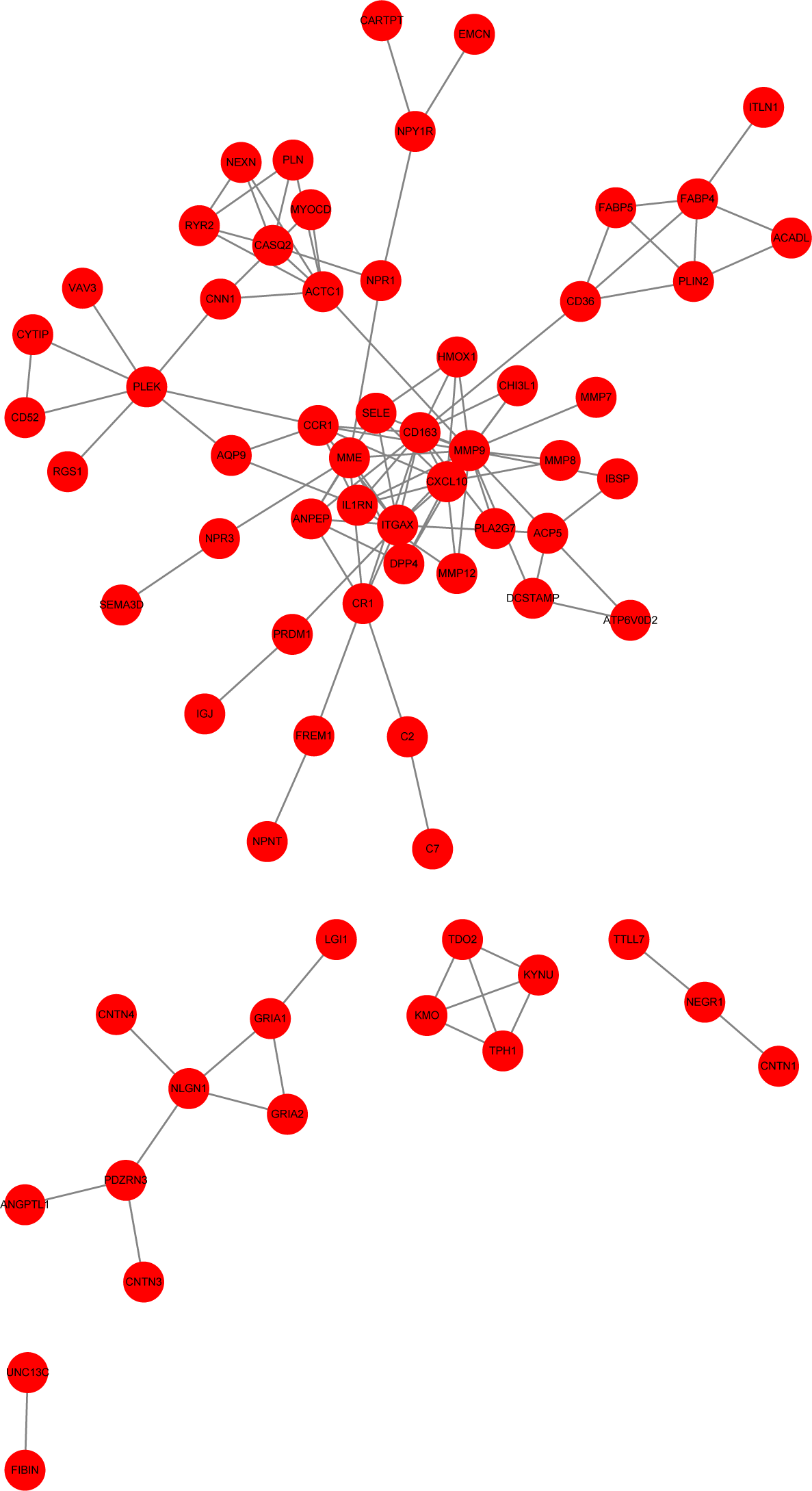


**Figure S1.** Construction of PPI network. The nodes and edges represented genes and interactions, respectively.
