# Supplementary material for: Integrated Analysis of Gene Expression and Methylation Data to Identify Potential Biomarkers Related to Atherosclerosis Onset
Source: Oxid Med Cell Longev. 2022 Jul 22;2022:5493051. doi: 10.1155/2022/5493051 (PMC9338736; doi:10.1155/2022/5493051)
Supplement: Supplementary 2 — Figure S2: expression levels of 5 annotated genes in GSE20129 dataset. (A–E) The expressions of RYR2, SLC22A3, CNTN4, CARTPT, and PDZRN3, separately. [file 5493051.f2.docx]

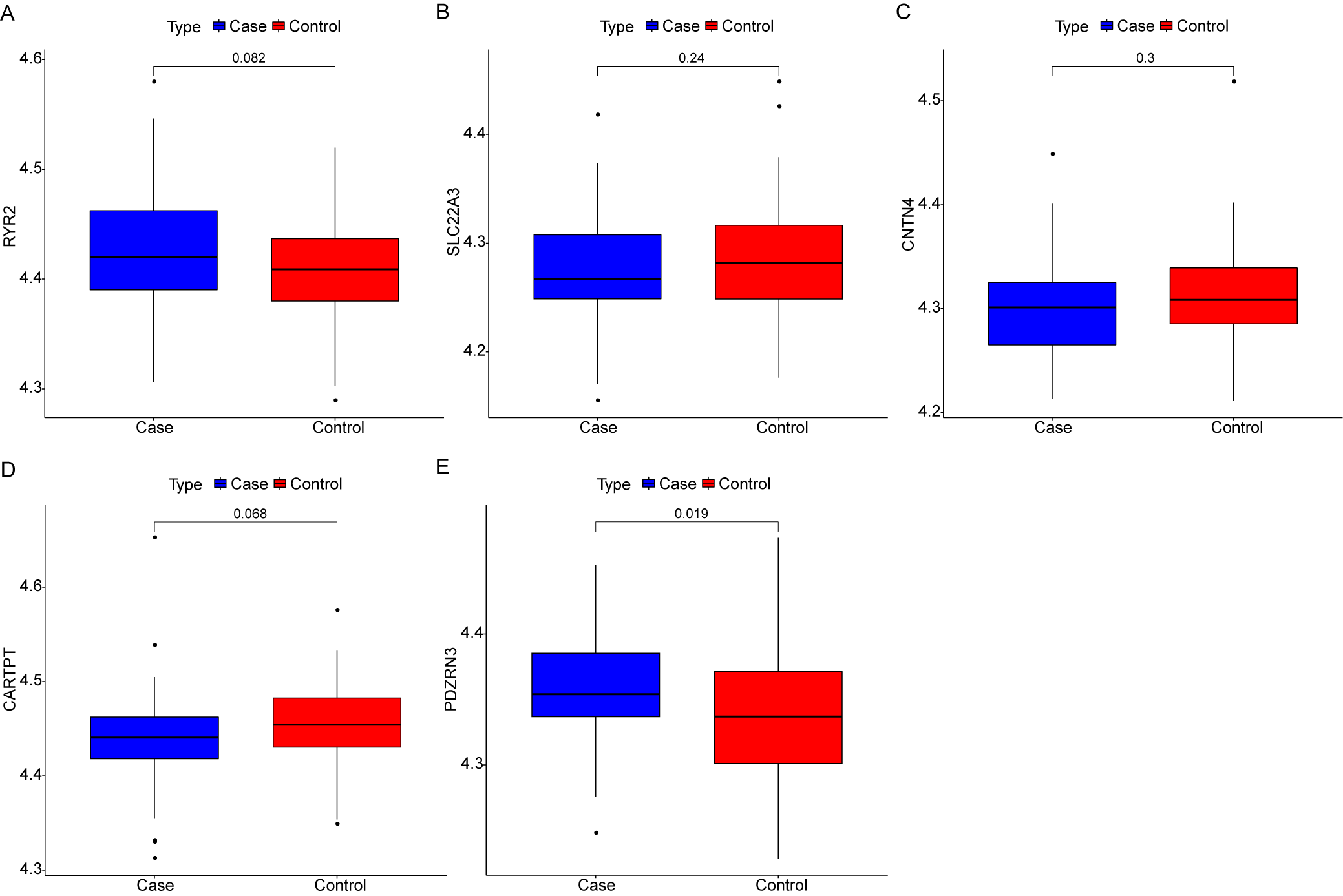


**Figure S2** Expression levels of 5 annotated genes in GSE20129 dataset. (A-E) The expressions of RYR2, SLC22A3, CNTN4, CARTPT, and PDZRN3, separately.
