# Supplementary material for: Integrated Analysis of Gene Expression and Methylation Data to Identify Potential Biomarkers Related to Atherosclerosis Onset
Source: Oxid Med Cell Longev. 2022 Jul 22;2022:5493051. doi: 10.1155/2022/5493051 (PMC9338736; doi:10.1155/2022/5493051)
Supplement: Supplementary 3 — Figure S3: evaluation of logistic regression model. (A) The ROC curve. X-axis: false positive rate (FPR), Y-axis: true positive rate (TPR). (B) There was no significant outlier in the model (COOK distance > 0.5 refers to influential points). (C) The component plus residual plot of the 6 CpG sites included in the model. [file 5493051.f3.docx]

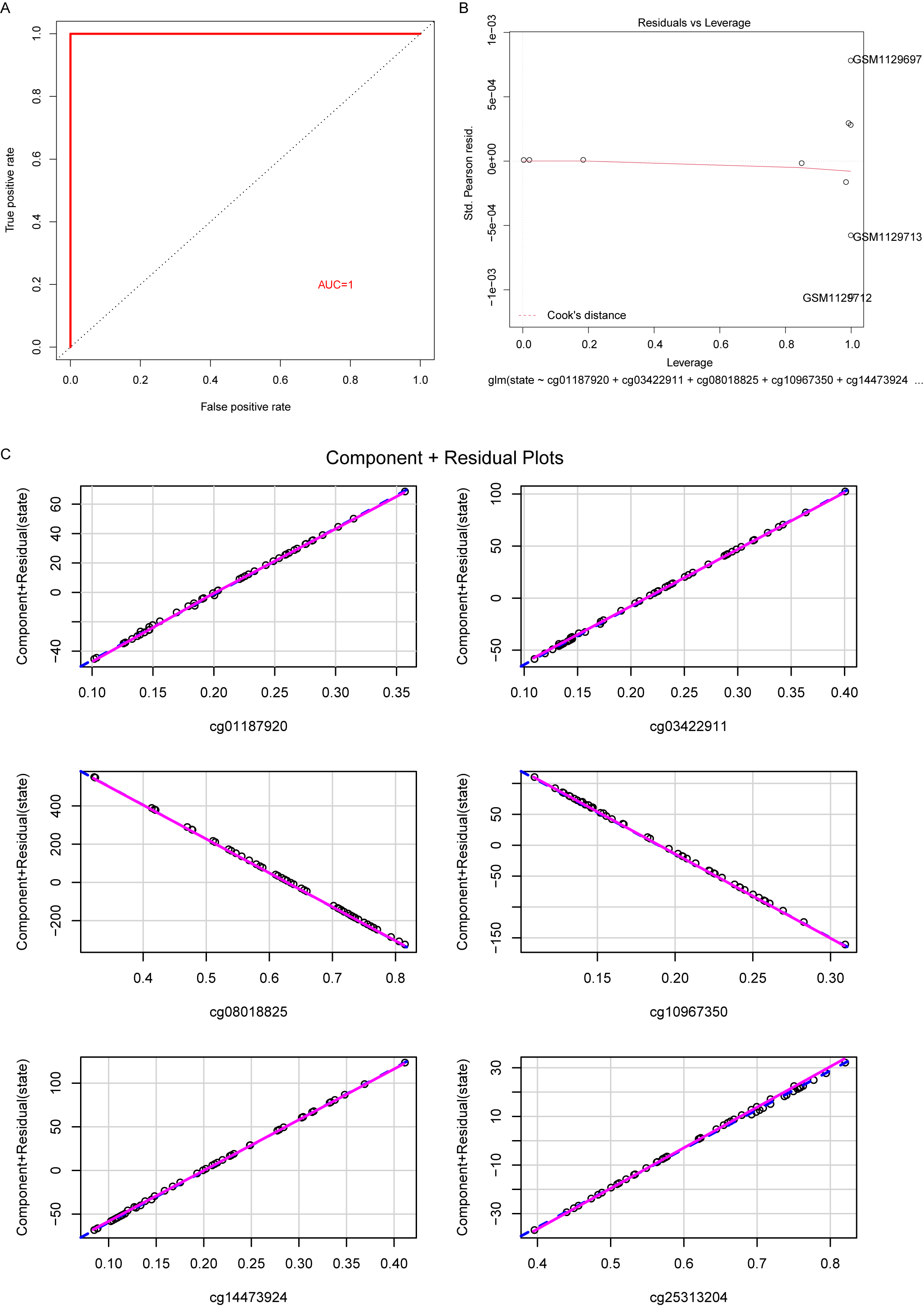


**Figure S3** Evaluation of logistic regression model. (A) The ROC curve. X-axis: false postive rate (FPR), Y-axis: true postive rate (TPR). (B) There was no significant outlier in the model (COOK distance >0.5 refers to influentia points). (C) The component plus residual plot of the 6 CpG sites included in the model.
