# Supplementary material for: Integrated Analysis of Gene Expression and Methylation Data to Identify Potential Biomarkers Related to Atherosclerosis Onset
Source: Oxid Med Cell Longev. 2022 Jul 22;2022:5493051. doi: 10.1155/2022/5493051 (PMC9338736; doi:10.1155/2022/5493051)
Supplement: Supplementary 4 — Table S1: detailed characterization of all samples. [file 5493051.f4.docx]

Table S1 Detailed characterization of all samples

| Sample | Type | Sex | Age(years) |
| --- | --- | --- | --- |
| [GSM1129686](https://www.ncbi.nlm.nih.gov/geo/query/acc.cgi?acc=GSM1129686) | Atherosclerotic lesion 93 | Female | 88 |
| 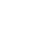[GSM1129687](https://www.ncbi.nlm.nih.gov/geo/query/acc.cgi?acc=GSM1129687) | Atherosclerotic lesion 95 | Female | 69 |
| [GSM1129688](https://www.ncbi.nlm.nih.gov/geo/query/acc.cgi?acc=GSM1129688) | Atherosclerotic lesion 96 | Male | 71 |
| [GSM1129689](https://www.ncbi.nlm.nih.gov/geo/query/acc.cgi?acc=GSM1129689) | Atherosclerotic lesion 97 | Male | 69 |
| [GSM1129690](https://www.ncbi.nlm.nih.gov/geo/query/acc.cgi?acc=GSM1129690) | Atherosclerotic lesion 98 | Male | 55 |
| [GSM1129691](https://www.ncbi.nlm.nih.gov/geo/query/acc.cgi?acc=GSM1129691) | Atherosclerotic lesion 99 | Female | 65 |
| [GSM1129692](https://www.ncbi.nlm.nih.gov/geo/query/acc.cgi?acc=GSM1129692) | Atherosclerotic lesion 100 | Male | 73 |
| [GSM1129693](https://www.ncbi.nlm.nih.gov/geo/query/acc.cgi?acc=GSM1129693) | Atherosclerotic lesion 101 | Male | 45 |
| [GSM1129694](https://www.ncbi.nlm.nih.gov/geo/query/acc.cgi?acc=GSM1129694) | Atherosclerotic lesion 102 | Male | 65 |
| [GSM1129695](https://www.ncbi.nlm.nih.gov/geo/query/acc.cgi?acc=GSM1129695) | Atherosclerotic lesion 103 | Male | 61 |
| [GSM1129696](https://www.ncbi.nlm.nih.gov/geo/query/acc.cgi?acc=GSM1129696) | Atherosclerotic lesion 104 | Female | 71 |
| [GSM1129697](https://www.ncbi.nlm.nih.gov/geo/query/acc.cgi?acc=GSM1129697) | Atherosclerotic lesion 5 | Female | 54 |
| [GSM1129698](https://www.ncbi.nlm.nih.gov/geo/query/acc.cgi?acc=GSM1129698) | Atherosclerotic lesion 6 | Male | 46 |
| [GSM1129699](https://www.ncbi.nlm.nih.gov/geo/query/acc.cgi?acc=GSM1129699) | Atherosclerotic lesion 7 | Male | 72 |
| [GSM1129700](https://www.ncbi.nlm.nih.gov/geo/query/acc.cgi?acc=GSM1129700) | Atherosclerotic lesion 9 | Male | 78 |
| [GSM1129701](https://www.ncbi.nlm.nih.gov/geo/query/acc.cgi?acc=GSM1129701) | Aortic tissue 93 | Female | 88 |
| [GSM1129702](https://www.ncbi.nlm.nih.gov/geo/query/acc.cgi?acc=GSM1129702) | Aortic tissue 95 | Female | 69 |
| [GSM1129703](https://www.ncbi.nlm.nih.gov/geo/query/acc.cgi?acc=GSM1129703) | Aortic tissue 96 | Male | 71 |
| [GSM1129704](https://www.ncbi.nlm.nih.gov/geo/query/acc.cgi?acc=GSM1129704) | Aortic tissue 97 | Male | 69 |
| [GSM1129705](https://www.ncbi.nlm.nih.gov/geo/query/acc.cgi?acc=GSM1129705) | Aortic tissue 98 | Male | 55 |
| [GSM1129706](https://www.ncbi.nlm.nih.gov/geo/query/acc.cgi?acc=GSM1129706) | Aortic tissue 99 | Female | 65 |
| [GSM1129707](https://www.ncbi.nlm.nih.gov/geo/query/acc.cgi?acc=GSM1129707) | Aortic tissue 100 | Male | 73 |
| [GSM1129708](https://www.ncbi.nlm.nih.gov/geo/query/acc.cgi?acc=GSM1129708) | Aortic tissue 101 | Male | 45 |
| [GSM1129709](https://www.ncbi.nlm.nih.gov/geo/query/acc.cgi?acc=GSM1129709) | Aortic tissue 102 | Male | 65 |
| [GSM1129710](https://www.ncbi.nlm.nih.gov/geo/query/acc.cgi?acc=GSM1129710) | Aortic tissue 103 | Male | 61 |
| [GSM1129711](https://www.ncbi.nlm.nih.gov/geo/query/acc.cgi?acc=GSM1129711) | Aortic tissue 104 | Female | 71 |
| [GSM1129712](https://www.ncbi.nlm.nih.gov/geo/query/acc.cgi?acc=GSM1129712) | Aortic tissue 5 | Female | 54 |
| [GSM1129713](https://www.ncbi.nlm.nih.gov/geo/query/acc.cgi?acc=GSM1129713) | Aortic tissue 6 | Male | 46 |
| [GSM1129714](https://www.ncbi.nlm.nih.gov/geo/query/acc.cgi?acc=GSM1129714) | Aortic tissue 7 | Male | 72 |
| [GSM1129715](https://www.ncbi.nlm.nih.gov/geo/query/acc.cgi?acc=GSM1129715) | Aortic tissue 9 | Male | 78 |
| [GSM1129716](https://www.ncbi.nlm.nih.gov/geo/query/acc.cgi?acc=GSM1129716) | Carotid atherosclerotic samples 30 | Male | 67 |
| [GSM1129717](https://www.ncbi.nlm.nih.gov/geo/query/acc.cgi?acc=GSM1129717) | Carotid atherosclerotic samples 154 | Female | 65 |
| [GSM1129718](https://www.ncbi.nlm.nih.gov/geo/query/acc.cgi?acc=GSM1129718) | Carotid atherosclerotic samples 172 | Female | 74 |
| [GSM1129719](https://www.ncbi.nlm.nih.gov/geo/query/acc.cgi?acc=GSM1129719) | Carotid atherosclerotic samples 180 | Male | 76 |
| [GSM1129720](https://www.ncbi.nlm.nih.gov/geo/query/acc.cgi?acc=GSM1129720) | Carotid atherosclerotic samples 219 | Male | 67 |
| [GSM1129721](https://www.ncbi.nlm.nih.gov/geo/query/acc.cgi?acc=GSM1129721) | Carotid atherosclerotic samples 220 | Male | 66 |
| [GSM1129722](https://www.ncbi.nlm.nih.gov/geo/query/acc.cgi?acc=GSM1129722) | Carotid atherosclerotic samples 344 | Male | 74 |
| [GSM1129723](https://www.ncbi.nlm.nih.gov/geo/query/acc.cgi?acc=GSM1129723) | Carotid atherosclerotic samples 390 | Female | 72 |
| [GSM1129724](https://www.ncbi.nlm.nih.gov/geo/query/acc.cgi?acc=GSM1129724) | Carotid atherosclerotic samples 413 | Male | 73 |
| [GSM1129725](https://www.ncbi.nlm.nih.gov/geo/query/acc.cgi?acc=GSM1129725) | Carotid atherosclerotic samples 414 | Male | 62 |
| [GSM1129726](https://www.ncbi.nlm.nih.gov/geo/query/acc.cgi?acc=GSM1129726) | Carotid atherosclerotic samples 423 | Male | 58 |
| [GSM1129727](https://www.ncbi.nlm.nih.gov/geo/query/acc.cgi?acc=GSM1129727) | Carotid atherosclerotic samples 427 | Male | 64 |
| [GSM1129728](https://www.ncbi.nlm.nih.gov/geo/query/acc.cgi?acc=GSM1129728) | Carotid atherosclerotic samples 490 | Male | 59 |
| [GSM1129729](https://www.ncbi.nlm.nih.gov/geo/query/acc.cgi?acc=GSM1129729) | Carotid atherosclerotic samples 495 | Male | 75 |
| [GSM1129730](https://www.ncbi.nlm.nih.gov/geo/query/acc.cgi?acc=GSM1129730) | Carotid atherosclerotic samples 505 | Female | 68 |
| [GSM1129731](https://www.ncbi.nlm.nih.gov/geo/query/acc.cgi?acc=GSM1129731) | Carotid atherosclerotic samples 517 | Female | 69 |
| [GSM1129732](https://www.ncbi.nlm.nih.gov/geo/query/acc.cgi?acc=GSM1129732) | Carotid atherosclerotic samples 523 | Male | 68 |
| [GSM1129733](https://www.ncbi.nlm.nih.gov/geo/query/acc.cgi?acc=GSM1129733) | Carotid atherosclerotic samples 547 | Female | 62 |
| [GSM1129734](https://www.ncbi.nlm.nih.gov/geo/query/acc.cgi?acc=GSM1129734) | Carotid atherosclerotic samples 564 | Male | 71 |
