# Supplementary material for: Integrated Analysis of Gene Expression and Methylation Data to Identify Potential Biomarkers Related to Atherosclerosis Onset
Source: Oxid Med Cell Longev. 2022 Jul 22;2022:5493051. doi: 10.1155/2022/5493051 (PMC9338736; doi:10.1155/2022/5493051)
Supplement: Supplementary 5 — Table S2: the detailed CpG sites list. [file 5493051.f5.docx]

Table S2. The detailed CpG sites list

| ID | FDR | UCSC_RefGene_Name | UCSC_RefGene_Group | Relation_to_UCSC_CpG_Island |
| --- | --- | --- | --- | --- |
| cg00003625 | 9.62E-07 | WFIKKN2 | TSS200 | Open Sea |
| cg00005112 | 2.34E-07 | WWP2 | TSS200 | Open Sea |
| cg00007076 | 6.22E-10 | RRS1 | 1stExon | N_Shore |
| cg00014118 | 2.07E-09 | KIAA1751 | TSS1500 | S_Shore |
| cg00015639 | 2.98E-07 | NRL | TSS1500 | S_Shelf |
| cg00019196 | 1.97E-07 | SIAH3 | TSS1500 | Open Sea |
| cg00026033 | 2.42E-08 | IKZF4 | TSS200 | Open Sea |
| cg00028013 | 2.80E-09 | LOXL1 | TSS200 | Island |
| cg00032205 | 7.54E-07 | TSPYL5 | TSS200 | Island |
| cg00034769 | 3.92E-07 | C14orf64 | TSS200 | Open Sea |
| cg00035316 | 9.46E-08 | HOXD8 | TSS1500 | Island |
| cg00036871 | 2.07E-09 | OBSL1 | TSS1500 | S_Shore |
| cg00038167 | 4.32E-07 | PNPLA6 | TSS1500 | Island |
| cg00038584 | 9.53E-07 | INSL3 | TSS1500 | S_Shore |
| cg00039326 | 8.60E-07 | TRAPPC3 | TSS1500 | S_Shore |
| cg00039480 | 3.04E-07 | SLC47A2 | TSS1500 | S_Shelf |
| cg00040426 | 9.98E-07 | LCN1 | TSS200 | Open Sea |
| cg00041564 | 1.09E-07 | RCVRN | TSS1500 | S_Shore |
| cg00044097 | 6.63E-07 | FHL2 | 1stExon | Open Sea |
| cg00044665 | 1.45E-09 | CDH5 | TSS200 | Open Sea |
| cg00044729 | 7.88E-09 | ENTHD1 | TSS1500 | Open Sea |
| cg00047287 | 2.85E-09 | CPT1B | TSS200 | Island |
| cg00048759 | 4.32E-07 | STAG3 | TSS200 | Island |
| cg00054210 | 1.25E-07 | CYP7B1 | TSS1500 | Island |
| cg00056257 | 7.11E-08 | GRM7 | TSS1500 | N_Shore |
| cg00056754 | 2.14E-08 | DUSP27 | TSS200 | Open Sea |
| cg00059225 | 2.05E-07 | GLRA1 | 1stExon | Island |
| cg00062282 | 7.65E-09 | PLXNB2 | TSS1500 | S_Shore |
| cg00063773 | 5.73E-08 | PLS1 | TSS1500 | N_Shore |
| cg00066748 | 4.47E-07 | C8orf42 | TSS1500 | S_Shore |
| cg00071887 | 1.68E-08 | LOXL1 | TSS1500 | N_Shore |
| cg00074348 | 2.37E-07 | APLNR | TSS1500 | Open Sea |
| cg00076936 | 5.86E-07 | C20orf118 | TSS1500 | Open Sea |
| cg00085448 | 8.57E-07 | OAS2 | 1stExon | Open Sea |
| cg00091633 | 2.45E-08 | FSTL1 | TSS1500 | Island |
| cg00102955 | 7.99E-08 | CA14 | 1stExon | Open Sea |
| cg00103329 | 4.44E-08 | ETNK2 | TSS1500 | S_Shore |
| cg00110039 | 9.40E-09 | CECR4 | TSS1500 | N_Shore |
| cg00110785 | 7.84E-09 | IGFBP1 | TSS200 | N_Shore |
| cg00118507 | 1.13E-07 | MOV10 | TSS1500 | N_Shore |
| cg00119314 | 2.75E-07 | RSAD1 | TSS1500 | N_Shore |
| cg00120505 | 2.08E-07 | KIAA1432 | TSS1500 | Open Sea |
| cg00124708 | 4.91E-08 | ARMCX3 | TSS1500 | Open Sea |
| cg00142670 | 5.07E-07 | C6orf154 | TSS200 | Island |
| cg00145141 | 2.16E-07 | GGT6 | TSS200 | Open Sea |
| cg00145352 | 4.66E-08 | LOC284009 | TSS1500 | Open Sea |
| cg00149061 | 1.02E-10 | UGT2A3 | 1stExon | Open Sea |
| cg00153306 | 3.51E-08 | TMIE | TSS1500 | N_Shore |
| cg00165394 | 7.33E-10 | SPTBN5 | TSS1500 | Open Sea |
| cg00172849 | 2.32E-07 | COL11A1 | TSS1500 | Open Sea |
| cg00174500 | 8.89E-08 | CMTM5 | 1stExon | Open Sea |
| cg00175735 | 5.78E-08 | TMEM30C | TSS1500 | Open Sea |
| cg00181439 | 1.42E-07 | HTR1B | 1stExon | Island |
| cg00184203 | 9.90E-07 | FAM171B | TSS1500 | N_Shore |
| cg00185909 | 1.57E-07 | ZNF165 | TSS1500 | Open Sea |
| cg00187503 | 4.67E-10 | MBP | TSS1500 | Open Sea |
| cg00192882 | 4.62E-07 | MFAP4 | TSS1500 | Open Sea |
| cg00201534 | 5.24E-07 | KRTAP10-10 | TSS1500 | Open Sea |
| cg00203913 | 5.44E-07 | HS3ST3A1 | TSS1500 | S_Shore |
| cg00204748 | 3.92E-07 | FAR2 | 1stExon | Open Sea |
| cg00207280 | 8.78E-08 | TRIB1 | TSS1500 | N_Shore |
| cg00211489 | 3.99E-10 | MRPL42P5 | TSS1500 | Open Sea |
| cg00219151 | 3.58E-09 | SLC16A9 | TSS1500 | S_Shore |
| cg00226225 | 5.88E-07 | LOC728264 | TSS1500 | Open Sea |
| cg00226984 | 1.82E-07 | KLHL30 | TSS200 | N_Shore |
| cg00233594 | 9.28E-07 | LOC151162 | TSS1500 | Open Sea |
| cg00238249 | 3.69E-08 | C6orf57 | TSS1500 | N_Shore |
| cg00240880 | 1.01E-07 | WISP2 | TSS1500 | Open Sea |
| cg00242839 | 1.43E-07 | RBM17 | TSS1500 | N_Shore |
| cg00250483 | 3.62E-07 | RGS5 | TSS200 | Open Sea |
| cg00253681 | 6.42E-10 | ART4 | TSS200 | Open Sea |
| cg00257271 | 1.99E-10 | C5orf39 | TSS1500 | Island |
| cg00261569 | 2.18E-07 | C3orf1 | TSS1500 | N_Shore |
| cg00261668 | 1.80E-07 | HSPB7 | TSS1500 | Open Sea |
| cg00270625 | 3.36E-07 | CPT1B | 1stExon | Island |
| cg00273068 | 2.99E-07 | EMILIN3 | TSS1500 | Island |
| cg00273449 | 3.78E-08 | C2orf65 | TSS200 | Island |
| cg00273657 | 1.30E-07 | LOC100169752 | TSS1500 | Open Sea |
| cg00280345 | 8.32E-09 | GPT | TSS1500 | S_Shore |
| cg00283876 | 9.52E-08 | BCL7C | TSS1500 | S_Shore |
| cg00287016 | 2.30E-07 | MIR25 | TSS1500 | Open Sea |
| cg00298399 | 5.25E-07 | PADI6 | TSS1500 | Open Sea |
| cg00298951 | 2.14E-07 | CMKLR1 | TSS1500 | Open Sea |
| cg00310463 | 2.31E-07 | KY | TSS1500 | S_Shore |
| cg00319595 | 7.76E-07 | BCAR1 | TSS1500 | S_Shore |
| cg00319692 | 1.17E-07 | ATP6V0D2 | TSS200 | Open Sea |
| cg00332048 | 1.94E-07 | HDAC9 | TSS1500 | Open Sea |
| cg00332146 | 9.72E-07 | PCDH24 | TSS1500 | Open Sea |
| cg00341387 | 4.22E-07 | C18orf25 | TSS1500 | N_Shore |
| cg00345862 | 8.06E-07 | LECT1 | 1stExon | Island |
| cg00346446 | 1.34E-08 | MRPL24 | TSS1500 | S_Shore |
| cg00350296 | 5.10E-08 | CD248 | TSS1500 | S_Shore |
| cg00351443 | 1.06E-08 | NEURL | TSS1500 | N_Shore |
| cg00351472 | 5.96E-09 | MIR23B | TSS1500 | N_Shore |
| cg00351537 | 5.71E-08 | ZNF358 | TSS1500 | N_Shore |
| cg00357958 | 5.46E-07 | GPR81 | 1stExon | Open Sea |
| cg00358609 | 1.19E-08 | EPS8L1 | TSS1500 | Open Sea |
| cg00366413 | 9.54E-07 | MIR141 | TSS1500 | Open Sea |
| cg00372375 | 1.69E-08 | MFHAS1 | 1stExon | Island |
| cg00373499 | 5.60E-07 | PKD2L2 | TSS1500 | N_Shore |
| cg00375025 | 2.80E-07 | KCNAB1 | 1stExon | Open Sea |
| cg00379467 | 3.71E-08 | EDNRA | TSS200 | N_Shore |
| cg00386405 | 2.23E-07 | ZNF536 | TSS200 | N_Shelf |
| cg00388154 | 1.50E-08 | CALML4 | TSS1500 | Open Sea |
| cg00395486 | 6.10E-08 | PHACTR2 | TSS1500 | N_Shore |
| cg00396991 | 7.22E-07 | HSPA6 | TSS200 | Island |
| cg00401972 | 7.10E-11 | CDH5 | TSS200 | Open Sea |
| cg00404025 | 4.85E-07 | WDFY4 | TSS1500 | Open Sea |
| cg00413617 | 3.74E-07 | KCNC1 | TSS200 | Island |
| cg00415993 | 1.37E-08 | F2RL2 | 1stExon | Open Sea |
| cg00416645 | 1.43E-08 | IGFBP5 | TSS1500 | S_Shore |
| cg00417131 | 3.71E-08 | SFRS13B | TSS1500 | S_Shore |
| cg00421624 | 7.22E-08 | SLC27A3 | TSS1500 | N_Shore |
| cg00424169 | 1.16E-09 | C13orf26 | 1stExon | Open Sea |
| cg00426659 | 1.56E-07 | ANKS1B | 1stExon | Open Sea |
| cg00428179 | 1.50E-07 | ZNF438 | TSS1500 | S_Shore |
| cg00430287 | 7.61E-08 | FRK | TSS1500 | Open Sea |
| cg00431114 | 6.29E-07 | TTPAL | TSS1500 | N_Shore |
| cg00434119 | 2.27E-07 | DGKG | TSS1500 | S_Shore |
| cg00444390 | 7.70E-08 | OR10A6 | TSS200 | Open Sea |
| cg00445443 | 2.06E-09 | HOXA2 | TSS1500 | Island |
| cg00448191 | 1.47E-07 | NKX6-3 | TSS200 | S_Shore |
| cg00449201 | 7.15E-07 | CPT1C | TSS1500 | Island |
| cg00449482 | 7.37E-08 | EPN1 | TSS200 | S_Shore |
| cg00449767 | 3.13E-08 | EXOC3L2 | TSS200 | Island |
| cg00457913 | 3.89E-07 | C6orf138 | TSS200 | S_Shore |
| cg00458754 | 6.95E-07 | LDB3 | TSS1500 | Open Sea |
| cg00463502 | 5.00E-07 | WFIKKN2 | TSS200 | Open Sea |
| cg00481644 | 5.52E-09 | LOC254559 | TSS200 | Island |
| cg00496967 | 1.01E-08 | CDRT4 | TSS1500 | Open Sea |
| cg00498305 | 1.25E-07 | SLC18A2 | TSS1500 | N_Shore |
| cg00500789 | 1.07E-07 | C1orf210 | TSS200 | Open Sea |
| cg00503866 | 5.31E-07 | MACF1 | TSS1500 | N_Shore |
| cg00504595 | 6.75E-07 | TNFRSF19 | 1stExon | Open Sea |
| cg00505073 | 1.50E-07 | C20orf165 | TSS1500 | N_Shelf |
| cg00507815 | 4.32E-09 | GLO1 | TSS1500 | S_Shore |
| cg00509187 | 6.46E-08 | ENOX1 | TSS1500 | S_Shore |
| cg00511334 | 1.75E-07 | NRN1L | 1stExon | Island |
| cg00514241 | 3.85E-08 | MN1 | 1stExon | Island |
| cg00515954 | 3.85E-07 | ANKS1B | TSS200 | Open Sea |
| cg00516678 | 1.91E-08 | AQP1 | TSS1500 | Open Sea |
| cg00516867 | 2.71E-07 | OPN1SW | TSS1500 | Open Sea |
| cg00519208 | 7.49E-07 | EVI5 | TSS1500 | Open Sea |
| cg00520380 | 2.65E-07 | LIMK2 | TSS1500 | Open Sea |
| cg00522588 | 5.05E-08 | MLL2 | 1stExon | Open Sea |
| cg00524179 | 1.63E-07 | HOXA3 | TSS1500 | N_Shelf |
| cg00524271 | 9.45E-09 | PLCL1 | TSS1500 | N_Shore |
| cg00527825 | 4.92E-07 | LOXL1 | TSS1500 | N_Shore |
| cg00530688 | 4.20E-07 | BTBD11 | TSS200 | N_Shore |
| cg00531453 | 6.24E-07 | TMEM229A | TSS1500 | S_Shore |
| cg00533390 | 7.62E-07 | HNRNPU | TSS1500 | S_Shore |
| cg00534655 | 3.80E-07 | PURA | TSS1500 | Island |
| cg00544337 | 1.69E-08 | DYDC2 | TSS1500 | Island |
| cg00545229 | 2.35E-07 | C1QTNF7 | TSS200 | Open Sea |
| cg00548268 | 4.03E-07 | NPTX2 | TSS1500 | Island |
| cg00549758 | 2.77E-08 | C20orf185 | TSS1500 | Open Sea |
| cg00555456 | 3.70E-07 | ACN9 | TSS1500 | N_Shore |
| cg00561287 | 2.37E-07 | CA14 | TSS200 | Open Sea |
| cg00562553 | 6.60E-08 | HOXA4 | 1stExon | Island |
| cg00569747 | 1.49E-09 | ABI3BP | TSS1500 | Open Sea |
| cg00571819 | 1.46E-07 | LOC100272217 | TSS1500 | S_Shore |
| cg00573124 | 4.82E-07 | C9orf69 | TSS1500 | S_Shore |
| cg00583733 | 6.02E-09 | ALDOA | TSS1500 | N_Shore |
| cg00584815 | 5.54E-08 | C1orf223 | TSS1500 | S_Shelf |
| cg00586700 | 2.20E-07 | FCGRT | TSS1500 | N_Shore |
| cg00595472 | 5.66E-08 | DKFZP434K028 | TSS200 | Open Sea |
| cg00597076 | 6.09E-07 | MYO1C | 1stExon | S_Shore |
| cg00599770 | 2.08E-08 | HOXA7 | TSS1500 | N_Shore |
| cg00606312 | 5.94E-08 | KMO | TSS200 | Open Sea |
| cg00606739 | 1.82E-07 | LOC440040 | TSS200 | Open Sea |
| cg00607755 | 1.12E-07 | PKDCC | TSS1500 | Open Sea |
| cg00610310 | 5.83E-08 | LOC146336 | TSS200 | Island |
| cg00615469 | 2.01E-08 | TSPAN5 | TSS1500 | S_Shore |
| cg00616135 | 4.99E-07 | LACTB | TSS1500 | N_Shore |
| cg00620224 | 1.44E-07 | NUBPL | TSS1500 | Open Sea |
| cg00622010 | 3.26E-07 | AQP1 | TSS1500 | Open Sea |
| cg00626515 | 2.15E-07 | PMEPA1 | TSS1500 | S_Shore |
| cg00629688 | 3.25E-07 | GRIA4 | TSS1500 | N_Shore |
| cg00636072 | 9.63E-07 | CCDC85A | TSS1500 | N_Shore |
| cg00658007 | 2.82E-07 | RD3 | 1stExon | Open Sea |
| cg00658082 | 2.03E-08 | RTKN | TSS1500 | Island |
| cg00661320 | 1.00E-08 | SPOCK2 | TSS200 | S_Shore |
| cg00664406 | 6.03E-07 | GRM2 | TSS1500 | Island |
| cg00665650 | 6.96E-09 | PAIP2 | TSS1500 | N_Shore |
| cg00666746 | 4.86E-07 | SYDE1 | TSS1500 | N_Shore |
| cg00666842 | 8.40E-08 | SMYD1 | TSS1500 | Open Sea |
| cg00667343 | 4.43E-07 | C1orf101 | TSS1500 | N_Shore |
| cg00670913 | 1.59E-07 | CNTN5 | TSS1500 | N_Shore |
| cg00687135 | 9.84E-07 | ZNF385D | TSS200 | Open Sea |
| cg00694520 | 1.48E-07 | KCNE4 | TSS200 | N_Shore |
| cg00697970 | 5.80E-07 | TDRD9 | TSS1500 | N_Shore |
| cg00701946 | 1.73E-07 | LOC100302652 | TSS200 | Island |
| cg00713275 | 8.20E-09 | ALDOA | TSS1500 | N_Shore |
| cg00729875 | 3.69E-07 | PALMD | 1stExon | Open Sea |
| cg00730561 | 2.26E-07 | SEC31B | TSS200 | Island |
| cg00735454 | 1.69E-07 | LOC121838 | TSS1500 | Open Sea |
| cg00736958 | 3.39E-07 | C3orf74 | TSS1500 | Open Sea |
| cg00741410 | 2.90E-07 | TAS1R1 | TSS200 | S_Shore |
| cg00743788 | 6.81E-07 | MAPK12 | TSS1500 | S_Shore |
| cg00748958 | 4.00E-08 | LRRC37B | 1stExon | Open Sea |
| cg00754253 | 4.74E-08 | HRASLS5 | TSS1500 | S_Shore |
| cg00754357 | 5.80E-08 | ZCCHC11 | TSS1500 | S_Shore |
| cg00755588 | 5.74E-08 | C9orf172 | TSS1500 | N_Shore |
| cg00757070 | 5.95E-07 | UPK2 | 1stExon | Open Sea |
| cg00762681 | 2.07E-07 | CCDC160 | TSS1500 | N_Shore |
| cg00764612 | 2.45E-07 | C1orf51 | TSS200 | S_Shore |
| cg00768409 | 2.15E-08 | BNC1 | TSS1500 | S_Shore |
| cg00771553 | 7.79E-08 | VANGL1 | TSS1500 | N_Shore |
| cg00771653 | 9.71E-07 | CHCHD10 | TSS1500 | S_Shore |
| cg00780666 | 1.07E-08 | C14orf179 | TSS1500 | N_Shore |
| cg00785193 | 5.75E-07 | CRIM1 | TSS1500 | N_Shore |
| cg00792251 | 1.23E-07 | CAPN2 | TSS1500 | Open Sea |
| cg00805496 | 3.02E-09 | LOC728640 | TSS200 | Open Sea |
| cg00807586 | 4.18E-07 | DLEC1 | 1stExon | Island |
| cg00808132 | 5.07E-07 | PSMB11 | TSS200 | Open Sea |
| cg00808555 | 7.04E-09 | ARL5C | TSS1500 | S_Shore |
| cg00808648 | 5.23E-08 | PACS2 | TSS1500 | N_Shore |
| cg00817502 | 1.55E-08 | SEC24D | TSS1500 | S_Shore |
| cg00819233 | 3.75E-07 | DDR2 | TSS1500 | Open Sea |
| cg00819362 | 4.05E-09 | CLIP3 | TSS1500 | S_Shore |
| cg00828689 | 2.82E-07 | PIGZ | TSS1500 | Island |
| cg00828709 | 1.10E-07 | C10orf71 | TSS200 | Open Sea |
| cg00830029 | 1.93E-07 | NRIP2 | 1stExon | Open Sea |
| cg00850538 | 3.40E-07 | CRIM1 | TSS1500 | N_Shore |
| cg00852595 | 4.82E-07 | ANKS1B | 1stExon | Open Sea |
| cg00855466 | 1.13E-07 | ALDH3A1 | TSS1500 | S_Shelf |
| cg00862588 | 2.79E-08 | HLA-DMB | TSS1500 | Open Sea |
| cg00863716 | 6.63E-07 | AKR1C2 | TSS1500 | Open Sea |
| cg00869941 | 2.09E-07 | LPP | TSS1500 | Open Sea |
| cg00870242 | 4.82E-07 | C8orf74 | 1stExon | Open Sea |
| cg00885918 | 1.78E-07 | MYLK2 | TSS200 | N_Shelf |
| cg00894103 | 2.84E-09 | ALPK3 | TSS1500 | N_Shore |
| cg00898111 | 8.41E-07 | BTBD17 | TSS1500 | S_Shelf |
| cg00898447 | 7.84E-08 | BCAR1 | TSS1500 | S_Shore |
| cg00898480 | 2.03E-09 | ST6GALNAC1 | TSS1500 | Open Sea |
| cg00901161 | 2.11E-07 | PEBP4 | TSS1500 | Open Sea |
| cg00908927 | 6.35E-07 | ODZ4 | TSS1500 | S_Shore |
| cg00910127 | 6.71E-07 | AARS2 | TSS1500 | S_Shore |
| cg00911398 | 7.81E-08 | ARSF | TSS1500 | Open Sea |
| cg00919591 | 2.32E-07 | BCR | 1stExon | Island |
| cg00920668 | 3.88E-08 | LOC148696 | TSS1500 | Open Sea |
| cg00921097 | 1.95E-07 | GPR37L1 | TSS1500 | Open Sea |
| cg00921973 | 4.52E-07 | DPM2 | TSS1500 | S_Shore |
| cg00922841 | 7.07E-08 | SPRR1A | TSS1500 | Open Sea |
| cg00924052 | 6.55E-08 | LOC402644 | TSS1500 | Open Sea |
| cg00927035 | 6.72E-08 | SLC35C1 | TSS200 | N_Shore |
| cg00927666 | 2.07E-07 | LOC402644 | TSS1500 | Open Sea |
| cg00928596 | 3.04E-07 | MIR365-1 | TSS200 | Open Sea |
| cg00930606 | 1.19E-09 | PATZ1 | 1stExon | N_Shore |
| cg00933411 | 7.60E-07 | DLC1 | TSS1500 | Open Sea |
| cg00940502 | 2.82E-07 | RNF125 | TSS200 | N_Shore |
| cg00940560 | 5.12E-07 | MBOAT4 | 1stExon | Open Sea |
| cg00940577 | 2.10E-07 | GOSR1 | TSS1500 | N_Shore |
| cg00945108 | 9.32E-07 | C1orf158 | TSS200 | Open Sea |
| cg00950343 | 4.35E-07 | C5orf13 | 1stExon | Open Sea |
| cg00951869 | 1.75E-07 | ADCY4 | TSS1500 | S_Shore |
| cg00954382 | 9.37E-07 | NPY2R | TSS1500 | N_Shore |
| cg00955686 | 1.78E-08 | MAP7D1 | TSS1500 | N_Shore |
| cg00955780 | 1.91E-08 | SLC2A14 | TSS1500 | Island |
| cg00959922 | 4.04E-08 | FOXL1 | TSS1500 | N_Shore |
| cg00966405 | 5.20E-10 | MIR589 | TSS1500 | S_Shore |
| cg00969405 | 2.25E-07 | HOXA5 | TSS1500 | Island |
| cg00974204 | 1.33E-07 | UACA | TSS1500 | S_Shore |
| cg00974629 | 7.26E-07 | EDNRA | TSS200 | N_Shore |
| cg00980784 | 6.57E-07 | ABI3 | TSS200 | Open Sea |
| cg01000188 | 5.27E-07 | QRFP | 1stExon | Open Sea |
| cg01006126 | 1.49E-07 | C1QTNF8 | TSS1500 | S_Shelf |
| cg01015175 | 5.80E-07 | BCO2 | TSS1500 | Open Sea |
| cg01016169 | 7.60E-07 | LETMD1 | TSS1500 | N_Shore |
| cg01020079 | 3.36E-08 | TMPRSS12 | 1stExon | Island |
| cg01022370 | 2.56E-07 | MFHAS1 | 1stExon | Island |
| cg01024247 | 1.76E-09 | MIR589 | TSS200 | S_Shore |
| cg01024668 | 1.64E-07 | C15orf28 | TSS1500 | Open Sea |
| cg01032675 | 5.01E-07 | GNA15 | 1stExon | Open Sea |
| cg01034396 | 2.03E-07 | SMAD9 | TSS1500 | S_Shore |
| cg01035170 | 3.25E-09 | C5orf49 | TSS1500 | S_Shore |
| cg01035826 | 2.86E-08 | CHEK1 | TSS1500 | N_Shore |
| cg01047613 | 1.07E-09 | EFCAB8 | TSS200 | Open Sea |
| cg01055594 | 2.86E-07 | WDR35 | TSS1500 | S_Shore |
| cg01057196 | 2.14E-07 | LOC284688 | TSS1500 | Open Sea |
| cg01065977 | 5.55E-08 | ISYNA1 | TSS1500 | S_Shore |
| cg01068601 | 3.02E-07 | NRN1L | TSS200 | Island |
| cg01068808 | 4.83E-11 | GLT25D2 | TSS1500 | S_Shore |
| cg01073837 | 5.82E-07 | PLEKHA2 | TSS1500 | N_Shore |
| cg01074797 | 2.53E-07 | PDZK1IP1 | 1stExon | Open Sea |
| cg01076495 | 1.29E-09 | ECM1 | 1stExon | Open Sea |
| cg01079738 | 5.61E-07 | C1orf14 | 1stExon | Island |
| cg01081346 | 8.26E-07 | CPT1B | TSS200 | S_Shore |
| cg01084740 | 8.33E-07 | TMEM30B | 1stExon | S_Shore |
| cg01087239 | 3.74E-07 | KIAA1683 | TSS200 | Open Sea |
| cg01097725 | 4.55E-07 | PCDHB4 | 1stExon | Island |
| cg01100043 | 4.17E-09 | RAMP1 | TSS1500 | N_Shore |
| cg01101472 | 2.17E-08 | SAMD9 | TSS1500 | Open Sea |
| cg01105427 | 5.10E-07 | CPZ | TSS1500 | N_Shore |
| cg01110418 | 1.70E-07 | FLJ44635 | TSS1500 | Open Sea |
| cg01115432 | 5.46E-07 | NCAPG2 | TSS1500 | S_Shore |
| cg01118900 | 8.31E-07 | ARMCX4 | TSS1500 | N_Shore |
| cg01128109 | 3.11E-07 | LRRC8B | TSS1500 | N_Shore |
| cg01132484 | 1.87E-09 | TBL1XR1 | TSS1500 | S_Shore |
| cg01137471 | 2.97E-09 | DMRT1 | TSS1500 | N_Shore |
| cg01139861 | 9.72E-07 | IKZF1 | TSS1500 | Island |
| cg01141812 | 3.43E-07 | CLVS2 | TSS1500 | N_Shore |
| cg01155092 | 6.38E-08 | TMEM100 | TSS1500 | Open Sea |
| cg01156077 | 2.14E-07 | LOC100132215 | TSS1500 | Island |
| cg01157280 | 3.99E-07 | RAB17 | 1stExon | Open Sea |
| cg01178040 | 2.44E-09 | TNXB | TSS1500 | Open Sea |
| cg01184539 | 3.63E-07 | FAM26D | TSS1500 | Open Sea |
| cg01185080 | 7.43E-07 | ZNF710 | TSS1500 | Island |
| cg01187920 | 5.79E-07 | CARTPT | 1stExon | Island |
| cg01189606 | 2.63E-07 | PLXNB1 | TSS1500 | S_Shore |
| cg01197294 | 1.23E-09 | MYL2 | TSS1500 | Open Sea |
| cg01197831 | 8.96E-10 | FBP2 | 1stExon | Open Sea |
| cg01198994 | 5.43E-07 | MXRA8 | TSS1500 | S_Shelf |
| cg01201802 | 1.32E-07 | KIAA1462 | TSS1500 | S_Shore |
| cg01203113 | 1.93E-07 | DNASE1 | TSS200 | Open Sea |
| cg01203550 | 2.26E-09 | MYH7 | TSS200 | Open Sea |
| cg01205267 | 1.15E-08 | LRCH1 | TSS1500 | N_Shore |
| cg01208614 | 1.12E-07 | FCHSD1 | TSS1500 | S_Shore |
| cg01209566 | 6.89E-07 | LOC157381 | TSS200 | Open Sea |
| cg01211097 | 9.14E-07 | USP10 | TSS1500 | N_Shore |
| cg01212326 | 2.14E-07 | POU2AF1 | TSS200 | Open Sea |
| cg01214063 | 6.72E-07 | PPP1R3A | TSS200 | Open Sea |
| cg01214458 | 5.01E-07 | MN1 | 1stExon | Island |
| cg01217984 | 7.17E-11 | HOXA2 | TSS1500 | Island |
| cg01218148 | 8.45E-07 | ENHO | TSS1500 | S_Shore |
| cg01226614 | 3.92E-07 | SERP2 | TSS1500 | Island |
| cg01227558 | 7.94E-10 | ODZ2 | TSS1500 | Open Sea |
| cg01237056 | 3.62E-08 | SCUBE1 | TSS1500 | Island |
| cg01239922 | 4.08E-08 | LRRC14B | TSS1500 | N_Shore |
| cg01243586 | 3.18E-09 | SAA2 | TSS1500 | Open Sea |
| cg01244346 | 5.37E-10 | TET3 | TSS1500 | Open Sea |
| cg01257309 | 3.71E-10 | DLEU7 | TSS1500 | S_Shore |
| cg01260308 | 4.07E-07 | KLHDC2 | TSS1500 | N_Shore |
| cg01264539 | 5.94E-08 | PREPL | 1stExon | N_Shore |
| cg01266287 | 9.43E-07 | RRAD | TSS1500 | S_Shore |
| cg01267165 | 1.84E-07 | LITAF | TSS1500 | S_Shore |
| cg01268381 | 3.14E-07 | DSCAM | TSS1500 | S_Shore |
| cg01273384 | 2.09E-07 | MIR1257 | TSS1500 | Open Sea |
| cg01277890 | 5.43E-09 | LOC389333 | TSS1500 | S_Shore |
| cg01278291 | 2.52E-09 | DOK3 | 1stExon | Open Sea |
| cg01287088 | 6.18E-08 | PFN3 | 1stExon | Island |
| cg01287342 | 1.43E-07 | TTPA | TSS1500 | S_Shore |
| cg01292722 | 8.90E-08 | CLRN1 | 1stExon | Open Sea |
| cg01295399 | 7.85E-08 | SLC18A3 | 1stExon | Island |
| cg01296593 | 3.92E-07 | FOXC2 | TSS1500 | Island |
| cg01298514 | 6.04E-07 | VEGFA | TSS1500 | N_Shore |
| cg01298758 | 5.11E-07 | USP47 | TSS1500 | N_Shore |
| cg01305539 | 2.79E-07 | DAB2IP | TSS1500 | Open Sea |
| cg01306688 | 3.38E-07 | GPR81 | TSS200 | Open Sea |
| cg01309726 | 1.08E-07 | LOC100270710 | TSS200 | S_Shelf |
| cg01311222 | 5.49E-09 | HEG1 | TSS1500 | S_Shore |
| cg01312858 | 4.03E-08 | CCDC85A | TSS1500 | N_Shore |
| cg01314252 | 7.77E-07 | IL17RE | TSS1500 | Open Sea |
| cg01320573 | 8.90E-08 | CUTA | TSS1500 | S_Shore |
| cg01323274 | 1.28E-08 | TMEM188 | TSS1500 | N_Shore |
| cg01323381 | 2.30E-07 | HOXA5 | TSS1500 | Island |
| cg01324261 | 3.44E-07 | SCRG1 | 1stExon | Open Sea |
| cg01329690 | 4.67E-07 | DSCR9 | TSS1500 | Open Sea |
| cg01335126 | 7.95E-07 | NPW | TSS1500 | N_Shore |
| cg01337047 | 5.57E-07 | DSG1 | TSS1500 | Open Sea |
| cg01341572 | 1.99E-08 | HNF1A | TSS200 | Island |
| cg01353646 | 5.35E-07 | AGAP2 | TSS1500 | Island |
| cg01353907 | 1.22E-07 | GPR133 | TSS200 | Open Sea |
| cg01355757 | 3.66E-09 | TET3 | TSS1500 | Open Sea |
| cg01357713 | 9.22E-08 | PRKAG3 | TSS200 | Open Sea |
| cg01359469 | 4.10E-07 | TRIP4 | TSS1500 | N_Shore |
| cg01370449 | 4.35E-09 | HOXA5 | TSS200 | Island |
| cg01378812 | 1.49E-07 | DDX52 | TSS1500 | S_Shore |
| cg01387036 | 1.57E-07 | CCL1 | TSS200 | Open Sea |
| cg01391299 | 1.92E-07 | SPINK5L3 | TSS200 | Open Sea |
| cg01394199 | 3.56E-08 | HNF1A | TSS200 | Island |
| cg01400468 | 9.07E-08 | GPR75 | TSS1500 | S_Shore |
| cg01401534 | 3.51E-07 | C6orf201 | TSS200 | Island |
| cg01404163 | 3.39E-07 | TOX3 | TSS200 | S_Shore |
| cg01406381 | 1.50E-07 | SLC1A5 | TSS200 | N_Shelf |
| cg01409985 | 1.21E-08 | SRPRB | TSS1500 | N_Shore |
| cg01420388 | 5.24E-07 | FBXO44 | TSS1500 | Island |
| cg01422009 | 4.14E-07 | MPG | TSS1500 | N_Shore |
| cg01424889 | 2.38E-07 | AVIL | TSS1500 | Open Sea |
| cg01429391 | 1.57E-08 | MYH7 | TSS200 | Open Sea |
| cg01444808 | 1.06E-07 | SEPT8 | TSS1500 | S_Shore |
| cg01446692 | 5.13E-08 | CER1 | TSS1500 | Open Sea |
| cg01447951 | 7.54E-07 | FBXL22 | TSS200 | N_Shelf |
| cg01448881 | 1.52E-07 | ATP1A2 | TSS1500 | Open Sea |
| cg01452110 | 1.05E-07 | HIST1H3A | TSS1500 | N_Shore |
| cg01459854 | 1.26E-08 | FLJ43390 | TSS1500 | N_Shore |
| cg01464052 | 3.15E-07 | C1orf51 | TSS200 | S_Shore |
| cg01467047 | 5.04E-08 | FOXL1 | TSS1500 | N_Shore |
| cg01467503 | 1.42E-07 | C1orf144 | TSS1500 | N_Shore |
| cg01471111 | 4.01E-07 | SLC6A13 | TSS1500 | Open Sea |
| cg01471654 | 5.03E-07 | PIGS | TSS1500 | S_Shore |
| cg01480180 | 3.22E-07 | FZD1 | 1stExon | Island |
| cg01486558 | 2.28E-09 | TRAK1 | 1stExon | Open Sea |
| cg01492523 | 2.89E-08 | DCHS2 | TSS1500 | S_Shore |
| cg01494441 | 7.59E-07 | CERKL | TSS1500 | S_Shore |
| cg01510588 | 1.08E-10 | C14orf183 | 1stExon | Open Sea |
| cg01512532 | 5.47E-07 | FSCN2 | TSS1500 | Island |
| cg01526089 | 7.28E-07 | P2RX1 | 1stExon | Open Sea |
| cg01534799 | 4.39E-08 | FLYWCH1 | TSS1500 | N_Shore |
| cg01535312 | 9.38E-07 | MOCS1 | TSS1500 | S_Shore |
| cg01536682 | 3.78E-08 | EARS2 | TSS1500 | S_Shore |
| cg01542384 | 6.03E-07 | GNAI2 | TSS200 | Open Sea |
| cg01548463 | 2.40E-07 | FAM180A | TSS200 | Open Sea |
| cg01554451 | 1.04E-10 | GLIPR1 | TSS1500 | Open Sea |
| cg01557754 | 8.33E-07 | FGF11 | TSS200 | N_Shore |
| cg01558401 | 3.89E-07 | LOC728264 | TSS200 | Open Sea |
| cg01560885 | 9.31E-07 | FAM123B | TSS1500 | S_Shore |
| cg01565130 | 2.34E-07 | MPZL1 | TSS1500 | N_Shore |
| cg01565314 | 1.08E-07 | EXOC3L2 | TSS200 | Island |
| cg01565608 | 2.11E-07 | ADAM32 | TSS1500 | N_Shore |
| cg01565774 | 2.24E-08 | IKZF4 | TSS200 | Open Sea |
| cg01566404 | 1.10E-08 | ETNK2 | TSS1500 | S_Shore |
| cg01568784 | 1.04E-07 | RAB38 | TSS200 | S_Shore |
| cg01569083 | 1.51E-07 | TNF | TSS200 | Open Sea |
| cg01573501 | 4.30E-09 | HADH | TSS1500 | N_Shore |
| cg01579001 | 8.02E-07 | ITGA7 | TSS1500 | N_Shelf |
| cg01580681 | 4.47E-10 | HAND2 | 1stExon | N_Shore |
| cg01583915 | 9.84E-07 | C20orf46 | 1stExon | N_Shore |
| cg01583940 | 7.17E-07 | PAQR6 | TSS1500 | S_Shelf |
| cg01584348 | 4.66E-07 | LOC91948 | TSS200 | Open Sea |
| cg01596520 | 7.84E-08 | PRKACA | TSS200 | N_Shelf |
| cg01598007 | 1.29E-08 | DUSP27 | TSS1500 | Open Sea |
| cg01599742 | 4.52E-07 | MOCS1 | TSS1500 | S_Shore |
| cg01603095 | 8.73E-07 | SLC2A13 | TSS1500 | S_Shore |
| cg01630479 | 3.23E-07 | MIR1306 | TSS1500 | N_Shore |
| cg01634664 | 2.18E-07 | C14orf159 | TSS1500 | Open Sea |
| cg01651284 | 2.92E-07 | CHRNA9 | TSS200 | Open Sea |
| cg01656133 | 9.45E-08 | USH1C | TSS1500 | S_Shore |
| cg01662455 | 5.89E-07 | PLVAP | 1stExon | Island |
| cg01664864 | 1.48E-08 | DIO3 | TSS200 | Island |
| cg01669366 | 6.30E-07 | TSC22D4 | TSS1500 | S_Shore |
| cg01670862 | 1.23E-08 | LOC641746 | TSS1500 | Open Sea |
| cg01671575 | 7.22E-07 | PGBD5 | TSS200 | Open Sea |
| cg01682285 | 4.02E-07 | TSHR | 1stExon | S_Shore |
| cg01695023 | 4.55E-07 | P2RX6P | TSS1500 | N_Shore |
| cg01697163 | 1.89E-07 | SH3BP1 | TSS1500 | N_Shore |
| cg01699740 | 5.52E-07 | GRAMD1B | TSS200 | Open Sea |
| cg01706698 | 1.99E-07 | CETP | TSS1500 | Open Sea |
| cg01709312 | 2.87E-07 | LOC282997 | TSS200 | N_Shore |
| cg01713348 | 8.70E-07 | C16orf91 | TSS1500 | N_Shore |
| cg01733599 | 2.52E-08 | LIPC | TSS1500 | Open Sea |
| cg01734240 | 3.54E-08 | NANOG | TSS1500 | Open Sea |
| cg01738638 | 1.05E-07 | APLP2 | TSS1500 | N_Shore |
| cg01739167 | 5.90E-08 | CHRNE | 1stExon | S_Shore |
| cg01751802 | 4.37E-07 | KANK2 | TSS1500 | S_Shore |
| cg01754756 | 3.58E-07 | C10orf116 | TSS200 | Island |
| cg01755541 | 3.03E-07 | TPPP3 | TSS1500 | Island |
| cg01760189 | 1.47E-10 | ACACA | TSS1500 | Open Sea |
| cg01765053 | 5.99E-07 | C20orf185 | 1stExon | Open Sea |
| cg01767116 | 8.58E-07 | FBXL22 | TSS1500 | N_Shelf |
| cg01768926 | 6.62E-07 | ZNF397 | TSS1500 | N_Shore |
| cg01769243 | 1.10E-10 | GFAP | TSS200 | S_Shelf |
| cg01780585 | 3.83E-07 | EIF2C1 | TSS1500 | N_Shore |
| cg01780943 | 7.56E-07 | TRAF5 | TSS1500 | N_Shore |
| cg01789576 | 7.90E-07 | GJC2 | TSS1500 | Open Sea |
| cg01791648 | 3.29E-07 | SENP7 | TSS1500 | S_Shore |
| cg01796223 | 4.38E-07 | CPA4 | 1stExon | Open Sea |
| cg01797043 | 1.23E-08 | RPL3L | TSS200 | Open Sea |
| cg01799015 | 1.88E-07 | PALM | TSS1500 | N_Shore |
| cg01804429 | 6.04E-07 | YTHDC1 | TSS1500 | S_Shore |
| cg01805732 | 3.25E-07 | PXDN | TSS1500 | Island |
| cg01811796 | 3.36E-08 | STMN1 | TSS1500 | Island |
| cg01812328 | 7.45E-09 | ACADL | TSS1500 | S_Shore |
| cg01812577 | 2.42E-07 | CHRM1 | TSS200 | N_Shore |
| cg01812894 | 2.61E-07 | ALDH1A1 | TSS1500 | Open Sea |
| cg01819502 | 3.86E-08 | GGT6 | TSS1500 | Open Sea |
| cg01821854 | 3.63E-07 | SFRP2 | TSS1500 | N_Shore |
| cg01827761 | 6.98E-07 | KLHDC7A | TSS1500 | N_Shelf |
| cg01835620 | 3.34E-08 | CHRNE | TSS1500 | S_Shelf |
| cg01835725 | 4.46E-07 | PCBP4 | TSS1500 | Open Sea |
| cg01848457 | 2.73E-10 | EID3 | 1stExon | Island |
| cg01867395 | 3.52E-07 | PAX6 | TSS200 | Island |
| cg01869896 | 8.03E-07 | MFSD2A | TSS1500 | N_Shore |
| cg01874730 | 2.11E-07 | CX3CL1 | 1stExon | Open Sea |
| cg01875467 | 5.99E-07 | CSF1R | TSS200 | Open Sea |
| cg01882880 | 5.23E-08 | HOXB2 | TSS1500 | N_Shelf |
| cg01885503 | 6.26E-07 | IRX3 | TSS1500 | Island |
| cg01888869 | 7.19E-07 | ITGAX | TSS200 | Open Sea |
| cg01896579 | 7.79E-08 | G3BP2 | TSS1500 | S_Shore |
| cg01897323 | 2.55E-07 | TXNDC6 | TSS1500 | S_Shore |
| cg01898628 | 1.07E-08 | SOST | TSS200 | S_Shelf |
| cg01901466 | 1.67E-08 | CHGA | TSS1500 | N_Shore |
| cg01901788 | 2.21E-07 | MAP1LC3A | TSS1500 | N_Shore |
| cg01903327 | 7.34E-07 | ACYP1 | TSS1500 | Open Sea |
| cg01904985 | 3.83E-07 | CPLX3 | TSS1500 | Open Sea |
| cg01905102 | 6.93E-07 | PPPDE1 | TSS1500 | N_Shore |
| cg01908020 | 8.43E-07 | SHF | TSS1500 | S_Shelf |
| cg01912455 | 3.93E-08 | SMARCB1 | TSS1500 | N_Shore |
| cg01919204 | 6.03E-09 | GPT | TSS1500 | N_Shore |
| cg01923099 | 4.47E-07 | KCNQ1DN | TSS1500 | N_Shore |
| cg01940297 | 5.33E-08 | MIR1207 | TSS1500 | Open Sea |
| cg01942372 | 3.20E-07 | LIMS1 | TSS1500 | Open Sea |
| cg01942646 | 8.71E-10 | NR0B2 | TSS200 | Open Sea |
| cg01944288 | 6.76E-08 | NTNG2 | TSS1500 | N_Shore |
| cg01947936 | 1.07E-08 | RAPSN | 1stExon | Open Sea |
| cg01954057 | 2.92E-07 | BCKDK | TSS1500 | N_Shore |
| cg01963297 | 4.52E-07 | KCNN3 | 1stExon | Open Sea |
| cg01963696 | 3.25E-09 | ELANE | TSS1500 | N_Shore |
| cg01966160 | 8.92E-08 | PEBP4 | TSS1500 | Open Sea |
| cg01970336 | 4.47E-07 | EPS15 | TSS1500 | S_Shore |
| cg01971181 | 4.82E-08 | MFSD1 | TSS1500 | N_Shore |
| cg01972843 | 9.59E-08 | CECR1 | TSS200 | Open Sea |
| cg01976034 | 7.23E-09 | ASB14 | TSS1500 | Open Sea |
| cg01981960 | 3.24E-07 | MYL10 | TSS1500 | Open Sea |
| cg01985396 | 2.52E-08 | DAAM2 | 1stExon | S_Shore |
| cg01987202 | 3.37E-07 | C15orf52 | TSS200 | Open Sea |
| cg02002258 | 6.93E-07 | OTOF | TSS200 | Open Sea |
| cg02006647 | 2.02E-07 | EXOC3L2 | TSS1500 | Island |
| cg02007567 | 2.65E-09 | FAM18A | TSS200 | Island |
| cg02009601 | 1.51E-07 | C8orf83 | TSS1500 | S_Shore |
| cg02013018 | 2.11E-11 | WDR66 | TSS1500 | N_Shore |
| cg02014107 | 2.82E-07 | DLX2 | TSS1500 | S_Shore |
| cg02026204 | 3.45E-07 | OSM | TSS200 | Open Sea |
| cg02026498 | 5.64E-09 | GNG8 | TSS1500 | Island |
| cg02026535 | 1.03E-07 | ZNF358 | TSS1500 | N_Shore |
| cg02027561 | 2.40E-08 | NCOA7 | TSS1500 | N_Shore |
| cg02034887 | 2.21E-07 | SLC15A2 | TSS200 | Open Sea |
| cg02037013 | 5.01E-07 | MC4R | 1stExon | Open Sea |
| cg02045294 | 1.10E-07 | MAMSTR | TSS1500 | N_Shore |
| cg02047547 | 1.83E-07 | ITPRIPL2 | 1stExon | Island |
| cg02056098 | 5.77E-10 | SCN9A | TSS1500 | S_Shore |
| cg02058108 | 4.13E-07 | SSPN | TSS200 | N_Shore |
| cg02059080 | 1.22E-08 | ZNF428 | TSS1500 | S_Shore |
| cg02059082 | 7.08E-08 | ZNF513 | TSS1500 | S_Shore |
| cg02069715 | 6.69E-07 | GABRG3 | TSS1500 | Island |
| cg02076607 | 1.76E-07 | C1QTNF8 | TSS200 | S_Shelf |
| cg02078525 | 2.32E-09 | CDH5 | TSS200 | Open Sea |
| cg02081065 | 4.53E-09 | LEAP2 | TSS1500 | Open Sea |
| cg02086355 | 2.03E-07 | C6orf126 | TSS1500 | N_Shore |
| cg02092616 | 1.88E-07 | INF2 | TSS1500 | N_Shore |
| cg02102075 | 7.07E-07 | RAB11FIP3 | TSS1500 | N_Shore |
| cg02102684 | 6.62E-07 | VASN | TSS1500 | N_Shore |
| cg02103401 | 8.09E-07 | EPM2AIP1 | 1stExon | N_Shore |
| cg02106466 | 3.44E-07 | PCDH12 | TSS1500 | Open Sea |
| cg02106682 | 3.45E-07 | HOXA5 | TSS1500 | Island |
| cg02109484 | 2.42E-08 | C2orf65 | TSS200 | Island |
| cg02124724 | 7.09E-07 | COL18A1 | TSS1500 | N_Shore |
| cg02131465 | 2.90E-09 | RD3 | TSS1500 | Open Sea |
| cg02137183 | 2.39E-08 | AGK | TSS1500 | N_Shore |
| cg02144874 | 4.77E-07 | KLHL30 | TSS200 | N_Shore |
| cg02159913 | 1.08E-07 | JAG2 | TSS1500 | Open Sea |
| cg02161701 | 6.73E-08 | KIF7 | TSS1500 | S_Shore |
| cg02176069 | 1.19E-07 | PYGM | 1stExon | Open Sea |
| cg02181639 | 2.63E-07 | NGB | 1stExon | Island |
| cg02197387 | 1.16E-07 | TCEA2 | TSS200 | Island |
| cg02208529 | 1.06E-07 | DBN1 | TSS1500 | Open Sea |
| cg02211646 | 2.37E-07 | FOXP2 | 1stExon | Open Sea |
| cg02218324 | 4.94E-07 | RSPH6A | 1stExon | N_Shore |
| cg02219026 | 3.45E-07 | ZNF589 | TSS1500 | N_Shore |
| cg02219197 | 8.91E-08 | PROC | TSS1500 | S_Shore |
| cg02221520 | 8.36E-07 | TLR6 | TSS1500 | Open Sea |
| cg02225599 | 1.67E-08 | HOXA2 | TSS1500 | Island |
| cg02226939 | 5.70E-08 | BLMH | TSS1500 | S_Shore |
| cg02241055 | 6.38E-07 | CLDN11 | 1stExon | Island |
| cg02247178 | 8.70E-09 | C13orf26 | TSS200 | Open Sea |
| cg02248486 | 1.30E-07 | HOXA5 | 1stExon | Island |
| cg02249228 | 8.18E-07 | LOC340357 | TSS200 | Open Sea |
| cg02249841 | 4.07E-07 | CD81 | TSS1500 | N_Shore |
| cg02253552 | 4.50E-08 | ZNF248 | TSS1500 | S_Shore |
| cg02254574 | 3.67E-07 | ACCN1 | 1stExon | N_Shore |
| cg02257057 | 1.24E-08 | MLH3 | TSS1500 | S_Shore |
| cg02269083 | 7.53E-07 | ALPK2 | TSS1500 | Open Sea |
| cg02274550 | 9.55E-07 | ACTG2 | TSS200 | Open Sea |
| cg02276070 | 5.07E-07 | ARSI | TSS1500 | S_Shore |
| cg02286663 | 2.94E-07 | TFR2 | TSS200 | Open Sea |
| cg02287710 | 3.76E-08 | DIO3 | TSS200 | Island |
| cg02303016 | 3.90E-07 | CPA1 | TSS200 | N_Shore |
| cg02315696 | 2.63E-08 | LOC157381 | TSS1500 | Open Sea |
| cg02317832 | 1.92E-08 | ASB18 | 1stExon | Open Sea |
| cg02318535 | 4.23E-08 | PPM1E | TSS1500 | N_Shore |
| cg02325250 | 5.88E-07 | CSF2 | TSS200 | Open Sea |
| cg02327997 | 7.46E-08 | ADAM12 | TSS200 | Island |
| cg02330106 | 4.76E-09 | MGMT | TSS1500 | N_Shore |
| cg02332117 | 6.39E-08 | S100A2 | TSS1500 | Open Sea |
| cg02335376 | 7.64E-07 | GDPD3 | TSS200 | Open Sea |
| cg02339542 | 4.50E-08 | CLLU1OS | TSS1500 | Open Sea |
| cg02341556 | 8.95E-07 | BCL9L | TSS1500 | S_Shore |
| cg02346062 | 9.24E-10 | GPLD1 | TSS1500 | N_Shelf |
| cg02346282 | 7.75E-07 | BTF3 | TSS1500 | N_Shore |
| cg02357257 | 4.00E-07 | THAP3 | TSS200 | Island |
| cg02363950 | 8.09E-07 | CRABP2 | TSS1500 | S_Shore |
| cg02369725 | 2.67E-08 | PDE11A | 1stExon | N_Shore |
| cg02370734 | 1.12E-09 | PDE7A | TSS1500 | Open Sea |
| cg02380585 | 2.59E-07 | SLFN13 | TSS1500 | Island |
| cg02382109 | 5.46E-07 | PEBP4 | TSS200 | Open Sea |
| cg02382666 | 5.26E-09 | CALD1 | TSS1500 | Open Sea |
| cg02383173 | 4.19E-08 | PDLIM5 | TSS1500 | N_Shore |
| cg02400942 | 9.12E-07 | CSPG4 | TSS1500 | Open Sea |
| cg02402423 | 2.76E-08 | C19orf54 | 1stExon | N_Shore |
| cg02404377 | 1.71E-08 | NAV2 | TSS1500 | Open Sea |
| cg02413187 | 3.88E-07 | MUM1 | TSS200 | S_Shore |
| cg02420724 | 9.66E-07 | COL5A2 | TSS1500 | Open Sea |
| cg02424654 | 1.77E-07 | S100Z | TSS200 | Open Sea |
| cg02432101 | 3.41E-07 | TCEB3CL | TSS1500 | S_Shore |
| cg02433785 | 6.79E-08 | RALA | TSS1500 | N_Shore |
| cg02441090 | 2.52E-08 | OXCT1 | TSS1500 | S_Shore |
| cg02456552 | 2.73E-07 | FDX1L | TSS1500 | S_Shore |
| cg02458483 | 2.53E-08 | KIAA1522 | TSS1500 | N_Shore |
| cg02468966 | 6.79E-08 | PMEPA1 | TSS1500 | S_Shore |
| cg02473540 | 2.44E-07 | ZNF135 | TSS200 | Island |
| cg02475695 | 3.76E-07 | NHLRC4 | TSS1500 | S_Shore |
| cg02481669 | 2.24E-07 | MCCD1 | TSS200 | Open Sea |
| cg02485345 | 6.60E-09 | SLC25A34 | TSS1500 | S_Shelf |
| cg02491017 | 4.41E-11 | HOXC4 | TSS1500 | N_Shore |
| cg02492708 | 3.47E-09 | RPL3L | TSS1500 | Open Sea |
| cg02494904 | 6.37E-08 | SMOX | TSS1500 | N_Shore |
| cg02503376 | 2.72E-07 | TNK1 | TSS1500 | N_Shore |
| cg02503815 | 3.52E-08 | DPY19L2P2 | TSS1500 | S_Shore |
| cg02506717 | 8.73E-07 | CREB5 | TSS200 | Open Sea |
| cg02506908 | 4.16E-07 | HPD | TSS1500 | Open Sea |
| cg02507296 | 3.71E-07 | C10orf95 | TSS200 | S_Shore |
| cg02513485 | 5.93E-08 | SPTBN1 | TSS1500 | N_Shore |
| cg02515467 | 6.38E-08 | THUMPD1 | TSS1500 | S_Shore |
| cg02516134 | 5.92E-08 | CALD1 | TSS1500 | Open Sea |
| cg02516530 | 1.93E-07 | C14orf72 | TSS1500 | Open Sea |
| cg02523400 | 1.28E-09 | SERPIND1 | TSS200 | Open Sea |
| cg02529358 | 6.81E-07 | LOC728264 | TSS200 | Open Sea |
| cg02531437 | 7.50E-07 | PRLHR | TSS200 | Island |
| cg02531794 | 2.01E-07 | SRCRB4D | TSS200 | Open Sea |
| cg02533339 | 9.40E-09 | MIR365-1 | TSS200 | Open Sea |
| cg02536838 | 1.05E-08 | ANGPT1 | TSS200 | Open Sea |
| cg02555579 | 4.68E-07 | SLC25A18 | TSS200 | Open Sea |
| cg02558476 | 2.72E-07 | ZG16B | 1stExon | Open Sea |
| cg02558684 | 3.93E-09 | MIRLET7A1 | TSS1500 | Open Sea |
| cg02572606 | 9.46E-07 | THUMPD1 | TSS1500 | S_Shore |
| cg02580606 | 4.10E-07 | KRT33B | TSS1500 | Open Sea |
| cg02589501 | 4.89E-07 | USP46 | TSS1500 | N_Shore |
| cg02596089 | 1.12E-07 | CCDC9 | TSS1500 | N_Shore |
| cg02596994 | 8.64E-07 | HPD | TSS200 | Open Sea |
| cg02597299 | 8.56E-07 | GLRA1 | TSS200 | Island |
| cg02611848 | 7.29E-08 | C2orf65 | TSS1500 | Island |
| cg02613108 | 4.79E-07 | PCDH15 | TSS200 | Open Sea |
| cg02632362 | 1.70E-07 | EDARADD | TSS200 | N_Shore |
| cg02633229 | 6.94E-08 | C15orf23 | TSS200 | N_Shore |
| cg02640604 | 9.06E-08 | QPRT | TSS200 | Open Sea |
| cg02640638 | 1.72E-07 | TMEM229A | TSS200 | Island |
| cg02641539 | 3.32E-11 | TM7SF4 | TSS1500 | Open Sea |
| cg02642822 | 5.38E-07 | LOC404266 | TSS1500 | N_Shelf |
| cg02647628 | 2.75E-07 | FOXP2 | TSS1500 | Open Sea |
| cg02654291 | 1.85E-08 | C9orf64 | TSS1500 | Island |
| cg02656169 | 1.52E-08 | ANAPC5 | TSS1500 | S_Shelf |
| cg02658214 | 3.46E-07 | SDR9C7 | 1stExon | Open Sea |
| cg02660440 | 1.73E-07 | MIR199A1 | TSS200 | Open Sea |
| cg02661623 | 2.17E-11 | FLJ23834 | 1stExon | Open Sea |
| cg02662828 | 1.59E-07 | ZAR1 | 1stExon | Island |
| cg02670343 | 8.51E-10 | EPS15 | TSS1500 | Open Sea |
| cg02675652 | 9.03E-08 | CSRNP2 | TSS1500 | S_Shore |
| cg02677388 | 3.55E-07 | HIST1H3C | 1stExon | Island |
| cg02680566 | 5.98E-11 | OSR1 | TSS1500 | N_Shore |
| cg02699218 | 1.13E-07 | ANKRD43 | 1stExon | Island |
| cg02709052 | 7.69E-08 | TMEM135 | TSS1500 | N_Shore |
| cg02710173 | 1.55E-07 | FLNA | TSS1500 | S_Shore |
| cg02710860 | 1.34E-08 | MAPKBP1 | TSS1500 | N_Shore |
| cg02711608 | 4.95E-07 | SLC1A5 | 1stExon | N_Shelf |
| cg02713760 | 4.63E-08 | BNC1 | TSS1500 | S_Shore |
| cg02715546 | 5.15E-07 | PYGO1 | TSS200 | Island |
| cg02717339 | 4.31E-08 | ANKRD39 | TSS1500 | S_Shore |
| cg02725334 | 5.66E-07 | C22orf30 | TSS1500 | S_Shore |
| cg02726291 | 1.19E-07 | GP9 | TSS200 | Open Sea |
| cg02726898 | 4.60E-08 | C1QTNF8 | TSS1500 | S_Shelf |
| cg02727591 | 2.97E-07 | LOC400696 | TSS1500 | Open Sea |
| cg02735486 | 2.00E-07 | ANK2 | 1stExon | Open Sea |
| cg02737379 | 4.98E-07 | POPDC2 | TSS200 | Open Sea |
| cg02741548 | 6.70E-07 | GADL1 | TSS1500 | S_Shore |
| cg02745822 | 8.62E-07 | WISP1 | 1stExon | Open Sea |
| cg02746232 | 2.73E-08 | MAP2K4 | TSS1500 | N_Shore |
| cg02747210 | 2.19E-08 | F10 | TSS200 | S_Shelf |
| cg02769951 | 1.42E-11 | TMEM200A | TSS200 | Open Sea |
| cg02774160 | 1.06E-08 | GGT1 | TSS200 | Open Sea |
| cg02800334 | 7.11E-08 | ANXA13 | TSS1500 | Open Sea |
| cg02804722 | 1.01E-07 | EMILIN1 | TSS1500 | Open Sea |
| cg02812767 | 4.63E-07 | LOXL1 | TSS1500 | N_Shore |
| cg02827278 | 2.43E-07 | ORAI1 | TSS1500 | N_Shore |
| cg02828306 | 6.53E-07 | LOC221122 | TSS1500 | Open Sea |
| cg02831419 | 2.56E-08 | KCNE4 | TSS1500 | N_Shore |
| cg02838877 | 9.52E-07 | MBNL2 | TSS1500 | Open Sea |
| cg02839330 | 5.33E-07 | TRIM63 | TSS200 | Open Sea |
| cg02844647 | 5.85E-07 | CHN2 | TSS200 | Open Sea |
| cg02845063 | 5.74E-07 | FBXO40 | 1stExon | Open Sea |
| cg02849766 | 8.29E-07 | MIR1470 | TSS1500 | Open Sea |
| cg02855207 | 4.79E-08 | RELT | TSS1500 | N_Shore |
| cg02855432 | 4.91E-07 | ERGIC1 | TSS1500 | N_Shore |
| cg02860797 | 1.03E-07 | OGG1 | TSS1500 | Island |
| cg02861056 | 8.56E-07 | PLEK | 1stExon | Open Sea |
| cg02862867 | 4.34E-07 | IPO9 | TSS1500 | N_Shore |
| cg02867514 | 2.29E-09 | CCL5 | 1stExon | Open Sea |
| cg02871021 | 6.81E-07 | MRVI1 | TSS1500 | Open Sea |
| cg02871995 | 4.22E-08 | LAYN | TSS1500 | Island |
| cg02872476 | 4.31E-08 | DBNDD1 | TSS1500 | S_Shore |
| cg02878461 | 2.35E-08 | CLIP3 | TSS1500 | S_Shore |
| cg02880877 | 3.47E-08 | HDDC2 | TSS1500 | S_Shore |
| cg02882785 | 4.57E-07 | CLDN22 | TSS200 | Open Sea |
| cg02883147 | 8.39E-07 | LOC144571 | TSS200 | S_Shore |
| cg02885771 | 8.18E-07 | LTV1 | TSS1500 | N_Shore |
| cg02898721 | 2.01E-07 | RARA | TSS1500 | N_Shore |
| cg02900183 | 1.09E-07 | CIB3 | TSS200 | Open Sea |
| cg02905900 | 7.21E-07 | ACOT11 | TSS1500 | Open Sea |
| cg02907064 | 2.86E-07 | MIR199A1 | TSS200 | Open Sea |
| cg02910281 | 2.58E-07 | RHBDL2 | 1stExon | Open Sea |
| cg02912476 | 2.63E-08 | PRKCZ | TSS1500 | N_Shelf |
| cg02916118 | 1.01E-07 | SNORD41 | TSS200 | Open Sea |
| cg02920396 | 1.21E-09 | MRGPRF | TSS1500 | S_Shelf |
| cg02923831 | 2.31E-07 | C8orf86 | TSS1500 | Open Sea |
| cg02930888 | 8.38E-07 | RBM20 | TSS1500 | Island |
| cg02933362 | 1.62E-07 | RFX8 | TSS1500 | S_Shore |
| cg02943595 | 9.18E-08 | LOC143188 | TSS200 | Open Sea |
| cg02952809 | 4.27E-07 | C7orf68 | TSS1500 | N_Shore |
| cg02958004 | 7.52E-09 | CTAGE1 | TSS200 | Open Sea |
| cg02965411 | 2.36E-07 | TSPAN8 | TSS200 | Open Sea |
| cg02975223 | 6.34E-08 | TMPRSS11D | TSS1500 | Open Sea |
| cg02979520 | 5.70E-10 | PLXNB1 | TSS1500 | S_Shore |
| cg02983759 | 8.10E-09 | NCRNA00111 | TSS1500 | Open Sea |
| cg02994956 | 6.03E-08 | NEFH | 1stExon | Island |
| cg02996181 | 2.36E-07 | SIGIRR | TSS1500 | Island |
| cg03005293 | 2.33E-09 | RORC | TSS1500 | Open Sea |
| cg03017264 | 1.99E-07 | NFAM1 | TSS1500 | Open Sea |
| cg03018496 | 1.02E-07 | NR2F2 | TSS200 | S_Shelf |
| cg03018949 | 1.27E-07 | C10orf122 | 1stExon | Open Sea |
| cg03020006 | 4.40E-08 | ALS2CR11 | 1stExon | Island |
| cg03020208 | 5.20E-08 | AQP5 | TSS1500 | Island |
| cg03029255 | 5.67E-07 | C8orf31 | 1stExon | Open Sea |
| cg03029752 | 4.87E-08 | NLRX1 | TSS1500 | N_Shore |
| cg03031383 | 1.43E-09 | TMPRSS11E | TSS1500 | Open Sea |
| cg03032512 | 2.00E-07 | C1QTNF1 | TSS200 | Open Sea |
| cg03034540 | 9.65E-07 | ZDHHC1 | TSS1500 | S_Shore |
| cg03037030 | 2.51E-07 | TNF | TSS200 | Open Sea |
| cg03039701 | 3.52E-07 | SLC2A12 | TSS1500 | Open Sea |
| cg03043417 | 8.56E-07 | CD53 | 1stExon | Open Sea |
| cg03043911 | 7.20E-10 | KLHL4 | TSS1500 | Open Sea |
| cg03053826 | 7.78E-07 | NR2F2 | TSS1500 | S_Shelf |
| cg03058664 | 2.60E-07 | C8orf31 | TSS200 | Open Sea |
| cg03065625 | 1.40E-08 | LRP4 | TSS1500 | S_Shore |
| cg03068051 | 2.22E-08 | CACHD1 | TSS1500 | N_Shore |
| cg03078972 | 1.53E-08 | SNAI1 | TSS1500 | N_Shore |
| cg03096803 | 1.98E-09 | ST6GALNAC1 | TSS1500 | Open Sea |
| cg03100044 | 6.52E-07 | CHN2 | TSS200 | Open Sea |
| cg03100639 | 8.85E-08 | TGFB1I1 | TSS1500 | N_Shore |
| cg03101058 | 1.53E-07 | CPSF4L | TSS1500 | Open Sea |
| cg03102548 | 5.73E-08 | HIST1H2AE | TSS1500 | N_Shore |
| cg03111114 | 1.76E-07 | NDST1 | TSS1500 | Open Sea |
| cg03126199 | 1.25E-07 | ZNF486 | TSS1500 | Open Sea |
| cg03127558 | 1.48E-07 | MAMSTR | TSS1500 | Island |
| cg03133371 | 9.29E-09 | SFRP1 | TSS1500 | S_Shore |
| cg03136646 | 7.51E-09 | MMP23A | TSS1500 | Island |
| cg03138124 | 9.42E-07 | C1D | TSS1500 | Open Sea |
| cg03155027 | 4.93E-07 | CAMKV | TSS1500 | S_Shore |
| cg03157395 | 3.50E-09 | ABCG8 | TSS200 | Open Sea |
| cg03160508 | 6.54E-07 | RHOD | TSS1500 | N_Shore |
| cg03161410 | 6.44E-07 | CHST6 | TSS200 | Island |
| cg03163767 | 4.65E-08 | FAM96B | TSS1500 | Island |
| cg03173367 | 7.11E-07 | ANKRD39 | TSS1500 | S_Shore |
| cg03180302 | 4.85E-07 | TTR | 1stExon | Open Sea |
| cg03184776 | 7.33E-07 | MIR2117 | TSS200 | Open Sea |
| cg03192897 | 8.10E-10 | METTL11B | 1stExon | Open Sea |
| cg03207666 | 3.13E-07 | HOXA5 | TSS1500 | Island |
| cg03212674 | 1.05E-07 | CLK2 | TSS1500 | S_Shore |
| cg03214087 | 1.01E-07 | TMEM120A | TSS1500 | S_Shore |
| cg03221266 | 3.19E-07 | ACTA2 | TSS200 | Open Sea |
| cg03226054 | 4.17E-07 | DPCR1 | TSS1500 | Open Sea |
| cg03230175 | 3.48E-07 | GPD2 | TSS1500 | N_Shore |
| cg03231860 | 4.59E-09 | RICS | 1stExon | Open Sea |
| cg03232842 | 8.40E-07 | CETP | TSS1500 | Open Sea |
| cg03234702 | 4.66E-08 | HIST1H3E | 1stExon | Island |
| cg03237218 | 4.35E-07 | SLFN12 | TSS1500 | Open Sea |
| cg03240509 | 6.81E-07 | KCNA6 | TSS200 | N_Shore |
| cg03241461 | 1.93E-07 | SVIL | TSS1500 | Open Sea |
| cg03242666 | 2.66E-09 | PMP22 | 1stExon | S_Shelf |
| cg03243768 | 1.01E-09 | FLJ46111 | TSS1500 | S_Shore |
| cg03245641 | 3.40E-07 | GPHA2 | TSS200 | Open Sea |
| cg03257293 | 1.72E-08 | ITPKB | TSS1500 | S_Shore |
| cg03260781 | 7.21E-08 | HSPB7 | TSS1500 | Open Sea |
| cg03263514 | 6.97E-07 | GP1BB | TSS200 | Island |
| cg03265490 | 3.24E-07 | TCF23 | TSS200 | N_Shore |
| cg03284308 | 2.55E-07 | CENPN | TSS1500 | N_Shore |
| cg03289906 | 1.14E-07 | PPP1R9A | TSS1500 | S_Shore |
| cg03295928 | 6.66E-07 | RTKN | TSS1500 | Island |
| cg03297192 | 3.29E-08 | CCL11 | TSS1500 | Open Sea |
| cg03299329 | 1.25E-07 | FAM26D | TSS200 | Open Sea |
| cg03302448 | 1.14E-10 | CUGBP2 | TSS1500 | Open Sea |
| cg03306615 | 1.18E-08 | ASCL2 | TSS1500 | Island |
| cg03308494 | 7.45E-07 | SLC26A10 | TSS1500 | Island |
| cg03309770 | 9.12E-09 | FAM18A | 1stExon | Island |
| cg03314548 | 3.13E-07 | ZNF530 | TSS1500 | N_Shore |
| cg03319695 | 2.10E-10 | CDH20 | TSS1500 | Open Sea |
| cg03321133 | 5.76E-08 | HOXD8 | TSS1500 | Island |
| cg03327403 | 1.15E-07 | KRT8 | TSS200 | S_Shore |
| cg03350299 | 6.80E-07 | APOB | TSS200 | Island |
| cg03351894 | 6.50E-07 | ERAS | TSS1500 | S_Shore |
| cg03353445 | 2.85E-08 | GPRC5A | TSS1500 | N_Shore |
| cg03353971 | 9.85E-08 | TMEM98 | TSS1500 | N_Shore |
| cg03356778 | 1.08E-07 | POU2F3 | TSS1500 | Island |
| cg03359095 | 8.14E-07 | C20orf151 | TSS1500 | Open Sea |
| cg03359362 | 2.31E-09 | SLC1A5 | TSS1500 | N_Shore |
| cg03363565 | 1.99E-07 | RAB11FIP3 | TSS1500 | N_Shore |
| cg03370704 | 1.96E-07 | LOC728264 | TSS1500 | Open Sea |
| cg03370951 | 5.45E-07 | C10orf128 | TSS1500 | Open Sea |
| cg03375371 | 3.43E-07 | NFU1 | TSS1500 | S_Shore |
| cg03379797 | 7.16E-08 | NHSL1 | 1stExon | Open Sea |
| cg03383006 | 4.11E-10 | GFAP | TSS200 | S_Shelf |
| cg03386869 | 7.32E-08 | ITGBL1 | TSS200 | N_Shore |
| cg03389133 | 8.02E-07 | SSTR3 | TSS1500 | Open Sea |
| cg03389164 | 1.61E-09 | ASB2 | TSS200 | Open Sea |
| cg03389701 | 5.04E-09 | DLEU7 | TSS1500 | S_Shore |
| cg03390207 | 1.74E-07 | CORO6 | TSS1500 | N_Shore |
| cg03393223 | 3.92E-07 | LOC399959 | TSS1500 | Open Sea |
| cg03398481 | 8.77E-07 | TMC3 | TSS1500 | Open Sea |
| cg03401213 | 6.59E-07 | NME2 | TSS1500 | N_Shore |
| cg03404566 | 2.76E-08 | ALOX12 | TSS200 | Island |
| cg03408904 | 2.01E-07 | CECR2 | TSS200 | Open Sea |
| cg03409272 | 1.50E-07 | CCL14-CCL15 | TSS1500 | Open Sea |
| cg03419014 | 3.92E-07 | HLCS | TSS1500 | Island |
| cg03419151 | 3.98E-07 | XAF1 | TSS1500 | Open Sea |
| cg03422070 | 6.57E-09 | KCNQ1OT1 | TSS1500 | Island |
| cg03422911 | 9.56E-07 | RYR2 | TSS1500 | Island |
| cg03431846 | 3.83E-08 | REC8 | TSS200 | Island |
| cg03435439 | 1.56E-11 | PDE4B | TSS200 | Open Sea |
| cg03439703 | 2.41E-07 | PDE3B | TSS1500 | N_Shore |
| cg03440588 | 2.94E-07 | FOXD2 | TSS1500 | Island |
| cg03443467 | 8.50E-09 | SH3BP2 | TSS1500 | Open Sea |
| cg03444122 | 1.50E-09 | TRIML1 | 1stExon | Open Sea |
| cg03459531 | 2.24E-07 | EMILIN3 | TSS1500 | S_Shore |
| cg03462322 | 1.90E-07 | MPG | TSS1500 | N_Shore |
| cg03463818 | 5.37E-10 | TMEM67 | TSS1500 | N_Shore |
| cg03478497 | 2.09E-07 | C18orf34 | TSS1500 | S_Shore |
| cg03483632 | 1.14E-07 | FJX1 | TSS1500 | N_Shore |
| cg03486832 | 4.47E-08 | KDM2B | TSS1500 | S_Shore |
| cg03491584 | 7.55E-07 | JAK3 | TSS200 | S_Shore |
| cg03505148 | 5.95E-07 | AGAP2 | 1stExon | Island |
| cg03514239 | 6.70E-07 | S100A9 | TSS1500 | Open Sea |
| cg03514843 | 5.56E-08 | EMILIN1 | TSS1500 | Open Sea |
| cg03518138 | 3.17E-10 | KIAA0754 | TSS1500 | N_Shore |
| cg03523740 | 5.05E-07 | TXLNA | TSS1500 | N_Shore |
| cg03536657 | 4.75E-09 | C15orf28 | TSS1500 | Open Sea |
| cg03540912 | 5.86E-07 | SNORD71 | TSS1500 | Open Sea |
| cg03546977 | 1.87E-08 | CMYA5 | TSS200 | Open Sea |
| cg03550233 | 6.24E-07 | KRT19 | TSS1500 | S_Shore |
| cg03552103 | 8.36E-08 | SEPT10 | TSS1500 | Island |
| cg03553386 | 7.23E-10 | IGF2 | TSS200 | Open Sea |
| cg03553576 | 3.94E-08 | LRRC14B | TSS1500 | N_Shore |
| cg03560685 | 5.04E-09 | MBP | TSS1500 | Open Sea |
| cg03562120 | 6.37E-09 | WISP2 | 1stExon | Open Sea |
| cg03562528 | 1.14E-10 | ASB2 | TSS200 | Open Sea |
| cg03565281 | 2.11E-08 | MLN | TSS200 | Open Sea |
| cg03566787 | 2.15E-07 | CUGBP1 | TSS1500 | Open Sea |
| cg03569412 | 4.06E-07 | MBD3 | TSS1500 | S_Shore |
| cg03589001 | 5.54E-08 | MORF4L1 | TSS1500 | N_Shore |
| cg03589230 | 1.77E-08 | MYOM2 | TSS1500 | Open Sea |
| cg03591467 | 9.57E-07 | FAP | TSS1500 | Open Sea |
| cg03592051 | 8.36E-07 | GSG1L | TSS1500 | Open Sea |
| cg03599338 | 4.73E-07 | SUSD2 | TSS200 | Open Sea |
| cg03605365 | 2.52E-07 | INADL | TSS1500 | N_Shore |
| cg03611487 | 7.28E-07 | LOC100126784 | TSS1500 | S_Shore |
| cg03618211 | 6.76E-07 | NHEDC1 | TSS1500 | S_Shore |
| cg03619586 | 2.99E-07 | CLIC1 | TSS1500 | N_Shelf |
| cg03630088 | 1.00E-07 | CPXM2 | TSS200 | S_Shore |
| cg03635171 | 6.47E-15 | IGFL2 | TSS1500 | Open Sea |
| cg03640944 | 7.74E-07 | ITPRIP | TSS200 | N_Shelf |
| cg03648724 | 1.62E-07 | C22orf34 | TSS1500 | Open Sea |
| cg03649589 | 5.07E-11 | CSGALNACT1 | TSS1500 | S_Shore |
| cg03650550 | 9.23E-07 | MIR575 | TSS1500 | Open Sea |
| cg03650851 | 6.16E-08 | C20orf107 | TSS1500 | Open Sea |
| cg03664443 | 7.47E-07 | MC2R | TSS1500 | Open Sea |
| cg03665785 | 4.16E-08 | C22orf31 | TSS1500 | Open Sea |
| cg03667871 | 6.04E-07 | NEK7 | TSS1500 | N_Shore |
| cg03675171 | 6.07E-07 | F10 | TSS200 | Open Sea |
| cg03677069 | 4.23E-07 | SNCG | 1stExon | Open Sea |
| cg03681383 | 3.19E-07 | IFLTD1 | TSS200 | Open Sea |
| cg03699566 | 9.25E-08 | FOLR1 | 1stExon | Open Sea |
| cg03699904 | 3.79E-10 | SLC2A2 | TSS1500 | N_Shore |
| cg03700944 | 1.43E-07 | PLA2G7 | TSS1500 | S_Shore |
| cg03703171 | 1.23E-08 | IL1RN | TSS1500 | Open Sea |
| cg03704645 | 7.89E-09 | SMYD1 | 1stExon | Open Sea |
| cg03705396 | 4.46E-07 | MAP6D1 | TSS1500 | Island |
| cg03705912 | 8.72E-07 |  | 1stExon | Island |
| cg03719040 | 5.00E-07 | TMEM128 | TSS1500 | S_Shore |
| cg03723510 | 7.75E-08 | C5orf39 | TSS200 | Island |
| cg03728296 | 6.99E-07 | SLC26A10 | TSS1500 | Island |
| cg03729889 | 1.21E-07 | MYO18B | TSS200 | Open Sea |
| cg03730969 | 8.65E-07 | METAP1 | TSS1500 | N_Shore |
| cg03731740 | 1.94E-08 | YTHDF2 | TSS1500 | N_Shore |
| cg03743584 | 5.30E-07 | PRAP1 | 1stExon | Open Sea |
| cg03744763 | 7.04E-09 | HOXA5 | TSS1500 | Island |
| cg03752885 | 3.82E-07 | DAPK3 | 1stExon | N_Shore |
| cg03760483 | 3.92E-08 | ALOX12 | TSS200 | Island |
| cg03762994 | 1.43E-08 | ALOX12 | TSS200 | Island |
| cg03771844 | 1.19E-07 | PRO1768 | TSS1500 | Open Sea |
| cg03771939 | 8.97E-07 | TBC1D12 | TSS1500 | N_Shore |
| cg03782584 | 7.00E-07 | SPOPL | TSS1500 | N_Shore |
| cg03785076 | 2.93E-07 | SNED1 | TSS1500 | N_Shore |
| cg03785728 | 3.35E-07 | BANF2 | TSS200 | Open Sea |
| cg03790427 | 1.94E-07 | C8orf74 | TSS200 | Open Sea |
| cg03795847 | 3.30E-07 | ZNF167 | TSS200 | N_Shore |
| cg03814957 | 1.31E-07 | CSF1R | TSS200 | Open Sea |
| cg03818303 | 2.63E-07 | KCNMB3 | TSS200 | Open Sea |
| cg03818307 | 6.22E-08 | ECM1 | 1stExon | Open Sea |
| cg03819243 | 2.10E-07 | PLCD1 | TSS1500 | S_Shore |
| cg03821689 | 5.93E-07 | FXYD6 | TSS1500 | S_Shore |
| cg03826976 | 9.31E-07 | CYB5R2 | TSS1500 | Island |
| cg03832197 | 1.18E-07 | AOX2P | TSS200 | Open Sea |
| cg03837313 | 5.93E-07 | SLC43A3 | TSS1500 | S_Shore |
| cg03838769 | 2.15E-07 | SPOCK3 | TSS1500 | S_Shore |
| cg03839161 | 4.80E-07 | SSTR5 | TSS200 | Island |
| cg03844121 | 1.36E-08 | ZFPM1 | TSS1500 | N_Shore |
| cg03849707 | 1.56E-07 | FEM1B | TSS1500 | N_Shore |
| cg03852144 | 1.18E-07 | GLRX | TSS1500 | Open Sea |
| cg03862705 | 7.95E-07 | NAT8B | TSS200 | Open Sea |
| cg03871354 | 8.11E-08 | SCARA5 | TSS1500 | Open Sea |
| cg03875996 | 2.53E-10 | RXFP1 | TSS1500 | Open Sea |
| cg03876697 | 1.73E-07 | IL17B | TSS1500 | Open Sea |
| cg03883256 | 8.73E-07 | USP2 | TSS1500 | S_Shore |
| cg03888814 | 7.56E-07 | DNAH7 | TSS1500 | S_Shore |
| cg03893150 | 5.97E-07 | TNFRSF25 | TSS200 | S_Shore |
| cg03894990 | 1.85E-08 | SPATS1 | TSS1500 | Open Sea |
| cg03900284 | 2.25E-07 | QRFP | TSS1500 | Open Sea |
| cg03908904 | 8.03E-07 | CAPS | TSS200 | N_Shore |
| cg03916225 | 7.08E-08 | CHI3L2 | TSS1500 | Open Sea |
| cg03930004 | 1.69E-07 | FASTK | TSS1500 | Island |
| cg03938110 | 1.09E-07 | NCRNA00110 | TSS200 | Open Sea |
| cg03943509 | 3.70E-08 | PYGM | TSS1500 | N_Shelf |
| cg03943605 | 6.63E-07 | DUPD1 | TSS1500 | Open Sea |
| cg03946671 | 1.32E-07 | TCEA2 | TSS200 | Island |
| cg03950476 | 4.29E-08 | ZCCHC11 | TSS1500 | S_Shore |
| cg03958883 | 2.44E-08 | ARHGEF17 | 1stExon | S_Shore |
| cg03958979 | 6.26E-07 | NR2E1 | TSS1500 | Island |
| cg03960066 | 5.76E-07 | C1orf51 | 1stExon | S_Shore |
| cg03962200 | 1.20E-08 | INPP5J | TSS1500 | Open Sea |
| cg03967293 | 5.31E-07 | LAMB3 | TSS1500 | Open Sea |
| cg03967327 | 1.83E-09 | ALDH5A1 | TSS1500 | N_Shore |
| cg03972466 | 5.57E-07 | C16orf72 | TSS1500 | Island |
| cg03979241 | 6.91E-08 | EPB49 | 1stExon | S_Shelf |
| cg03980224 | 6.46E-07 | RAI14 | TSS1500 | Open Sea |
| cg03985459 | 1.78E-07 | ZFAND3 | TSS1500 | N_Shore |
| cg03985801 | 1.35E-09 | LGR6 | TSS1500 | N_Shore |
| cg03992976 | 1.56E-07 | FOXRED2 | TSS1500 | S_Shore |
| cg03996539 | 5.90E-07 | FAM198B | TSS1500 | Open Sea |
| cg03997643 | 2.22E-07 | RNU6ATAC | TSS1500 | S_Shore |
| cg03998066 | 5.75E-07 | TGFB1I1 | TSS1500 | N_Shore |
| cg04004590 | 2.10E-08 | PRKG1 | TSS1500 | N_Shore |
| cg04004830 | 5.22E-08 | SDPR | 1stExon | Open Sea |
| cg04011712 | 2.64E-07 | OR6C70 | 1stExon | Open Sea |
| cg04018325 | 4.67E-08 | MIR193B | TSS200 | S_Shore |
| cg04020908 | 5.88E-09 | NAGLU | TSS1500 | N_Shore |
| cg04021557 | 7.24E-08 | HIST1H4B | TSS1500 | S_Shore |
| cg04025965 | 7.48E-08 | FASTK | TSS1500 | Island |
| cg04027302 | 4.18E-08 | LGALS8 | TSS1500 | N_Shore |
| cg04027736 | 1.15E-10 | HOXA2 | TSS1500 | Island |
| cg04039397 | 8.44E-08 | CD96 | TSS200 | Open Sea |
| cg04041000 | 1.45E-08 | PTH1R | TSS200 | Open Sea |
| cg04044664 | 4.53E-07 | ANKRD43 | 1stExon | Island |
| cg04046364 | 9.29E-09 | AVIL | TSS1500 | Open Sea |
| cg04048339 | 6.16E-08 | MCCD1 | TSS200 | Open Sea |
| cg04062119 | 5.53E-08 | MIR1289-2 | TSS1500 | Open Sea |
| cg04063235 | 9.15E-07 | MIRLET7A3 | TSS200 | Open Sea |
| cg04064028 | 8.53E-07 | BBS5 | TSS1500 | N_Shore |
| cg04067612 | 3.63E-07 | C1QTNF7 | TSS200 | Open Sea |
| cg04086834 | 1.91E-10 | SMAP1 | TSS1500 | N_Shore |
| cg04093149 | 8.31E-07 | SLC6A9 | TSS1500 | S_Shore |
| cg04101806 | 2.44E-08 | AFF3 | TSS1500 | S_Shore |
| cg04103514 | 5.69E-07 | EMR2 | TSS200 | Open Sea |
| cg04103993 | 1.58E-08 | DNAH7 | TSS1500 | S_Shore |
| cg04112102 | 1.17E-08 | SLC39A10 | TSS1500 | N_Shore |
| cg04114655 | 8.71E-07 | ACCN4 | TSS1500 | N_Shore |
| cg04118119 | 9.14E-07 | ESD | TSS1500 | S_Shore |
| cg04129469 | 1.54E-07 | PAX7 | TSS1500 | N_Shore |
| cg04140974 | 2.09E-07 | RNF144B | TSS1500 | N_Shore |
| cg04141648 | 4.64E-07 | CTNNA3 | TSS1500 | Open Sea |
| cg04153489 | 2.76E-07 | ANK1 | TSS1500 | Island |
| cg04154027 | 9.12E-08 | CMYA5 | TSS200 | Open Sea |
| cg04168474 | 5.07E-07 | LOC146336 | TSS200 | Island |
| cg04177015 | 7.32E-07 | MC1R | 1stExon | Island |
| cg04189723 | 2.99E-07 | ZXDB | 1stExon | S_Shelf |
| cg04193901 | 4.01E-07 | WISP2 | TSS200 | Open Sea |
| cg04203702 | 6.17E-08 | MIR572 | TSS1500 | N_Shore |
| cg04206967 | 1.45E-08 | TRHR | TSS1500 | Open Sea |
| cg04210922 | 5.11E-09 | GPRASP2 | TSS1500 | N_Shore |
| cg04211581 | 6.22E-10 | ESR1 | 1stExon | Open Sea |
| cg04218760 | 4.34E-09 | TMEM72 | TSS200 | Open Sea |
| cg04220416 | 5.36E-07 | C14orf180 | TSS200 | Open Sea |
| cg04221167 | 1.45E-07 | AMMECR1 | 1stExon | Open Sea |
| cg04225775 | 7.11E-07 | HMHB1 | 1stExon | Open Sea |
| cg04226536 | 2.68E-07 | LAYN | TSS200 | Island |
| cg04226745 | 1.68E-08 | C17orf67 | TSS1500 | Open Sea |
| cg04228042 | 2.19E-10 | ART4 | TSS200 | Open Sea |
| cg04237609 | 1.38E-08 | TMEM8B | TSS1500 | N_Shore |
| cg04250451 | 4.27E-07 | RUNX3 | TSS1500 | Island |
| cg04259168 | 2.80E-08 | PLIN4 | 1stExon | Open Sea |
| cg04259907 | 3.05E-07 | LYPD3 | TSS200 | S_Shore |
| cg04261607 | 5.50E-07 | SLC25A45 | TSS1500 | N_Shelf |
| cg04264633 | 6.54E-07 | BFSP1 | TSS1500 | Open Sea |
| cg04265051 | 3.34E-07 | LRP5 | TSS1500 | N_Shore |
| cg04265576 | 9.97E-07 | HOXA6 | TSS200 | Island |
| cg04268405 | 1.96E-07 | CHST3 | TSS1500 | N_Shore |
| cg04270402 | 7.48E-09 | PON2 | TSS1500 | S_Shore |
| cg04279139 | 1.45E-07 | LOC100133893 | TSS1500 | Open Sea |
| cg04279801 | 1.72E-07 | RNF112 | TSS200 | Open Sea |
| cg04282029 | 1.35E-10 | SV2A | TSS1500 | Open Sea |
| cg04289036 | 2.82E-08 | C1orf210 | 1stExon | Open Sea |
| cg04290171 | 1.93E-09 | CD46 | TSS1500 | N_Shore |
| cg04292941 | 5.93E-07 | DNHD1 | TSS200 | Open Sea |
| cg04292976 | 5.71E-07 | SLC26A9 | TSS1500 | Open Sea |
| cg04308089 | 3.63E-07 | NR2F2 | TSS200 | S_Shelf |
| cg04308185 | 5.26E-08 | ORMDL3 | TSS1500 | S_Shore |
| cg04312620 | 8.06E-07 | SALL4 | TSS200 | S_Shore |
| cg04318619 | 2.27E-07 | ABR | 1stExon | Open Sea |
| cg04319606 | 1.35E-07 | C2orf70 | TSS200 | Island |
| cg04322718 | 2.48E-07 | ATF6B | TSS1500 | N_Shore |
| cg04325632 | 6.25E-07 | ADRA2C | TSS1500 | Island |
| cg04328562 | 3.84E-07 | CNN1 | 1stExon | Open Sea |
| cg04333296 | 2.83E-07 | LOC653653 | TSS200 | Open Sea |
| cg04337854 | 9.05E-07 | BVES | TSS1500 | S_Shore |
| cg04338134 | 3.11E-07 | FAM96B | TSS1500 | N_Shore |
| cg04343883 | 6.35E-09 | LGR6 | TSS1500 | N_Shore |
| cg04354393 | 1.44E-07 | SLFN13 | TSS200 | N_Shore |
| cg04359602 | 2.42E-08 | NDRG2 | TSS1500 | S_Shore |
| cg04361266 | 3.16E-07 | LYRM1 | TSS1500 | N_Shore |
| cg04374393 | 3.67E-07 | SOX14 | TSS200 | Island |
| cg04375036 | 5.03E-07 | PPP1CC | TSS1500 | S_Shore |
| cg04380653 | 5.53E-07 | ASCL2 | TSS1500 | S_Shore |
| cg04390865 | 2.20E-08 | MIR320B1 | TSS1500 | S_Shelf |
| cg04416734 | 3.27E-07 | ALDOA | TSS1500 | N_Shore |
| cg04421348 | 6.33E-07 | SH2D4B | TSS1500 | S_Shelf |
| cg04427490 | 9.48E-09 | PALM | TSS1500 | N_Shore |
| cg04436755 | 1.63E-09 | LOXL1 | TSS200 | Island |
| cg04438074 | 4.13E-07 | C10orf107 | TSS200 | Island |
| cg04452432 | 3.11E-07 | CX3CL1 | 1stExon | Open Sea |
| cg04453050 | 9.27E-08 | GRM2 | TSS200 | Island |
| cg04454571 | 1.04E-08 | DKFZP434K028 | TSS200 | Open Sea |
| cg04472592 | 6.94E-07 | KRT80 | TSS200 | Open Sea |
| cg04475868 | 9.98E-07 | LOC732275 | TSS1500 | Open Sea |
| cg04477962 | 4.61E-07 | METTL7A | TSS1500 | Open Sea |
| cg04489366 | 4.78E-08 | SMPX | TSS1500 | Open Sea |
| cg04489573 | 3.61E-08 | TRIM63 | 1stExon | Open Sea |
| cg04492847 | 5.23E-07 | NUPR1 | TSS200 | Open Sea |
| cg04497992 | 4.59E-07 | NHLRC4 | TSS1500 | S_Shore |
| cg04508701 | 1.42E-08 | OLIG3 | 1stExon | N_Shore |
| cg04513227 | 9.74E-07 | GPATCH1 | TSS200 | N_Shore |
| cg04517429 | 7.59E-10 | DCHS2 | TSS1500 | S_Shore |
| cg04518959 | 2.49E-07 | TNIP2 | TSS1500 | S_Shore |
| cg04521765 | 1.56E-08 | LOXL4 | TSS1500 | S_Shore |
| cg04527918 | 7.81E-07 | UCN | TSS200 | Island |
| cg04529658 | 1.62E-07 | PGGT1B | TSS1500 | S_Shore |
| cg04533291 | 3.10E-07 | CIAO1 | TSS1500 | N_Shore |
| cg04540406 | 4.42E-07 | SHCBP1 | TSS1500 | S_Shore |
| cg04543865 | 4.15E-07 | GAN | TSS1500 | N_Shore |
| cg04543957 | 4.41E-09 | PHYH | TSS1500 | N_Shore |
| cg04551500 | 4.57E-09 | SYNC | TSS1500 | S_Shore |
| cg04551925 | 8.21E-09 | AQP1 | 1stExon | Open Sea |
| cg04552852 | 8.11E-07 | TSPAN4 | TSS200 | S_Shore |
| cg04558553 | 9.61E-09 | UGT2B7 | TSS1500 | Open Sea |
| cg04573398 | 3.12E-07 | CCDC81 | 1stExon | Island |
| cg04587910 | 9.20E-07 | NHEJ1 | TSS200 | S_Shore |
| cg04589674 | 2.23E-07 | HTRA4 | TSS200 | N_Shore |
| cg04593696 | 5.89E-07 | SLC38A8 | TSS1500 | Open Sea |
| cg04594452 | 1.03E-07 | HMX2 | TSS1500 | N_Shore |
| cg04611437 | 1.38E-07 | SEC31B | TSS200 | Island |
| cg04612566 | 7.99E-08 | C8orf31 | 1stExon | Open Sea |
| cg04629204 | 7.73E-07 | EXTL1 | 1stExon | Open Sea |
| cg04635849 | 1.56E-07 | CHN2 | TSS200 | Open Sea |
| cg04641787 | 9.30E-08 | FLJ45983 | TSS1500 | Island |
| cg04663564 | 3.84E-08 | AKR1B1 | TSS1500 | S_Shore |
| cg04664126 | 6.63E-08 | SCAND3 | 1stExon | N_Shore |
| cg04670168 | 3.11E-08 | MYOZ3 | TSS1500 | S_Shelf |
| cg04676799 | 6.43E-11 | CDO1 | TSS1500 | S_Shore |
| cg04678141 | 5.85E-07 | SVIL | TSS200 | Open Sea |
| cg04682135 | 3.38E-07 | C3orf55 | TSS1500 | Island |
| cg04682967 | 6.16E-07 | SSTR3 | TSS1500 | Open Sea |
| cg04683149 | 9.30E-07 | WISP1 | TSS200 | Open Sea |
| cg04688815 | 1.86E-07 | LOC100132163 | TSS200 | Open Sea |
| cg04689048 | 1.15E-08 | PNMAL2 | TSS200 | Island |
| cg04695027 | 7.74E-07 | CATSPER1 | 1stExon | S_Shelf |
| cg04700814 | 2.58E-07 | HEXIM1 | TSS1500 | N_Shore |
| cg04702538 | 1.25E-07 | TRPV2 | 1stExon | Open Sea |
| cg04707254 | 4.25E-08 | DENND5B | TSS1500 | S_Shore |
| cg04707715 | 4.48E-07 | LOC728264 | TSS1500 | Open Sea |
| cg04710179 | 1.10E-07 | TBC1D1 | TSS1500 | N_Shore |
| cg04718492 | 1.22E-07 | VTCN1 | TSS200 | Open Sea |
| cg04719949 | 1.00E-08 | ZNF135 | TSS1500 | N_Shore |
| cg04735123 | 5.34E-10 | MYL2 | TSS1500 | Open Sea |
| cg04738827 | 8.76E-07 | C2orf68 | TSS1500 | S_Shore |
| cg04742550 | 4.98E-08 | ITGAX | TSS200 | Open Sea |
| cg04779161 | 7.35E-08 | DMPK | TSS1500 | N_Shelf |
| cg04792712 | 9.69E-08 | MTMR7 | TSS200 | Island |
| cg04794532 | 1.76E-07 | CRYZ | TSS1500 | Open Sea |
| cg04811512 | 5.26E-07 | PAX1 | TSS1500 | N_Shore |
| cg04813911 | 1.60E-07 | CILP | TSS200 | Open Sea |
| cg04818845 | 4.96E-07 | PLEKHG5 | TSS200 | Open Sea |
| cg04833514 | 5.42E-08 | SLC6A9 | TSS1500 | S_Shore |
| cg04833731 | 7.60E-07 | GDPD3 | 1stExon | Open Sea |
| cg04835284 | 2.15E-07 | SEC31B | TSS200 | S_Shore |
| cg04840930 | 2.66E-07 | PLAGL1 | TSS1500 | S_Shore |
| cg04858155 | 5.98E-07 | ALX4 | TSS1500 | Island |
| cg04859929 | 2.54E-07 | GBA | TSS1500 | Open Sea |
| cg04861096 | 9.32E-07 | STUB1 | TSS1500 | N_Shore |
| cg04862556 | 2.77E-08 | SLFN13 | TSS200 | N_Shore |
| cg04863892 | 5.49E-07 | HOXA5 | TSS200 | Island |
| cg04876474 | 5.27E-07 | KCNQ1DN | TSS1500 | N_Shore |
| cg04880751 | 4.25E-07 | KMO | TSS1500 | Open Sea |
| cg04893119 | 3.95E-08 | PI15 | TSS1500 | Open Sea |
| cg04895345 | 8.08E-07 | STARD13 | TSS200 | Open Sea |
| cg04895975 | 2.18E-07 | TCAP | TSS200 | N_Shelf |
| cg04904385 | 3.85E-08 | TWIST1 | TSS1500 | S_Shore |
| cg04904458 | 2.11E-08 | EPPK1 | TSS1500 | S_Shore |
| cg04907595 | 4.68E-07 | RUNX3 | TSS1500 | Island |
| cg04909854 | 8.88E-08 | LOC284688 | TSS1500 | Open Sea |
| cg04912297 | 1.23E-07 | ASAH1 | TSS1500 | S_Shore |
| cg04912984 | 4.53E-08 | VANGL1 | TSS1500 | N_Shore |
| cg04922320 | 1.67E-07 | CST5 | TSS1500 | Open Sea |
| cg04922803 | 9.83E-07 | GLT8D2 | TSS1500 | Open Sea |
| cg04927537 | 1.71E-07 | LGALS3BP | TSS200 | Open Sea |
| cg04935278 | 1.45E-07 | ITPRIP | TSS200 | N_Shelf |
| cg04937851 | 7.53E-07 | COPS8 | TSS1500 | N_Shore |
| cg04943586 | 2.29E-07 | RFX4 | TSS1500 | Open Sea |
| cg04946561 | 2.29E-07 | ARRDC5 | TSS200 | Open Sea |
| cg04950931 | 2.85E-07 | TSPAN8 | TSS1500 | Open Sea |
| cg04959480 | 3.08E-07 | SFRP2 | TSS1500 | N_Shore |
| cg04965050 | 3.24E-07 | SCUBE1 | TSS1500 | Island |
| cg04968084 | 2.83E-09 | LARP4B | TSS1500 | Open Sea |
| cg04968127 | 2.44E-08 | MRVI1 | 1stExon | Open Sea |
| cg04969921 | 1.35E-08 | WDFY1 | TSS1500 | S_Shore |
| cg04973418 | 5.83E-08 | NR2C1 | TSS1500 | S_Shore |
| cg04977602 | 2.46E-07 | PRLR | 1stExon | Open Sea |
| cg04983151 | 4.17E-09 | ALS2CR11 | 1stExon | Island |
| cg04985372 | 2.56E-07 | LOC100132163 | TSS1500 | Open Sea |
| cg04985661 | 5.55E-09 | PHACTR1 | TSS1500 | Open Sea |
| cg04986504 | 3.71E-08 | DKK3 | TSS1500 | S_Shore |
| cg04986555 | 2.03E-07 | TMEM61 | 1stExon | Island |
| cg04987857 | 7.51E-09 | FRS2 | TSS1500 | N_Shore |
| cg04991214 | 1.84E-10 | PFDN2 | TSS1500 | S_Shore |
| cg05003411 | 1.65E-07 | C1QTNF7 | TSS1500 | Open Sea |
| cg05006384 | 9.74E-11 | C14orf49 | TSS200 | Open Sea |
| cg05025211 | 6.51E-07 | LCN9 | TSS1500 | Open Sea |
| cg05036937 | 7.96E-07 | ITGA2 | TSS1500 | N_Shore |
| cg05046020 | 3.14E-07 | CD1D | TSS1500 | S_Shore |
| cg05050042 | 7.34E-07 | SFRP2 | TSS1500 | N_Shore |
| cg05059480 | 7.33E-08 | C15orf52 | TSS200 | Open Sea |
| cg05061471 | 1.98E-07 | CALCA | TSS1500 | N_Shore |
| cg05061886 | 8.54E-07 | MN1 | 1stExon | Island |
| cg05068202 | 3.59E-08 | ASCL2 | TSS1500 | S_Shore |
| cg05081497 | 1.29E-08 | RASSF2 | TSS1500 | S_Shore |
| cg05092353 | 1.90E-08 | F10 | TSS200 | Open Sea |
| cg05104080 | 2.88E-07 | ILKAP | TSS1500 | S_Shore |
| cg05113927 | 3.01E-07 | UCN | TSS200 | Island |
| cg05116719 | 9.77E-11 | EIF3F | TSS1500 | N_Shore |
| cg05124692 | 1.99E-07 | PADI4 | TSS1500 | Open Sea |
| cg05129081 | 1.34E-07 | TP63 | TSS1500 | Open Sea |
| cg05133314 | 1.33E-08 | GPR153 | TSS1500 | S_Shore |
| cg05138203 | 4.24E-07 | ITGBL1 | TSS1500 | N_Shore |
| cg05146436 | 6.52E-07 | C2orf81 | TSS1500 | N_Shore |
| cg05152503 | 1.85E-07 | MYLK2 | TSS200 | N_Shelf |
| cg05158074 | 4.76E-07 | CCDC63 | TSS1500 | Open Sea |
| cg05162533 | 6.89E-07 | FOXJ1 | TSS200 | Island |
| cg05171937 | 5.81E-08 | STK38L | TSS1500 | N_Shore |
| cg05178502 | 2.31E-07 | HSPA7 | TSS200 | Island |
| cg05180540 | 6.52E-10 | PDE7A | TSS1500 | Open Sea |
| cg05183271 | 7.71E-07 | TMPRSS5 | TSS1500 | Open Sea |
| cg05185738 | 2.73E-11 | MIR629 | TSS200 | Open Sea |
| cg05188948 | 5.02E-08 | HEPHL1 | TSS200 | Open Sea |
| cg05192898 | 9.83E-08 | TXNRD1 | TSS1500 | N_Shore |
| cg05198819 | 5.84E-07 | RAD50 | TSS200 | N_Shore |
| cg05203217 | 3.39E-07 | KCNS1 | TSS1500 | S_Shore |
| cg05211836 | 1.73E-08 | F2RL3 | TSS1500 | N_Shore |
| cg05220968 | 1.98E-07 | EPM2A | TSS1500 | S_Shore |
| cg05226283 | 5.07E-07 | LOC554202 | TSS1500 | S_Shore |
| cg05228186 | 3.40E-07 | DLG2 | 1stExon | Open Sea |
| cg05228408 | 3.22E-07 | CLCN6 | TSS1500 | N_Shore |
| cg05229803 | 1.15E-08 | FUT4 | 1stExon | Island |
| cg05241277 | 6.26E-07 | SFRP2 | TSS1500 | N_Shore |
| cg05241828 | 5.43E-08 | GPT | TSS1500 | N_Shore |
| cg05242371 | 1.07E-07 | DMPK | TSS1500 | N_Shelf |
| cg05243389 | 7.33E-07 | MYEF2 | TSS1500 | S_Shore |
| cg05245650 | 5.47E-07 | CDC42EP3 | TSS1500 | S_Shore |
| cg05247640 | 1.32E-08 | VAMP2 | TSS1500 | S_Shore |
| cg05251190 | 8.65E-07 | MIR146B | TSS200 | S_Shelf |
| cg05264101 | 8.80E-08 | FLJ45079 | TSS200 | N_Shelf |
| cg05272790 | 3.24E-08 | TMPRSS13 | TSS200 | Open Sea |
| cg05278256 | 9.52E-08 | MYO18B | TSS200 | Open Sea |
| cg05281206 | 4.30E-07 | CCL13 | TSS1500 | Open Sea |
| cg05286280 | 9.90E-07 | C3orf70 | TSS1500 | S_Shore |
| cg05290737 | 2.14E-07 | GPR81 | TSS200 | Open Sea |
| cg05291374 | 6.52E-07 | DIXDC1 | TSS1500 | N_Shore |
| cg05303901 | 1.50E-08 | CREB5 | TSS1500 | Open Sea |
| cg05304507 | 5.87E-07 | FRK | TSS200 | Open Sea |
| cg05316065 | 4.78E-08 | GSDMC | 1stExon | Open Sea |
| cg05328461 | 2.52E-07 | EIF3G | TSS1500 | S_Shore |
| cg05331340 | 2.18E-07 | ASGR1 | TSS200 | Open Sea |
| cg05335315 | 8.53E-08 | JAG2 | TSS1500 | Open Sea |
| cg05337019 | 4.61E-08 | SLC25A34 | TSS1500 | S_Shelf |
| cg05343404 | 4.43E-07 | TBXA2R | TSS1500 | S_Shore |
| cg05346831 | 2.00E-07 | MIR614 | TSS1500 | Open Sea |
| cg05348123 | 6.99E-08 | SCRG1 | TSS1500 | Open Sea |
| cg05348852 | 3.04E-07 | C13orf29 | TSS200 | Open Sea |
| cg05350879 | 1.47E-08 | SPARCL1 | TSS1500 | Open Sea |
| cg05355346 | 7.08E-08 | UTP23 | TSS1500 | N_Shore |
| cg05362168 | 5.02E-08 | FAM65A | TSS1500 | N_Shore |
| cg05363903 | 2.21E-09 | IQCA1 | 1stExon | N_Shore |
| cg05365729 | 2.32E-07 | LOXL2 | TSS1500 | S_Shore |
| cg05383490 | 6.36E-07 | FAM155A | 1stExon | N_Shore |
| cg05384271 | 9.69E-07 | CRB3 | TSS1500 | Island |
| cg05390307 | 2.55E-07 | FEZ1 | TSS1500 | S_Shore |
| cg05393736 | 5.11E-09 | NRBF2 | TSS1500 | N_Shore |
| cg05397697 | 1.98E-07 | PRO1768 | TSS1500 | Open Sea |
| cg05397738 | 1.67E-07 | PGRMC1 | TSS1500 | N_Shore |
| cg05401069 | 1.45E-10 | TTC39C | TSS200 | Open Sea |
| cg05414908 | 8.18E-07 | LOC100302652 | TSS200 | Island |
| cg05419425 | 8.87E-07 | KIAA1024 | TSS1500 | N_Shore |
| cg05426966 | 1.23E-08 | CDKL2 | TSS1500 | S_Shore |
| cg05430989 | 3.22E-07 | SLCO2A1 | TSS1500 | S_Shore |
| cg05432017 | 2.65E-07 | JMJD7 | TSS1500 | N_Shore |
| cg05439368 | 7.18E-08 | TRIM69 | TSS1500 | Open Sea |
| cg05441897 | 3.82E-07 | ZNF232 | TSS1500 | Island |
| cg05445291 | 3.65E-10 | C8orf12 | TSS200 | Open Sea |
| cg05447833 | 7.79E-07 | GDF6 | TSS1500 | Island |
| cg05458220 | 9.79E-07 | ADNP2 | TSS1500 | Open Sea |
| cg05460716 | 2.29E-09 | DUSP27 | TSS1500 | Open Sea |
| cg05460965 | 2.62E-07 | CDKN1A | TSS1500 | N_Shore |
| cg05465916 | 3.09E-07 | LOC284023 | TSS1500 | Island |
| cg05465935 | 8.40E-10 | C5orf34 | TSS1500 | S_Shore |
| cg05472974 | 7.13E-07 | ANKRD53 | TSS200 | N_Shore |
| cg05476956 | 8.28E-09 | PCDHB13 | 1stExon | Island |
| cg05483406 | 1.08E-07 | ZNF34 | TSS1500 | S_Shore |
| cg05485060 | 7.00E-07 | CTNNAL1 | TSS1500 | S_Shore |
| cg05485462 | 4.24E-07 | CLDN9 | TSS200 | N_Shore |
| cg05499367 | 1.67E-08 | TMEM26 | TSS1500 | S_Shore |
| cg05507908 | 9.37E-08 | ANKRD31 | TSS1500 | S_Shore |
| cg05509753 | 2.70E-07 | TGFB1I1 | TSS1500 | N_Shore |
| cg05517572 | 3.89E-07 | STAP2 | 1stExon | Open Sea |
| cg05523897 | 2.21E-07 | C1orf86 | TSS200 | S_Shore |
| cg05528131 | 4.43E-08 | FAM8A1 | TSS1500 | N_Shore |
| cg05532178 | 5.81E-08 | HKDC1 | TSS1500 | Open Sea |
| cg05544396 | 5.43E-07 | RRAD | TSS1500 | S_Shore |
| cg05546878 | 9.14E-07 | PRKAG3 | TSS200 | Open Sea |
| cg05547778 | 7.67E-07 | PLCB2 | TSS200 | Open Sea |
| cg05548425 | 4.96E-07 | RILP | TSS200 | S_Shore |
| cg05557262 | 9.18E-11 | FAM180A | TSS1500 | Open Sea |
| cg05558937 | 1.86E-07 | MYH4 | TSS1500 | Open Sea |
| cg05559445 | 6.35E-07 | CDKN1C | TSS1500 | Island |
| cg05565052 | 9.31E-07 | TMEM184B | TSS1500 | S_Shore |
| cg05569220 | 3.56E-08 | LOC388428 | TSS1500 | N_Shore |
| cg05570980 | 1.61E-08 | C3orf52 | TSS200 | Island |
| cg05576959 | 6.82E-08 | DGKZ | TSS200 | N_Shore |
| cg05581394 | 6.26E-08 | SLC2A13 | TSS1500 | S_Shore |
| cg05581415 | 2.78E-08 | DNASE1L2 | 1stExon | N_Shore |
| cg05600864 | 6.69E-07 | C1QTNF2 | TSS1500 | S_Shore |
| cg05607053 | 1.84E-07 | SOX6 | TSS1500 | Open Sea |
| cg05616995 | 5.99E-07 | GNPDA1 | TSS1500 | S_Shore |
| cg05617886 | 3.36E-08 | OAZ3 | TSS200 | S_Shelf |
| cg05619785 | 4.17E-07 | TNXB | TSS1500 | Open Sea |
| cg05623727 | 2.06E-09 | RBM5 | TSS1500 | N_Shore |
| cg05626376 | 1.77E-07 | STK19 | TSS200 | N_Shore |
| cg05643373 | 7.44E-08 | MAMSTR | TSS1500 | N_Shelf |
| cg05643453 | 3.09E-07 | CASQ1 | TSS1500 | Open Sea |
| cg05650171 | 1.57E-09 | PFDN2 | TSS1500 | S_Shore |
| cg05650674 | 4.80E-08 | CDH3 | TSS200 | N_Shore |
| cg05654164 | 2.24E-07 | C1orf52 | TSS1500 | S_Shore |
| cg05655806 | 6.16E-10 | CD96 | TSS200 | Open Sea |
| cg05660795 | 7.74E-07 | IGFBP1 | 1stExon | Island |
| cg05667256 | 9.10E-08 | MC2R | TSS200 | Open Sea |
| cg05669832 | 7.19E-07 | PRKD3 | TSS1500 | Open Sea |
| cg05669853 | 5.56E-07 | BEND3 | 1stExon | N_Shore |
| cg05670596 | 1.54E-07 | CCRL2 | TSS1500 | Open Sea |
| cg05671070 | 1.23E-07 | FLJ45983 | TSS1500 | Island |
| cg05681757 | 8.68E-07 | FGD4 | TSS200 | Open Sea |
| cg05684622 | 8.01E-11 | RAB40AL | TSS200 | N_Shore |
| cg05685023 | 1.00E-07 | LPP | TSS1500 | N_Shore |
| cg05691004 | 4.66E-07 | SPATS2L | TSS200 | N_Shore |
| cg05694095 | 1.67E-11 | C5orf58 | TSS1500 | N_Shore |
| cg05700897 | 1.18E-08 | LOC148696 | TSS1500 | Open Sea |
| cg05713242 | 9.92E-08 | R3HCC1 | TSS1500 | N_Shore |
| cg05714559 | 6.36E-10 | SYNPO | 1stExon | Open Sea |
| cg05724197 | 5.49E-09 | CX3CL1 | TSS1500 | Open Sea |
| cg05725703 | 2.46E-08 | MYL3 | TSS1500 | Open Sea |
| cg05726935 | 4.53E-07 | AKT1 | TSS1500 | S_Shore |
| cg05730038 | 1.25E-07 | MFSD6L | TSS200 | Island |
| cg05756622 | 5.25E-07 | C20orf166 | TSS200 | Island |
| cg05759150 | 1.54E-08 | HMX2 | TSS1500 | N_Shore |
| cg05771342 | 2.48E-08 | MRGPRF | TSS1500 | S_Shelf |
| cg05776336 | 4.60E-08 | CP | 1stExon | Open Sea |
| cg05776861 | 2.58E-07 | ARHGEF7 | TSS200 | Open Sea |
| cg05777316 | 1.70E-07 | ELMO3 | TSS1500 | N_Shore |
| cg05779272 | 3.26E-09 | B4GALT1 | TSS1500 | S_Shore |
| cg05782445 | 3.49E-07 | KCNK3 | TSS1500 | N_Shore |
| cg05785988 | 1.67E-07 | LRRN2 | TSS1500 | S_Shore |
| cg05794117 | 5.07E-07 | OBSCN | TSS1500 | Open Sea |
| cg05796261 | 1.53E-07 | PTPN23 | TSS1500 | N_Shore |
| cg05796704 | 3.67E-08 | FBXO44 | TSS1500 | Island |
| cg05798627 | 8.62E-07 | CABP2 | TSS1500 | S_Shelf |
| cg05800289 | 2.84E-07 | AQP11 | 1stExon | S_Shore |
| cg05800416 | 5.87E-09 | CSGALNACT1 | TSS200 | Island |
| cg05803296 | 1.88E-07 | ATXN7L1 | TSS200 | Open Sea |
| cg05815196 | 1.20E-07 | PLA2G3 | TSS200 | Open Sea |
| cg05815275 | 4.10E-07 | ANKK1 | TSS200 | N_Shore |
| cg05823563 | 5.24E-08 | C9orf125 | TSS1500 | S_Shore |
| cg05824218 | 2.75E-08 | RARA | 1stExon | S_Shore |
| cg05835416 | 4.89E-07 | CYP2C18 | TSS1500 | Open Sea |
| cg05839709 | 6.56E-07 | ZC3H12C | TSS1500 | N_Shore |
| cg05843788 | 2.28E-07 | GPRC5C | TSS1500 | N_Shore |
| cg05845376 | 8.31E-08 | SLC25A2 | TSS200 | Island |
| cg05856884 | 5.13E-07 | SMPX | TSS200 | Open Sea |
| cg05859267 | 7.57E-07 | ADAMTS18 | TSS1500 | S_Shore |
| cg05860978 | 8.23E-08 | IL17B | TSS200 | Open Sea |
| cg05863098 | 8.08E-10 | C14orf48 | TSS1500 | Open Sea |
| cg05864261 | 5.31E-09 | ABCG8 | TSS200 | Open Sea |
| cg05876069 | 1.32E-07 | SYNPO | 1stExon | Open Sea |
| cg05881697 | 1.21E-07 | C4orf27 | TSS1500 | S_Shore |
| cg05884705 | 6.30E-07 | PLCB2 | 1stExon | Open Sea |
| cg05885484 | 4.80E-07 | CSNK1G3 | TSS1500 | N_Shore |
| cg05887421 | 4.17E-07 | ATPBD4 | TSS1500 | S_Shore |
| cg05905586 | 2.34E-11 | SSBP2 | TSS1500 | S_Shore |
| cg05908587 | 3.93E-07 | GALNTL1 | TSS1500 | Island |
| cg05915981 | 7.66E-07 | AKAP7 | 1stExon | Open Sea |
| cg05923226 | 2.24E-07 | CCDC105 | TSS200 | N_Shore |
| cg05928186 | 1.48E-09 | HOXA6 | 1stExon | Island |
| cg05934698 | 3.47E-08 | HNF1A | TSS200 | N_Shore |
| cg05936800 | 1.56E-09 | TNFSF18 | TSS1500 | Open Sea |
| cg05937873 | 2.28E-08 | FLOT1 | TSS1500 | Island |
| cg05941060 | 1.09E-07 | PCDHB9 | 1stExon | Island |
| cg05945615 | 4.05E-07 | SH3BP1 | TSS1500 | Island |
| cg05949660 | 1.57E-07 | MICAL1 | TSS1500 | Island |
| cg05950212 | 1.05E-07 | HIST2H2AB | TSS1500 | S_Shore |
| cg05952543 | 3.97E-09 | MKRN3 | TSS200 | Open Sea |
| cg05955301 | 9.19E-07 | PRELP | TSS1500 | Open Sea |
| cg05958582 | 2.56E-07 | PKD2L1 | TSS200 | Open Sea |
| cg05962092 | 6.96E-08 | KCNA7 | 1stExon | Island |
| cg05964918 | 1.20E-08 | C10orf129 | TSS200 | Open Sea |
| cg05969591 | 3.39E-07 | C2orf88 | TSS1500 | N_Shore |
| cg05970811 | 6.24E-07 | SRGAP1 | TSS1500 | N_Shore |
| cg05973792 | 2.22E-07 | DCTN4 | TSS1500 | S_Shore |
| cg05977992 | 4.37E-07 | MTHFD1L | TSS1500 | N_Shore |
| cg05979020 | 7.14E-07 | HOXD10 | TSS200 | Island |
| cg05979400 | 7.19E-07 | LOC100128731 | TSS1500 | S_Shore |
| cg05989693 | 5.81E-07 | RASSF1 | TSS1500 | N_Shore |
| cg05990366 | 6.47E-07 | FAM101A | TSS200 | Open Sea |
| cg06002197 | 5.35E-07 | CENPN | TSS1500 | N_Shore |
| cg06008378 | 1.87E-08 | PDLIM7 | TSS1500 | S_Shore |
| cg06033764 | 9.35E-07 | RNF138 | TSS1500 | N_Shore |
| cg06051146 | 4.30E-07 | CLP1 | TSS1500 | N_Shore |
| cg06051753 | 2.36E-08 | GPR179 | TSS200 | Open Sea |
| cg06055845 | 6.80E-08 | MIR641 | TSS1500 | N_Shore |
| cg06055873 | 1.14E-08 | HOXA2 | 1stExon | N_Shore |
| cg06061966 | 9.08E-07 | DGKZ | TSS1500 | N_Shore |
| cg06062102 | 8.57E-11 | STRA6 | TSS1500 | Open Sea |
| cg06065608 | 1.59E-07 | PACRG | TSS200 | N_Shore |
| cg06092815 | 5.15E-07 | SPHKAP | TSS200 | Island |
| cg06095115 | 8.05E-07 | CLEC3A | 1stExon | Open Sea |
| cg06095752 | 4.41E-07 | MAP4K4 | TSS1500 | N_Shore |
| cg06096805 | 1.77E-07 | FAM102A | TSS1500 | S_Shore |
| cg06106428 | 2.89E-08 | ARHGAP20 | TSS1500 | S_Shore |
| cg06107216 | 2.14E-07 | NCRNA00114 | TSS200 | Open Sea |
| cg06110286 | 3.41E-07 | GPT | TSS1500 | N_Shore |
| cg06110816 | 2.66E-07 | DUSP5 | TSS1500 | N_Shore |
| cg06114987 | 8.60E-08 | FOXL1 | TSS1500 | N_Shore |
| cg06126815 | 2.91E-09 | PON2 | TSS1500 | S_Shore |
| cg06136185 | 1.06E-08 | LOC388428 | TSS1500 | N_Shore |
| cg06136702 | 2.99E-07 | PSORS1C1 | 1stExon | Open Sea |
| cg06154570 | 1.78E-07 | HEYL | TSS1500 | S_Shore |
| cg06158227 | 4.87E-07 | TUBGCP4 | TSS1500 | N_Shore |
| cg06159340 | 7.56E-09 | CTF1 | TSS1500 | S_Shore |
| cg06159484 | 4.62E-09 | ERRFI1 | TSS1500 | S_Shore |
| cg06160973 | 8.71E-08 | LRP1 | 1stExon | S_Shore |
| cg06166490 | 7.71E-11 | HOXA2 | TSS1500 | Island |
| cg06166767 | 4.11E-09 | SFRP1 | TSS1500 | S_Shore |
| cg06173513 | 1.52E-09 | LOC145474 | TSS1500 | Open Sea |
| cg06177278 | 5.07E-07 | ACTN2 | TSS1500 | N_Shore |
| cg06177754 | 4.90E-07 | MIR550-2 | TSS1500 | S_Shelf |
| cg06182329 | 2.43E-07 | MYH14 | TSS1500 | Island |
| cg06182359 | 6.27E-07 | PFDN2 | TSS1500 | S_Shore |
| cg06182584 | 3.97E-07 | C6orf136 | TSS1500 | N_Shore |
| cg06188744 | 1.43E-08 | PFKP | TSS1500 | Island |
| cg06195280 | 6.58E-07 | XAF1 | TSS1500 | Open Sea |
| cg06206357 | 4.91E-07 | MYL10 | TSS1500 | Open Sea |
| cg06219995 | 2.00E-07 | CLCNKB | TSS1500 | Open Sea |
| cg06223162 | 5.29E-07 | GPR88 | TSS200 | N_Shore |
| cg06239352 | 1.51E-07 | SRCIN1 | TSS1500 | S_Shore |
| cg06270993 | 2.12E-07 | C7orf30 | TSS1500 | N_Shore |
| cg06271561 | 6.39E-07 | ALPK3 | TSS200 | N_Shore |
| cg06273075 | 1.15E-08 | MIR193B | TSS200 | S_Shore |
| cg06273125 | 3.12E-07 | SIDT1 | TSS1500 | N_Shore |
| cg06287137 | 6.71E-08 | DNAJC5G | TSS1500 | Open Sea |
| cg06288089 | 7.22E-08 | KCNQ1OT1 | TSS1500 | S_Shore |
| cg06288355 | 2.47E-09 | RAG1 | TSS200 | Open Sea |
| cg06289725 | 4.03E-08 | CHRM1 | TSS200 | N_Shore |
| cg06293365 | 6.42E-07 | TMEM120A | TSS1500 | S_Shore |
| cg06301529 | 5.50E-08 | NOS2 | 1stExon | Open Sea |
| cg06303238 | 6.25E-08 | SALL4 | 1stExon | Island |
| cg06308084 | 7.26E-07 | TTLL8 | TSS1500 | N_Shelf |
| cg06322064 | 7.12E-08 | CYSLTR2 | TSS1500 | Open Sea |
| cg06325088 | 3.32E-07 | C10orf71 | TSS1500 | Open Sea |
| cg06327732 | 6.68E-08 | CD86 | TSS1500 | Open Sea |
| cg06334307 | 8.14E-07 | MYO7A | 1stExon | Open Sea |
| cg06342490 | 8.16E-07 | MTA3 | TSS1500 | N_Shore |
| cg06351503 | 5.32E-07 | RDBP | TSS1500 | S_Shore |
| cg06359968 | 2.49E-07 | ZFPM1 | TSS1500 | Island |
| cg06363129 | 2.44E-07 | SOSTDC1 | TSS200 | Open Sea |
| cg06371044 | 4.69E-07 | NRBP2 | TSS1500 | S_Shore |
| cg06383163 | 2.03E-07 | FAM135B | TSS200 | Island |
| cg06387669 | 1.31E-08 | SERPINF2 | 1stExon | Open Sea |
| cg06390484 | 5.18E-07 | CALD1 | TSS1500 | Open Sea |
| cg06400255 | 1.27E-07 | ALDOA | TSS1500 | N_Shore |
| cg06401979 | 7.87E-08 | HOXA2 | TSS1500 | S_Shore |
| cg06405206 | 5.81E-07 | NXPH3 | TSS1500 | Island |
| cg06417962 | 1.27E-07 | RNH1 | TSS1500 | Island |
| cg06420834 | 4.43E-09 | MYL9 | TSS1500 | Island |
| cg06422039 | 6.61E-07 | SSTR5 | TSS200 | Island |
| cg06427957 | 3.86E-08 | ATP6AP1L | 1stExon | Open Sea |
| cg06429466 | 2.45E-08 | TRPV6 | 1stExon | Open Sea |
| cg06430061 | 1.77E-09 | C5orf37 | TSS1500 | S_Shore |
| cg06431953 | 7.08E-08 | LOC282997 | TSS200 | N_Shore |
| cg06440087 | 4.77E-07 | ITIH2 | TSS200 | Open Sea |
| cg06444781 | 1.84E-08 | HNF1A | TSS200 | Island |
| cg06445343 | 1.72E-08 | PPP1R9A | TSS1500 | S_Shore |
| cg06453916 | 2.34E-07 | QPRT | 1stExon | Open Sea |
| cg06459724 | 5.23E-11 | RAB7L1 | TSS1500 | S_Shore |
| cg06460691 | 1.41E-07 | FAM169A | TSS1500 | S_Shore |
| cg06462780 | 2.78E-07 | IGFALS | TSS200 | S_Shore |
| cg06466222 | 9.56E-07 | NPFFR1 | TSS1500 | Open Sea |
| cg06471905 | 4.25E-07 | ADAM11 | TSS1500 | N_Shore |
| cg06472341 | 7.05E-08 | MUC2 | TSS1500 | Open Sea |
| cg06475006 | 2.59E-09 | MC1R | 1stExon | Island |
| cg06477069 | 1.76E-07 | C20orf117 | TSS1500 | S_Shore |
| cg06484553 | 2.50E-07 | C10orf72 | TSS1500 | S_Shore |
| cg06498267 | 4.99E-07 | HCN1 | 1stExon | Island |
| cg06502279 | 1.61E-07 | RAB11FIP3 | TSS1500 | N_Shore |
| cg06521299 | 1.60E-08 | PAFAH2 | TSS1500 | S_Shore |
| cg06523224 | 8.90E-09 | BNC1 | TSS1500 | Island |
| cg06523556 | 1.53E-07 | CHRNA6 | TSS200 | Open Sea |
| cg06526620 | 5.18E-08 | FUT4 | 1stExon | Island |
| cg06530441 | 6.40E-07 | CPT1B | TSS200 | Island |
| cg06531741 | 9.62E-07 | HTR3B | TSS200 | Open Sea |
| cg06532379 | 6.89E-08 | ALPK3 | 1stExon | Island |
| cg06538684 | 1.22E-08 | LOH12CR2 | TSS1500 | S_Shore |
| cg06540876 | 3.91E-07 | ZBTB12 | TSS1500 | S_Shore |
| cg06549238 | 6.58E-07 | C11orf75 | TSS1500 | S_Shore |
| cg06565975 | 1.04E-07 | SLURP1 | TSS200 | S_Shelf |
| cg06566994 | 2.35E-07 | ZNF167 | TSS1500 | N_Shore |
| cg06594281 | 5.83E-08 | SLC16A5 | TSS1500 | N_Shore |
| cg06615154 | 1.61E-07 | S100A3 | TSS1500 | Open Sea |
| cg06618097 | 7.00E-07 | PNMAL2 | TSS1500 | S_Shore |
| cg06618474 | 3.69E-08 | CRIP1 | TSS1500 | N_Shore |
| cg06624056 | 7.05E-09 | LOC402644 | TSS200 | Open Sea |
| cg06631310 | 9.14E-07 | SLC26A10 | TSS1500 | Island |
| cg06634886 | 3.87E-07 | LOC340094 | TSS1500 | Open Sea |
| cg06638811 | 9.80E-07 | RNU6ATAC | TSS1500 | Island |
| cg06639320 | 1.38E-07 | FHL2 | TSS200 | Island |
| cg06639691 | 7.55E-07 | KCNK18 | TSS200 | Open Sea |
| cg06641366 | 1.37E-07 | LRRC8C | TSS1500 | N_Shore |
| cg06642677 | 2.49E-07 | SNRPE | TSS1500 | N_Shore |
| cg06644553 | 9.89E-07 | LVRN | TSS1500 | Island |
| cg06649280 | 6.27E-07 | MYOM3 | TSS200 | Open Sea |
| cg06650067 | 2.83E-08 | DENND4A | TSS1500 | Island |
| cg06650260 | 3.75E-09 | NR0B2 | 1stExon | Open Sea |
| cg06654628 | 5.93E-08 | SYNPO | TSS1500 | Open Sea |
| cg06658391 | 5.34E-09 | IL1RN | TSS1500 | Open Sea |
| cg06663317 | 1.77E-09 | MT2A | TSS1500 | N_Shore |
| cg06665622 | 3.80E-08 | RCAN2 | TSS200 | Open Sea |
| cg06668829 | 3.15E-07 | EPPK1 | TSS1500 | S_Shore |
| cg06675455 | 5.33E-07 | OR51T1 | TSS1500 | Open Sea |
| cg06675531 | 9.54E-09 | SYNPO | TSS1500 | Open Sea |
| cg06685111 | 2.29E-08 | HCG18 | TSS1500 | S_Shore |
| cg06686156 | 2.30E-07 | ACSM5 | TSS1500 | Open Sea |
| cg06686742 | 7.75E-07 | LASS4 | TSS1500 | N_Shore |
| cg06697595 | 1.89E-07 | TRIM38 | TSS1500 | Open Sea |
| cg06698332 | 4.01E-07 | S100A10 | TSS1500 | S_Shore |
| cg06704605 | 7.71E-08 | ZMYND10 | 1stExon | Island |
| cg06705591 | 8.57E-07 | C17orf60 | TSS200 | Open Sea |
| cg06707978 | 6.09E-07 | ZIK1 | TSS1500 | Island |
| cg06710648 | 4.06E-07 | DAB1 | TSS200 | S_Shore |
| cg06711259 | 4.42E-07 | JOSD1 | 1stExon | N_Shore |
| cg06711560 | 3.50E-07 | MAMDC4 | TSS1500 | S_Shore |
| cg06713671 | 1.65E-07 | CAPS | TSS200 | N_Shore |
| cg06720722 | 3.17E-07 | TP63 | TSS200 | Open Sea |
| cg06727703 | 1.98E-07 | TMEM105 | TSS1500 | Open Sea |
| cg06750167 | 3.76E-07 | TESC | TSS1500 | S_Shore |
| cg06751920 | 1.18E-09 | AADACL4 | TSS1500 | Open Sea |
| cg06754197 | 1.05E-07 | MIR199A1 | TSS200 | Open Sea |
| cg06757810 | 1.87E-09 | CNGB3 | TSS1500 | Open Sea |
| cg06761377 | 4.30E-07 | SLC25A34 | TSS200 | Open Sea |
| cg06761584 | 2.89E-10 | FLNC | TSS1500 | N_Shore |
| cg06762457 | 3.28E-07 | ZC3H12D | TSS1500 | Open Sea |
| cg06766636 | 1.69E-07 | TSPYL1 | 1stExon | N_Shelf |
| cg06768010 | 5.96E-08 | LOC151534 | TSS1500 | Island |
| cg06770536 | 5.46E-08 | C1orf127 | TSS1500 | Open Sea |
| cg06775759 | 9.33E-08 | KLHL34 | TSS1500 | Island |
| cg06787669 | 7.95E-07 | MYO1G | TSS200 | Open Sea |
| cg06804705 | 4.43E-07 | NCRNA00114 | TSS1500 | Open Sea |
| cg06812840 | 7.06E-08 | SOX15 | 1stExon | Island |
| cg06825512 | 5.35E-08 | APCDD1 | TSS1500 | N_Shore |
| cg06830148 | 1.14E-09 | RPL3L | TSS200 | Open Sea |
| cg06830319 | 2.84E-07 | CX3CL1 | TSS1500 | Open Sea |
| cg06833564 | 2.62E-08 | FAM100A | TSS1500 | Island |
| cg06846752 | 7.26E-08 | ZBTB8B | TSS1500 | N_Shore |
| cg06848073 | 3.15E-07 | FBXO44 | TSS1500 | N_Shore |
| cg06864079 | 1.25E-07 | REM2 | TSS1500 | N_Shelf |
| cg06865255 | 4.57E-07 | BMP5 | 1stExon | Open Sea |
| cg06868946 | 6.73E-07 | XKR4 | 1stExon | S_Shore |
| cg06870213 | 3.12E-07 | HMGCLL1 | TSS1500 | S_Shore |
| cg06874426 | 3.67E-08 | ABI3 | TSS200 | Open Sea |
| cg06877848 | 1.45E-07 | NME2 | TSS200 | N_Shore |
| cg06885583 | 1.91E-07 | MRVI1 | TSS1500 | Open Sea |
| cg06885782 | 3.70E-07 | KCNQ4 | TSS1500 | N_Shore |
| cg06888900 | 2.21E-08 | BCAR1 | TSS1500 | S_Shore |
| cg06889108 | 4.31E-08 | CPEB4 | 1stExon | S_Shore |
| cg06890415 | 4.07E-07 | C8orf46 | TSS200 | Open Sea |
| cg06892912 | 6.52E-07 | RNF39 | TSS1500 | S_Shore |
| cg06902698 | 2.24E-07 | SCNM1 | TSS1500 | Open Sea |
| cg06905367 | 4.15E-07 | SPATS2L | TSS1500 | N_Shore |
| cg06913600 | 1.57E-08 | HAAO | TSS1500 | S_Shore |
| cg06916001 | 1.11E-07 | MIR365-1 | TSS200 | Open Sea |
| cg06929414 | 1.40E-07 | POFUT2 | TSS1500 | Island |
| cg06940127 | 5.59E-07 | SERPINF1 | 1stExon | Open Sea |
| cg06941635 | 3.99E-08 | SEPT8 | TSS1500 | S_Shore |
| cg06942814 | 4.39E-08 | HOXA4 | TSS1500 | S_Shore |
| cg06948989 | 1.37E-08 | PCDHB15 | 1stExon | Island |
| cg06953865 | 2.41E-07 | ISYNA1 | TSS1500 | S_Shore |
| cg06956052 | 1.01E-07 | ATP1A4 | TSS200 | Open Sea |
| cg06960881 | 4.45E-10 | PMP22 | 1stExon | S_Shore |
| cg06961205 | 5.80E-10 | LOC285780 | TSS200 | Open Sea |
| cg06962787 | 3.83E-07 | CDRT1 | 1stExon | Open Sea |
| cg06965373 | 9.70E-08 | TMEM133 | TSS200 | Open Sea |
| cg06969845 | 5.69E-07 | HRH2 | TSS1500 | N_Shore |
| cg06970090 | 4.11E-09 | SPOCK2 | TSS200 | S_Shore |
| cg06972903 | 2.13E-07 | PLEKHG5 | TSS1500 | Open Sea |
| cg06975311 | 5.07E-07 | SIPA1 | TSS200 | N_Shore |
| cg06976994 | 4.36E-10 | OSTN | TSS1500 | Open Sea |
| cg06977987 | 1.03E-08 | OR51T1 | TSS1500 | Open Sea |
| cg06979924 | 1.91E-07 | FDPS | TSS1500 | N_Shore |
| cg06981910 | 1.48E-07 | C7orf45 | TSS1500 | S_Shore |
| cg06998765 | 3.47E-07 | RPS6KL1 | TSS1500 | N_Shore |
| cg07002058 | 1.98E-07 | BZRAP1 | TSS1500 | Open Sea |
| cg07005767 | 2.83E-07 | ABRA | TSS200 | Open Sea |
| cg07013258 | 5.63E-07 | BCL7C | TSS1500 | S_Shore |
| cg07016258 | 1.56E-08 | BNC1 | TSS1500 | Island |
| cg07026448 | 8.76E-09 | SLC22A2 | TSS1500 | S_Shore |
| cg07028661 | 4.55E-10 | GRIK1 | TSS1500 | S_Shore |
| cg07048210 | 1.98E-07 | C1orf86 | TSS200 | S_Shore |
| cg07048516 | 1.64E-08 | C22orf15 | TSS200 | Open Sea |
| cg07048519 | 8.10E-08 | CDRT1 | 1stExon | Open Sea |
| cg07049592 | 1.13E-07 | HOXA5 | TSS1500 | Island |
| cg07052627 | 5.20E-08 | MDK | TSS1500 | N_Shore |
| cg07054208 | 5.68E-07 | DCDC2 | TSS1500 | N_Shore |
| cg07054292 | 8.35E-10 | CYP27C1 | 1stExon | Open Sea |
| cg07054895 | 1.97E-08 | NPR2 | TSS200 | S_Shore |
| cg07084358 | 1.55E-08 | GLRB | TSS1500 | N_Shore |
| cg07095346 | 5.30E-07 | ITIH4 | TSS200 | Open Sea |
| cg07103722 | 1.20E-07 | MICA | TSS1500 | N_Shore |
| cg07112210 | 4.63E-07 | MGAT5B | TSS1500 | N_Shore |
| cg07116947 | 9.00E-08 | DPH1 | TSS1500 | N_Shore |
| cg07122905 | 1.04E-08 | TC2N | 1stExon | Open Sea |
| cg07124995 | 1.42E-07 | POL3S | TSS1500 | Open Sea |
| cg07125469 | 4.29E-07 | HDDC2 | TSS1500 | S_Shore |
| cg07135629 | 3.00E-08 | AQP1 | 1stExon | Open Sea |
| cg07145284 | 4.47E-07 | CD248 | TSS200 | S_Shore |
| cg07147033 | 5.08E-07 | MIB2 | TSS1500 | N_Shore |
| cg07148458 | 7.20E-07 | KCTD11 | TSS1500 | N_Shore |
| cg07150830 | 9.73E-08 | NOS2 | 1stExon | Open Sea |
| cg07153965 | 9.72E-08 | ACRV1 | TSS1500 | Open Sea |
| cg07154254 | 7.03E-08 | ELF3 | TSS1500 | Open Sea |
| cg07161827 | 3.19E-07 | DYSF | TSS200 | Island |
| cg07175301 | 4.52E-07 | IGSF9 | TSS1500 | S_Shore |
| cg07177379 | 2.96E-08 | ATP1A2 | TSS200 | Open Sea |
| cg07179457 | 2.01E-07 | ACOT2 | TSS1500 | N_Shore |
| cg07187268 | 4.02E-07 | C22orf15 | TSS200 | Open Sea |
| cg07187607 | 2.63E-07 | NDUFB4 | TSS1500 | N_Shore |
| cg07197059 | 3.25E-07 | EFS | TSS1500 | S_Shore |
| cg07197585 | 1.10E-09 | EPHX4 | TSS1500 | N_Shore |
| cg07216529 | 6.78E-08 | ACTA2 | TSS200 | Open Sea |
| cg07219955 | 1.76E-07 | PLA2G7 | TSS1500 | S_Shore |
| cg07220448 | 7.83E-07 | SOSTDC1 | TSS200 | Open Sea |
| cg07224067 | 4.38E-07 | RSAD1 | TSS1500 | N_Shore |
| cg07225966 | 3.56E-08 | CYP26C1 | TSS1500 | Island |
| cg07233761 | 1.98E-07 | ESM1 | TSS200 | Open Sea |
| cg07236884 | 1.66E-09 | MIR614 | TSS200 | Open Sea |
| cg07238926 | 1.48E-10 | PNLIPRP1 | TSS200 | Open Sea |
| cg07251857 | 8.89E-08 | ALPK3 | 1stExon | Island |
| cg07269146 | 1.47E-10 | CCL21 | TSS1500 | Open Sea |
| cg07269682 | 2.37E-07 | TSPAN4 | TSS1500 | S_Shore |
| cg07271580 | 1.13E-07 | FAM198B | TSS1500 | Open Sea |
| cg07274204 | 9.21E-08 | POL3S | 1stExon | Open Sea |
| cg07280097 | 9.15E-07 | GP9 | TSS200 | Open Sea |
| cg07281370 | 6.66E-07 | MIR125B1 | TSS1500 | Open Sea |
| cg07284261 | 3.28E-07 | CSF1R | TSS200 | Open Sea |
| cg07286253 | 7.80E-07 | SNORA13 | TSS1500 | N_Shore |
| cg07289133 | 2.85E-08 | FAM92B | TSS1500 | Open Sea |
| cg07290269 | 7.23E-07 | FAM60A | TSS1500 | S_Shore |
| cg07294814 | 2.24E-07 | GLI4 | TSS1500 | N_Shore |
| cg07301105 | 4.41E-07 | C2orf52 | TSS1500 | Island |
| cg07305719 | 3.31E-10 | ZNF365 | TSS1500 | N_Shore |
| cg07306755 | 5.33E-07 | GEM | TSS1500 | S_Shore |
| cg07313504 | 1.07E-07 | CRYGC | TSS1500 | Open Sea |
| cg07317017 | 1.44E-09 | LOXL1 | TSS200 | Island |
| cg07328351 | 6.25E-07 | S100B | TSS1500 | Open Sea |
| cg07331053 | 7.96E-07 | EPHX2 | TSS1500 | N_Shore |
| cg07349136 | 9.84E-07 | MLN | TSS200 | Open Sea |
| cg07350057 | 5.36E-09 | GGT1 | TSS200 | Open Sea |
| cg07352544 | 1.25E-07 | SEPN1 | TSS1500 | Open Sea |
| cg07368661 | 2.34E-07 | CASKIN2 | TSS1500 | Open Sea |
| cg07368817 | 5.99E-09 | TRPV2 | TSS200 | Open Sea |
| cg07376029 | 1.23E-07 | GC | TSS1500 | Open Sea |
| cg07378309 | 3.11E-07 | FBXO40 | TSS200 | Open Sea |
| cg07382070 | 1.28E-08 | POPDC2 | TSS200 | Open Sea |
| cg07397616 | 5.44E-07 | SH3BP1 | TSS1500 | N_Shore |
| cg07423149 | 1.65E-07 | CHI3L1 | TSS1500 | Open Sea |
| cg07434082 | 2.91E-08 | ARHGEF7 | TSS200 | Open Sea |
| cg07436701 | 1.13E-10 | MMRN2 | TSS200 | Open Sea |
| cg07436807 | 7.28E-07 | ACTA2 | TSS200 | Open Sea |
| cg07439128 | 1.11E-08 | KCNQ1DN | TSS1500 | N_Shore |
| cg07441143 | 3.13E-07 | SLURP1 | TSS200 | S_Shelf |
| cg07452306 | 2.02E-07 | SFXN5 | TSS1500 | S_Shore |
| cg07464571 | 4.25E-08 | LEP | TSS1500 | Island |
| cg07464859 | 7.88E-08 | C16orf45 | TSS1500 | N_Shore |
| cg07478210 | 2.73E-07 | PQLC3 | TSS1500 | N_Shore |
| cg07481491 | 8.18E-07 | TBXA2R | TSS1500 | Island |
| cg07482766 | 1.60E-09 | DBN1 | TSS1500 | Open Sea |
| cg07488259 | 3.72E-07 | SOX15 | TSS1500 | S_Shore |
| cg07488576 | 1.21E-07 | SDPR | 1stExon | Open Sea |
| cg07496545 | 1.95E-07 | SLC25A2 | TSS200 | Island |
| cg07499806 | 5.75E-07 | ZSCAN5A | TSS1500 | S_Shore |
| cg07504127 | 1.28E-08 | CLDN25 | TSS1500 | Open Sea |
| cg07507951 | 6.85E-07 | AHCTF1 | TSS1500 | S_Shore |
| cg07516252 | 2.71E-08 | REC8 | TSS200 | Island |
| cg07525299 | 9.45E-07 | ASCL2 | TSS1500 | S_Shore |
| cg07533239 | 1.05E-07 | PLEKHG6 | 1stExon | S_Shelf |
| cg07544451 | 7.27E-08 | SNAPC1 | TSS1500 | N_Shore |
| cg07545306 | 3.57E-07 | NME2 | TSS200 | N_Shore |
| cg07571745 | 7.57E-07 | LCK | TSS1500 | S_Shore |
| cg07571951 | 2.60E-07 | HSPB1 | TSS1500 | N_Shore |
| cg07573985 | 2.98E-07 | CUTA | TSS1500 | S_Shore |
| cg07575680 | 3.93E-09 | DKFZp761E198 | TSS1500 | Island |
| cg07576074 | 1.90E-07 | C21orf29 | TSS1500 | S_Shelf |
| cg07580475 | 1.83E-11 | SEMA3B | TSS1500 | Open Sea |
| cg07597892 | 7.07E-08 | RRBP1 | TSS1500 | S_Shore |
| cg07598199 | 3.67E-07 | MYOZ2 | TSS200 | Open Sea |
| cg07604732 | 1.79E-07 | LOC728276 | TSS1500 | Open Sea |
| cg07611666 | 1.71E-10 | MBP | TSS1500 | Open Sea |
| cg07612928 | 1.11E-09 | FRMD6 | 1stExon | Open Sea |
| cg07617759 | 5.73E-07 | LOC285045 | TSS200 | Open Sea |
| cg07626482 | 1.35E-08 | SLC1A5 | TSS1500 | N_Shore |
| cg07639783 | 3.08E-08 | PSMG3 | TSS1500 | S_Shore |
| cg07644368 | 6.09E-08 | CDO1 | TSS1500 | S_Shore |
| cg07645844 | 5.91E-09 | ADRA1A | 1stExon | Island |
| cg07654588 | 1.23E-09 | TBC1D5 | 1stExon | N_Shore |
| cg07655948 | 4.03E-07 | C20orf85 | TSS200 | Island |
| cg07658280 | 2.60E-07 | GPT | TSS1500 | N_Shore |
| cg07658590 | 7.60E-08 | SLC19A1 | TSS1500 | S_Shore |
| cg07664856 | 2.79E-07 | CNTN6 | TSS200 | Open Sea |
| cg07665222 | 9.90E-09 | ACRV1 | TSS1500 | Open Sea |
| cg07665535 | 1.35E-08 | MIR193B | TSS200 | S_Shore |
| cg07674459 | 5.22E-07 | FLJ42289 | TSS1500 | S_Shore |
| cg07675031 | 5.29E-07 | SPI1 | 1stExon | Island |
| cg07684019 | 6.59E-09 | PSD2 | TSS1500 | N_Shore |
| cg07685357 | 5.28E-07 | MIR125B1 | TSS1500 | Open Sea |
| cg07686859 | 1.35E-08 | SUMO1P1 | TSS1500 | Open Sea |
| cg07694754 | 8.00E-07 | TCEB3CL | TSS1500 | S_Shore |
| cg07695566 | 8.87E-07 | CYB561 | TSS1500 | S_Shore |
| cg07702888 | 1.39E-08 | RHBG | TSS1500 | Island |
| cg07703790 | 9.69E-08 | DSG1 | TSS1500 | Open Sea |
| cg07704981 | 5.53E-07 | NOS2 | 1stExon | Open Sea |
| cg07709692 | 1.06E-07 | CSTT | TSS1500 | Open Sea |
| cg07720174 | 5.77E-08 | ALS2CR4 | TSS1500 | S_Shore |
| cg07721547 | 6.07E-07 | C22orf32 | TSS1500 | N_Shore |
| cg07727884 | 6.02E-07 | MFAP4 | TSS200 | Open Sea |
| cg07741984 | 2.09E-07 | C3orf52 | TSS200 | Island |
| cg07747553 | 2.50E-08 | RASD2 | TSS1500 | Island |
| cg07748217 | 5.34E-07 | FOXL1 | 1stExon | Island |
| cg07751793 | 6.36E-08 | C1QTNF2 | TSS200 | S_Shore |
| cg07763768 | 3.09E-07 | C9orf45 | TSS200 | Open Sea |
| cg07765167 | 2.08E-09 | MRPL45 | TSS1500 | N_Shore |
| cg07766743 | 6.67E-07 | EPHX2 | TSS1500 | N_Shore |
| cg07773434 | 3.15E-07 | PPP1R14B | TSS1500 | Island |
| cg07787240 | 2.59E-09 | SLC2A5 | TSS1500 | N_Shore |
| cg07793952 | 2.84E-08 | HSD17B7 | TSS1500 | N_Shore |
| cg07795968 | 7.18E-07 | JPH2 | TSS1500 | Open Sea |
| cg07802909 | 8.78E-07 | KCNMB1 | TSS1500 | Open Sea |
| cg07805642 | 5.68E-07 | TAAR6 | TSS1500 | Open Sea |
| cg07808761 | 6.94E-07 | ZNF790 | TSS1500 | S_Shore |
| cg07809909 | 3.76E-07 | FOXS1 | TSS200 | S_Shore |
| cg07813254 | 4.49E-07 | FAM43B | TSS1500 | N_Shore |
| cg07816439 | 2.08E-07 | C1QTNF2 | TSS200 | S_Shore |
| cg07821739 | 1.42E-09 | EXTL1 | TSS1500 | Open Sea |
| cg07828169 | 5.81E-10 |  | TSS1500 | S_Shore |
| cg07831164 | 5.50E-08 | NUBP1 | TSS1500 | N_Shore |
| cg07835270 | 2.53E-07 | MT1DP | TSS1500 | N_Shore |
| cg07838048 | 5.16E-08 | MTSS1L | TSS1500 | Island |
| cg07846167 | 1.60E-07 | FBLIM1 | TSS1500 | N_Shore |
| cg07850154 | 2.20E-07 | RNF180 | TSS1500 | N_Shore |
| cg07859439 | 5.28E-08 | PGAP1 | TSS1500 | S_Shore |
| cg07870378 | 8.52E-09 | FAM100A | TSS1500 | Island |
| cg07873325 | 1.45E-07 | KRCC1 | TSS1500 | S_Shore |
| cg07890104 | 5.90E-07 | AGXT2L1 | TSS1500 | S_Shore |
| cg07892167 | 2.68E-09 | R3HCC1 | TSS1500 | N_Shore |
| cg07903677 | 4.06E-07 | KCNA3 | TSS1500 | S_Shore |
| cg07908160 | 9.57E-08 | MKS1 | TSS200 | S_Shore |
| cg07910680 | 3.99E-10 | ALPK2 | TSS1500 | Open Sea |
| cg07913153 | 1.00E-07 | LGALS8 | TSS1500 | Open Sea |
| cg07918341 | 2.73E-10 | GFAP | 1stExon | S_Shelf |
| cg07926025 | 2.81E-07 | CER1 | TSS200 | Open Sea |
| cg07942479 | 5.41E-07 | ACSL1 | TSS1500 | S_Shore |
| cg07951417 | 9.61E-07 | ELN | TSS1500 | Open Sea |
| cg07957491 | 7.70E-07 | UCK1 | TSS1500 | S_Shore |
| cg07959380 | 3.90E-07 | SPIRE2 | TSS1500 | Island |
| cg07960762 | 2.50E-07 | PALMD | 1stExon | Open Sea |
| cg07960944 | 9.38E-07 | LDLR | TSS1500 | N_Shore |
| cg07962311 | 5.94E-07 | KLHL38 | TSS1500 | Open Sea |
| cg07977490 | 8.12E-09 | C16orf45 | TSS1500 | N_Shore |
| cg07993743 | 9.49E-07 | WNT9B | TSS1500 | Island |
| cg08009487 | 2.01E-08 | KRT4 | 1stExon | Open Sea |
| cg08018825 | 9.20E-07 | SEL1L3 | TSS1500 | Island |
| cg08027034 | 5.87E-07 | MUC21 | TSS1500 | Open Sea |
| cg08030987 | 4.96E-07 | MIR575 | TSS1500 | Open Sea |
| cg08036798 | 2.34E-07 | MYO7A | TSS200 | Open Sea |
| cg08057985 | 1.95E-07 | FAM109B | TSS200 | Island |
| cg08066035 | 1.11E-10 | F2RL3 | TSS1500 | N_Shore |
| cg08067617 | 3.66E-07 | F2RL3 | TSS200 | N_Shore |
| cg08081778 | 9.96E-07 | PTPRE | TSS200 | Open Sea |
| cg08089513 | 1.45E-07 | FAM3B | TSS1500 | N_Shore |
| cg08092863 | 9.06E-08 | PFDN5 | TSS1500 | N_Shore |
| cg08095437 | 1.79E-09 | C2orf80 | TSS1500 | Open Sea |
| cg08095700 | 8.01E-07 | EHHADH | TSS1500 | S_Shore |
| cg08097631 | 4.72E-09 | C14orf48 | TSS1500 | Open Sea |
| cg08105844 | 3.30E-07 | TMEM89 | TSS200 | Open Sea |
| cg08114317 | 2.73E-07 | KIAA1751 | TSS1500 | S_Shore |
| cg08121984 | 2.96E-07 | APOC1P1 | TSS200 | Open Sea |
| cg08124030 | 4.92E-07 | TM4SF1 | 1stExon | Open Sea |
| cg08125125 | 2.65E-07 | SYNPO | TSS200 | Open Sea |
| cg08129583 | 5.00E-07 | GYPC | TSS1500 | N_Shore |
| cg08130179 | 2.73E-07 | JAK3 | TSS200 | S_Shore |
| cg08134856 | 6.95E-07 | STMN1 | TSS1500 | S_Shore |
| cg08136313 | 8.50E-09 | PRO0611 | TSS1500 | Open Sea |
| cg08136772 | 2.18E-07 | PCDH8 | 1stExon | Island |
| cg08147330 | 9.18E-08 | NAP1L5 | 1stExon | Open Sea |
| cg08151705 | 1.74E-07 | SLFN13 | TSS200 | N_Shore |
| cg08156531 | 2.73E-10 | GFAP | TSS200 | S_Shelf |
| cg08159663 | 1.42E-09 | NLRC5 | TSS1500 | N_Shore |
| cg08160331 | 1.76E-07 | KLHL35 | 1stExon | Island |
| cg08161546 | 8.16E-07 | ESR1 | TSS1500 | Open Sea |
| cg08163160 | 2.52E-07 | C12orf57 | TSS1500 | N_Shore |
| cg08176410 | 7.63E-08 | IPO5 | TSS200 | Open Sea |
| cg08180187 | 1.56E-07 | SOD3 | TSS1500 | Open Sea |
| cg08180525 | 1.38E-08 | DEGS2 | TSS1500 | S_Shore |
| cg08187089 | 1.91E-07 | HTA | TSS1500 | Open Sea |
| cg08199003 | 9.67E-07 | DHX32 | TSS1500 | Open Sea |
| cg08201311 | 4.71E-07 | OBSL1 | 1stExon | Island |
| cg08218799 | 3.85E-07 | ADCY4 | TSS1500 | S_Shore |
| cg08221781 | 5.62E-07 | RHOD | TSS1500 | N_Shore |
| cg08223837 | 1.19E-07 | CSNK1D | TSS1500 | S_Shore |
| cg08227353 | 2.36E-08 | KAZALD1 | 1stExon | Island |
| cg08231493 | 5.81E-08 | MTMR7 | TSS200 | Island |
| cg08231710 | 5.02E-07 | MMP23A | TSS1500 | Island |
| cg08239804 | 5.03E-08 | SGK1 | TSS1500 | Island |
| cg08241785 | 3.33E-07 | F2RL2 | TSS200 | Open Sea |
| cg08246428 | 6.37E-07 | CASQ1 | TSS200 | Open Sea |
| cg08247921 | 5.57E-09 | MANBA | TSS1500 | S_Shore |
| cg08256691 | 9.01E-08 | RIT1 | TSS1500 | Open Sea |
| cg08265926 | 1.02E-08 | CENPN | TSS1500 | N_Shore |
| cg08271031 | 5.24E-07 | PARVG | TSS200 | Open Sea |
| cg08271842 | 7.15E-09 | SLC28A1 | TSS1500 | Open Sea |
| cg08296385 | 3.30E-08 | TMEM67 | TSS1500 | N_Shore |
| cg08298827 | 8.92E-08 | SCGB1D2 | TSS200 | Open Sea |
| cg08299089 | 8.54E-08 | LTBP2 | TSS1500 | S_Shore |
| cg08300860 | 4.95E-08 | LDB3 | TSS200 | Open Sea |
| cg08307963 | 1.45E-08 | GJA5 | TSS200 | Open Sea |
| cg08322289 | 4.07E-07 | HMOX2 | TSS1500 | N_Shore |
| cg08342194 | 1.32E-07 | LY6G6E | TSS1500 | Open Sea |
| cg08343644 | 6.79E-08 | GPR56 | TSS1500 | Open Sea |
| cg08343834 | 2.06E-07 | PMP22 | TSS200 | S_Shelf |
| cg08350126 | 2.73E-08 | HPX | TSS1500 | Open Sea |
| cg08355301 | 2.62E-07 | CD79B | 1stExon | Open Sea |
| cg08358392 | 4.66E-07 | L3MBTL3 | TSS1500 | N_Shore |
| cg08358624 | 2.66E-09 | GFAP | 1stExon | S_Shelf |
| cg08361238 | 1.20E-08 | PTGFRN | TSS1500 | Island |
| cg08371934 | 1.85E-07 | RANBP3 | TSS1500 | S_Shore |
| cg08378442 | 2.54E-09 | TUBA4B | TSS1500 | N_Shore |
| cg08378932 | 8.95E-08 | TM4SF4 | TSS1500 | Open Sea |
| cg08389151 | 1.18E-08 | NCRNA00181 | TSS1500 | Island |
| cg08390254 | 6.26E-07 | ATP1A2 | TSS1500 | Open Sea |
| cg08390901 | 9.46E-07 | LOC100287216 | TSS1500 | S_Shore |
| cg08396863 | 2.46E-08 | TUBA4B | TSS1500 | N_Shore |
| cg08397344 | 3.21E-07 | FNDC1 | TSS1500 | Island |
| cg08400935 | 5.38E-09 | TPH1 | TSS1500 | Open Sea |
| cg08409642 | 3.03E-08 | MT2A | TSS1500 | N_Shore |
| cg08416522 | 1.98E-07 | C21orf7 | TSS1500 | Open Sea |
| cg08427977 | 9.95E-07 | C10orf72 | TSS1500 | S_Shore |
| cg08430680 | 3.59E-07 | TCF3 | TSS200 | S_Shelf |
| cg08441822 | 4.70E-07 | LIPC | TSS1500 | Open Sea |
| cg08443357 | 8.26E-07 | SDCBP2 | TSS200 | S_Shelf |
| cg08456334 | 6.79E-07 | PI4KB | 1stExon | N_Shore |
| cg08464513 | 6.33E-07 | MAPK3 | TSS1500 | S_Shore |
| cg08466034 | 2.43E-08 | TRPV2 | 1stExon | Open Sea |
| cg08469845 | 1.38E-09 | KRT15 | TSS1500 | Open Sea |
| cg08471896 | 5.89E-07 | RAB3GAP1 | TSS1500 | Open Sea |
| cg08473663 | 2.91E-08 | SEBOX | TSS1500 | N_Shelf |
| cg08473858 | 9.36E-09 | PHLDB1 | TSS1500 | N_Shore |
| cg08477779 | 8.49E-07 | PANK3 | TSS1500 | S_Shore |
| cg08478116 | 2.42E-07 | SCG5 | 1stExon | Open Sea |
| cg08478627 | 8.94E-07 | NBPF15 | TSS1500 | S_Shore |
| cg08479073 | 2.03E-10 | IL20 | TSS1500 | Open Sea |
| cg08481491 | 3.22E-10 | ALDH1L1 | TSS1500 | S_Shore |
| cg08493959 | 6.47E-07 | OBSL1 | TSS1500 | S_Shore |
| cg08494221 | 4.78E-07 | PURG | TSS1500 | Open Sea |
| cg08499840 | 1.77E-09 | CCL11 | TSS1500 | Open Sea |
| cg08506127 | 9.66E-07 | CD8A | TSS200 | S_Shore |
| cg08528616 | 5.81E-07 | ZXDC | TSS1500 | S_Shore |
| cg08528778 | 9.72E-08 | FAM129C | TSS200 | S_Shelf |
| cg08530859 | 8.99E-08 | LYNX1 | TSS200 | Open Sea |
| cg08534147 | 2.14E-09 | PLXNB1 | TSS1500 | S_Shore |
| cg08544436 | 9.77E-07 | SNX18 | 1stExon | Island |
| cg08548500 | 1.24E-07 | F10 | 1stExon | Open Sea |
| cg08550353 | 5.30E-07 | SGK1 | TSS1500 | Island |
| cg08563054 | 5.37E-07 | PLIN4 | TSS200 | Open Sea |
| cg08567517 | 3.50E-07 | PMEPA1 | TSS1500 | S_Shore |
| cg08571680 | 2.77E-07 | TBX10 | TSS1500 | Open Sea |
| cg08572214 | 1.40E-08 | COX18 | TSS1500 | S_Shore |
| cg08573687 | 6.21E-08 | TH | TSS200 | Open Sea |
| cg08586426 | 4.40E-07 | LMO2 | TSS1500 | Open Sea |
| cg08594936 | 6.02E-07 | C7orf16 | TSS200 | Open Sea |
| cg08598988 | 5.67E-08 | ADCY5 | 1stExon | Island |
| cg08605991 | 2.11E-08 | SIAH3 | TSS1500 | Open Sea |
| cg08611810 | 2.54E-07 | LOC100302652 | TSS200 | Island |
| cg08614290 | 3.83E-07 | VIPR2 | TSS1500 | Island |
| cg08615333 | 3.04E-07 | TGFB3 | 1stExon | N_Shore |
| cg08619954 | 2.14E-07 | C4orf6 | TSS200 | Open Sea |
| cg08625564 | 3.67E-07 | RILP | TSS200 | S_Shore |
| cg08629379 | 9.66E-10 | PNLIPRP1 | TSS200 | Open Sea |
| cg08636620 | 1.19E-07 | LRRC17 | TSS1500 | Open Sea |
| cg08638320 | 3.39E-07 | FOXD2 | TSS1500 | Island |
| cg08638929 | 1.56E-08 | TSPAN1 | TSS1500 | Open Sea |
| cg08640498 | 7.43E-07 | INHBC | TSS200 | Open Sea |
| cg08640923 | 1.75E-10 | TUBA4B | TSS200 | Island |
| cg08641278 | 7.37E-08 | SPOCK2 | 1stExon | S_Shore |
| cg08642707 | 5.45E-07 | NAP1L4 | TSS1500 | S_Shore |
| cg08642731 | 3.16E-07 | C10orf71 | TSS200 | Open Sea |
| cg08645907 | 4.71E-08 | ATXN7L1 | TSS200 | Open Sea |
| cg08650629 | 7.79E-09 | FAM109B | TSS200 | Island |
| cg08654655 | 5.81E-07 | CADM3 | TSS1500 | N_Shore |
| cg08656816 | 8.33E-10 | CCL5 | TSS200 | Open Sea |
| cg08657492 | 3.63E-08 | HOXA4 | TSS1500 | S_Shore |
| cg08660915 | 3.21E-08 | MLC1 | 1stExon | Open Sea |
| cg08667128 | 4.07E-07 | MIR193A | TSS200 | Island |
| cg08679238 | 2.21E-09 | CCRL2 | 1stExon | Open Sea |
| cg08697665 | 3.26E-07 | RARRES1 | TSS1500 | Island |
| cg08698854 | 3.08E-07 | CALD1 | TSS1500 | Open Sea |
| cg08709276 | 2.25E-07 | APOBEC2 | TSS1500 | Open Sea |
| cg08711674 | 9.71E-07 | AKT1S1 | TSS1500 | S_Shore |
| cg08715146 | 7.79E-07 | ATXN10 | TSS1500 | N_Shore |
| cg08726417 | 2.06E-07 | MYLK2 | TSS200 | N_Shelf |
| cg08732300 | 4.99E-07 | C10orf71 | TSS1500 | Open Sea |
| cg08738571 | 1.04E-07 | FGD4 | 1stExon | Open Sea |
| cg08742424 | 3.01E-08 | KLHDC5 | TSS1500 | N_Shore |
| cg08744726 | 6.62E-08 | ZNF767 | TSS1500 | S_Shore |
| cg08748913 | 2.34E-07 | KLHL3 | TSS200 | Open Sea |
| cg08749443 | 9.55E-07 | PLIN1 | TSS1500 | Open Sea |
| cg08750061 | 6.05E-07 | IL13RA1 | TSS1500 | N_Shore |
| cg08759026 | 7.85E-07 | MYEOV | TSS200 | Open Sea |
| cg08759041 | 5.94E-07 | MCC | TSS200 | S_Shore |
| cg08788438 | 3.62E-08 | FLRT1 | TSS1500 | Open Sea |
| cg08795640 | 7.71E-08 | SS18L2 | TSS1500 | N_Shore |
| cg08823182 | 1.43E-07 | S100A5 | TSS1500 | Open Sea |
| cg08825200 | 4.91E-09 | KCNMB3 | TSS200 | Open Sea |
| cg08828193 | 1.98E-07 | ZMYND11 | TSS1500 | N_Shore |
| cg08829841 | 5.10E-07 | FOXI2 | TSS200 | Island |
| cg08831077 | 9.11E-08 | HRH1 | TSS200 | Open Sea |
| cg08841048 | 6.90E-07 | ZNF428 | TSS1500 | S_Shore |
| cg08841829 | 4.94E-07 | ABCG1 | TSS1500 | N_Shore |
| cg08849095 | 9.54E-07 | MIR23B | TSS1500 | N_Shelf |
| cg08855449 | 3.30E-07 | ZIC1 | TSS1500 | N_Shore |
| cg08861493 | 1.60E-07 | MCAM | TSS1500 | S_Shore |
| cg08862033 | 3.65E-07 | LOC400940 | TSS1500 | Open Sea |
| cg08863777 | 1.66E-09 | FUT4 | 1stExon | Island |
| cg08869244 | 1.34E-07 | NDUFV1 | TSS1500 | N_Shore |
| cg08872742 | 1.48E-07 | CDH5 | TSS1500 | Open Sea |
| cg08882547 | 4.75E-08 | EXOC3L2 | TSS1500 | Island |
| cg08888956 | 2.33E-10 | NTS | TSS1500 | Open Sea |
| cg08895618 | 8.57E-07 | NDST1 | TSS1500 | Open Sea |
| cg08896939 | 8.28E-07 | RNF135 | TSS1500 | N_Shore |
| cg08903631 | 7.61E-07 | CD300LG | TSS200 | Open Sea |
| cg08909156 | 3.43E-07 | TRAPPC3 | TSS1500 | S_Shore |
| cg08911391 | 4.47E-08 | DDOST | TSS1500 | S_Shore |
| cg08923614 | 9.56E-07 | POLD3 | TSS1500 | N_Shore |
| cg08926287 | 2.99E-08 | SLC6A6 | TSS1500 | Island |
| cg08932654 | 1.10E-08 | ORMDL3 | TSS1500 | S_Shore |
| cg08934785 | 2.42E-07 | HOXA7 | TSS1500 | N_Shore |
| cg08936785 | 3.72E-08 | PTGFRN | TSS1500 | Island |
| cg08937612 | 3.42E-07 | VSIG8 | TSS1500 | Open Sea |
| cg08943292 | 3.42E-07 | CHRNA9 | TSS200 | Open Sea |
| cg08956226 | 1.96E-07 | CFB | 1stExon | Open Sea |
| cg08960448 | 4.99E-08 | SEPT12 | TSS1500 | Open Sea |
| cg08961196 | 9.82E-07 | MFHAS1 | 1stExon | Island |
| cg08965235 | 3.80E-07 | LTBP3 | 1stExon | Island |
| cg08968222 | 1.97E-07 | FAM13C | TSS1500 | S_Shore |
| cg08970682 | 2.54E-07 | FLJ20184 | TSS1500 | Open Sea |
| cg08972081 | 2.07E-07 | RBP1 | TSS1500 | S_Shore |
| cg08987989 | 4.05E-07 | PGGT1B | TSS1500 | S_Shore |
| cg08990507 | 8.10E-07 | ANKRD55 | TSS200 | Open Sea |
| cg08992827 | 4.15E-07 | HTA | TSS1500 | Open Sea |
| cg09001777 | 8.91E-07 | FUT3 | TSS200 | Open Sea |
| cg09001939 | 6.72E-08 | NTSR2 | TSS1500 | S_Shore |
| cg09004241 | 6.35E-08 | B3GNT7 | TSS1500 | N_Shore |
| cg09017434 | 1.48E-07 |  | 1stExon | Island |
| cg09028651 | 2.65E-07 | SLC25A34 | TSS1500 | S_Shelf |
| cg09031823 | 8.81E-07 | MIR575 | TSS1500 | Open Sea |
| cg09033997 | 5.00E-08 | CCR9 | TSS1500 | Open Sea |
| cg09037712 | 3.05E-07 | HLCS | TSS1500 | Island |
| cg09037813 | 1.49E-07 | LRRFIP1 | TSS1500 | N_Shore |
| cg09038914 | 1.09E-09 | GFAP | 1stExon | S_Shelf |
| cg09040752 | 6.26E-07 | AOC3 | TSS200 | Open Sea |
| cg09041720 | 1.66E-07 | C6orf195 | TSS200 | S_Shore |
| cg09054518 | 5.04E-08 | KEL | TSS1500 | Open Sea |
| cg09060914 | 2.98E-10 | SELP | TSS200 | Open Sea |
| cg09061993 | 4.41E-08 | TRAF5 | TSS1500 | N_Shore |
| cg09062970 | 3.53E-08 | NDUFV1 | TSS1500 | N_Shore |
| cg09065714 | 3.08E-07 | UCN | TSS1500 | S_Shore |
| cg09069900 | 1.30E-07 | F10 | TSS200 | S_Shelf |
| cg09071459 | 1.59E-07 | UBN1 | 1stExon | Open Sea |
| cg09072870 | 1.13E-07 | PARD6B | TSS1500 | N_Shore |
| cg09086087 | 7.89E-07 | SLC12A4 | 1stExon | N_Shore |
| cg09093993 | 1.80E-07 | PLEKHF2 | TSS1500 | N_Shore |
| cg09099500 | 4.35E-07 | MIR942 | TSS1500 | Open Sea |
| cg09132215 | 4.01E-08 | TAS1R1 | TSS200 | S_Shore |
| cg09135399 | 2.07E-07 | APOBEC3H | TSS200 | Open Sea |
| cg09135441 | 3.07E-09 | GRIP1 | TSS1500 | Open Sea |
| cg09138437 | 2.14E-08 | PYGM | 1stExon | Open Sea |
| cg09143221 | 7.81E-09 | CAMKK1 | TSS1500 | N_Shore |
| cg09147400 | 3.14E-08 | ABCC11 | TSS1500 | Open Sea |
| cg09148649 | 5.17E-07 | MRGPRE | TSS200 | N_Shore |
| cg09152089 | 4.76E-07 | IL22RA1 | 1stExon | Open Sea |
| cg09154591 | 5.56E-07 | VIT | TSS200 | Open Sea |
| cg09154850 | 8.36E-09 | CDRT4 | TSS1500 | Open Sea |
| cg09155044 | 3.90E-08 | VKORC1 | TSS1500 | S_Shore |
| cg09157302 | 3.14E-07 | SLC34A2 | TSS1500 | N_Shore |
| cg09163021 | 2.38E-08 | RAPSN | TSS200 | Open Sea |
| cg09168997 | 1.11E-08 | ATP1A2 | TSS1500 | Open Sea |
| cg09175009 | 7.54E-08 | FAM18A | TSS200 | Island |
| cg09175834 | 8.99E-07 | NAV3 | 1stExon | Open Sea |
| cg09176539 | 8.09E-09 | LOC440040 | TSS200 | Open Sea |
| cg09181792 | 1.14E-07 | CFTR | TSS1500 | Open Sea |
| cg09183671 | 7.76E-07 | LOC282997 | TSS200 | N_Shore |
| cg09190051 | 3.73E-09 | ZNF280D | TSS1500 | Island |
| cg09196146 | 6.46E-08 | PRO1768 | TSS200 | Open Sea |
| cg09205190 | 7.67E-08 | LOC728392 | TSS1500 | S_Shore |
| cg09206890 | 9.28E-09 | CUGBP1 | TSS1500 | Open Sea |
| cg09218250 | 3.13E-08 | IL17RE | TSS1500 | Open Sea |
| cg09228051 | 4.22E-07 | ABCC11 | 1stExon | Open Sea |
| cg09229893 | 6.47E-07 | BMP4 | TSS1500 | S_Shore |
| cg09239591 | 6.35E-07 | XDH | TSS1500 | Open Sea |
| cg09246302 | 2.26E-07 | C7orf53 | 1stExon | Open Sea |
| cg09247319 | 1.75E-07 | DNMT3L | TSS1500 | Open Sea |
| cg09247392 | 1.27E-08 | ASB2 | TSS1500 | Open Sea |
| cg09251429 | 6.04E-07 | ROBO3 | TSS200 | Island |
| cg09257796 | 2.15E-07 | GABRG3 | TSS1500 | Island |
| cg09265563 | 4.87E-07 | FZD9 | 1stExon | Island |
| cg09269866 | 9.08E-07 | FOXD2 | TSS1500 | Island |
| cg09276451 | 2.21E-07 | VASN | TSS1500 | N_Shore |
| cg09286367 | 2.84E-09 | MIR589 | TSS200 | S_Shore |
| cg09286468 | 2.13E-08 | LY6G6E | TSS1500 | Open Sea |
| cg09293816 | 7.84E-08 | FCGRT | TSS200 | N_Shore |
| cg09307264 | 7.76E-07 | INCA1 | TSS1500 | S_Shore |
| cg09315134 | 6.07E-07 | CTSZ | TSS1500 | S_Shore |
| cg09315367 | 8.19E-07 | SLC44A2 | TSS200 | Island |
| cg09317772 | 2.11E-07 | NME2 | TSS200 | N_Shore |
| cg09317928 | 7.90E-08 | BEST1 | TSS1500 | Open Sea |
| cg09318840 | 8.14E-08 | CD8A | TSS200 | S_Shore |
| cg09323728 | 1.62E-07 | TP53INP1 | TSS1500 | Island |
| cg09338412 | 2.79E-09 | GGT1 | TSS200 | Open Sea |
| cg09346993 | 9.62E-07 | STK19 | TSS1500 | N_Shore |
| cg09348985 | 6.92E-08 | CXCL12 | TSS1500 | Island |
| cg09357934 | 9.59E-07 | DAPL1 | 1stExon | Open Sea |
| cg09359349 | 2.90E-09 | ZNF589 | TSS1500 | N_Shore |
| cg09364688 | 9.65E-07 | TFAP2E | TSS200 | N_Shore |
| cg09372750 | 9.89E-07 | SLC22A17 | TSS1500 | S_Shore |
| cg09408571 | 5.20E-08 | GPR88 | TSS200 | N_Shore |
| cg09411212 | 4.55E-08 | POPDC2 | 1stExon | Open Sea |
| cg09414535 | 3.30E-09 | GRIP1 | TSS200 | Open Sea |
| cg09427311 | 2.31E-07 | ANGPTL2 | TSS1500 | Open Sea |
| cg09432376 | 4.14E-07 | APOL6 | TSS200 | Open Sea |
| cg09435415 | 6.72E-08 | DPYS | TSS200 | S_Shore |
| cg09450024 | 1.26E-07 | EXOC3L2 | TSS200 | Island |
| cg09452728 | 3.92E-07 | LOC100128239 | TSS200 | Open Sea |
| cg09462269 | 1.76E-07 | C1orf210 | TSS200 | Open Sea |
| cg09468328 | 5.10E-07 | SPSB1 | TSS1500 | Open Sea |
| cg09470638 | 1.53E-07 | CCDC105 | TSS200 | N_Shore |
| cg09471204 | 3.60E-07 | LOC339674 | TSS200 | Open Sea |
| cg09476950 | 3.57E-07 | GPR179 | TSS200 | Open Sea |
| cg09478591 | 2.69E-07 | C6orf153 | TSS1500 | N_Shore |
| cg09481121 | 1.77E-07 | CMYA5 | TSS1500 | Open Sea |
| cg09482093 | 8.81E-09 | RNF112 | TSS200 | Open Sea |
| cg09486642 | 6.11E-09 | TRPV6 | TSS1500 | Open Sea |
| cg09490371 | 8.20E-07 | ECEL1P2 | TSS1500 | Island |
| cg09498440 | 1.04E-08 | TMEM171 | TSS1500 | N_Shore |
| cg09499482 | 1.53E-07 | FBXL22 | TSS200 | N_Shelf |
| cg09502207 | 1.75E-08 | TMEM71 | TSS1500 | Open Sea |
| cg09504131 | 8.76E-08 | STK24 | TSS1500 | Open Sea |
| cg09513758 | 1.08E-08 | AHCYL1 | TSS1500 | N_Shore |
| cg09518518 | 1.14E-08 | AADACL4 | TSS1500 | Open Sea |
| cg09521647 | 1.38E-09 | AFF3 | TSS1500 | S_Shore |
| cg09521743 | 5.10E-08 | SYNPO2L | 1stExon | Open Sea |
| cg09533390 | 6.18E-07 | FAM19A1 | TSS200 | N_Shelf |
| cg09539538 | 6.59E-07 | FERMT1 | TSS200 | S_Shore |
| cg09539720 | 2.67E-08 | ASB10 | TSS1500 | Open Sea |
| cg09547777 | 2.83E-08 | CASQ1 | TSS200 | Open Sea |
| cg09548638 | 8.89E-08 | SH3TC2 | TSS1500 | Open Sea |
| cg09552892 | 5.38E-07 | SNCG | TSS200 | Open Sea |
| cg09554856 | 4.35E-07 | LMO2 | TSS1500 | Open Sea |
| cg09555736 | 3.09E-07 | RAB11FIP3 | TSS1500 | N_Shore |
| cg09560590 | 2.62E-07 | HMHB1 | TSS200 | Open Sea |
| cg09561663 | 4.07E-07 | TGM3 | TSS200 | Open Sea |
| cg09563617 | 5.56E-07 | SLC2A12 | TSS1500 | Open Sea |
| cg09569569 | 2.45E-08 | C2orf81 | TSS1500 | S_Shore |
| cg09573585 | 2.83E-07 | C10orf116 | TSS200 | Island |
| cg09582200 | 2.55E-07 | FXYD6 | TSS1500 | S_Shore |
| cg09582482 | 4.50E-07 | TNNI1 | 1stExon | Open Sea |
| cg09584591 | 1.69E-07 | TGFBI | TSS1500 | N_Shore |
| cg09584711 | 9.51E-11 | HPR | TSS200 | Open Sea |
| cg09593860 | 1.14E-07 | ZBTB16 | TSS1500 | N_Shore |
| cg09596958 | 1.20E-07 | AGAP2 | TSS200 | N_Shore |
| cg09598276 | 3.19E-08 | GGT1 | TSS200 | Open Sea |
| cg09607047 | 4.44E-07 | MIR365-1 | TSS1500 | Open Sea |
| cg09608949 | 5.62E-07 | FXYD6 | TSS1500 | S_Shore |
| cg09619146 | 1.17E-07 | CPXM2 | 1stExon | Island |
| cg09623056 | 1.49E-07 | NEURL3 | TSS1500 | S_Shore |
| cg09632936 | 7.01E-08 | CHRNA9 | TSS1500 | Open Sea |
| cg09634469 | 2.59E-07 | SERPIND1 | 1stExon | Open Sea |
| cg09637757 | 6.17E-08 | RIMS2 | 1stExon | Open Sea |
| cg09638264 | 8.02E-11 | WDR69 | TSS1500 | N_Shore |
| cg09639715 | 1.13E-07 | GFAP | TSS1500 | Open Sea |
| cg09639931 | 1.80E-07 | ZPBP2 | TSS200 | Island |
| cg09642020 | 2.42E-07 | C9orf163 | 1stExon | Island |
| cg09645336 | 6.81E-07 | C5orf43 | TSS1500 | S_Shore |
| cg09645475 | 4.05E-09 | FAM110A | TSS1500 | S_Shore |
| cg09652746 | 4.48E-07 | E2F4 | TSS1500 | Open Sea |
| cg09654300 | 4.84E-10 | TRAK1 | 1stExon | Open Sea |
| cg09655403 | 2.29E-07 | CMYA5 | TSS200 | Open Sea |
| cg09660867 | 5.84E-07 | LOC728264 | TSS1500 | Open Sea |
| cg09663111 | 1.31E-08 | C20orf85 | TSS200 | Island |
| cg09672233 | 4.18E-07 | LCP2 | 1stExon | Open Sea |
| cg09681043 | 3.13E-09 | MYO7B | TSS200 | Open Sea |
| cg09684429 | 3.01E-07 | ROBO4 | TSS200 | Open Sea |
| cg09684557 | 1.10E-08 | OR6C68 | TSS1500 | Open Sea |
| cg09684846 | 6.22E-10 | FAM18A | TSS200 | Island |
| cg09696411 | 1.62E-07 | SLC26A10 | TSS200 | Island |
| cg09712066 | 3.95E-08 | PART1 | TSS200 | Open Sea |
| cg09717809 | 3.04E-08 | BOLA3 | TSS1500 | S_Shore |
| cg09720117 | 5.13E-07 | KCNA5 | TSS1500 | N_Shore |
| cg09722609 | 1.34E-07 | NDST1 | TSS1500 | Open Sea |
| cg09731113 | 4.83E-11 | MYOCD | TSS1500 | N_Shore |
| cg09731694 | 6.01E-08 | C9orf50 | 1stExon | Island |
| cg09737019 | 3.16E-08 | ZNF354A | TSS1500 | S_Shore |
| cg09737314 | 2.67E-07 | ALOX12 | TSS200 | Island |
| cg09744397 | 2.75E-07 | TARSL2 | TSS1500 | S_Shore |
| cg09757588 | 1.52E-10 | TPO | TSS200 | Open Sea |
| cg09763086 | 4.40E-10 | MYL2 | TSS200 | Open Sea |
| cg09778919 | 4.66E-07 | C12orf40 | TSS200 | Open Sea |
| cg09782034 | 7.63E-07 | RORA | TSS1500 | S_Shore |
| cg09785991 | 7.75E-08 | CLCA1 | TSS200 | Open Sea |
| cg09788492 | 2.26E-07 | NKX2-3 | TSS1500 | Island |
| cg09790829 | 5.16E-07 | BACH2 | TSS1500 | S_Shore |
| cg09795195 | 2.29E-07 | DHX40 | TSS1500 | N_Shore |
| cg09804496 | 6.41E-07 | FZD9 | 1stExon | S_Shore |
| cg09810818 | 9.73E-08 | AMMECR1 | TSS200 | Open Sea |
| cg09820729 | 9.07E-07 | MFN2 | TSS1500 | N_Shore |
| cg09824721 | 8.19E-09 | RPL22L1 | TSS1500 | S_Shore |
| cg09826857 | 5.61E-07 | MGC16142 | TSS1500 | Open Sea |
| cg09835085 | 1.72E-07 | KCNE4 | TSS1500 | N_Shore |
| cg09842125 | 1.25E-07 | SOX30 | TSS1500 | S_Shore |
| cg09851245 | 1.79E-09 | LOC645676 | TSS1500 | N_Shore |
| cg09856299 | 1.57E-09 | PARP10 | 1stExon | N_Shelf |
| cg09858022 | 4.21E-07 | RARA | TSS200 | Open Sea |
| cg09858176 | 3.24E-07 | HSPB7 | TSS200 | Open Sea |
| cg09858862 | 6.62E-08 | ID3 | TSS1500 | S_Shore |
| cg09862440 | 7.33E-07 | RABL5 | TSS1500 | S_Shore |
| cg09877173 | 3.49E-07 | PDE2A | TSS200 | Open Sea |
| cg09879794 | 2.51E-07 | CCDC136 | 1stExon | S_Shore |
| cg09880291 | 2.57E-07 | HOXA5 | TSS1500 | Island |
| cg09889350 | 6.26E-08 | CETP | TSS200 | Open Sea |
| cg09889477 | 2.68E-07 | FLJ45079 | TSS1500 | N_Shelf |
| cg09899220 | 2.68E-08 | ELMO3 | TSS1500 | N_Shore |
| cg09906145 | 3.03E-07 | FAM65A | TSS1500 | N_Shore |
| cg09907758 | 3.43E-07 | C11orf95 | TSS1500 | S_Shore |
| cg09908750 | 4.39E-10 | HOXA2 | TSS1500 | S_Shore |
| cg09912793 | 3.57E-07 | PHC1 | TSS1500 | N_Shore |
| cg09922481 | 3.03E-07 | DPT | TSS200 | Open Sea |
| cg09926488 | 3.97E-07 | TPM4 | 1stExon | Island |
| cg09957864 | 4.46E-08 | GPT | TSS1500 | N_Shore |
| cg09958483 | 2.12E-07 | PYGM | 1stExon | Open Sea |
| cg09969043 | 1.98E-07 | NR2F2 | TSS200 | S_Shelf |
| cg09970023 | 1.16E-07 | SIX4 | TSS1500 | S_Shore |
| cg09974780 | 3.93E-07 | KIF2A | TSS1500 | N_Shore |
| cg09982918 | 6.22E-07 | MYO18B | TSS1500 | Open Sea |
| cg09990481 | 3.79E-07 | ACTA2 | TSS200 | Open Sea |
| cg10007222 | 9.29E-08 | GSTTP2 | TSS1500 | Open Sea |
| cg10013501 | 4.62E-08 | RUNX3 | TSS1500 | Island |
| cg10027723 | 1.23E-09 | EPS15 | TSS1500 | Open Sea |
| cg10039928 | 1.06E-07 | MATN2 | TSS1500 | N_Shore |
| cg10045354 | 4.52E-07 | C11orf93 | TSS1500 | Island |
| cg10046892 | 2.70E-07 | PAQR6 | TSS200 | S_Shelf |
| cg10046893 | 4.95E-07 | SNORD114-30 | TSS1500 | Open Sea |
| cg10047173 | 1.54E-09 | ART4 | TSS200 | Open Sea |
| cg10059687 | 3.86E-08 | TNFRSF25 | 1stExon | Island |
| cg10061906 | 6.36E-07 | ZNF34 | TSS1500 | S_Shore |
| cg10066188 | 2.03E-07 | NAT8B | 1stExon | Open Sea |
| cg10081469 | 3.43E-07 | TMEM30B | 1stExon | S_Shore |
| cg10091775 | 5.95E-07 | CA12 | TSS1500 | S_Shore |
| cg10094238 | 1.38E-07 | ARHGAP27 | 1stExon | Island |
| cg10098221 | 9.88E-08 | USP46 | TSS1500 | N_Shore |
| cg10099638 | 4.60E-07 | COL6A2 | TSS1500 | N_Shore |
| cg10102384 | 4.24E-07 | PACRG | TSS200 | N_Shore |
| cg10105149 | 2.97E-07 | SYCE1L | TSS1500 | Open Sea |
| cg10106388 | 3.49E-09 | CD244 | 1stExon | Open Sea |
| cg10107382 | 2.04E-08 | ZCCHC3 | TSS1500 | N_Shore |
| cg10115348 | 4.42E-11 | ARHGEF10L | TSS1500 | N_Shore |
| cg10122452 | 3.43E-07 | SMARCB1 | TSS1500 | N_Shore |
| cg10122877 | 6.06E-07 | C4orf19 | TSS1500 | Open Sea |
| cg10123201 | 1.80E-07 | MYL3 | TSS1500 | Open Sea |
| cg10123662 | 1.70E-09 | ARHGEF10L | TSS1500 | N_Shore |
| cg10126205 | 1.11E-07 | TWIST1 | TSS1500 | S_Shore |
| cg10126372 | 3.14E-07 | PXDNL | TSS1500 | Open Sea |
| cg10130811 | 8.23E-07 | BBS1 | TSS1500 | Open Sea |
| cg10132917 | 7.42E-08 | AQP1 | 1stExon | Open Sea |
| cg10133500 | 8.52E-07 | TMEM115 | 1stExon | N_Shore |
| cg10135474 | 5.72E-07 | LOC221122 | TSS1500 | Open Sea |
| cg10137231 | 1.30E-07 | REC8 | TSS1500 | N_Shore |
| cg10140309 | 1.68E-08 | ADNP | TSS1500 | S_Shore |
| cg10147507 | 6.88E-07 | ARHGEF3 | TSS1500 | Island |
| cg10149582 | 1.07E-07 | CMTM5 | 1stExon | Open Sea |
| cg10149889 | 3.80E-08 | LPP | TSS200 | Open Sea |
| cg10149996 | 4.32E-07 | PGRMC1 | TSS1500 | N_Shore |
| cg10150902 | 9.02E-07 | TC2N | TSS1500 | S_Shore |
| cg10153349 | 8.78E-07 | SPON1 | TSS200 | N_Shore |
| cg10159032 | 2.07E-07 | LRRC37B | TSS200 | Open Sea |
| cg10159529 | 2.80E-11 | IL5RA | TSS1500 | Open Sea |
| cg10163377 | 4.91E-07 | LOC100270710 | TSS1500 | S_Shelf |
| cg10164367 | 4.61E-08 | ITGBL1 | TSS200 | N_Shore |
| cg10168635 | 2.91E-07 | C2orf88 | TSS1500 | Island |
| cg10177528 | 4.17E-09 | TRAF5 | TSS1500 | N_Shore |
| cg10189661 | 2.58E-07 | LY6G6E | TSS1500 | Open Sea |
| cg10195901 | 9.15E-08 | HORMAD2 | TSS200 | Island |
| cg10199250 | 1.66E-07 | LVRN | TSS1500 | Island |
| cg10210624 | 5.19E-07 | ZNF329 | TSS1500 | S_Shore |
| cg10219957 | 2.70E-07 | TMEM52 | TSS1500 | S_Shore |
| cg10222534 | 1.03E-08 | KCNJ14 | 1stExon | S_Shelf |
| cg10235817 | 5.39E-07 | ADRA2C | 1stExon | Island |
| cg10242602 | 5.70E-07 | ZFP42 | TSS200 | Island |
| cg10243348 | 2.32E-07 | ITGBL1 | 1stExon | N_Shore |
| cg10248279 | 8.51E-07 | B4GALNT3 | TSS1500 | N_Shore |
| cg10249224 | 8.68E-07 | TSSK3 | 1stExon | S_Shore |
| cg10257870 | 1.10E-07 | CMYA5 | TSS200 | Open Sea |
| cg10257905 | 2.74E-10 | MYOCD | TSS1500 | N_Shore |
| cg10262710 | 2.82E-08 | LOC399959 | TSS1500 | Open Sea |
| cg10266490 | 5.08E-07 | ACOT11 | TSS200 | Open Sea |
| cg10275315 | 1.37E-09 | BNC1 | TSS1500 | S_Shore |
| cg10276301 | 7.58E-09 | LOC100133545 | TSS1500 | N_Shore |
| cg10277175 | 5.58E-07 | ANKRD39 | TSS1500 | S_Shore |
| cg10290679 | 2.29E-07 | ETS1 | 1stExon | Open Sea |
| cg10307548 | 1.75E-07 | SOD3 | TSS1500 | Open Sea |
| cg10315334 | 5.34E-10 | CCL5 | 1stExon | Open Sea |
| cg10319053 | 1.14E-09 | HOXA2 | TSS1500 | Island |
| cg10323433 | 5.36E-07 | HTR2A | TSS1500 | Open Sea |
| cg10324158 | 8.55E-07 | CLEC3B | TSS200 | Open Sea |
| cg10325224 | 4.00E-07 | IGFALS | TSS200 | S_Shore |
| cg10325659 | 2.32E-11 | ASB2 | TSS200 | Open Sea |
| cg10328844 | 4.92E-11 | LOC91948 | TSS200 | Open Sea |
| cg10351914 | 6.57E-08 | MFAP4 | TSS200 | Open Sea |
| cg10354495 | 4.19E-07 | NFU1 | TSS1500 | S_Shore |
| cg10361659 | 5.39E-09 | RNASEH2B | TSS1500 | N_Shore |
| cg10365880 | 1.42E-07 | PKD2L1 | 1stExon | Open Sea |
| cg10368049 | 3.66E-07 | COL1A2 | TSS200 | Open Sea |
| cg10371560 | 2.59E-07 | ITGAM | TSS1500 | Open Sea |
| cg10372188 | 1.39E-07 | FXYD6 | TSS1500 | S_Shore |
| cg10372921 | 1.16E-09 | LOXL1 | TSS200 | Island |
| cg10376731 | 5.21E-07 | BTRC | TSS1500 | N_Shore |
| cg10377153 | 2.43E-07 | PTPDC1 | TSS200 | Open Sea |
| cg10380289 | 1.48E-08 | HIST2H2BF | 1stExon | Island |
| cg10381440 | 2.60E-07 | HOXB2 | TSS1500 | S_Shore |
| cg10386300 | 1.24E-07 | PPL | TSS1500 | S_Shore |
| cg10399210 | 3.98E-09 | C7orf53 | TSS200 | Open Sea |
| cg10400707 | 5.71E-07 | VANGL2 | TSS1500 | N_Shore |
| cg10404009 | 4.85E-07 | MAMSTR | TSS1500 | Island |
| cg10407113 | 5.74E-08 | AP1G1 | TSS1500 | S_Shore |
| cg10412602 | 5.91E-07 | HSD3B7 | TSS1500 | S_Shelf |
| cg10426370 | 1.28E-07 | ESM1 | TSS1500 | Open Sea |
| cg10433128 | 2.26E-07 | MTSS1L | TSS1500 | Island |
| cg10433327 | 6.00E-08 | NEDD4 | TSS200 | Open Sea |
| cg10439737 | 2.64E-07 | KRTAP12-3 | TSS200 | Open Sea |
| cg10440011 | 4.69E-07 | DPH1 | TSS1500 | Island |
| cg10444806 | 2.32E-07 | ORMDL3 | TSS1500 | S_Shore |
| cg10449466 | 3.88E-07 | MGST1 | TSS1500 | Open Sea |
| cg10457446 | 2.20E-07 | SPNS2 | TSS1500 | N_Shore |
| cg10458446 | 1.28E-07 | RAB17 | TSS1500 | Open Sea |
| cg10467098 | 5.36E-07 | C11orf68 | TSS1500 | S_Shore |
| cg10479629 | 5.62E-08 | GPR179 | TSS200 | Open Sea |
| cg10482632 | 1.52E-08 | CSRP1 | TSS1500 | S_Shore |
| cg10486610 | 4.67E-09 | LOC100127888 | TSS1500 | Open Sea |
| cg10500653 | 5.33E-07 | RHBDL2 | TSS200 | Open Sea |
| cg10502150 | 5.18E-09 | ODF4 | TSS1500 | Open Sea |
| cg10502244 | 7.66E-07 | COL2A1 | TSS1500 | Island |
| cg10509692 | 2.32E-07 | LRRC30 | TSS200 | Open Sea |
| cg10513118 | 8.28E-07 | SCN2B | 1stExon | Open Sea |
| cg10513862 | 4.42E-07 | ARHGEF10L | TSS1500 | Open Sea |
| cg10523671 | 4.62E-09 | SLC15A2 | TSS1500 | Open Sea |
| cg10529796 | 5.74E-07 | MYO15A | TSS200 | Open Sea |
| cg10535845 | 1.24E-07 | TCAP | TSS200 | N_Shelf |
| cg10538146 | 8.82E-09 | KLHDC5 | TSS1500 | N_Shore |
| cg10539898 | 1.44E-07 | TNFRSF25 | TSS200 | S_Shore |
| cg10543503 | 2.44E-08 | ARHGEF10L | TSS1500 | Open Sea |
| cg10549753 | 6.88E-08 | MIR492 | TSS1500 | Open Sea |
| cg10553596 | 3.62E-07 | NEU4 | TSS200 | N_Shelf |
| cg10556330 | 9.86E-09 | ITGA6 | TSS1500 | N_Shore |
| cg10559803 | 5.56E-09 | RALGPS2 | TSS1500 | N_Shore |
| cg10561989 | 7.60E-07 | C14orf19 | TSS1500 | Open Sea |
| cg10574499 | 1.04E-07 | NRN1L | TSS200 | Island |
| cg10581837 | 3.61E-07 | LMO7 | TSS200 | Open Sea |
| cg10588362 | 3.45E-07 | C11orf85 | TSS1500 | Open Sea |
| cg10588962 | 1.62E-07 | LOC404266 | TSS1500 | N_Shore |
| cg10593400 | 9.81E-07 | DDAH1 | TSS200 | S_Shore |
| cg10596956 | 5.76E-08 | OR52M1 | TSS200 | Open Sea |
| cg10604646 | 4.76E-08 | RGS5 | 1stExon | Open Sea |
| cg10621012 | 1.57E-07 | ARAP3 | TSS1500 | Open Sea |
| cg10622586 | 3.68E-08 | STAB2 | 1stExon | Open Sea |
| cg10624122 | 1.59E-07 | TWIST1 | TSS1500 | S_Shore |
| cg10625666 | 7.93E-07 | SLC39A10 | TSS1500 | N_Shore |
| cg10632000 | 8.73E-07 | PRR19 | TSS200 | Island |
| cg10632525 | 3.93E-08 | MN1 | 1stExon | Island |
| cg10635061 | 2.37E-07 | FHL2 | 1stExon | Open Sea |
| cg10635330 | 1.05E-07 | C8orf46 | TSS200 | Open Sea |
| cg10640119 | 7.59E-07 | C9orf50 | TSS200 | S_Shore |
| cg10658197 | 1.58E-10 | ENTHD1 | TSS1500 | Open Sea |
| cg10661615 | 5.66E-07 | PRLHR | TSS200 | Island |
| cg10663078 | 2.32E-07 | SFRP2 | TSS1500 | N_Shore |
| cg10677144 | 4.25E-07 | MYOM1 | TSS200 | Open Sea |
| cg10690919 | 7.20E-07 | WFIKKN2 | TSS200 | Open Sea |
| cg10702041 | 9.90E-08 | FASTK | TSS1500 | Island |
| cg10709021 | 4.28E-07 | PURG | 1stExon | Open Sea |
| cg10720210 | 1.18E-09 | C17orf66 | TSS1500 | Open Sea |
| cg10725344 | 4.79E-08 | FAM180A | TSS200 | Open Sea |
| cg10735607 | 1.64E-08 | TMEM109 | TSS1500 | N_Shore |
| cg10736208 | 4.68E-07 | TNXB | TSS1500 | Open Sea |
| cg10736330 | 4.86E-07 | GPR183 | TSS1500 | Open Sea |
| cg10752676 | 3.22E-07 | IQUB | TSS1500 | S_Shore |
| cg10763483 | 9.62E-07 | C13orf33 | TSS1500 | N_Shore |
| cg10772169 | 9.80E-07 | AGL | TSS1500 | N_Shore |
| cg10784341 | 1.12E-08 | PHACTR2 | TSS200 | Open Sea |
| cg10786043 | 7.83E-09 | TACC1 | TSS1500 | N_Shore |
| cg10792307 | 2.67E-07 | TREML1 | TSS1500 | Open Sea |
| cg10802444 | 3.61E-09 | GRIP1 | TSS200 | Open Sea |
| cg10806586 | 5.85E-07 | C14orf28 | TSS1500 | N_Shore |
| cg10806639 | 6.32E-07 | STARD13 | 1stExon | Open Sea |
| cg10822172 | 1.65E-07 | CREB5 | TSS200 | Open Sea |
| cg10822545 | 2.19E-07 | MIR181D | TSS200 | S_Shore |
| cg10835823 | 8.17E-10 | C5orf58 | TSS200 | N_Shore |
| cg10836392 | 8.06E-07 | SULT4A1 | 1stExon | Island |
| cg10842095 | 3.18E-08 | TIMELESS | TSS1500 | S_Shore |
| cg10849289 | 9.80E-07 | KRTAP12-2 | TSS1500 | Open Sea |
| cg10853231 | 3.99E-08 | GLTSCR1 | TSS200 | N_Shore |
| cg10857774 | 8.15E-08 | DMPK | TSS1500 | N_Shelf |
| cg10864680 | 8.65E-09 | TNFSF13B | 1stExon | Open Sea |
| cg10865238 | 4.13E-11 | GRIP1 | TSS200 | Open Sea |
| cg10866623 | 1.72E-09 | TTLL6 | TSS1500 | S_Shore |
| cg10881110 | 2.12E-07 | SLC25A34 | TSS200 | Open Sea |
| cg10893683 | 5.69E-07 | KLHDC5 | TSS1500 | N_Shore |
| cg10906284 | 7.15E-07 | AVPR1A | 1stExon | Island |
| cg10916651 | 2.02E-07 | C1QA | TSS200 | Open Sea |
| cg10919177 | 4.34E-07 | ZNF445 | TSS1500 | S_Shore |
| cg10923408 | 3.38E-09 | C13orf26 | TSS200 | Open Sea |
| cg10929210 | 9.42E-07 | DNAH6 | TSS1500 | N_Shore |
| cg10932086 | 2.12E-09 | PRO1768 | TSS1500 | Open Sea |
| cg10933774 | 7.41E-10 | STARD10 | TSS1500 | S_Shore |
| cg10941749 | 6.47E-07 | LYSMD2 | TSS200 | S_Shore |
| cg10943359 | 8.85E-07 |  | TSS200 | Island |
| cg10949007 | 3.34E-08 | GLRX | TSS1500 | Open Sea |
| cg10952220 | 4.38E-08 | CCRL2 | TSS1500 | Open Sea |
| cg10954182 | 9.06E-07 | NFYB | TSS1500 | Island |
| cg10959651 | 9.72E-07 | RSAD2 | 1stExon | Open Sea |
| cg10960375 | 8.87E-07 | ZBTB47 | TSS1500 | N_Shore |
| cg10961323 | 7.24E-08 | GALE | TSS1500 | S_Shore |
| cg10963949 | 5.53E-07 | PRDX6 | TSS1500 | N_Shore |
| cg10967350 | 8.04E-07 | CNTN4 | TSS1500 | Island |
| cg10969006 | 1.03E-08 | IBSP | TSS200 | Open Sea |
| cg10970124 | 9.19E-07 | BAT4 | TSS1500 | S_Shore |
| cg10976861 | 3.94E-07 | HES6 | TSS1500 | S_Shore |
| cg10984625 | 9.61E-07 | LRRFIP1 | TSS1500 | Island |
| cg10990081 | 1.60E-07 | TRNT1 | TSS1500 | N_Shore |
| cg10990993 | 1.45E-08 | EPM2AIP1 | 1stExon | N_Shore |
| cg10999598 | 4.20E-07 | PHAX | TSS1500 | N_Shore |
| cg11015251 | 2.08E-07 | HOXA4 | TSS200 | Island |
| cg11018313 | 1.21E-08 | ALPK3 | 1stExon | N_Shore |
| cg11028075 | 2.00E-08 | SORBS1 | 1stExon | Open Sea |
| cg11035202 | 1.11E-07 | MSX2P1 | TSS1500 | N_Shore |
| cg11042501 | 1.83E-11 | OR51S1 | TSS200 | Open Sea |
| cg11043251 | 1.05E-08 | RUNX1T1 | TSS1500 | Open Sea |
| cg11077026 | 7.67E-08 | IGSF9 | TSS1500 | S_Shore |
| cg11077681 | 8.41E-08 | MRVI1 | 1stExon | Open Sea |
| cg11081272 | 2.32E-08 | GAB2 | TSS1500 | S_Shore |
| cg11095122 | 4.68E-07 | CSGALNACT1 | TSS1500 | Open Sea |
| cg11101926 | 8.63E-07 | ACCN1 | 1stExon | N_Shore |
| cg11105610 | 5.80E-07 | LGALS3BP | TSS1500 | Open Sea |
| cg11108991 | 3.70E-07 | KCNMB3 | TSS200 | Open Sea |
| cg11115622 | 4.47E-10 | PLEKHG3 | TSS1500 | N_Shore |
| cg11118778 | 2.21E-07 | PAWR | TSS1500 | S_Shore |
| cg11135108 | 9.33E-07 | PBXIP1 | TSS1500 | Open Sea |
| cg11135168 | 1.56E-11 | IGFL2 | TSS1500 | Open Sea |
| cg11139878 | 7.78E-07 | CD5L | TSS200 | Open Sea |
| cg11146367 | 1.72E-07 | PAGE4 | TSS200 | Open Sea |
| cg11147193 | 1.13E-07 | HPCAL1 | TSS1500 | N_Shore |
| cg11152302 | 6.03E-07 | NYNRIN | TSS1500 | N_Shore |
| cg11157076 | 7.80E-09 | AADACL4 | TSS1500 | Open Sea |
| cg11157816 | 1.17E-07 | OLFML3 | 1stExon | Open Sea |
| cg11171811 | 1.76E-07 | LTBP3 | TSS1500 | S_Shore |
| cg11196848 | 8.11E-07 | FGF20 | TSS200 | S_Shore |
| cg11200042 | 4.51E-07 | C10orf84 | TSS1500 | S_Shore |
| cg11202345 | 6.76E-08 | LGALS3BP | 1stExon | Open Sea |
| cg11205312 | 5.44E-07 | SCNM1 | TSS1500 | Open Sea |
| cg11210703 | 5.55E-08 | GBA3 | TSS1500 | Open Sea |
| cg11210878 | 5.66E-07 | CHRM1 | TSS200 | N_Shore |
| cg11211563 | 5.46E-07 | GEFT | TSS1500 | Island |
| cg11212178 | 1.53E-07 | MIR492 | TSS1500 | Open Sea |
| cg11222672 | 3.22E-08 | LOC100192426 | TSS200 | N_Shore |
| cg11223373 | 5.41E-07 | APC2 | TSS1500 | N_Shore |
| cg11229611 | 3.90E-07 | SCNN1D | TSS1500 | Open Sea |
| cg11241750 | 5.22E-07 | GRAMD3 | TSS1500 | Open Sea |
| cg11254726 | 2.87E-10 | PIK3C2B | TSS1500 | N_Shelf |
| cg11263011 | 5.05E-07 | MIR195 | TSS1500 | N_Shelf |
| cg11267546 | 6.84E-07 | HOXA3 | TSS1500 | N_Shore |
| cg11277966 | 4.85E-07 | KIF3C | TSS1500 | S_Shore |
| cg11293029 | 4.85E-07 | SLC12A6 | TSS1500 | S_Shore |
| cg11306628 | 7.17E-07 | PDGFA | TSS1500 | Island |
| cg11314292 | 4.54E-07 | RNF125 | TSS200 | N_Shore |
| cg11316386 | 7.67E-08 | C1orf229 | TSS200 | S_Shore |
| cg11322879 | 1.80E-07 | GATSL1 | TSS1500 | Open Sea |
| cg11327391 | 2.79E-07 | EIF1AX | TSS1500 | S_Shore |
| cg11327857 | 1.05E-07 | FGF2 | TSS1500 | N_Shore |
| cg11339849 | 7.73E-07 | SLMAP | TSS200 | S_Shore |
| cg11340260 | 2.31E-08 | GP1BA | TSS200 | Open Sea |
| cg11341086 | 2.58E-08 | DKFZp566F0947 | TSS200 | Open Sea |
| cg11342437 | 7.70E-08 | LRCH1 | TSS1500 | N_Shore |
| cg11348442 | 2.57E-08 | TUBA4B | TSS200 | N_Shore |
| cg11366363 | 8.58E-09 | GUCY1B2 | TSS1500 | Open Sea |
| cg11377136 | 2.50E-07 | PKDREJ | 1stExon | Island |
| cg11384661 | 8.79E-07 | LOC100130331 | TSS200 | Open Sea |
| cg11393773 | 4.55E-07 | C6orf136 | TSS1500 | N_Shore |
| cg11397957 | 4.02E-10 | LEAP2 | TSS200 | Open Sea |
| cg11405475 | 4.44E-08 | SNRPE | TSS1500 | N_Shore |
| cg11407540 | 7.74E-07 | EGF | TSS1500 | Open Sea |
| cg11407989 | 9.34E-09 | RPL13AP17 | TSS1500 | Open Sea |
| cg11410682 | 2.06E-07 | PNMAL2 | TSS200 | Island |
| cg11410718 | 8.91E-07 | HOXA4 | TSS200 | Island |
| cg11412713 | 3.48E-08 | FUT4 | 1stExon | Island |
| cg11426157 | 4.75E-07 | SYNPO | TSS1500 | Open Sea |
| cg11428546 | 3.16E-09 | AFF3 | TSS1500 | S_Shelf |
| cg11436222 | 1.83E-08 | HIST1H3E | 1stExon | Island |
| cg11438310 | 7.83E-07 | CMYA5 | TSS1500 | Open Sea |
| cg11485451 | 4.43E-09 | TCF7 | TSS200 | Island |
| cg11523799 | 1.05E-07 | C6orf154 | TSS1500 | S_Shore |
| cg11524454 | 6.01E-07 | PLEKHG7 | TSS1500 | Open Sea |
| cg11540997 | 8.21E-07 | DUOX2 | TSS1500 | N_Shore |
| cg11547724 | 4.73E-08 | HPX | TSS200 | Open Sea |
| cg11562153 | 6.69E-08 | GPX5 | TSS1500 | Open Sea |
| cg11569431 | 1.35E-07 | SFRS16 | TSS1500 | Island |
| cg11577355 | 4.41E-11 | AFF3 | TSS1500 | S_Shore |
| cg11585358 | 2.52E-08 | EPHX4 | TSS1500 | N_Shore |
| cg11587635 | 2.25E-08 | MAG | TSS1500 | N_Shelf |
| cg11593949 | 6.56E-09 | IGFBP1 | TSS1500 | N_Shore |
| cg11594299 | 7.38E-07 | RADIL | TSS1500 | S_Shore |
| cg11596897 | 5.21E-08 | CALCA | TSS1500 | N_Shore |
| cg11601000 | 7.35E-07 | LOC339674 | TSS200 | Open Sea |
| cg11601297 | 3.91E-08 | ITPKC | 1stExon | S_Shore |
| cg11625005 | 3.87E-08 | TERT | TSS1500 | Island |
| cg11632438 | 7.07E-08 | MFSD1 | TSS1500 | N_Shore |
| cg11633204 | 3.43E-07 | GSR | TSS1500 | S_Shore |
| cg11638071 | 2.30E-08 | BZRAP1 | 1stExon | S_Shelf |
| cg11643442 | 1.23E-07 | SNORA38 | TSS1500 | S_Shore |
| cg11644479 | 7.28E-07 | ASCL2 | TSS1500 | Island |
| cg11649713 | 1.62E-07 | AMMECR1 | TSS200 | Open Sea |
| cg11653233 | 1.06E-07 | CCR10 | TSS1500 | N_Shore |
| cg11653466 | 4.60E-07 | C8orf86 | TSS200 | Open Sea |
| cg11661281 | 6.97E-07 | OR6Q1 | 1stExon | Open Sea |
| cg11666921 | 1.72E-07 | KCNQ1OT1 | TSS1500 | Island |
| cg11671688 | 4.67E-07 | GPR6 | 1stExon | Island |
| cg11672277 | 2.24E-07 | MAPRE2 | TSS1500 | N_Shore |
| cg11675225 | 2.85E-07 | LSP1 | TSS1500 | N_Shelf |
| cg11679069 | 2.05E-10 | DNAJC15 | TSS1500 | N_Shore |
| cg11685223 | 6.54E-07 | GZF1 | TSS1500 | N_Shore |
| cg11685249 | 8.65E-07 | TSSK1B | TSS1500 | Open Sea |
| cg11688949 | 3.91E-08 | STMN4 | TSS200 | Open Sea |
| cg11689732 | 1.09E-07 | LOC388428 | TSS1500 | N_Shore |
| cg11709544 | 9.16E-09 | STMN1 | TSS1500 | Island |
| cg11711057 | 2.32E-08 | BTBD11 | TSS200 | N_Shore |
| cg11720054 | 4.01E-07 | PANX3 | TSS1500 | Open Sea |
| cg11721450 | 1.11E-07 | OPTC | TSS1500 | Open Sea |
| cg11725101 | 2.06E-09 | NBPF16 | 1stExon | Open Sea |
| cg11731337 | 1.56E-07 | CHST5 | TSS200 | Open Sea |
| cg11732492 | 1.78E-10 | NXPH3 | TSS1500 | N_Shore |
| cg11746813 | 1.18E-09 | HOXC4 | 1stExon | Island |
| cg11755251 | 2.88E-07 | MIR557 | TSS1500 | Open Sea |
| cg11764966 | 2.73E-08 | WFDC10A | TSS200 | Open Sea |
| cg11776930 | 3.24E-07 | LDB3 | TSS1500 | Open Sea |
| cg11783497 | 3.50E-09 | IL1RN | TSS200 | Open Sea |
| cg11809958 | 4.18E-07 | FGD4 | TSS200 | Open Sea |
| cg11812202 | 2.00E-12 | PNLIP | TSS200 | Open Sea |
| cg11817892 | 7.29E-07 | ANKRD23 | TSS1500 | Open Sea |
| cg11823624 | 5.35E-07 | FASTK | TSS1500 | Island |
| cg11823736 | 2.67E-08 | IDO2 | TSS200 | Open Sea |
| cg11824191 | 6.09E-07 | NMNAT2 | TSS200 | Open Sea |
| cg11825652 | 5.71E-07 | CAV2 | TSS200 | N_Shore |
| cg11832339 | 1.42E-08 | BBOX1 | TSS1500 | Open Sea |
| cg11847636 | 9.57E-07 | FCGRT | TSS200 | N_Shore |
| cg11848487 | 3.03E-07 | C6orf138 | 1stExon | N_Shore |
| cg11850943 | 1.08E-07 | LANCL2 | TSS1500 | N_Shore |
| cg11858733 | 4.59E-07 | DHH | TSS1500 | S_Shore |
| cg11861562 | 9.42E-07 | TAGLN | TSS1500 | Open Sea |
| cg11865601 | 3.77E-10 | GPLD1 | TSS1500 | N_Shelf |
| cg11869184 | 2.43E-08 | C7orf61 | TSS200 | Open Sea |
| cg11874323 | 2.70E-08 | C3orf52 | TSS1500 | N_Shore |
| cg11874627 | 8.88E-07 | PLA2G7 | TSS1500 | S_Shore |
| cg11884546 | 8.75E-08 | ITGAX | TSS200 | Open Sea |
| cg11887214 | 1.41E-07 | PYGM | 1stExon | Open Sea |
| cg11911305 | 2.30E-08 | APOC1P1 | TSS1500 | Open Sea |
| cg11914795 | 5.79E-07 | C10orf107 | TSS200 | Island |
| cg11920122 | 1.46E-07 | GNAL | TSS200 | Island |
| cg11920449 | 7.70E-07 | CDKN1A | TSS1500 | N_Shore |
| cg11923631 | 2.89E-07 | AIRE | TSS200 | Island |
| cg11928198 | 3.28E-07 | SCNM1 | TSS1500 | Open Sea |
| cg11935344 | 8.48E-08 | FAM13AOS | TSS1500 | Open Sea |
| cg11941420 | 1.45E-07 | CRYBA4 | TSS200 | Open Sea |
| cg11943820 | 3.76E-08 | ITPRIP | TSS1500 | N_Shelf |
| cg11950778 | 1.00E-07 | UGT8 | TSS1500 | N_Shore |
| cg11957475 | 1.86E-07 | PMAIP1 | TSS1500 | N_Shore |
| cg11969213 | 6.12E-07 | LOC100133991 | TSS1500 | N_Shore |
| cg11976164 | 3.42E-10 | MIR2054 | TSS1500 | Open Sea |
| cg11987682 | 2.05E-07 | NET1 | TSS1500 | N_Shore |
| cg11999288 | 8.27E-07 | LY6G5B | TSS1500 | S_Shelf |
| cg12003026 | 3.28E-09 | HIST1H2BI | 1stExon | Island |
| cg12009516 | 1.95E-08 | EXOC3L2 | TSS1500 | Island |
| cg12009856 | 2.69E-10 | ACBD7 | TSS1500 | S_Shore |
| cg12013321 | 3.16E-07 | GCLC | TSS1500 | S_Shore |
| cg12020230 | 8.06E-07 | CXCL13 | TSS200 | Open Sea |
| cg12020444 | 8.98E-07 | PIWIL1 | TSS1500 | N_Shore |
| cg12023692 | 6.40E-07 | IER3 | TSS1500 | S_Shore |
| cg12030667 | 1.23E-08 | PLXNB1 | TSS1500 | S_Shore |
| cg12033214 | 3.31E-07 | CRYBA4 | TSS200 | Open Sea |
| cg12039197 | 2.48E-07 | ACTG2 | 1stExon | Open Sea |
| cg12042952 | 7.48E-09 | AFF3 | TSS1500 | S_Shelf |
| cg12052661 | 5.48E-08 | CACNA1B | 1stExon | Island |
| cg12072024 | 1.01E-08 | CYBRD1 | TSS1500 | N_Shore |
| cg12077168 | 7.18E-09 | ATP1A4 | TSS200 | Open Sea |
| cg12079271 | 1.24E-07 | SREBF1 | TSS200 | Island |
| cg12080306 | 7.66E-07 | YPEL2 | TSS1500 | N_Shore |
| cg12091331 | 4.71E-07 | PLAT | TSS200 | Open Sea |
| cg12093180 | 8.87E-07 | ADNP | TSS1500 | S_Shore |
| cg12100791 | 4.68E-07 | PYCARD | TSS200 | S_Shore |
| cg12102016 | 5.37E-08 | EML5 | TSS1500 | S_Shore |
| cg12110437 | 3.81E-07 | LY6E | TSS1500 | N_Shore |
| cg12113819 | 8.88E-08 | MED13L | TSS1500 | Open Sea |
| cg12120359 | 9.81E-08 | HSPA2 | TSS1500 | N_Shore |
| cg12125117 | 4.78E-07 | GPR97 | TSS1500 | Open Sea |
| cg12127991 | 7.53E-07 | RHOU | TSS1500 | N_Shore |
| cg12128164 | 2.40E-07 | LRFN3 | TSS1500 | N_Shore |
| cg12135835 | 1.63E-07 | ZNF431 | TSS1500 | Open Sea |
| cg12137156 | 1.79E-07 | C8orf22 | 1stExon | Open Sea |
| cg12153755 | 7.79E-07 | ARAP3 | TSS1500 | Open Sea |
| cg12160664 | 1.16E-08 | OLFML3 | TSS200 | Open Sea |
| cg12163845 | 2.62E-07 | HAPLN4 | TSS200 | S_Shore |
| cg12176783 | 8.27E-09 | TCEA2 | TSS200 | Island |
| cg12177001 | 4.92E-08 | IFI27 | TSS1500 | Open Sea |
| cg12177334 | 1.07E-07 | DOK3 | 1stExon | Open Sea |
| cg12179658 | 6.18E-08 | HRH1 | TSS200 | Open Sea |
| cg12182284 | 1.43E-08 | CLTB | TSS1500 | S_Shore |
| cg12186702 | 7.33E-07 | PLVAP | 1stExon | Island |
| cg12188416 | 8.04E-09 | TP63 | TSS1500 | Open Sea |
| cg12196406 | 2.54E-07 | FXYD1 | TSS200 | N_Shelf |
| cg12214451 | 4.42E-07 | ARSJ | 1stExon | N_Shore |
| cg12225220 | 4.53E-07 | MAPK10 | TSS200 | Open Sea |
| cg12234455 | 6.16E-08 | MAMSTR | TSS200 | N_Shelf |
| cg12243453 | 1.17E-07 | HTR3B | TSS200 | Open Sea |
| cg12243582 | 1.51E-07 | ZNF138 | TSS1500 | N_Shore |
| cg12243822 | 1.35E-08 | DNASE1L3 | TSS1500 | Open Sea |
| cg12253175 | 1.16E-07 | AGAP2 | TSS200 | N_Shore |
| cg12254648 | 2.69E-08 | MYL9 | TSS1500 | N_Shore |
| cg12258811 | 8.69E-07 | PER3 | TSS200 | Island |
| cg12261786 | 9.51E-07 | C10orf116 | TSS1500 | N_Shore |
| cg12262372 | 8.91E-09 | NFIA | TSS1500 | N_Shore |
| cg12262378 | 8.82E-09 | ALOX12 | 1stExon | Island |
| cg12267786 | 9.10E-11 | TKTL2 | TSS200 | S_Shore |
| cg12288473 | 7.59E-08 | SALL4 | TSS1500 | S_Shore |
| cg12294678 | 5.66E-07 | NOS3 | TSS1500 | Open Sea |
| cg12302428 | 2.02E-07 | ZNF649 | TSS1500 | Open Sea |
| cg12302621 | 3.79E-08 | SLC28A1 | TSS1500 | Open Sea |
| cg12303623 | 3.39E-09 | USP54 | 1stExon | Open Sea |
| cg12303685 | 1.09E-08 | PAQR6 | TSS1500 | S_Shelf |
| cg12307484 | 4.07E-07 | TWIST1 | TSS1500 | S_Shore |
| cg12329853 | 2.00E-09 | MIR604 | TSS1500 | Open Sea |
| cg12354861 | 5.27E-07 | C1orf200 | TSS200 | S_Shore |
| cg12368752 | 5.13E-08 | TMPRSS13 | TSS200 | Open Sea |
| cg12374682 | 7.53E-07 | GPHA2 | TSS200 | Open Sea |
| cg12405136 | 1.61E-07 | MRGPRF | TSS1500 | S_Shelf |
| cg12409149 | 5.88E-07 | LOC100270710 | TSS1500 | S_Shelf |
| cg12410310 | 3.84E-07 | LGALS12 | 1stExon | Open Sea |
| cg12417689 | 5.99E-07 | C17orf97 | TSS1500 | N_Shore |
| cg12424624 | 8.53E-07 | ALS2CL | TSS200 | Open Sea |
| cg12437024 | 1.42E-07 | C20orf166 | TSS200 | Island |
| cg12437809 | 1.75E-07 | FMO2 | TSS1500 | Open Sea |
| cg12442803 | 2.93E-07 | CDON | TSS1500 | S_Shore |
| cg12451530 | 3.45E-07 | LOC100302652 | TSS200 | Island |
| cg12455187 | 2.08E-09 | CCL5 | TSS1500 | Open Sea |
| cg12456825 | 5.26E-07 | FBXO40 | TSS200 | Open Sea |
| cg12461677 | 1.47E-09 | NCRNA00181 | TSS1500 | Island |
| cg12469809 | 8.63E-07 | LOC284276 | TSS1500 | Open Sea |
| cg12476188 | 2.57E-07 | SMAD6 | TSS1500 | S_Shelf |
| cg12488187 | 7.34E-07 | MSRB3 | TSS1500 | N_Shore |
| cg12492027 | 5.43E-07 | DLGAP4 | TSS1500 | N_Shore |
| cg12499235 | 9.52E-07 | ASCL2 | TSS1500 | S_Shore |
| cg12500707 | 1.62E-09 | METTL11B | TSS200 | Open Sea |
| cg12506165 | 5.75E-07 | MAP1LC3B2 | TSS200 | Open Sea |
| cg12512013 | 1.81E-07 | DLC1 | TSS1500 | Open Sea |
| cg12526471 | 1.25E-07 | ZBTB16 | TSS1500 | N_Shore |
| cg12526849 | 5.80E-09 | EPS15 | TSS1500 | Open Sea |
| cg12529228 | 4.12E-08 | NHLH1 | 1stExon | N_Shelf |
| cg12532477 | 3.71E-08 | CHST6 | TSS1500 | S_Shore |
| cg12581592 | 1.96E-07 | SPON1 | 1stExon | N_Shore |
| cg12590494 | 4.32E-09 | BNIPL | TSS1500 | Open Sea |
| cg12591454 | 4.79E-07 | TRDMT1 | TSS1500 | S_Shore |
| cg12594244 | 1.98E-09 | LOXL1 | TSS200 | Island |
| cg12598089 | 3.18E-09 | KCNAB1 | TSS200 | N_Shore |
| cg12600371 | 7.60E-07 | CTAG2 | TSS1500 | Open Sea |
| cg12622182 | 5.24E-08 | POU3F1 | TSS1500 | Island |
| cg12623536 | 4.69E-09 | SPOCK2 | TSS200 | S_Shore |
| cg12632264 | 4.96E-08 | ZNF518A | TSS200 | N_Shore |
| cg12633680 | 8.95E-07 | FASTK | TSS1500 | Island |
| cg12634957 | 8.23E-08 | SYNC | TSS1500 | S_Shore |
| cg12636369 | 4.37E-07 | NES | 1stExon | Island |
| cg12639429 | 3.34E-10 | TTC39C | TSS200 | Open Sea |
| cg12646227 | 5.77E-09 | NSUN4 | TSS1500 | N_Shore |
| cg12647497 | 2.26E-07 | GSTM2 | TSS1500 | N_Shore |
| cg12660343 | 2.77E-09 | C10orf76 | TSS1500 | S_Shore |
| cg12663553 | 8.68E-07 | GCET2 | TSS200 | Open Sea |
| cg12664806 | 3.98E-08 | FOXO3 | 1stExon | Island |
| cg12670073 | 1.23E-09 | PMEPA1 | TSS1500 | S_Shelf |
| cg12678834 | 1.22E-08 | CXCR5 | TSS1500 | Open Sea |
| cg12682573 | 7.15E-07 | MIR451 | TSS1500 | Open Sea |
| cg12698626 | 9.57E-07 | CCL2 | TSS1500 | Open Sea |
| cg12708802 | 5.47E-07 | TUSC4 | TSS1500 | S_Shore |
| cg12710552 | 1.08E-09 | SAA2 | TSS1500 | Open Sea |
| cg12711961 | 9.52E-07 | LRRC14B | TSS200 | N_Shore |
| cg12712768 | 6.32E-07 | HNF1A | TSS1500 | N_Shore |
| cg12718502 | 8.28E-07 | TULP3 | TSS1500 | N_Shore |
| cg12729166 | 1.19E-07 | PALMD | TSS1500 | Open Sea |
| cg12729518 | 8.72E-07 | MRGPRD | 1stExon | Open Sea |
| cg12731430 | 6.50E-07 | ADC | TSS1500 | N_Shore |
| cg12732155 | 7.20E-07 | LAPTM5 | 1stExon | Open Sea |
| cg12741184 | 1.25E-07 | MYO1C | TSS1500 | S_Shore |
| cg12752420 | 1.36E-07 | C1orf210 | TSS200 | Open Sea |
| cg12754854 | 7.11E-07 | PHYHD1 | TSS1500 | Open Sea |
| cg12758687 | 9.06E-07 | DRD2 | TSS1500 | Island |
| cg12762474 | 1.49E-07 | OR51V1 | TSS1500 | Open Sea |
| cg12771178 | 8.42E-11 | FAM170B | TSS200 | S_Shore |
| cg12776033 | 9.93E-07 | STK19 | TSS1500 | N_Shore |
| cg12779575 | 3.87E-07 | ERN1 | TSS1500 | S_Shore |
| cg12782180 | 1.55E-07 | LEP | TSS1500 | Island |
| cg12793123 | 2.41E-07 | ADORA2A | 1stExon | S_Shelf |
| cg12800266 | 3.50E-07 | TBC1D22B | TSS1500 | N_Shore |
| cg12802310 | 4.00E-07 | CYP1B1 | TSS1500 | S_Shore |
| cg12805865 | 1.62E-08 | BBOX1 | TSS1500 | Open Sea |
| cg12810626 | 1.17E-07 | LY9 | TSS1500 | Open Sea |
| cg12812838 | 8.18E-07 | MIR103-1 | TSS1500 | Open Sea |
| cg12831293 | 5.71E-07 | TAS2R5 | TSS200 | Open Sea |
| cg12845808 | 5.74E-07 | PCDH12 | 1stExon | Open Sea |
| cg12846837 | 3.70E-07 | KIAA0146 | TSS1500 | Island |
| cg12853539 | 3.57E-07 | HDDC2 | TSS200 | S_Shore |
| cg12854483 | 9.82E-07 | CNN1 | 1stExon | Open Sea |
| cg12855547 | 6.21E-07 | MCC | TSS200 | S_Shore |
| cg12857875 | 5.14E-07 | KLHL30 | TSS200 | N_Shore |
| cg12865405 | 3.03E-08 | C11orf40 | TSS1500 | Open Sea |
| cg12866859 | 2.20E-07 | HEXIM1 | TSS1500 | N_Shore |
| cg12867987 | 5.22E-07 | BBS7 | TSS1500 | S_Shore |
| cg12870518 | 1.70E-08 | FLJ41941 | TSS200 | Open Sea |
| cg12871835 | 7.49E-07 | SGK1 | TSS1500 | S_Shore |
| cg12876594 | 7.72E-07 | NPR2 | TSS1500 | Island |
| cg12883767 | 4.49E-07 | SLC26A10 | TSS1500 | N_Shore |
| cg12893736 | 2.94E-07 | GPRC5C | TSS200 | S_Shore |
| cg12894524 | 1.29E-07 | MAP1LC3B2 | TSS200 | Open Sea |
| cg12894984 | 8.80E-09 | PNMT | TSS1500 | N_Shore |
| cg12910830 | 9.42E-11 | MAT2B | TSS1500 | N_Shelf |
| cg12913122 | 1.57E-07 | JAG2 | TSS1500 | Open Sea |
| cg12935478 | 1.22E-09 | C21orf84 | TSS200 | Open Sea |
| cg12935937 | 2.43E-08 | RASSF2 | TSS1500 | S_Shore |
| cg12937434 | 9.20E-07 | BZRAP1 | 1stExon | S_Shelf |
| cg12945194 | 6.15E-07 | GPHA2 | 1stExon | Open Sea |
| cg12965202 | 4.46E-09 | GTF2A1 | TSS1500 | S_Shore |
| cg12965512 | 1.41E-07 | TSPAN8 | TSS1500 | Open Sea |
| cg12974258 | 4.33E-07 | CSF1R | TSS200 | Open Sea |
| cg12979844 | 2.47E-08 | SIAH3 | TSS200 | Open Sea |
| cg12984086 | 2.96E-08 | GOLGA4 | TSS1500 | N_Shore |
| cg13001878 | 1.65E-07 | F10 | TSS200 | Open Sea |
| cg13009087 | 9.41E-08 | PLCH1 | TSS1500 | Open Sea |
| cg13011452 | 7.95E-07 | C6orf136 | TSS1500 | N_Shore |
| cg13015284 | 1.60E-07 | NRXN2 | 1stExon | Island |
| cg13026773 | 3.25E-07 | FAM108C1 | TSS1500 | N_Shore |
| cg13030582 | 1.03E-07 | MFAP4 | TSS1500 | Open Sea |
| cg13032616 | 9.13E-07 | TPBG | TSS1500 | N_Shore |
| cg13046271 | 5.48E-07 | TCF7 | TSS200 | Island |
| cg13046608 | 6.62E-07 | SLC43A3 | TSS1500 | S_Shore |
| cg13048147 | 4.58E-08 | ATP5H | TSS1500 | S_Shore |
| cg13048591 | 2.66E-07 | HSPB7 | TSS1500 | Open Sea |
| cg13055001 | 1.80E-07 | PPP1CA | TSS1500 | S_Shore |
| cg13067816 | 2.70E-07 | GSTTP2 | TSS200 | Open Sea |
| cg13069385 | 6.80E-09 | KRT222 | TSS1500 | Open Sea |
| cg13094752 | 5.83E-07 | MMP14 | TSS1500 | N_Shore |
| cg13097993 | 7.40E-07 | ATL1 | TSS200 | N_Shore |
| cg13100753 | 9.52E-08 | ATP6V1G3 | TSS200 | Open Sea |
| cg13102294 | 6.74E-07 | PPT2 | TSS1500 | N_Shore |
| cg13102889 | 3.30E-10 | HES5 | TSS1500 | S_Shore |
| cg13107973 | 9.91E-07 | KCNK2 | TSS1500 | Open Sea |
| cg13119182 | 7.01E-08 | SLC10A6 | TSS1500 | Open Sea |
| cg13119853 | 7.64E-07 | TOB1 | 1stExon | N_Shelf |
| cg13123009 | 2.84E-07 | LY6G6E | TSS200 | Open Sea |
| cg13126638 | 7.71E-07 | SCNN1A | TSS1500 | Open Sea |
| cg13128596 | 9.89E-09 | CASP4 | TSS1500 | Open Sea |
| cg13138089 | 6.86E-07 | ECEL1P2 | TSS200 | Island |
| cg13145528 | 5.41E-07 | OR2F2 | TSS1500 | Open Sea |
| cg13149442 | 6.22E-08 | SLC14A1 | TSS1500 | Open Sea |
| cg13149890 | 4.47E-08 | ATP1A2 | TSS1500 | Open Sea |
| cg13172432 | 2.47E-07 | SLC26A9 | TSS1500 | Open Sea |
| cg13173552 | 1.52E-07 | FBLIM1 | 1stExon | Open Sea |
| cg13179074 | 8.30E-09 | DUSP27 | 1stExon | Open Sea |
| cg13179549 | 5.95E-08 | KRT16 | TSS200 | Open Sea |
| cg13187009 | 4.66E-07 | RBM38 | TSS1500 | N_Shore |
| cg13192836 | 4.67E-07 | HAS2AS | TSS1500 | N_Shore |
| cg13195486 | 3.50E-07 | RRP15 | TSS1500 | N_Shore |
| cg13201297 | 1.12E-08 | LINGO4 | 1stExon | Open Sea |
| cg13206063 | 1.86E-07 | MICAL1 | TSS1500 | Island |
| cg13209298 | 1.47E-07 | SEMA3B | TSS1500 | Open Sea |
| cg13210467 | 1.39E-07 | STAG3 | TSS200 | Island |
| cg13211181 | 7.24E-08 | IFLTD1 | 1stExon | Open Sea |
| cg13244211 | 3.02E-07 | SNORA19 | TSS1500 | Open Sea |
| cg13247398 | 1.87E-07 | DGKZ | TSS200 | N_Shore |
| cg13248581 | 1.91E-07 | CECR1 | TSS200 | Open Sea |
| cg13292202 | 3.80E-09 | TSSK1B | TSS1500 | Open Sea |
| cg13300273 | 4.08E-08 | GPR25 | 1stExon | Island |
| cg13300939 | 9.74E-07 | GPR37L1 | TSS200 | Open Sea |
| cg13307058 | 4.86E-07 | SGK1 | TSS1500 | Island |
| cg13319266 | 1.50E-09 | TMEM72 | TSS200 | Open Sea |
| cg13319446 | 8.94E-07 | LOC283663 | TSS1500 | Open Sea |
| cg13320181 | 3.57E-07 | HSPB7 | TSS200 | Open Sea |
| cg13324128 | 1.84E-07 | TOP1 | TSS1500 | N_Shore |
| cg13334727 | 5.03E-07 | SEPT8 | TSS200 | Island |
| cg13335054 | 3.69E-07 | LOC100131551 | TSS1500 | N_Shelf |
| cg13344757 | 3.23E-08 | ALDOC | TSS1500 | Open Sea |
| cg13347910 | 9.46E-07 | GCNT2 | TSS200 | Open Sea |
| cg13351161 | 7.50E-07 | SCARA3 | TSS1500 | N_Shore |
| cg13352306 | 1.44E-07 | EML3 | TSS1500 | S_Shore |
| cg13356175 | 6.34E-07 | S100A7L2 | TSS1500 | Open Sea |
| cg13356253 | 2.29E-07 | LOC100302652 | TSS200 | Island |
| cg13357229 | 4.41E-07 | SFRP2 | TSS1500 | N_Shore |
| cg13358623 | 6.53E-07 | FIGF | TSS200 | Open Sea |
| cg13366537 | 9.01E-07 | SCRN1 | TSS1500 | Island |
| cg13377324 | 9.27E-09 | HSD17B7 | TSS1500 | N_Shore |
| cg13377885 | 3.44E-07 | CEP120 | TSS1500 | S_Shore |
| cg13381984 | 2.00E-08 | LEP | 1stExon | Island |
| cg13382768 | 2.41E-09 | GGT1 | TSS1500 | Open Sea |
| cg13388731 | 8.01E-08 | DNAJB2 | TSS1500 | N_Shore |
| cg13390800 | 2.98E-07 | DCHS1 | TSS1500 | S_Shore |
| cg13392029 | 3.19E-09 | ARTN | TSS1500 | N_Shore |
| cg13393036 | 2.22E-07 | TP53INP1 | TSS1500 | Island |
| cg13393580 | 1.51E-07 | MXRA8 | TSS1500 | S_Shelf |
| cg13393917 | 1.36E-08 | OLFML3 | TSS1500 | Open Sea |
| cg13396738 | 1.73E-07 | DDR1 | TSS1500 | N_Shore |
| cg13402847 | 9.71E-08 | RHOBTB2 | TSS200 | Open Sea |
| cg13408152 | 1.72E-07 | FSTL1 | TSS1500 | Island |
| cg13411229 | 8.60E-07 | SLURP1 | TSS200 | S_Shelf |
| cg13419925 | 3.00E-08 | MFSD1 | TSS1500 | N_Shore |
| cg13423172 | 2.56E-07 | CLDN22 | 1stExon | Open Sea |
| cg13423770 | 2.34E-08 | RHBG | TSS1500 | Island |
| cg13430376 | 1.15E-10 | KRT13 | TSS1500 | Open Sea |
| cg13434757 | 5.25E-08 | SLC22A1 | TSS1500 | Open Sea |
| cg13443575 | 5.78E-09 | SLFN13 | TSS200 | N_Shore |
| cg13444413 | 1.25E-07 | AGAP2 | 1stExon | Island |
| cg13446622 | 1.14E-11 | SDR9C7 | TSS1500 | Open Sea |
| cg13451637 | 8.63E-08 | C11orf40 | TSS1500 | Open Sea |
| cg13453168 | 1.24E-07 | PTPRE | TSS200 | Open Sea |
| cg13458210 | 3.11E-08 | TSTD1 | TSS1500 | S_Shore |
| cg13459606 | 7.11E-08 | GRB10 | TSS1500 | S_Shore |
| cg13461273 | 4.01E-07 | ZNF620 | TSS1500 | N_Shore |
| cg13463203 | 2.99E-07 | FREQ | TSS1500 | Open Sea |
| cg13463639 | 7.21E-07 | SIGIRR | TSS1500 | S_Shore |
| cg13465995 | 5.80E-07 | SURF6 | TSS1500 | S_Shore |
| cg13470063 | 4.33E-07 | TIE1 | TSS1500 | Open Sea |
| cg13475578 | 1.78E-07 | ANKS1B | TSS200 | Open Sea |
| cg13488201 | 6.60E-08 | ADAM12 | TSS200 | Island |
| cg13492337 | 3.62E-07 | PXDNL | TSS200 | Open Sea |
| cg13494428 | 1.91E-07 | AKR7A2 | TSS1500 | S_Shore |
| cg13495118 | 3.96E-07 | TM4SF18 | TSS200 | Open Sea |
| cg13518442 | 2.58E-07 | ALDH1A2 | TSS1500 | S_Shore |
| cg13521286 | 5.54E-10 | FAM189A2 | TSS1500 | N_Shore |
| cg13523819 | 9.10E-08 | MITF | 1stExon | Open Sea |
| cg13530946 | 1.60E-08 | IARS2 | TSS1500 | N_Shore |
| cg13531120 | 1.60E-07 | C13orf29 | TSS1500 | Open Sea |
| cg13535489 | 5.56E-07 | LILRB5 | TSS1500 | Open Sea |
| cg13537510 | 2.19E-07 | MOG | TSS1500 | Open Sea |
| cg13549461 | 1.75E-08 | CALD1 | TSS1500 | Open Sea |
| cg13555278 | 4.88E-07 | EXTL1 | 1stExon | Open Sea |
| cg13555662 | 3.43E-07 | LCN9 | TSS1500 | Open Sea |
| cg13558754 | 1.54E-08 | HSPB6 | 1stExon | Island |
| cg13560452 | 9.05E-09 | OLIG3 | 1stExon | N_Shore |
| cg13561081 | 1.55E-08 | DNAJC15 | TSS1500 | N_Shore |
| cg13561879 | 2.82E-07 | UNC5D | TSS1500 | Island |
| cg13570660 | 3.67E-07 | ZNF483 | TSS200 | N_Shore |
| cg13586457 | 3.16E-07 | SPTBN5 | TSS1500 | Open Sea |
| cg13586599 | 2.58E-07 | SCGN | TSS200 | Island |
| cg13590277 | 7.90E-07 | SYNPO | TSS1500 | Open Sea |
| cg13590876 | 2.17E-07 | INF2 | TSS1500 | N_Shore |
| cg13603171 | 5.84E-07 | MOXD1 | TSS1500 | S_Shore |
| cg13607226 | 5.69E-07 | GGTA1 | TSS1500 | S_Shore |
| cg13610769 | 1.08E-07 | ALS2CR11 | TSS1500 | S_Shore |
| cg13612207 | 1.84E-07 | MDFI | TSS1500 | Island |
| cg13613439 | 1.44E-07 | PHYHD1 | 1stExon | Open Sea |
| cg13619772 | 1.53E-07 | C1orf49 | TSS1500 | Open Sea |
| cg13620526 | 1.15E-07 | IRF9 | TSS1500 | Open Sea |
| cg13620799 | 1.01E-07 | LNX1 | 1stExon | Open Sea |
| cg13631444 | 1.53E-09 | TRHR | TSS1500 | Open Sea |
| cg13645954 | 9.30E-08 | ASB10 | TSS1500 | Open Sea |
| cg13646005 | 1.08E-07 | MUM1 | TSS200 | S_Shore |
| cg13647528 | 2.88E-07 | GRAMD1B | TSS200 | Open Sea |
| cg13652336 | 3.78E-07 | PREX2 | TSS1500 | N_Shore |
| cg13654183 | 5.83E-07 | TNXB | TSS1500 | Open Sea |
| cg13655803 | 2.92E-08 | WFIKKN2 | 1stExon | Open Sea |
| cg13675849 | 2.43E-07 | TRPV5 | TSS1500 | Open Sea |
| cg13679778 | 2.64E-09 | GPX5 | TSS1500 | Open Sea |
| cg13683534 | 6.46E-08 | GPSM1 | TSS1500 | Open Sea |
| cg13684305 | 6.37E-07 | C1orf65 | TSS1500 | N_Shore |
| cg13694680 | 7.98E-08 | FIBIN | 1stExon | Open Sea |
| cg13694927 | 1.08E-07 | HOXA5 | TSS1500 | Island |
| cg13697715 | 6.28E-07 | LOC202181 | TSS1500 | S_Shore |
| cg13699009 | 1.28E-07 | WDR66 | TSS1500 | N_Shore |
| cg13704497 | 2.23E-07 | SNORA38 | TSS1500 | S_Shore |
| cg13706582 | 9.68E-07 | ZNF385D | TSS1500 | Open Sea |
| cg13712154 | 7.11E-08 | DIAPH2 | TSS1500 | N_Shore |
| cg13714844 | 3.41E-08 | GNG10 | TSS1500 | N_Shore |
| cg13715010 | 6.42E-07 | GTF3C5 | TSS1500 | N_Shore |
| cg13725803 | 3.89E-07 | GUCY1B2 | TSS200 | Open Sea |
| cg13729116 | 5.59E-07 | LETM1 | TSS1500 | S_Shore |
| cg13732865 | 1.91E-07 | SFRP2 | TSS1500 | N_Shore |
| cg13734518 | 3.54E-08 | DLD | TSS1500 | N_Shore |
| cg13738195 | 1.20E-07 | DCHS2 | TSS1500 | S_Shore |
| cg13746919 | 3.78E-08 | C10orf57 | TSS1500 | N_Shore |
| cg13750441 | 7.75E-07 | PXK | TSS1500 | N_Shore |
| cg13751330 | 9.82E-07 | POMT1 | TSS1500 | N_Shore |
| cg13753515 | 8.61E-08 | SIGLEC1 | TSS200 | Open Sea |
| cg13761998 | 2.85E-07 | GRB7 | TSS200 | Open Sea |
| cg13762320 | 1.57E-07 | ASCL2 | TSS1500 | Island |
| cg13765621 | 7.90E-08 | CD1D | TSS1500 | N_Shore |
| cg13772174 | 9.41E-07 | CNN1 | TSS1500 | Open Sea |
| cg13774825 | 4.71E-07 | AKT1 | TSS1500 | S_Shore |
| cg13777609 | 2.31E-07 | CCL27 | TSS1500 | N_Shore |
| cg13784855 | 3.94E-07 | MIR1204 | TSS1500 | S_Shore |
| cg13798289 | 4.04E-08 | THAP11 | TSS1500 | N_Shore |
| cg13802789 | 5.71E-07 | GARNL3 | 1stExon | Open Sea |
| cg13804182 | 1.74E-07 | FLOT1 | TSS1500 | Island |
| cg13808198 | 1.43E-07 | ERRFI1 | TSS1500 | S_Shore |
| cg13823701 | 6.07E-08 | TNXB | TSS1500 | Open Sea |
| cg13825083 | 5.56E-09 | GGT1 | TSS1500 | Open Sea |
| cg13836098 | 7.57E-07 | HIST1H3E | TSS200 | N_Shore |
| cg13841742 | 9.61E-07 | OCIAD2 | TSS1500 | S_Shore |
| cg13855776 | 3.74E-07 | C10orf71 | TSS1500 | Open Sea |
| cg13857119 | 5.45E-07 | PLEKHG5 | TSS1500 | Open Sea |
| cg13857519 | 1.46E-07 | TPH1 | TSS1500 | Open Sea |
| cg13858106 | 2.00E-08 | OBSL1 | TSS1500 | S_Shore |
| cg13860849 | 1.12E-08 | CD248 | 1stExon | Island |
| cg13861948 | 7.44E-07 | LOC400804 | TSS200 | Open Sea |
| cg13866190 | 5.23E-09 | FLJ14107 | TSS200 | Open Sea |
| cg13868361 | 1.70E-08 | GTF2B | TSS1500 | S_Shore |
| cg13870494 | 6.12E-07 | MAMDC2 | TSS200 | N_Shore |
| cg13870539 | 5.06E-07 | PLCE1 | TSS200 | Open Sea |
| cg13871633 | 5.58E-08 | ZNF662 | TSS1500 | N_Shore |
| cg13871826 | 3.89E-07 | CYR61 | TSS1500 | N_Shore |
| cg13876650 | 3.73E-07 | FAM54B | TSS1500 | N_Shore |
| cg13879455 | 2.68E-07 | AGAP2 | TSS1500 | S_Shore |
| cg13879776 | 5.37E-07 | CLDN11 | TSS1500 | Island |
| cg13881341 | 8.26E-07 | FGF21 | TSS200 | S_Shelf |
| cg13888838 | 4.43E-08 | NHEJ1 | TSS200 | S_Shore |
| cg13889425 | 1.03E-07 | WDR85 | TSS1500 | S_Shore |
| cg13891189 | 9.14E-09 | ALAD | TSS1500 | S_Shore |
| cg13913407 | 2.64E-07 | C1QTNF2 | TSS1500 | S_Shore |
| cg13927938 | 3.05E-10 | PIK3C2B | TSS200 | N_Shelf |
| cg13928306 | 2.07E-07 | ZNF205 | TSS200 | Open Sea |
| cg13928759 | 2.49E-07 | LGI1 | TSS200 | Open Sea |
| cg13935558 | 5.49E-09 | TRIM24 | TSS1500 | N_Shore |
| cg13962321 | 1.18E-07 | HIST2H2BF | 1stExon | Island |
| cg13975098 | 2.41E-08 | RGS10 | TSS1500 | S_Shore |
| cg13980799 | 6.75E-08 | SLC18A2 | TSS1500 | N_Shore |
| cg13981356 | 7.51E-07 | IMPACT | TSS1500 | Island |
| cg13983640 | 8.69E-13 | TSPO2 | TSS1500 | Open Sea |
| cg13985518 | 1.07E-09 | HOXA2 | TSS1500 | S_Shore |
| cg13993274 | 9.23E-07 | AK3 | TSS1500 | S_Shore |
| cg13997068 | 6.39E-07 | TPSG1 | TSS1500 | Open Sea |
| cg13998850 | 1.38E-07 | CPNE6 | 1stExon | Open Sea |
| cg14002185 | 4.26E-09 | KRT23 | TSS200 | Open Sea |
| cg14010405 | 7.58E-09 | GTF2B | TSS1500 | S_Shore |
| cg14015441 | 3.79E-07 | DPYS | TSS200 | Island |
| cg14016390 | 1.91E-08 | C1QTNF8 | TSS200 | S_Shelf |
| cg14018690 | 5.14E-08 | C3orf52 | TSS1500 | N_Shore |
| cg14020824 | 8.19E-07 | FLJ20184 | TSS1500 | Open Sea |
| cg14020904 | 2.78E-07 | C1QTNF1 | TSS1500 | N_Shore |
| cg14021168 | 5.61E-09 | ODF4 | TSS1500 | Open Sea |
| cg14021375 | 2.18E-07 | F2RL3 | TSS200 | N_Shore |
| cg14021880 | 2.79E-09 | EMILIN1 | TSS200 | N_Shelf |
| cg14025021 | 1.66E-07 | MAP1A | TSS1500 | Open Sea |
| cg14027234 | 3.13E-08 | CD248 | 1stExon | Island |
| cg14030728 | 4.29E-07 | NPR2 | 1stExon | S_Shore |
| cg14037354 | 5.82E-07 | MIR654 | TSS1500 | Open Sea |
| cg14039909 | 2.66E-07 | GUCY2E | TSS1500 | Open Sea |
| cg14044120 | 3.61E-07 | ENTPD2 | TSS1500 | S_Shore |
| cg14068657 | 1.14E-08 | NCRNA00092 | TSS1500 | S_Shore |
| cg14069412 | 5.48E-08 | TXNDC15 | TSS200 | N_Shore |
| cg14073716 | 1.22E-07 | EEF1G | TSS1500 | S_Shore |
| cg14074251 | 2.38E-07 | SPEG | TSS1500 | N_Shore |
| cg14075424 | 5.89E-07 | NANOS3 | 1stExon | Island |
| cg14076977 | 1.75E-07 | PLCB2 | TSS200 | Open Sea |
| cg14077898 | 6.23E-07 | SLC17A8 | TSS200 | Open Sea |
| cg14080585 | 2.83E-08 | TAF4 | 1stExon | Island |
| cg14084888 | 9.74E-09 | UTP23 | TSS1500 | N_Shore |
| cg14086013 | 2.93E-07 | MDFI | TSS1500 | Island |
| cg14091103 | 7.45E-07 | LPP | TSS1500 | N_Shore |
| cg14092276 | 5.68E-07 | DLK1 | TSS1500 | Island |
| cg14101638 | 1.01E-08 | HNF1A | 1stExon | Island |
| cg14102437 | 9.39E-07 | LTBP1 | 1stExon | Open Sea |
| cg14103343 | 6.09E-08 | MAMSTR | TSS200 | N_Shelf |
| cg14108840 | 1.23E-10 | HOXC4 | TSS1500 | N_Shore |
| cg14108978 | 1.06E-07 | ARHGEF17 | 1stExon | S_Shore |
| cg14115756 | 1.86E-07 | GPR21 | TSS1500 | Open Sea |
| cg14122599 | 2.47E-08 | DAB2IP | TSS1500 | Open Sea |
| cg14123992 | 9.91E-07 | APOE | TSS1500 | N_Shelf |
| cg14130977 | 4.98E-09 | GP1BB | TSS200 | Island |
| cg14131220 | 9.02E-08 | CNTFR | TSS1500 | Island |
| cg14136186 | 4.03E-07 | NME2 | TSS200 | N_Shore |
| cg14150727 | 9.41E-09 | LGR6 | TSS1500 | N_Shore |
| cg14153069 | 1.44E-07 | PHYHD1 | 1stExon | Open Sea |
| cg14156381 | 4.45E-07 | FBN3 | TSS1500 | N_Shore |
| cg14160518 | 2.10E-08 | SALL4 | 1stExon | Island |
| cg14164080 | 1.92E-07 | GPSM1 | TSS1500 | Open Sea |
| cg14166756 | 7.53E-07 | ZNF774 | TSS1500 | N_Shore |
| cg14173599 | 5.20E-07 | BIRC7 | TSS200 | N_Shelf |
| cg14184817 | 4.28E-08 | EXOC3L2 | TSS200 | Island |
| cg14199723 | 2.10E-07 | PLEC1 | TSS1500 | Island |
| cg14202783 | 3.24E-07 | ST6GALNAC6 | TSS1500 | S_Shore |
| cg14204430 | 1.50E-09 | NDST4 | TSS200 | Open Sea |
| cg14205663 | 8.38E-11 | NR2F2 | TSS1500 | N_Shore |
| cg14205820 | 3.89E-07 | RPL13AP17 | TSS200 | Open Sea |
| cg14211097 | 8.13E-08 | GPR135 | 1stExon | N_Shore |
| cg14214182 | 6.60E-08 | NCRNA00092 | TSS1500 | S_Shore |
| cg14215689 | 3.37E-07 | INPP5E | TSS1500 | S_Shore |
| cg14223995 | 3.04E-07 | UCP1 | 1stExon | N_Shore |
| cg14232358 | 7.39E-07 | PINX1 | TSS1500 | S_Shore |
| cg14244577 | 1.72E-07 | DDX19B | TSS200 | Open Sea |
| cg14250984 | 2.30E-07 | EEF1G | TSS1500 | S_Shore |
| cg14254767 | 3.01E-07 | TJP2 | TSS200 | Open Sea |
| cg14256699 | 5.15E-07 | SOST | 1stExon | S_Shelf |
| cg14260530 | 3.57E-07 | AKNA | TSS1500 | N_Shelf |
| cg14264795 | 1.06E-07 | TBX5 | TSS1500 | N_Shore |
| cg14281922 | 6.00E-08 | LDB3 | TSS200 | Open Sea |
| cg14292823 | 4.45E-08 | EEF1D | TSS1500 | Island |
| cg14301713 | 8.63E-10 | HOXC8 | TSS1500 | N_Shore |
| cg14312133 | 1.48E-07 | NCRNA00092 | TSS1500 | S_Shore |
| cg14321837 | 2.42E-08 | UXS1 | TSS1500 | S_Shore |
| cg14329157 | 4.15E-08 | WDR69 | TSS200 | N_Shore |
| cg14330078 | 7.69E-08 | LPP | TSS200 | Open Sea |
| cg14334389 | 1.12E-08 | SPARCL1 | TSS1500 | Open Sea |
| cg14345012 | 6.88E-07 | MIR663 | TSS1500 | Island |
| cg14354418 | 1.74E-08 | WIPF3 | 1stExon | Open Sea |
| cg14371292 | 2.28E-10 | MIR654 | TSS1500 | Open Sea |
| cg14374432 | 2.18E-07 | GRAMD1B | TSS200 | Open Sea |
| cg14375499 | 3.22E-08 | ALOX12 | TSS200 | Island |
| cg14380517 | 3.41E-07 | BTG3 | TSS1500 | S_Shore |
| cg14383549 | 1.83E-08 | DAB2IP | TSS1500 | Open Sea |
| cg14387743 | 4.70E-07 | VNN3 | TSS1500 | Open Sea |
| cg14391419 | 3.21E-08 | TWIST1 | TSS1500 | S_Shore |
| cg14391622 | 7.19E-07 | SLC12A8 | TSS1500 | S_Shore |
| cg14395885 | 2.18E-07 | DPM2 | TSS200 | S_Shore |
| cg14399060 | 7.75E-08 | HOXD4 | TSS1500 | Island |
| cg14410016 | 1.02E-09 | HOXD9 | TSS1500 | Island |
| cg14411375 | 2.33E-07 | NME2 | 1stExon | N_Shore |
| cg14413262 | 2.03E-07 | SLC45A1 | TSS1500 | N_Shore |
| cg14413700 | 3.21E-08 | FAM184A | TSS200 | Open Sea |
| cg14423778 | 3.51E-07 | MBNL1 | TSS1500 | N_Shore |
| cg14427009 | 3.31E-07 | PCDH17 | 1stExon | Island |
| cg14428178 | 1.52E-07 | GLP2R | TSS1500 | Open Sea |
| cg14428590 | 2.03E-08 | FSIP1 | TSS1500 | S_Shore |
| cg14435807 | 4.09E-09 | LOXL1 | TSS200 | Island |
| cg14442061 | 2.24E-07 | SREBF1 | TSS200 | Island |
| cg14448145 | 7.69E-07 | DNAJC5 | TSS1500 | N_Shore |
| cg14465237 | 3.83E-09 | CCDC73 | TSS200 | Open Sea |
| cg14473145 | 4.62E-07 | CLEC14A | TSS200 | S_Shore |
| cg14473924 | 6.03E-07 | PDZRN3 | TSS200 | Island |
| cg14476479 | 9.60E-07 | GPT | TSS1500 | S_Shore |
| cg14478475 | 2.15E-07 | MCART1 | TSS1500 | S_Shore |
| cg14481208 | 8.40E-08 | RTKN | TSS1500 | Island |
| cg14483935 | 2.36E-08 | TUBA1B | TSS1500 | S_Shore |
| cg14494090 | 1.60E-07 | KNDC1 | TSS1500 | N_Shore |
| cg14494812 | 6.00E-08 | TOB1 | 1stExon | N_Shore |
| cg14499385 | 4.12E-07 | SLC40A1 | TSS1500 | S_Shore |
| cg14507146 | 3.08E-07 | C1QTNF3 | TSS200 | Open Sea |
| cg14509403 | 5.48E-07 | HORMAD2 | TSS200 | Island |
| cg14520913 | 5.23E-07 | NNMT | TSS200 | Open Sea |
| cg14532519 | 8.33E-09 | TCF20 | TSS1500 | Open Sea |
| cg14547240 | 1.56E-07 | C1QTNF7 | TSS1500 | Open Sea |
| cg14550760 | 2.73E-08 | CEL | TSS1500 | Open Sea |
| cg14553824 | 9.10E-11 | IL22RA2 | TSS1500 | Open Sea |
| cg14564939 | 8.73E-09 | MYOM3 | 1stExon | Open Sea |
| cg14566475 | 2.67E-08 | GPIHBP1 | TSS200 | Open Sea |
| cg14567374 | 2.83E-08 | ANAPC2 | TSS1500 | S_Shore |
| cg14572467 | 8.83E-10 | TMPRSS13 | TSS200 | Open Sea |
| cg14577373 | 9.41E-08 | CYP20A1 | TSS1500 | N_Shore |
| cg14579118 | 5.73E-09 | BLVRA | TSS1500 | N_Shore |
| cg14588738 | 5.46E-07 | MIR548Q | TSS1500 | Open Sea |
| cg14592092 | 2.57E-07 | KCNE4 | TSS1500 | N_Shore |
| cg14593033 | 1.75E-07 | MKS1 | TSS200 | S_Shore |
| cg14601444 | 9.87E-07 | DCTN1 | TSS200 | Open Sea |
| cg14601868 | 4.15E-11 | HOXD8 | TSS1500 | N_Shore |
| cg14607642 | 6.13E-09 | LBR | TSS1500 | S_Shore |
| cg14608275 | 3.83E-07 | NCEH1 | TSS1500 | S_Shore |
| cg14609668 | 6.33E-07 | IL11RA | TSS1500 | Open Sea |
| cg14613470 | 2.94E-07 | KLHL3 | TSS200 | Open Sea |
| cg14617041 | 8.49E-07 | TMEM171 | TSS1500 | Island |
| cg14621053 | 2.12E-07 | ADAM12 | TSS200 | Island |
| cg14621323 | 9.68E-07 | PFN3 | 1stExon | Island |
| cg14622443 | 3.31E-09 |  | TSS1500 | S_Shore |
| cg14626525 | 6.27E-07 | MIR602 | TSS1500 | S_Shore |
| cg14631053 | 4.09E-07 | SSTR4 | TSS200 | Island |
| cg14640621 | 1.49E-07 | HMX2 | TSS1500 | N_Shore |
| cg14646075 | 1.52E-08 | WDR69 | TSS1500 | N_Shore |
| cg14674856 | 6.93E-08 | LTC4S | TSS1500 | N_Shelf |
| cg14676407 | 2.88E-07 | CPXM2 | 1stExon | Island |
| cg14688272 | 1.59E-07 | FN3KRP | TSS1500 | N_Shore |
| cg14690065 | 3.45E-08 | APOL6 | TSS200 | Open Sea |
| cg14703589 | 2.39E-07 | LIMK2 | 1stExon | Open Sea |
| cg14706455 | 1.48E-09 | HEG1 | TSS1500 | S_Shore |
| cg14708990 | 1.02E-07 | MYO7A | TSS200 | Open Sea |
| cg14710465 | 5.96E-07 | VANGL2 | TSS1500 | N_Shore |
| cg14712784 | 3.30E-07 | TMEM179B | TSS1500 | N_Shore |
| cg14719129 | 5.40E-07 | MIR302D | TSS1500 | Open Sea |
| cg14719352 | 1.47E-07 | FGF21 | TSS1500 | S_Shore |
| cg14725537 | 5.59E-07 | PLP1 | 1stExon | Open Sea |
| cg14742211 | 2.29E-07 | CLCNKA | TSS200 | Open Sea |
| cg14753355 | 1.80E-07 | F2RL3 | TSS200 | N_Shore |
| cg14753599 | 8.23E-07 | C10orf116 | 1stExon | Island |
| cg14754581 | 1.95E-08 | CCRL2 | TSS1500 | Open Sea |
| cg14762670 | 8.99E-07 | CACNA2D1 | TSS1500 | S_Shore |
| cg14766231 | 8.09E-07 | TTC32 | TSS1500 | S_Shore |
| cg14769792 | 6.79E-08 | FAM13AOS | TSS1500 | Open Sea |
| cg14772955 | 6.61E-07 | KIAA0319L | TSS1500 | S_Shore |
| cg14773523 | 2.92E-08 | MINA | TSS1500 | S_Shore |
| cg14777903 | 4.38E-07 | HRASLS | TSS1500 | N_Shore |
| cg14780416 | 3.60E-07 | MIR196B | TSS200 | Island |
| cg14791081 | 4.79E-08 | ALS2CR4 | TSS1500 | S_Shore |
| cg14792480 | 5.47E-08 | CDH16 | TSS1500 | N_Shore |
| cg14795572 | 2.36E-08 | AGAP2 | 1stExon | Island |
| cg14802355 | 1.78E-08 | MANBA | TSS1500 | S_Shore |
| cg14830003 | 2.01E-07 | DNALI1 | 1stExon | Island |
| cg14833706 | 9.84E-07 | ZXDB | TSS1500 | N_Shore |
| cg14837598 | 6.80E-07 | EPB49 | TSS200 | S_Shelf |
| cg14838970 | 4.02E-07 | PPFIBP2 | TSS1500 | N_Shore |
| cg14841203 | 6.94E-07 | ULBP1 | TSS1500 | N_Shore |
| cg14870271 | 9.89E-08 | LGALS3BP | 1stExon | Open Sea |
| cg14876077 | 2.18E-07 | EXOC3L2 | TSS1500 | Island |
| cg14895298 | 2.47E-08 | BIRC7 | TSS200 | N_Shelf |
| cg14900246 | 6.06E-07 | MERTK | TSS1500 | N_Shore |
| cg14903689 | 1.66E-07 | COL18A1 | TSS1500 | N_Shore |
| cg14904725 | 4.08E-07 | F10 | TSS200 | Open Sea |
| cg14904908 | 3.88E-10 | SFRP1 | TSS1500 | S_Shore |
| cg14942952 | 7.18E-09 | PARVG | TSS1500 | Open Sea |
| cg14944362 | 4.00E-07 | PDYN | TSS1500 | Open Sea |
| cg14944538 | 3.40E-07 | MYOM1 | TSS1500 | Open Sea |
| cg14945774 | 6.33E-08 | LRP5L | TSS200 | S_Shelf |
| cg14950751 | 8.77E-07 | SLC16A11 | TSS1500 | Island |
| cg14961476 | 3.07E-08 | SLC2A5 | TSS1500 | N_Shore |
| cg14974772 | 5.93E-07 | FBLN5 | TSS1500 | S_Shore |
| cg14983728 | 2.63E-07 | TOM1 | TSS1500 | N_Shore |
| cg14983771 | 8.62E-10 | MNAT1 | TSS1500 | Open Sea |
| cg14990478 | 1.55E-07 | TM2D1 | TSS1500 | S_Shore |
| cg14990644 | 8.06E-09 | TSSK1B | TSS1500 | Open Sea |
| cg14996810 | 9.55E-09 | HNF1A | TSS200 | Island |
| cg15012282 | 2.98E-07 | TMEM14E | TSS1500 | Open Sea |
| cg15013617 | 1.41E-07 | C20orf141 | TSS1500 | Open Sea |
| cg15013801 | 1.29E-09 | ASCC1 | TSS1500 | S_Shore |
| cg15026767 | 1.85E-07 | S100A3 | TSS200 | Open Sea |
| cg15028128 | 1.24E-07 | MIR135A2 | TSS1500 | Open Sea |
| cg15033552 | 2.21E-07 | CAPN2 | TSS200 | Open Sea |
| cg15044270 | 5.36E-07 | TM4SF19 | TSS1500 | Open Sea |
| cg15044573 | 8.47E-07 | FAM53B | TSS1500 | S_Shore |
| cg15047774 | 3.85E-07 | ASAM | TSS1500 | S_Shore |
| cg15056189 | 6.39E-07 | ADCY6 | 1stExon | Island |
| cg15056348 | 7.19E-08 | INPP5A | TSS1500 | N_Shore |
| cg15058557 | 9.12E-08 | SYNC | TSS1500 | S_Shore |
| cg15062470 | 1.03E-09 | FNDC4 | TSS1500 | S_Shore |
| cg15067907 | 7.60E-07 | FUT6 | 1stExon | Open Sea |
| cg15075897 | 2.37E-09 | DPPA3 | TSS1500 | S_Shelf |
| cg15079885 | 1.14E-07 | TINAGL1 | TSS200 | Open Sea |
| cg15095906 | 1.84E-08 | FAM176B | TSS200 | S_Shore |
| cg15096140 | 1.92E-07 | MYO1B | TSS1500 | N_Shore |
| cg15100426 | 3.37E-07 | PNKD | TSS1500 | N_Shore |
| cg15101392 | 4.21E-07 | FXYD6 | TSS1500 | S_Shore |
| cg15102749 | 3.08E-07 | C11orf80 | TSS1500 | N_Shore |
| cg15110101 | 1.43E-07 | BEST1 | TSS200 | Open Sea |
| cg15114651 | 9.47E-08 | SLC1A5 | TSS1500 | N_Shore |
| cg15119221 | 1.63E-08 | MFAP4 | TSS1500 | Open Sea |
| cg15120085 | 1.53E-07 | TINAGL1 | TSS200 | Open Sea |
| cg15120942 | 5.13E-07 | PLCD1 | TSS1500 | Island |
| cg15140562 | 5.03E-08 | TMEM89 | TSS200 | Open Sea |
| cg15153887 | 9.83E-07 | TREML1 | TSS1500 | Open Sea |
| cg15154229 | 6.34E-07 | CPA1 | TSS1500 | N_Shore |
| cg15156367 | 1.09E-07 | EPHX4 | TSS1500 | N_Shore |
| cg15158310 | 2.09E-09 | IGFL2 | TSS1500 | Open Sea |
| cg15162869 | 2.76E-09 | PISD | TSS1500 | S_Shore |
| cg15163151 | 2.39E-07 | COX6C | TSS1500 | S_Shore |
| cg15166523 | 9.73E-08 | VSIG4 | TSS1500 | Open Sea |
| cg15175162 | 5.70E-08 | FBXL5 | TSS1500 | Island |
| cg15177917 | 8.49E-08 | DAND5 | TSS200 | Open Sea |
| cg15190354 | 7.83E-08 | CHEK1 | TSS1500 | N_Shore |
| cg15197065 | 1.76E-07 | CMYA5 | TSS200 | Open Sea |
| cg15209376 | 9.42E-08 | ITGBL1 | 1stExon | N_Shore |
| cg15209808 | 1.10E-07 | HORMAD2 | TSS200 | Island |
| cg15224955 | 1.07E-08 | AKR1B15 | 1stExon | Open Sea |
| cg15227610 | 9.71E-07 | HSPB2 | TSS1500 | Open Sea |
| cg15228492 | 6.53E-07 | YEATS2 | TSS1500 | N_Shore |
| cg15232290 | 1.95E-07 | CTSD | TSS1500 | S_Shore |
| cg15232971 | 4.24E-08 | CCDC6 | TSS200 | S_Shore |
| cg15237857 | 5.56E-08 | FAM110C | 1stExon | Island |
| cg15238382 | 2.05E-07 | DIO3 | TSS200 | Island |
| cg15269226 | 1.60E-07 | PLXNB2 | TSS1500 | S_Shore |
| cg15278699 | 3.12E-08 | PWWP2B | TSS1500 | N_Shore |
| cg15282281 | 2.91E-08 | MIR346 | TSS200 | S_Shore |
| cg15283950 | 4.89E-08 | MRVI1 | 1stExon | Open Sea |
| cg15291476 | 2.35E-08 | FAM25A | TSS1500 | Open Sea |
| cg15320998 | 5.17E-08 | ZNF212 | TSS1500 | N_Shore |
| cg15321396 | 1.69E-07 | KIAA0895L | TSS1500 | Island |
| cg15326452 | 5.28E-07 | TCF23 | TSS200 | N_Shore |
| cg15331996 | 9.04E-08 | SPOCK2 | TSS200 | S_Shore |
| cg15336091 | 1.27E-07 | HIST1H4B | TSS1500 | S_Shore |
| cg15349474 | 3.43E-08 | CHEK1 | TSS1500 | N_Shore |
| cg15350036 | 1.45E-08 | CROT | TSS1500 | N_Shore |
| cg15353597 | 6.87E-08 | MAP4K4 | TSS1500 | N_Shore |
| cg15376972 | 1.98E-08 | PON2 | TSS1500 | S_Shore |
| cg15380603 | 8.29E-08 | BACH2 | TSS1500 | S_Shore |
| cg15381304 | 6.52E-07 | GPR6 | TSS200 | Island |
| cg15394255 | 6.07E-07 | MGAT4C | TSS200 | Open Sea |
| cg15412718 | 3.84E-09 | FLJ43860 | TSS1500 | Open Sea |
| cg15414132 | 3.51E-08 | SLC25A45 | TSS1500 | N_Shelf |
| cg15421520 | 3.18E-07 | C14orf180 | 1stExon | Open Sea |
| cg15433297 | 6.89E-08 | PKHD1L1 | 1stExon | Island |
| cg15439862 | 3.68E-07 | DSC3 | 1stExon | Island |
| cg15440512 | 9.27E-08 | SPNS2 | TSS1500 | N_Shore |
| cg15456144 | 9.93E-07 | C11orf75 | TSS1500 | S_Shore |
| cg15456821 | 2.36E-08 | BEST1 | TSS200 | Open Sea |
| cg15457079 | 1.28E-08 | CPN1 | TSS200 | Open Sea |
| cg15464226 | 2.22E-07 | TNFSF11 | 1stExon | Open Sea |
| cg15469709 | 1.32E-08 | C1orf161 | TSS1500 | Open Sea |
| cg15474579 | 5.99E-07 | CDKN1A | TSS1500 | N_Shore |
| cg15481791 | 2.56E-07 | SNRPE | TSS1500 | N_Shore |
| cg15487620 | 2.85E-08 | ATP1A2 | TSS1500 | Open Sea |
| cg15494458 | 6.49E-07 | BPI | TSS1500 | Open Sea |
| cg15495372 | 5.07E-07 | ZNF579 | TSS1500 | S_Shelf |
| cg15504662 | 3.35E-07 | LIMK2 | TSS200 | Open Sea |
| cg15507475 | 8.55E-07 | TMEM120A | TSS1500 | S_Shore |
| cg15532667 | 1.81E-07 | CRIP2 | TSS1500 | N_Shore |
| cg15537850 | 1.10E-07 | NAPRT1 | TSS1500 | S_Shore |
| cg15556672 | 1.40E-07 | MRPS36 | TSS1500 | N_Shore |
| cg15557878 | 1.64E-07 | TSLP | TSS1500 | N_Shelf |
| cg15585341 | 3.55E-07 | ITGA10 | 1stExon | Open Sea |
| cg15590133 | 3.28E-08 | SNORD116-24 | TSS1500 | Open Sea |
| cg15590780 | 8.80E-09 | USH2A | 1stExon | Open Sea |
| cg15594205 | 7.61E-07 | POU3F3 | TSS1500 | Island |
| cg15597855 | 2.96E-07 | VTCN1 | TSS200 | Open Sea |
| cg15617545 | 6.63E-07 | SERPING1 | TSS1500 | Open Sea |
| cg15628222 | 3.15E-07 | TRAM1 | TSS1500 | S_Shore |
| cg15648345 | 7.77E-07 | MKS1 | TSS1500 | S_Shore |
| cg15651758 | 1.05E-09 | CSN3 | 1stExon | Open Sea |
| cg15651941 | 4.17E-07 | KCNQ1OT1 | TSS1500 | Island |
| cg15655714 | 1.74E-07 | ASPDH | TSS1500 | N_Shore |
| cg15662902 | 7.14E-09 | TNNI2 | TSS1500 | Open Sea |
| cg15676719 | 2.29E-07 | RCSD1 | TSS1500 | N_Shore |
| cg15679098 | 6.23E-11 | RAPSN | 1stExon | Open Sea |
| cg15684563 | 3.35E-07 | TSPAN8 | TSS1500 | Open Sea |
| cg15692360 | 1.19E-08 | FERMT1 | TSS200 | S_Shore |
| cg15694987 | 9.90E-07 | EXT1 | TSS1500 | S_Shore |
| cg15696906 | 1.01E-07 | TRPC4 | TSS200 | Island |
| cg15697257 | 2.73E-07 | SYNPO | 1stExon | Open Sea |
| cg15700776 | 8.30E-07 | CYP2U1 | TSS1500 | N_Shore |
| cg15702185 | 7.59E-08 | MIRLET7A3 | TSS1500 | Open Sea |
| cg15703035 | 7.06E-07 | C13orf29 | TSS1500 | Open Sea |
| cg15709214 | 3.14E-10 | CALD1 | TSS1500 | Open Sea |
| cg15726326 | 9.03E-08 | ALDH3A1 | TSS1500 | S_Shelf |
| cg15728909 | 1.87E-07 | CDH19 | TSS1500 | Open Sea |
| cg15730857 | 3.63E-07 | ST7OT4 | TSS1500 | N_Shore |
| cg15731053 | 5.90E-07 | TMEM229A | TSS1500 | S_Shore |
| cg15733114 | 4.41E-08 | ART4 | TSS200 | Open Sea |
| cg15740508 | 4.02E-11 | AMPD1 | TSS1500 | Open Sea |
| cg15740759 | 7.71E-07 | ERCC1 | 1stExon | N_Shore |
| cg15743533 | 3.88E-07 | FAM110A | TSS1500 | S_Shore |
| cg15744134 | 3.87E-07 | SCG5 | TSS200 | Open Sea |
| cg15747595 | 1.01E-07 | TSPYL5 | 1stExon | Island |
| cg15747933 | 7.84E-08 | NMI | TSS200 | S_Shore |
| cg15763258 | 4.11E-07 | FLI1 | TSS1500 | N_Shore |
| cg15768226 | 2.46E-07 | RBM38 | TSS1500 | Island |
| cg15773251 | 1.91E-07 | TNXB | TSS1500 | Open Sea |
| cg15780361 | 4.30E-07 | ALS2CR11 | TSS200 | S_Shore |
| cg15781504 | 2.03E-10 | OR51S1 | TSS1500 | Open Sea |
| cg15782984 | 3.49E-08 | SLC26A8 | TSS1500 | N_Shore |
| cg15797834 | 5.55E-07 | STAP2 | TSS200 | Open Sea |
| cg15798153 | 1.83E-07 | CDK14 | 1stExon | Open Sea |
| cg15803221 | 1.06E-07 | RARA | TSS1500 | Island |
| cg15806304 | 2.03E-07 | SLC18A2 | TSS1500 | N_Shore |
| cg15813594 | 7.69E-07 | EGFLAM | TSS200 | Open Sea |
| cg15816503 | 5.17E-07 | C8orf74 | TSS200 | Open Sea |
| cg15817482 | 8.00E-10 | ASB2 | TSS200 | Open Sea |
| cg15823100 | 1.01E-09 | PISD | TSS1500 | S_Shore |
| cg15823698 | 7.33E-08 | RNF125 | TSS200 | N_Shore |
| cg15825417 | 9.68E-09 | MEIS3P1 | TSS1500 | Island |
| cg15826810 | 3.11E-07 | LOC285419 | TSS1500 | Open Sea |
| cg15835805 | 2.61E-07 | SHROOM1 | TSS1500 | S_Shore |
| cg15840005 | 5.30E-07 | C1orf86 | TSS1500 | S_Shelf |
| cg15840039 | 5.05E-07 | AR | TSS1500 | Open Sea |
| cg15845821 | 8.26E-07 | NWD1 | TSS200 | Open Sea |
| cg15854333 | 1.61E-09 | HSD17B8 | TSS1500 | N_Shore |
| cg15857661 | 6.74E-08 | MIR146B | TSS200 | S_Shelf |
| cg15863322 | 1.11E-09 | MTUS2 | 1stExon | Open Sea |
| cg15878685 | 1.01E-09 | UGT2A3 | TSS1500 | Open Sea |
| cg15881238 | 2.99E-07 | SLC10A6 | TSS1500 | Open Sea |
| cg15884223 | 3.48E-07 | NME2 | TSS200 | N_Shore |
| cg15885788 | 1.92E-07 | INTS2 | TSS1500 | S_Shore |
| cg15890075 | 4.25E-07 | MYO15A | TSS200 | Open Sea |
| cg15891218 | 1.93E-07 | TMEM30B | 1stExon | S_Shore |
| cg15892784 | 5.02E-07 | GPR25 | 1stExon | Island |
| cg15894467 | 8.09E-07 | C1orf190 | TSS1500 | N_Shore |
| cg15895197 | 8.03E-09 | EMILIN1 | 1stExon | N_Shelf |
| cg15901722 | 7.01E-09 | PCDH24 | TSS1500 | S_Shelf |
| cg15901783 | 1.93E-07 | KCTD12 | TSS1500 | S_Shore |
| cg15905329 | 9.72E-07 | CALD1 | TSS1500 | Open Sea |
| cg15906055 | 6.31E-07 | TDH | TSS1500 | Open Sea |
| cg15907473 | 3.79E-07 | GPR180 | TSS1500 | N_Shore |
| cg15912800 | 1.26E-07 | MIR196B | TSS200 | Island |
| cg15916628 | 4.47E-08 | ZNF579 | TSS1500 | S_Shore |
| cg15918732 | 8.25E-07 | SERPING1 | TSS1500 | Open Sea |
| cg15922246 | 3.51E-07 | FASTK | TSS1500 | Island |
| cg15926004 | 2.40E-07 | C1orf133 | TSS1500 | S_Shore |
| cg15926420 | 2.06E-07 | CASQ1 | TSS1500 | Open Sea |
| cg15926585 | 8.27E-07 | COMT | TSS1500 | N_Shore |
| cg15945754 | 1.08E-07 | C6orf204 | TSS1500 | S_Shore |
| cg15946133 | 7.58E-07 | CXCL13 | TSS1500 | Open Sea |
| cg15947301 | 1.09E-07 | TRPV3 | TSS1500 | Open Sea |
| cg15953921 | 5.52E-08 | RBM17 | TSS1500 | N_Shore |
| cg15965006 | 4.90E-07 | STK19 | TSS200 | N_Shore |
| cg15966757 | 2.36E-07 | SLC6A13 | TSS1500 | Open Sea |
| cg15978778 | 6.26E-08 | C7orf65 | TSS1500 | Open Sea |
| cg15979173 | 8.84E-08 | BARHL2 | 1stExon | Island |
| cg15991175 | 3.66E-07 | WDR82 | TSS1500 | S_Shore |
| cg15999796 | 9.77E-09 | MOG | TSS1500 | Open Sea |
| cg16001913 | 7.27E-07 | HK1 | TSS200 | Open Sea |
| cg16006965 | 5.67E-07 | GCET2 | TSS1500 | Open Sea |
| cg16008138 | 6.58E-11 |  | TSS200 | S_Shore |
| cg16014085 | 3.22E-07 | ZNF48 | TSS1500 | N_Shore |
| cg16014606 | 3.59E-07 | MRVI1 | 1stExon | Open Sea |
| cg16019612 | 6.92E-09 | PALM | TSS1500 | Island |
| cg16020904 | 7.67E-07 | SPATA20 | TSS1500 | N_Shore |
| cg16021126 | 3.17E-07 | SERP2 | TSS1500 | Island |
| cg16044595 | 6.67E-07 | GPR137B | TSS1500 | N_Shore |
| cg16047117 | 2.08E-07 | FAM109B | TSS200 | Island |
| cg16049391 | 1.27E-08 | BNC1 | TSS1500 | S_Shore |
| cg16051685 | 1.79E-07 | TRIM63 | 1stExon | Open Sea |
| cg16057826 | 2.15E-07 | CLIC6 | TSS1500 | Island |
| cg16063716 | 2.18E-07 | C14orf180 | TSS200 | Open Sea |
| cg16068038 | 9.67E-08 | C15orf28 | TSS1500 | Open Sea |
| cg16083447 | 1.84E-07 | C2orf64 | TSS1500 | S_Shore |
| cg16083838 | 4.64E-08 | KCNQ1DN | TSS1500 | N_Shore |
| cg16084190 | 3.30E-07 | BEST1 | TSS1500 | Open Sea |
| cg16091995 | 3.00E-07 | ALS2CR11 | TSS1500 | S_Shore |
| cg16092895 | 4.99E-08 | PPP1R1A | TSS1500 | S_Shore |
| cg16098340 | 5.82E-09 | MIR548C | TSS1500 | Open Sea |
| cg16109415 | 1.59E-08 | C7orf53 | TSS200 | Open Sea |
| cg16110455 | 2.52E-07 | HSPB7 | 1stExon | Open Sea |
| cg16112467 | 2.89E-08 | PRO1768 | TSS1500 | Open Sea |
| cg16113793 | 7.47E-07 | LAMA3 | TSS1500 | Open Sea |
| cg16121744 | 6.17E-08 | COL18A1 | TSS1500 | N_Shore |
| cg16131766 | 2.67E-09 | MKRN3 | TSS200 | Open Sea |
| cg16133681 | 1.64E-07 | IFLTD1 | TSS200 | Open Sea |
| cg16151082 | 3.28E-07 | PLEKHF2 | TSS1500 | N_Shore |
| cg16155588 | 3.94E-07 | CUL4A | TSS1500 | N_Shore |
| cg16158779 | 6.80E-07 | ANKK1 | TSS200 | Island |
| cg16162970 | 3.02E-08 | PACS2 | TSS1500 | N_Shore |
| cg16166968 | 3.52E-07 | ADAM30 | 1stExon | Island |
| cg16168153 | 2.44E-07 | SMPX | TSS200 | Open Sea |
| cg16168668 | 4.72E-08 | TWIST1 | TSS1500 | S_Shore |
| cg16175725 | 1.46E-09 | HNF1A | 1stExon | Island |
| cg16176600 | 5.44E-08 | FRK | 1stExon | Open Sea |
| cg16177693 | 1.03E-09 | NR0B2 | TSS200 | Open Sea |
| cg16198692 | 3.87E-08 | CDO1 | TSS1500 | S_Shore |
| cg16204414 | 4.59E-12 | C3orf42 | TSS1500 | Open Sea |
| cg16210447 | 6.28E-07 | CASS4 | TSS1500 | Open Sea |
| cg16210718 | 5.19E-07 | GP1BB | TSS200 | Island |
| cg16212219 | 4.33E-07 | MYLK | TSS1500 | Open Sea |
| cg16219535 | 1.79E-07 | RPS29 | TSS1500 | S_Shore |
| cg16224163 | 2.60E-07 | LPP | TSS1500 | N_Shore |
| cg16230982 | 2.03E-10 | PDE7A | TSS1500 | Open Sea |
| cg16240480 | 9.38E-07 | EDARADD | TSS1500 | N_Shore |
| cg16254374 | 6.25E-10 | BNC1 | TSS1500 | S_Shore |
| cg16258854 | 1.72E-07 | RHOB | 1stExon | S_Shore |
| cg16259591 | 3.74E-08 | PNLIP | TSS200 | Open Sea |
| cg16266925 | 9.40E-07 | TXNDC6 | TSS1500 | S_Shore |
| cg16279158 | 4.56E-07 | HBE1 | 1stExon | Open Sea |
| cg16290689 | 5.73E-08 | C13orf26 | TSS200 | Open Sea |
| cg16292132 | 4.30E-08 | NHSL1 | 1stExon | Open Sea |
| cg16293118 | 1.32E-08 | ITGBL1 | 1stExon | N_Shore |
| cg16310003 | 6.11E-07 | HPD | TSS1500 | Open Sea |
| cg16313910 | 4.73E-09 | SOX30 | 1stExon | Island |
| cg16317901 | 1.05E-07 | SEPT9 | TSS200 | Open Sea |
| cg16323609 | 2.35E-07 | PRKAR2A | TSS1500 | S_Shore |
| cg16326367 | 6.06E-09 | LHFPL2 | TSS1500 | S_Shore |
| cg16334524 | 7.08E-09 | C21orf84 | TSS200 | Open Sea |
| cg16337574 | 1.96E-07 | BLOC1S1 | TSS1500 | N_Shore |
| cg16345647 | 5.83E-07 | DSCR6 | TSS1500 | Island |
| cg16357582 | 4.80E-09 | CSRP1 | TSS200 | S_Shore |
| cg16358034 | 3.49E-08 | SNX18 | 1stExon | Island |
| cg16360432 | 4.96E-08 | TRPV6 | TSS200 | Open Sea |
| cg16360861 | 4.69E-07 | RAI14 | TSS200 | Open Sea |
| cg16374656 | 3.62E-08 | EXT1 | TSS1500 | S_Shore |
| cg16396933 | 1.63E-07 | NT5C2 | TSS1500 | S_Shore |
| cg16408593 | 1.27E-07 | VTCN1 | TSS200 | Open Sea |
| cg16425038 | 7.93E-08 | REC8 | TSS200 | Island |
| cg16427315 | 5.05E-08 | CCR3 | TSS1500 | Open Sea |
| cg16432350 | 1.38E-08 | KRT80 | 1stExon | Open Sea |
| cg16438210 | 5.88E-08 | CCDC88C | TSS1500 | S_Shore |
| cg16440442 | 5.47E-09 | SYNPO | 1stExon | Open Sea |
| cg16454426 | 2.41E-08 | C8orf22 | TSS1500 | Open Sea |
| cg16456919 | 1.33E-07 | LRRN2 | TSS1500 | S_Shore |
| cg16463700 | 5.04E-09 | SULT1C4 | TSS1500 | Open Sea |
| cg16473141 | 8.47E-09 | REC8 | 1stExon | Island |
| cg16477774 | 5.64E-07 | LTBP3 | 1stExon | Island |
| cg16483033 | 1.87E-07 | ABR | 1stExon | Open Sea |
| cg16489360 | 3.41E-08 | TMEM209 | TSS1500 | S_Shore |
| cg16489926 | 2.99E-08 | CDH19 | TSS200 | Open Sea |
| cg16493531 | 3.64E-08 | FLOT1 | TSS1500 | Island |
| cg16501323 | 1.12E-07 | AIRE | TSS200 | Island |
| cg16506185 | 9.52E-10 | C14orf48 | TSS200 | Open Sea |
| cg16509710 | 3.55E-09 | LOC402644 | TSS200 | Open Sea |
| cg16512990 | 9.24E-11 | FAM170B | TSS200 | S_Shore |
| cg16516295 | 6.56E-07 | C3orf25 | TSS1500 | S_Shore |
| cg16519300 | 1.06E-07 | FBLIM1 | TSS1500 | N_Shore |
| cg16520038 | 3.20E-08 | MIR130A | TSS200 | Open Sea |
| cg16523463 | 9.67E-09 | MYL2 | 1stExon | Open Sea |
| cg16527552 | 6.89E-08 | TMEM145 | TSS1500 | N_Shore |
| cg16530165 | 2.35E-07 | NPPC | TSS1500 | S_Shore |
| cg16534307 | 4.43E-09 | CREB5 | 1stExon | S_Shelf |
| cg16536824 | 9.79E-08 | PLA2G7 | TSS1500 | S_Shore |
| cg16544169 | 8.66E-07 | NKX2-6 | TSS1500 | Island |
| cg16548780 | 3.68E-07 | KIAA0895L | TSS1500 | Island |
| cg16550453 | 1.92E-07 | TDRD1 | TSS200 | Island |
| cg16559243 | 3.12E-07 | EGFLAM | TSS200 | Open Sea |
| cg16563370 | 6.32E-09 | SLFN13 | TSS200 | N_Shore |
| cg16567290 | 6.95E-07 | SLC26A10 | TSS200 | Island |
| cg16570507 | 1.75E-08 | MIR346 | TSS200 | S_Shore |
| cg16575322 | 4.31E-07 | PEBP4 | TSS1500 | Open Sea |
| cg16581199 | 1.61E-09 | TSSK1B | 1stExon | Open Sea |
| cg16582174 | 9.90E-07 | CLINT1 | TSS1500 | S_Shore |
| cg16588417 | 2.62E-07 | MIR1246 | TSS1500 | Open Sea |
| cg16591681 | 2.30E-07 | CLCNKA | TSS1500 | Open Sea |
| cg16597737 | 1.24E-09 | SLC9A11 | TSS200 | Open Sea |
| cg16601489 | 3.50E-10 | SNORD116-4 | TSS1500 | Open Sea |
| cg16617366 | 6.54E-07 | KIAA0427 | TSS1500 | N_Shore |
| cg16617723 | 8.89E-07 | AQP4 | TSS1500 | S_Shore |
| cg16619193 | 2.49E-07 | IKBKE | TSS1500 | Open Sea |
| cg16627247 | 2.58E-07 | SNORA80 | TSS200 | Open Sea |
| cg16627285 | 5.58E-07 | SGCA | TSS200 | Open Sea |
| cg16627786 | 1.90E-07 | GP1BB | TSS200 | Island |
| cg16631088 | 1.67E-08 | C16orf45 | TSS200 | N_Shore |
| cg16642695 | 1.28E-10 | ADHFE1 | TSS1500 | N_Shore |
| cg16648137 | 5.13E-09 | GOT1L1 | TSS1500 | Open Sea |
| cg16649728 | 3.06E-08 | DRAP1 | TSS1500 | Island |
| cg16651126 | 8.35E-07 | HOXA4 | TSS200 | Island |
| cg16653138 | 4.31E-07 | PRKCZ | 1stExon | Island |
| cg16658460 | 1.92E-07 | ODZ2 | TSS200 | Open Sea |
| cg16658862 | 1.81E-07 | OPN4 | TSS1500 | Open Sea |
| cg16667508 | 6.17E-07 | MATN4 | 1stExon | S_Shore |
| cg16677191 | 4.92E-07 | GLRX | TSS1500 | Open Sea |
| cg16678564 | 9.01E-07 | EPB49 | 1stExon | S_Shelf |
| cg16681526 | 4.50E-08 | TNFRSF25 | TSS1500 | S_Shore |
| cg16686158 | 4.60E-07 | HORMAD2 | TSS1500 | Island |
| cg16691944 | 9.97E-07 | ZNF445 | TSS1500 | S_Shore |
| cg16692277 | 3.82E-08 | GUCY1B2 | TSS1500 | Open Sea |
| cg16696476 | 9.64E-07 | RANGRF | TSS1500 | N_Shore |
| cg16701167 | 5.58E-09 | ME1 | TSS1500 | S_Shore |
| cg16706260 | 4.53E-08 | REP15 | TSS200 | Open Sea |
| cg16713727 | 8.03E-09 | SLC25A34 | TSS200 | Open Sea |
| cg16714091 | 8.99E-07 | ADAMTSL1 | TSS1500 | Open Sea |
| cg16716189 | 2.93E-07 | FAM19A3 | TSS1500 | Island |
| cg16723488 | 2.45E-07 | APOB | TSS200 | Island |
| cg16725050 | 1.14E-09 | TUBA4B | TSS200 | N_Shore |
| cg16725721 | 5.96E-10 | FSCN3 | 1stExon | Open Sea |
| cg16728604 | 2.23E-07 | KIAA1522 | TSS1500 | N_Shore |
| cg16731240 | 6.69E-07 | ZNF577 | TSS200 | Island |
| cg16732648 | 3.98E-09 | TRPV2 | TSS1500 | Open Sea |
| cg16745104 | 2.54E-07 | MIR941-1 | TSS1500 | N_Shore |
| cg16745596 | 5.38E-08 | SYCN | TSS1500 | S_Shore |
| cg16751451 | 2.02E-07 | EGFR | TSS1500 | N_Shore |
| cg16752583 | 9.96E-09 | TRPV6 | TSS1500 | Open Sea |
| cg16762386 | 5.38E-09 | NR0B2 | 1stExon | Open Sea |
| cg16764848 | 4.61E-07 | GNPNAT1 | TSS1500 | S_Shore |
| cg16779976 | 1.25E-08 | BLNK | 1stExon | Open Sea |
| cg16786458 | 8.84E-07 | PPARGC1B | TSS1500 | N_Shore |
| cg16796959 | 5.55E-07 | PARP10 | TSS1500 | N_Shelf |
| cg16797790 | 5.97E-07 | KRTAP10-12 | 1stExon | Open Sea |
| cg16806210 | 1.38E-07 | TTC36 | 1stExon | N_Shelf |
| cg16839921 | 6.88E-07 | KCNE3 | TSS1500 | S_Shore |
| cg16854606 | 1.36E-08 | DAND5 | 1stExon | Open Sea |
| cg16857801 | 6.73E-08 | LOC440040 | TSS200 | Open Sea |
| cg16859931 | 5.01E-07 | MIR25 | TSS1500 | Open Sea |
| cg16867657 | 2.66E-07 | ELOVL2 | TSS1500 | Island |
| cg16882684 | 6.86E-08 | C9orf173 | TSS200 | N_Shelf |
| cg16887179 | 4.52E-07 | LOC339674 | TSS200 | Open Sea |
| cg16899036 | 9.34E-08 | HOMER3 | TSS1500 | S_Shore |
| cg16911672 | 1.19E-07 | SELENBP1 | TSS1500 | Open Sea |
| cg16928487 | 2.19E-08 | SREBF1 | TSS1500 | N_Shore |
| cg16963138 | 1.18E-10 |  | TSS1500 | S_Shore |
| cg16967583 | 4.69E-07 | AGXT | TSS1500 | Open Sea |
| cg16985632 | 5.46E-08 | FOXS1 | 1stExon | Island |
| cg16987606 | 4.17E-07 | GPRC5C | TSS1500 | N_Shore |
| cg17010657 | 8.73E-07 | PHAX | TSS1500 | N_Shore |
| cg17012513 | 6.90E-07 | ARSF | TSS200 | Open Sea |
| cg17019053 | 3.02E-08 | C2orf65 | TSS200 | Island |
| cg17022727 | 8.85E-08 | MIR25 | TSS1500 | Open Sea |
| cg17024257 | 3.81E-07 | PLD1 | TSS1500 | S_Shore |
| cg17034109 | 2.82E-07 | CYB561D1 | TSS1500 | N_Shore |
| cg17037018 | 8.36E-07 | LOC653566 | TSS1500 | Open Sea |
| cg17038857 | 4.00E-08 | C3orf23 | TSS1500 | N_Shore |
| cg17045844 | 6.78E-08 | C4orf6 | TSS200 | Open Sea |
| cg17047659 | 2.23E-08 | HOXA10 | 1stExon | Island |
| cg17053060 | 4.91E-08 | HEPHL1 | TSS200 | Open Sea |
| cg17073859 | 5.41E-07 | EDNRA | TSS1500 | N_Shore |
| cg17077762 | 2.81E-08 | OAZ2 | TSS1500 | S_Shore |
| cg17095929 | 2.37E-07 | ZNHIT2 | 1stExon | N_Shore |
| cg17096412 | 3.88E-08 | TMEM98 | TSS1500 | N_Shore |
| cg17098103 | 2.50E-08 | HIVEP3 | TSS1500 | S_Shore |
| cg17104151 | 6.16E-07 | C1QC | 1stExon | N_Shelf |
| cg17105014 | 5.83E-07 | GYPC | TSS1500 | N_Shore |
| cg17105755 | 2.19E-09 | C2orf65 | TSS200 | Island |
| cg17113158 | 6.09E-07 | RBL1 | TSS1500 | S_Shore |
| cg17113856 | 5.68E-07 | PPT2 | TSS1500 | N_Shore |
| cg17124224 | 3.96E-09 | BNC1 | TSS1500 | Island |
| cg17126555 | 8.55E-07 | RAVER1 | TSS1500 | Island |
| cg17128799 | 4.79E-08 | PANK4 | TSS1500 | S_Shore |
| cg17132079 | 3.96E-08 | MXRA8 | TSS1500 | S_Shelf |
| cg17168157 | 4.29E-07 | RGS22 | TSS1500 | S_Shore |
| cg17168630 | 8.22E-07 | PLAUR | TSS1500 | S_Shore |
| cg17185710 | 4.91E-07 | MBNL1 | TSS1500 | N_Shore |
| cg17191715 | 5.19E-11 | CA1 | TSS1500 | Open Sea |
| cg17200441 | 5.02E-07 | KRT83 | TSS200 | S_Shore |
| cg17203352 | 9.86E-07 | WFIKKN2 | TSS200 | Open Sea |
| cg17207000 | 1.71E-08 | KIF19 | TSS1500 | N_Shore |
| cg17210938 | 3.05E-07 | TACSTD2 | TSS200 | Island |
| cg17217691 | 3.09E-07 | DDR2 | TSS1500 | Open Sea |
| cg17219660 | 8.48E-07 | GPR37L1 | TSS200 | Open Sea |
| cg17221738 | 7.49E-07 | CLRN1 | 1stExon | Open Sea |
| cg17237813 | 1.90E-08 | TTLL7 | TSS1500 | S_Shore |
| cg17239103 | 8.75E-07 | CCDC19 | TSS200 | Island |
| cg17241776 | 4.16E-07 | DLEU7 | 1stExon | Island |
| cg17243643 | 2.08E-07 | RDH5 | 1stExon | S_Shelf |
| cg17253785 | 2.05E-07 | C8orf44 | TSS1500 | Open Sea |
| cg17260706 | 3.24E-07 | BCL9L | TSS1500 | S_Shore |
| cg17266431 | 3.68E-07 | NADK | TSS1500 | S_Shore |
| cg17272126 | 9.18E-11 | HPX | TSS1500 | Open Sea |
| cg17274742 | 2.87E-07 | GPNMB | 1stExon | N_Shore |
| cg17276021 | 9.33E-07 | FBLIM1 | TSS1500 | N_Shore |
| cg17279103 | 4.03E-08 | MRPS14 | TSS1500 | S_Shore |
| cg17284162 | 3.04E-10 | PYGM | TSS1500 | N_Shelf |
| cg17295720 | 5.66E-07 | LILRB3 | TSS1500 | Open Sea |
| cg17311022 | 1.32E-07 | POPDC2 | TSS1500 | Open Sea |
| cg17329287 | 5.18E-09 | RNF220 | TSS1500 | N_Shore |
| cg17330765 | 2.56E-07 | CNKSR1 | TSS1500 | Open Sea |
| cg17333767 | 2.02E-07 | CECR1 | TSS200 | Open Sea |
| cg17334359 | 2.65E-07 | MAT2B | TSS1500 | N_Shelf |
| cg17339905 | 7.15E-08 | RAB3GAP1 | TSS1500 | Open Sea |
| cg17349406 | 2.58E-07 | ADIG | 1stExon | Open Sea |
| cg17355153 | 2.71E-08 | KIAA1462 | TSS1500 | S_Shore |
| cg17356252 | 4.66E-07 | AIRE | TSS200 | Island |
| cg17359975 | 7.65E-08 | CTGF | TSS1500 | S_Shore |
| cg17367832 | 6.32E-07 | MIR149 | TSS200 | Island |
| cg17372758 | 9.18E-09 | ASCC1 | TSS1500 | S_Shore |
| cg17382841 | 1.56E-07 | EGFLAM | TSS200 | Open Sea |
| cg17395184 | 1.94E-07 | ZFP106 | TSS1500 | Open Sea |
| cg17397420 | 1.71E-07 | MS4A10 | TSS1500 | Open Sea |
| cg17405000 | 2.64E-08 | IL31RA | TSS200 | Open Sea |
| cg17407629 | 9.60E-08 | PAM | TSS1500 | N_Shore |
| cg17408972 | 7.73E-07 | RAI14 | 1stExon | Open Sea |
| cg17412005 | 1.51E-08 | MUTYH | TSS1500 | S_Shore |
| cg17419731 | 4.55E-10 | MIR589 | TSS200 | S_Shore |
| cg17425484 | 5.86E-09 | KCNMB1 | TSS1500 | Open Sea |
| cg17439694 | 7.78E-07 | CP | 1stExon | Open Sea |
| cg17441401 | 3.41E-09 | LRP8 | TSS1500 | S_Shore |
| cg17459635 | 5.37E-08 | ISG20 | TSS1500 | Open Sea |
| cg17470143 | 1.35E-07 | DCLK1 | TSS1500 | S_Shore |
| cg17473673 | 1.44E-07 | PGLYRP2 | TSS1500 | Open Sea |
| cg17482224 | 6.27E-07 | DNAH14 | TSS1500 | N_Shore |
| cg17487170 | 6.45E-08 | TCF7 | TSS200 | Island |
| cg17496788 | 4.06E-07 | DDR2 | TSS1500 | Open Sea |
| cg17496921 | 5.14E-07 | TSPAN16 | 1stExon | S_Shelf |
| cg17497608 | 3.88E-07 | FZD1 | 1stExon | S_Shore |
| cg17501395 | 6.36E-07 | ZC3H12D | TSS200 | Open Sea |
| cg17508063 | 1.41E-08 | KRT8 | 1stExon | S_Shore |
| cg17518965 | 5.18E-08 | S1PR4 | 1stExon | Island |
| cg17519037 | 1.97E-07 | MRPL45 | TSS1500 | N_Shore |
| cg17526462 | 1.77E-07 | WDFY4 | TSS1500 | Open Sea |
| cg17573933 | 6.18E-07 | HOXB2 | TSS1500 | S_Shelf |
| cg17579753 | 7.20E-07 | GAS2L2 | TSS200 | Open Sea |
| cg17588038 | 1.08E-09 | CREB5 | TSS1500 | Open Sea |
| cg17589341 | 2.37E-07 | SLC14A1 | TSS200 | Open Sea |
| cg17594003 | 4.00E-07 | ZEB2 | TSS1500 | N_Shelf |
| cg17594351 | 4.10E-07 | MYF6 | TSS200 | N_Shore |
| cg17602126 | 1.12E-07 | HEYL | TSS1500 | Island |
| cg17623879 | 8.23E-10 | C22orf39 | TSS1500 | S_Shore |
| cg17631429 | 5.47E-08 | FOXS1 | 1stExon | Island |
| cg17632937 | 5.29E-07 | HORMAD2 | TSS200 | Island |
| cg17643662 | 1.42E-07 | MYLK2 | TSS200 | N_Shelf |
| cg17650057 | 8.72E-08 | BIRC7 | TSS200 | N_Shelf |
| cg17667972 | 2.02E-08 | KRT4 | 1stExon | Open Sea |
| cg17670868 | 3.63E-08 | ZNF165 | TSS200 | Open Sea |
| cg17679621 | 9.87E-07 | SLC5A7 | TSS1500 | N_Shore |
| cg17691545 | 1.35E-07 | SLC10A6 | TSS200 | Open Sea |
| cg17697835 | 8.18E-07 | SEPT9 | TSS200 | Open Sea |
| cg17715149 | 8.35E-10 | ZNF702P | TSS1500 | S_Shore |
| cg17726692 | 8.81E-07 | LOC284276 | TSS200 | Open Sea |
| cg17740645 | 6.53E-07 | GRB7 | TSS200 | Open Sea |
| cg17741629 | 1.84E-07 | SETDB2 | TSS1500 | Open Sea |
| cg17744295 | 5.31E-07 | TPM4 | TSS1500 | N_Shore |
| cg17744605 | 7.49E-07 | LOC442245 | TSS200 | Open Sea |
| cg17747879 | 5.39E-07 | PBXIP1 | TSS1500 | Open Sea |
| cg17749384 | 6.39E-10 | MPP7 | TSS200 | Open Sea |
| cg17749509 | 1.35E-07 | SLC16A4 | TSS1500 | Open Sea |
| cg17751430 | 5.04E-12 | PNLIPRP1 | TSS200 | Open Sea |
| cg17759475 | 8.14E-09 | SELENBP1 | TSS1500 | Open Sea |
| cg17791651 | 2.21E-08 | POU3F1 | TSS1500 | Island |
| cg17792740 | 1.72E-07 | AGAP11 | TSS1500 | N_Shore |
| cg17800426 | 1.12E-07 | MYOZ3 | TSS1500 | S_Shelf |
| cg17801352 | 7.12E-09 | PXDN | TSS1500 | S_Shore |
| cg17802464 | 5.21E-07 | SEC14L3 | TSS200 | Open Sea |
| cg17802633 | 3.57E-08 | PROKR1 | TSS1500 | S_Shore |
| cg17807479 | 1.43E-07 | SHISA6 | 1stExon | Island |
| cg17808195 | 4.21E-07 | CLRN1 | 1stExon | Open Sea |
| cg17814047 | 1.47E-08 | PDGFA | TSS1500 | Island |
| cg17819538 | 1.13E-07 | PABPN1 | TSS200 | N_Shore |
| cg17826518 | 1.48E-07 | PDPN | 1stExon | S_Shore |
| cg17826679 | 3.19E-07 | SLC44A2 | TSS200 | Island |
| cg17826834 | 1.20E-09 | NR2F2 | TSS1500 | S_Shelf |
| cg17830682 | 1.03E-07 | LGR6 | TSS1500 | N_Shore |
| cg17836145 | 2.63E-07 | VNN2 | TSS200 | Open Sea |
| cg17836612 | 3.03E-08 | LGALS3BP | TSS1500 | Open Sea |
| cg17839166 | 5.22E-07 | HSPB3 | TSS200 | Open Sea |
| cg17839399 | 1.69E-08 | ETNK1 | TSS1500 | N_Shore |
| cg17866732 | 3.90E-08 | SEPT10 | TSS1500 | Island |
| cg17869161 | 1.16E-07 | GPT | TSS200 | N_Shore |
| cg17880320 | 2.76E-07 | PEX19 | TSS1500 | Open Sea |
| cg17890764 | 8.05E-07 | ITIH4 | TSS200 | Open Sea |
| cg17891011 | 4.39E-08 | FLJ45983 | TSS1500 | Island |
| cg17891968 | 2.30E-12 | BANF2 | TSS1500 | Open Sea |
| cg17897148 | 6.12E-07 | EEFSEC | TSS1500 | N_Shore |
| cg17904988 | 7.10E-07 | NDUFS2 | TSS1500 | N_Shelf |
| cg17915961 | 3.10E-07 | LDB1 | TSS1500 | S_Shore |
| cg17923947 | 4.15E-09 | C14orf138 | TSS1500 | S_Shore |
| cg17928876 | 1.91E-07 | TGFB3 | 1stExon | N_Shore |
| cg17935233 | 1.05E-07 | RHBG | TSS200 | Island |
| cg17939585 | 2.73E-07 | PLXNB2 | TSS1500 | S_Shore |
| cg17941330 | 9.14E-09 | GJA5 | TSS200 | Open Sea |
| cg17952468 | 2.84E-07 | EDN3 | TSS1500 | N_Shore |
| cg17952826 | 3.22E-07 | ACAT2 | TSS1500 | N_Shore |
| cg17953764 | 2.58E-07 | ZAR1 | 1stExon | Island |
| cg17957640 | 1.80E-07 | MYO7A | TSS200 | Open Sea |
| cg17961057 | 6.33E-07 | C2orf81 | TSS1500 | Island |
| cg17963840 | 2.73E-07 | ADRA1A | 1stExon | Island |
| cg17965690 | 2.53E-07 | SLC44A2 | TSS200 | Island |
| cg17972013 | 4.54E-07 | SNORD123 | TSS1500 | S_Shore |
| cg17980364 | 4.99E-10 | TMEM135 | TSS1500 | N_Shore |
| cg18022921 | 3.37E-07 | VEGFA | TSS1500 | N_Shore |
| cg18034295 | 5.17E-07 | C22orf32 | TSS1500 | N_Shore |
| cg18049045 | 1.29E-07 | FCHO2 | TSS1500 | N_Shore |
| cg18050520 | 4.24E-08 | PEBP4 | TSS1500 | Open Sea |
| cg18059933 | 1.34E-07 | TP53INP1 | TSS1500 | S_Shore |
| cg18067859 | 6.65E-09 | SLFN13 | TSS1500 | N_Shore |
| cg18069290 | 6.96E-07 | SLC26A10 | TSS200 | Island |
| cg18072756 | 8.21E-08 | ZNRF4 | TSS200 | N_Shore |
| cg18081258 | 4.81E-07 | NDRG2 | TSS1500 | Island |
| cg18082788 | 8.46E-07 | ZC3H12D | TSS200 | Open Sea |
| cg18083248 | 7.60E-07 | ATP10A | TSS1500 | S_Shore |
| cg18085435 | 1.48E-08 | ATP8B1 | TSS1500 | Open Sea |
| cg18087520 | 6.86E-07 | CALM1 | TSS1500 | N_Shore |
| cg18093120 | 4.43E-09 | MLC1 | 1stExon | Open Sea |
| cg18094404 | 1.82E-07 | RPS15 | TSS1500 | N_Shore |
| cg18097189 | 6.52E-07 | GPR149 | TSS1500 | S_Shore |
| cg18097224 | 1.32E-07 | TCF7 | TSS200 | Island |
| cg18100153 | 1.03E-08 | A2LD1 | TSS1500 | S_Shore |
| cg18113332 | 6.92E-07 | B4GALNT4 | TSS1500 | Open Sea |
| cg18117780 | 6.05E-08 | LOC440461 | TSS1500 | Island |
| cg18121224 | 3.37E-08 | NSD1 | TSS1500 | Island |
| cg18125510 | 4.79E-08 | WARS | 1stExon | N_Shore |
| cg18148488 | 1.57E-07 | SMYD1 | TSS1500 | Open Sea |
| cg18161890 | 1.94E-07 | KRT72 | 1stExon | Island |
| cg18165914 | 7.77E-07 | TMPRSS5 | TSS200 | Open Sea |
| cg18170080 | 2.28E-09 | ESAM | 1stExon | N_Shore |
| cg18177659 | 4.79E-07 | RIOK3 | TSS1500 | N_Shore |
| cg18181954 | 6.29E-09 | MYOCD | TSS1500 | N_Shore |
| cg18184769 | 9.69E-08 | MFN2 | TSS1500 | N_Shore |
| cg18199617 | 5.83E-07 | HMGCS2 | TSS200 | Open Sea |
| cg18204200 | 4.40E-07 | PLEKHH1 | TSS1500 | N_Shore |
| cg18208742 | 1.10E-07 | GFAP | 1stExon | S_Shelf |
| cg18212039 | 5.75E-08 | EXTL1 | TSS1500 | Open Sea |
| cg18219418 | 5.43E-09 | PARP6 | 1stExon | N_Shore |
| cg18224798 | 1.17E-07 | KLHDC1 | TSS1500 | N_Shore |
| cg18230216 | 1.46E-09 | NYX | TSS1500 | Open Sea |
| cg18234296 | 1.44E-07 | C1orf133 | TSS1500 | S_Shore |
| cg18240400 | 1.07E-07 | ANUBL1 | TSS1500 | Island |
| cg18243357 | 1.56E-08 | C3orf51 | TSS200 | Open Sea |
| cg18259504 | 3.32E-07 | ATL1 | 1stExon | S_Shore |
| cg18261909 | 4.48E-07 | VARS2 | TSS1500 | Island |
| cg18274788 | 3.77E-08 | MIR320B1 | TSS1500 | S_Shelf |
| cg18286285 | 2.56E-07 | LDB3 | TSS200 | Open Sea |
| cg18286850 | 5.43E-07 | TF | TSS1500 | N_Shore |
| cg18291437 | 6.47E-07 | FAM70A | TSS1500 | S_Shore |
| cg18298494 | 6.13E-08 | TGFB3 | 1stExon | N_Shore |
| cg18313790 | 1.67E-07 | PCDHB13 | 1stExon | Island |
| cg18314765 | 6.44E-08 | TNP1 | 1stExon | Open Sea |
| cg18322510 | 9.90E-07 | LGALS8 | TSS200 | N_Shore |
| cg18328933 | 1.49E-07 | ABHD14B | 1stExon | N_Shore |
| cg18343556 | 4.73E-07 | C3orf45 | TSS200 | S_Shore |
| cg18344063 | 3.47E-07 | MGAT4C | 1stExon | Open Sea |
| cg18344745 | 4.53E-08 | GLRB | TSS1500 | N_Shore |
| cg18347642 | 9.21E-07 | KCNIP4 | TSS1500 | Island |
| cg18349298 | 1.23E-09 | RARRES1 | TSS1500 | Island |
| cg18357371 | 5.01E-07 | SLC17A3 | TSS200 | Open Sea |
| cg18363417 | 1.86E-09 | MIR573 | TSS200 | Open Sea |
| cg18367578 | 5.90E-07 | NNMT | TSS200 | Open Sea |
| cg18378811 | 1.84E-08 | ARSJ | 1stExon | N_Shore |
| cg18379537 | 2.22E-08 | EPHX4 | TSS1500 | N_Shore |
| cg18385440 | 8.41E-07 | APBA2 | TSS1500 | Open Sea |
| cg18395636 | 5.31E-08 | RAB38 | TSS200 | S_Shore |
| cg18396533 | 3.30E-09 | DYDC2 | TSS1500 | Island |
| cg18404374 | 5.85E-07 | LRRC3B | TSS1500 | N_Shore |
| cg18404652 | 9.04E-08 | AP3M2 | TSS1500 | N_Shore |
| cg18413025 | 2.06E-07 | PALLD | TSS200 | Open Sea |
| cg18418457 | 4.48E-08 | TGFBI | TSS1500 | N_Shore |
| cg18422654 | 1.50E-10 | MYH7 | TSS1500 | Open Sea |
| cg18433146 | 1.82E-10 | CD36 | 1stExon | Open Sea |
| cg18444689 | 1.10E-07 | MAP3K13 | TSS1500 | Open Sea |
| cg18448949 | 2.26E-07 | HOXD8 | TSS1500 | Island |
| cg18449062 | 8.79E-08 | CNNM2 | 1stExon | S_Shore |
| cg18449120 | 2.92E-07 | SOX11 | 1stExon | Island |
| cg18450832 | 7.41E-07 | C5orf42 | TSS1500 | S_Shore |
| cg18450931 | 9.88E-07 | PLCH1 | TSS1500 | Open Sea |
| cg18454133 | 1.26E-08 | EFS | TSS1500 | Island |
| cg18457613 | 2.78E-09 | LHFPL2 | TSS1500 | S_Shore |
| cg18484189 | 6.38E-08 | NLRP10 | 1stExon | Open Sea |
| cg18497587 | 1.10E-07 | FBN3 | 1stExon | N_Shore |
| cg18499148 | 2.36E-08 | OR51F2 | TSS1500 | Open Sea |
| cg18504989 | 1.36E-07 | DMPK | TSS1500 | N_Shelf |
| cg18514949 | 3.30E-07 | PPP2CA | TSS1500 | S_Shore |
| cg18525582 | 1.25E-09 | TNS1 | TSS1500 | Open Sea |
| cg18530324 | 9.92E-09 | KIAA0427 | TSS1500 | N_Shore |
| cg18534491 | 2.55E-07 | REC8 | TSS200 | Island |
| cg18535283 | 1.58E-07 | CYB5R3 | TSS1500 | N_Shore |
| cg18537730 | 7.64E-07 | IZUMO1 | 1stExon | N_Shelf |
| cg18544365 | 2.12E-07 | MIR199A1 | TSS200 | Open Sea |
| cg18546006 | 8.89E-07 | TRIM13 | TSS1500 | N_Shore |
| cg18546437 | 8.90E-07 | CSF2RB | TSS1500 | Open Sea |
| cg18546840 | 5.41E-07 | FOXP2 | 1stExon | Open Sea |
| cg18555289 | 3.07E-09 | GPT | TSS1500 | N_Shore |
| cg18556834 | 4.61E-08 | REC8 | TSS200 | Island |
| cg18565264 | 5.98E-08 | C1orf86 | TSS1500 | N_Shelf |
| cg18567504 | 3.88E-08 | VTI1B | TSS1500 | S_Shore |
| cg18569182 | 8.45E-07 | TRAIP | TSS1500 | S_Shore |
| cg18572214 | 8.05E-11 | ASB2 | 1stExon | Open Sea |
| cg18585693 | 7.09E-07 | DSCAML1 | TSS200 | S_Shore |
| cg18596369 | 3.38E-07 | NRTN | TSS1500 | Island |
| cg18599081 | 1.29E-07 | CCRL2 | TSS1500 | Open Sea |
| cg18607308 | 9.72E-08 | FAM96B | TSS1500 | Island |
| cg18628371 | 3.05E-07 | REC8 | TSS200 | Island |
| cg18630644 | 8.34E-07 | C6orf201 | 1stExon | S_Shore |
| cg18637232 | 3.02E-08 | ADAMTSL3 | TSS1500 | N_Shore |
| cg18655384 | 2.24E-07 | FLJ43390 | TSS1500 | Island |
| cg18674899 | 5.11E-07 | PGM3 | TSS1500 | S_Shore |
| cg18689454 | 5.33E-07 | AIRE | TSS200 | Island |
| cg18691434 | 8.93E-07 | STAG3 | TSS200 | Island |
| cg18704047 | 2.57E-07 | PART1 | TSS200 | Open Sea |
| cg18709349 | 3.74E-07 | PNMAL2 | TSS200 | Island |
| cg18714204 | 4.58E-07 | DDX46 | TSS1500 | N_Shore |
| cg18715793 | 4.18E-10 | PLIN4 | TSS1500 | Open Sea |
| cg18719665 | 9.40E-10 | TTC39C | TSS200 | Open Sea |
| cg18722124 | 2.60E-07 | ZNF536 | TSS1500 | N_Shelf |
| cg18727936 | 8.63E-07 | VENTX | TSS1500 | Island |
| cg18734330 | 5.65E-07 | C14orf139 | TSS200 | Open Sea |
| cg18738581 | 1.69E-07 | IL17RE | TSS200 | Open Sea |
| cg18741586 | 5.66E-07 | MUSTN1 | 1stExon | Open Sea |
| cg18743287 | 1.77E-07 | RDH13 | TSS1500 | S_Shore |
| cg18745416 | 4.44E-07 | ESR1 | TSS1500 | Open Sea |
| cg18752854 | 2.47E-08 | TNS1 | TSS1500 | Open Sea |
| cg18752880 | 9.71E-07 | C1QTNF3 | TSS200 | Open Sea |
| cg18757087 | 1.06E-08 | CPEB4 | 1stExon | S_Shore |
| cg18764771 | 1.59E-07 | FRK | TSS200 | Open Sea |
| cg18773937 | 3.66E-07 | IL1B | TSS1500 | Open Sea |
| cg18789547 | 9.08E-07 | CCK | TSS1500 | S_Shore |
| cg18799069 | 6.27E-07 | LYPLA1 | TSS1500 | S_Shore |
| cg18805892 | 7.01E-08 | LY9 | TSS1500 | Open Sea |
| cg18816996 | 9.33E-08 | COL23A1 | TSS1500 | S_Shore |
| cg18818135 | 7.56E-08 | C12orf40 | TSS1500 | Open Sea |
| cg18827787 | 1.80E-08 | C18orf20 | TSS1500 | Open Sea |
| cg18830527 | 1.55E-09 | CD8A | TSS200 | S_Shore |
| cg18850728 | 6.48E-08 | MRO | TSS1500 | Open Sea |
| cg18856296 | 2.35E-07 | CCDC86 | TSS200 | N_Shore |
| cg18862215 | 9.15E-08 | SCGBL | TSS200 | Open Sea |
| cg18862562 | 3.12E-07 | CASQ1 | 1stExon | Open Sea |
| cg18865207 | 3.82E-07 | LY9 | TSS200 | Open Sea |
| cg18867010 | 3.54E-08 | OR6A2 | 1stExon | Open Sea |
| cg18871253 | 2.48E-07 | FOXP2 | 1stExon | Open Sea |
| cg18872321 | 5.15E-07 | GATA5 | TSS1500 | Island |
| cg18877514 | 5.12E-07 | PXMP2 | TSS1500 | N_Shore |
| cg18879300 | 6.77E-08 | PADI2 | TSS1500 | S_Shore |
| cg18899064 | 5.07E-10 | PLAT | TSS1500 | Open Sea |
| cg18920397 | 5.49E-07 | LY9 | TSS200 | Open Sea |
| cg18923051 | 1.08E-07 | MEX3A | TSS1500 | S_Shore |
| cg18935353 | 4.89E-07 | CD248 | TSS1500 | S_Shore |
| cg18944099 | 7.69E-09 | C6orf47 | TSS1500 | S_Shore |
| cg18951718 | 2.13E-07 | LPAR5 | TSS200 | Open Sea |
| cg18972123 | 9.10E-07 | MBL2 | TSS1500 | Open Sea |
| cg18990667 | 1.26E-09 | MEG8 | TSS1500 | Open Sea |
| cg19004285 | 5.70E-12 | NR2C1 | TSS1500 | S_Shore |
| cg19008088 | 4.35E-09 | TLX1 | TSS1500 | N_Shore |
| cg19009305 | 2.12E-07 | ISG20 | TSS1500 | Open Sea |
| cg19013391 | 2.86E-08 | ADCY5 | 1stExon | Island |
| cg19014419 | 1.79E-07 | ZNF300 | TSS200 | Island |
| cg19015961 | 1.69E-07 | ACTA2 | TSS200 | Open Sea |
| cg19023847 | 2.77E-07 | DHDPSL | 1stExon | Open Sea |
| cg19030737 | 1.57E-08 | ITPKB | TSS1500 | S_Shore |
| cg19038230 | 4.17E-07 | LRCH1 | TSS1500 | N_Shore |
| cg19047340 | 5.25E-07 | EIF6 | TSS1500 | S_Shore |
| cg19064523 | 2.10E-07 | TWIST1 | TSS1500 | S_Shore |
| cg19066520 | 6.42E-11 | CCDC50 | TSS1500 | N_Shore |
| cg19071879 | 3.79E-07 | AFF3 | TSS200 | S_Shore |
| cg19074493 | 6.62E-07 | SMYD1 | TSS1500 | Open Sea |
| cg19074496 | 5.65E-08 | TUBA4B | TSS200 | N_Shore |
| cg19083007 | 1.97E-07 | RCAN2 | TSS1500 | Open Sea |
| cg19091930 | 7.18E-10 | LRRC14B | TSS1500 | N_Shore |
| cg19096967 | 7.87E-07 | CNIH2 | TSS1500 | N_Shore |
| cg19097280 | 7.25E-07 | LRRN4 | TSS1500 | S_Shelf |
| cg19102412 | 6.26E-07 | LOC84931 | TSS1500 | Open Sea |
| cg19103609 | 3.82E-07 | PKN1 | TSS1500 | N_Shore |
| cg19108747 | 1.67E-07 | FAM124B | 1stExon | Open Sea |
| cg19114310 | 5.44E-07 | GDF1 | TSS1500 | Island |
| cg19145398 | 5.77E-08 | FOXS1 | TSS1500 | S_Shore |
| cg19145607 | 3.20E-07 | CXCR6 | TSS1500 | Open Sea |
| cg19146902 | 2.01E-08 | CUGBP1 | TSS1500 | Open Sea |
| cg19149020 | 1.14E-08 | MEIG1 | TSS1500 | N_Shore |
| cg19154600 | 1.87E-08 | SYNPO2L | TSS200 | Open Sea |
| cg19155932 | 7.10E-08 | C20orf85 | TSS200 | Island |
| cg19159092 | 1.07E-08 | FLJ39609 | TSS1500 | S_Shore |
| cg19172487 | 1.91E-09 | PARVB | TSS1500 | S_Shelf |
| cg19188306 | 1.48E-08 | LAYN | TSS1500 | Island |
| cg19194233 | 1.52E-08 | MLC1 | 1stExon | Open Sea |
| cg19202014 | 4.45E-08 | SIGIRR | TSS200 | Island |
| cg19214331 | 2.46E-08 | MYL9 | TSS1500 | Island |
| cg19216211 | 4.02E-07 | C8orf58 | TSS1500 | N_Shore |
| cg19216731 | 4.50E-08 | CSRP3 | TSS200 | Open Sea |
| cg19221489 | 5.18E-09 | SPATA2 | TSS1500 | N_Shore |
| cg19222574 | 3.42E-07 | PLXNA1 | TSS1500 | N_Shore |
| cg19233001 | 1.41E-07 | TNNT2 | TSS200 | Open Sea |
| cg19238824 | 2.49E-07 | FAM180A | TSS200 | Open Sea |
| cg19240569 | 5.26E-07 | HRASLS2 | TSS1500 | Open Sea |
| cg19240857 | 1.21E-08 | LY6G6E | TSS1500 | Open Sea |
| cg19242688 | 1.22E-07 | GNG8 | TSS1500 | Island |
| cg19245773 | 3.01E-08 | ANTXRL | TSS1500 | S_Shore |
| cg19255053 | 6.09E-08 | FAM129A | TSS1500 | S_Shore |
| cg19257102 | 3.91E-07 | LOXL1 | TSS1500 | N_Shore |
| cg19257550 | 1.17E-07 | CA9 | TSS200 | N_Shore |
| cg19264571 | 9.98E-08 | APCDD1 | TSS1500 | Island |
| cg19275745 | 9.28E-08 | GRK7 | TSS1500 | N_Shore |
| cg19279346 | 2.47E-07 | LILRB2 | TSS1500 | Open Sea |
| cg19284039 | 4.48E-08 | ASCL2 | TSS1500 | Island |
| cg19286437 | 4.08E-07 | LDB3 | TSS1500 | Open Sea |
| cg19286687 | 2.63E-07 | DES | TSS1500 | N_Shore |
| cg19287817 | 8.80E-07 | BRD4 | TSS200 | Open Sea |
| cg19303187 | 3.59E-07 | DLX2 | TSS1500 | S_Shore |
| cg19311153 | 4.55E-08 | MARVELD3 | TSS1500 | Island |
| cg19314866 | 3.06E-07 | CAPN2 | TSS1500 | Open Sea |
| cg19317715 | 3.30E-08 | AOC2 | TSS200 | Open Sea |
| cg19324627 | 1.17E-07 | APOA1 | 1stExon | S_Shore |
| cg19343034 | 4.77E-07 | MAMSTR | TSS1500 | Island |
| cg19344626 | 3.39E-08 | NWD1 | TSS200 | Open Sea |
| cg19352605 | 2.62E-07 | GPT | TSS1500 | N_Shore |
| cg19356389 | 2.21E-07 | RING1 | TSS1500 | N_Shore |
| cg19362196 | 4.43E-08 | STMN1 | TSS1500 | Island |
| cg19371795 | 4.88E-07 | INSR | TSS1500 | Island |
| cg19380722 | 1.27E-07 | PKD2L1 | TSS200 | Open Sea |
| cg19392831 | 8.20E-08 | PRLHR | TSS1500 | Island |
| cg19399165 | 2.33E-07 | EMILIN1 | TSS200 | Open Sea |
| cg19403534 | 3.51E-07 | PRLHR | TSS1500 | Island |
| cg19405883 | 4.20E-07 | RAI14 | TSS200 | Open Sea |
| cg19406367 | 1.10E-07 | SGIP1 | 1stExon | S_Shore |
| cg19418958 | 2.38E-10 | TEX15 | 1stExon | Open Sea |
| cg19422019 | 3.39E-08 | C21orf34 | TSS200 | Open Sea |
| cg19432362 | 8.14E-08 | SIAH3 | TSS1500 | Open Sea |
| cg19432993 | 1.32E-10 | HOXA2 | TSS1500 | Island |
| cg19433021 | 3.03E-07 | NFYB | TSS1500 | S_Shore |
| cg19433435 | 6.33E-07 | FEZ1 | TSS1500 | S_Shore |
| cg19442470 | 4.84E-08 | CLU | TSS1500 | N_Shore |
| cg19458020 | 5.46E-07 | RARA | TSS1500 | Island |
| cg19460817 | 9.54E-07 | CUGBP1 | TSS1500 | S_Shore |
| cg19463256 | 4.36E-08 | CCDC8 | 1stExon | Island |
| cg19473653 | 5.01E-07 | RB1 | TSS1500 | N_Shore |
| cg19474517 | 3.92E-07 | BEX2 | TSS1500 | S_Shore |
| cg19476647 | 2.95E-07 | LTBR | TSS1500 | Island |
| cg19485804 | 2.11E-11 | NGEF | TSS1500 | Open Sea |
| cg19502936 | 5.02E-07 | RILP | TSS200 | S_Shore |
| cg19503826 | 7.48E-09 | EPN1 | TSS200 | S_Shore |
| cg19513903 | 1.11E-07 | C14orf167 | TSS1500 | S_Shore |
| cg19515035 | 9.30E-07 | RTKN | TSS1500 | S_Shore |
| cg19530885 | 5.36E-07 | SCGB3A2 | TSS200 | Open Sea |
| cg19532939 | 1.57E-08 | ART4 | TSS200 | Open Sea |
| cg19534753 | 1.54E-07 | MYLK | TSS1500 | Open Sea |
| cg19536559 | 4.06E-07 | VWC2L | TSS200 | Open Sea |
| cg19544434 | 8.70E-07 | PTPRE | TSS200 | Open Sea |
| cg19547929 | 3.05E-07 | NOTCH3 | TSS1500 | S_Shore |
| cg19562321 | 3.52E-07 | SLC36A2 | 1stExon | Open Sea |
| cg19567594 | 1.34E-08 | MRVI1 | 1stExon | Open Sea |
| cg19567891 | 3.71E-08 | LOC254559 | TSS200 | Island |
| cg19579782 | 7.02E-08 | TRDMT1 | TSS200 | S_Shore |
| cg19582647 | 7.31E-07 | MIR942 | TSS200 | Open Sea |
| cg19583655 | 3.18E-07 | PCYT2 | TSS1500 | Island |
| cg19584871 | 1.71E-07 | NTSR2 | TSS200 | S_Shore |
| cg19585100 | 8.53E-07 | PAPLN | TSS1500 | N_Shore |
| cg19592945 | 4.07E-07 | P2RX6 | 1stExon | S_Shore |
| cg19594666 | 5.25E-08 | LEP | TSS200 | Island |
| cg19599045 | 1.23E-07 | SLC7A7 | TSS1500 | N_Shore |
| cg19599666 | 2.16E-07 | LPP | TSS1500 | N_Shore |
| cg19619331 | 1.79E-09 | FAM71F2 | TSS1500 | Open Sea |
| cg19627213 | 7.79E-07 | SLC22A2 | TSS200 | S_Shore |
| cg19628299 | 1.03E-07 | SMOC2 | TSS1500 | N_Shore |
| cg19628333 | 4.22E-07 | RBMS2 | TSS1500 | Open Sea |
| cg19628553 | 2.16E-08 | TMEM9 | TSS1500 | S_Shore |
| cg19640007 | 1.46E-07 | C4orf51 | TSS1500 | Open Sea |
| cg19643053 | 2.58E-07 | HOXA5 | TSS1500 | Island |
| cg19645616 | 8.32E-07 | RAVER1 | TSS1500 | Island |
| cg19654743 | 2.43E-07 | SEPT9 | TSS200 | N_Shore |
| cg19658046 | 8.33E-07 | FAM193B | TSS1500 | S_Shore |
| cg19671120 | 7.27E-07 | CNGA3 | 1stExon | Island |
| cg19672873 | 8.59E-08 | FAM177B | TSS200 | Open Sea |
| cg19678538 | 1.36E-07 | PCDHB14 | 1stExon | Island |
| cg19684083 | 4.24E-07 | LNX1 | TSS200 | Open Sea |
| cg19686637 | 4.68E-07 | SLC28A1 | TSS200 | Open Sea |
| cg19689368 | 3.99E-07 | FAM180A | TSS200 | Open Sea |
| cg19700094 | 1.59E-07 | DRAP1 | TSS1500 | Island |
| cg19701264 | 3.48E-07 | FBN3 | TSS200 | N_Shore |
| cg19702271 | 6.02E-08 | PLA2G7 | TSS1500 | S_Shore |
| cg19706795 | 9.72E-08 | DFNA5 | TSS1500 | S_Shore |
| cg19716018 | 6.38E-08 | RPS15 | TSS1500 | N_Shore |
| cg19719807 | 7.31E-08 | CD164L2 | 1stExon | Island |
| cg19731612 | 2.77E-07 | NSD1 | TSS1500 | Island |
| cg19747604 | 3.55E-07 | CCDC8 | 1stExon | Island |
| cg19749975 | 5.63E-08 | ZRANB2 | TSS1500 | S_Shore |
| cg19759064 | 7.61E-07 | PHKG1 | 1stExon | Open Sea |
| cg19759481 | 2.70E-07 | HOXA5 | TSS200 | Island |
| cg19759788 | 6.74E-07 | FBLIM1 | TSS1500 | N_Shore |
| cg19764047 | 7.84E-10 | CDH18 | TSS1500 | Open Sea |
| cg19764555 | 4.33E-07 | AHNAK | TSS1500 | S_Shore |
| cg19770906 | 1.99E-08 | UCP3 | TSS1500 | Open Sea |
| cg19771781 | 8.04E-09 | RAPSN | TSS200 | Open Sea |
| cg19784428 | 3.63E-09 | NWD1 | TSS200 | Open Sea |
| cg19789505 | 6.00E-07 | OBSCN | TSS1500 | Open Sea |
| cg19802138 | 2.63E-07 | SOX1 | 1stExon | Island |
| cg19805160 | 9.14E-07 | CCDC19 | TSS1500 | S_Shore |
| cg19809039 | 2.74E-07 | C11orf70 | 1stExon | S_Shore |
| cg19815803 | 4.44E-07 | SIPA1L1 | TSS1500 | Open Sea |
| cg19816290 | 3.96E-07 |  | TSS1500 | Island |
| cg19822229 | 1.23E-07 | TRAPPC3 | TSS1500 | S_Shore |
| cg19831197 | 8.15E-07 | NEIL1 | TSS200 | N_Shore |
| cg19833103 | 2.45E-08 | LRCH1 | TSS1500 | N_Shore |
| cg19836423 | 1.03E-07 | MS4A15 | TSS1500 | Open Sea |
| cg19838963 | 1.42E-07 | LOC100126784 | TSS1500 | S_Shelf |
| cg19844653 | 3.25E-08 | TCF7 | TSS200 | S_Shore |
| cg19847577 | 8.57E-07 | APBA2 | TSS200 | Open Sea |
| cg19850333 | 7.19E-08 | CCRL2 | TSS1500 | Open Sea |
| cg19856273 | 3.79E-07 | C1orf190 | TSS1500 | N_Shore |
| cg19862427 | 1.50E-09 | MYL2 | TSS200 | Open Sea |
| cg19863405 | 5.68E-07 | ACTL7B | TSS1500 | S_Shore |
| cg19864808 | 3.35E-07 | STK19 | TSS200 | N_Shore |
| cg19866195 | 3.06E-07 | CCNA1 | TSS1500 | N_Shore |
| cg19868730 | 8.70E-09 | POPDC2 | TSS1500 | Open Sea |
| cg19871235 | 1.57E-07 | TSPAN8 | TSS1500 | Open Sea |
| cg19876649 | 9.92E-07 | MYOM1 | TSS200 | Open Sea |
| cg19877103 | 2.78E-08 | LYNX1 | TSS200 | Open Sea |
| cg19878627 | 5.57E-07 | LOC100302652 | TSS200 | Island |
| cg19895047 | 2.36E-07 | CSGALNACT1 | TSS200 | S_Shore |
| cg19898258 | 5.49E-07 | GRASP | TSS1500 | N_Shore |
| cg19903805 | 9.90E-09 | TC2N | 1stExon | Open Sea |
| cg19904425 | 3.24E-07 | SERPINA12 | TSS1500 | Open Sea |
| cg19907725 | 2.28E-07 | S100A2 | TSS1500 | Open Sea |
| cg19908768 | 9.73E-07 | SULT1C4 | TSS200 | Open Sea |
| cg19921377 | 4.18E-07 | ZNF358 | TSS1500 | N_Shore |
| cg19952821 | 2.48E-08 | SERPINB11 | TSS1500 | Open Sea |
| cg19953843 | 2.44E-07 | CCDC117 | TSS1500 | N_Shore |
| cg19954017 | 5.93E-07 | UTS2R | 1stExon | Island |
| cg19958750 | 6.02E-08 | SH2D6 | TSS1500 | N_Shelf |
| cg19964641 | 8.59E-08 | SEC14L4 | TSS200 | S_Shore |
| cg19969694 | 4.04E-07 | VPS18 | TSS1500 | N_Shore |
| cg19975916 | 7.00E-07 | GPR81 | TSS200 | Open Sea |
| cg19977179 | 5.29E-07 | GP9 | TSS200 | Open Sea |
| cg19981409 | 3.05E-07 | NOX4 | TSS1500 | S_Shore |
| cg19985421 | 4.05E-08 | NDUFC2 | TSS200 | S_Shore |
| cg19986126 | 8.57E-08 | MIR1257 | TSS1500 | Open Sea |
| cg19987965 | 8.96E-07 | TULP4 | 1stExon | Open Sea |
| cg19989295 | 2.10E-07 | REC8 | TSS200 | Island |
| cg20003983 | 3.45E-07 | C20orf85 | TSS1500 | N_Shore |
| cg20004424 | 5.85E-07 | TBL2 | TSS1500 | S_Shore |
| cg20006729 | 6.87E-09 | ATN1 | TSS1500 | N_Shelf |
| cg20014063 | 4.29E-08 | HSPA2 | TSS1500 | N_Shore |
| cg20016914 | 4.68E-07 | C14orf181 | TSS200 | S_Shore |
| cg20019546 | 2.42E-07 | SFRP4 | 1stExon | Island |
| cg20023324 | 9.21E-08 | LRRC37B | TSS200 | Open Sea |
| cg20036162 | 5.40E-11 | C8orf46 | TSS1500 | Open Sea |
| cg20039814 | 7.49E-07 | TP53INP1 | TSS1500 | S_Shore |
| cg20050108 | 1.77E-07 | FOXP2 | TSS1500 | Open Sea |
| cg20050484 | 5.58E-07 | MYLK | 1stExon | Open Sea |
| cg20050826 | 8.40E-08 | KRT72 | 1stExon | Island |
| cg20054248 | 2.45E-08 | IKZF4 | TSS200 | Open Sea |
| cg20066612 | 9.63E-07 | KCNJ9 | TSS1500 | N_Shelf |
| cg20067142 | 1.33E-07 | ACOT2 | TSS1500 | N_Shore |
| cg20083676 | 3.35E-08 | C9orf47 | 1stExon | Island |
| cg20090497 | 7.90E-12 | TAS2R9 | 1stExon | Open Sea |
| cg20092036 | 7.86E-07 | UBE3B | TSS1500 | N_Shore |
| cg20103124 | 1.96E-07 | ALOX5AP | TSS200 | Open Sea |
| cg20107987 | 3.97E-09 | FAM111B | TSS1500 | N_Shore |
| cg20110535 | 4.89E-08 | PRKACA | TSS1500 | N_Shelf |
| cg20114394 | 4.10E-08 | FSTL1 | TSS1500 | Island |
| cg20116804 | 1.77E-07 | C6orf138 | 1stExon | Island |
| cg20116935 | 1.69E-08 | SEMA3B | TSS1500 | Open Sea |
| cg20124223 | 1.20E-07 | DIXDC1 | TSS1500 | N_Shore |
| cg20132791 | 7.08E-08 | SLC26A8 | TSS1500 | N_Shelf |
| cg20141229 | 8.90E-08 | CLDN22 | TSS200 | Open Sea |
| cg20170271 | 8.98E-07 | SH3BP1 | TSS1500 | N_Shore |
| cg20170989 | 6.03E-07 | POM121L12 | TSS1500 | N_Shore |
| cg20176648 | 1.80E-07 | AQP1 | TSS1500 | Open Sea |
| cg20177385 | 9.25E-07 | SLC41A3 | TSS200 | Open Sea |
| cg20184238 | 4.89E-07 | C1QL1 | TSS1500 | N_Shore |
| cg20203041 | 1.04E-07 | DUSP3 | TSS1500 | S_Shore |
| cg20206204 | 3.52E-08 | MIR614 | TSS1500 | Open Sea |
| cg20208990 | 5.16E-07 | PMEPA1 | TSS1500 | S_Shore |
| cg20226764 | 3.71E-07 | MLN | TSS200 | Open Sea |
| cg20236750 | 3.55E-07 | LGI1 | TSS1500 | Open Sea |
| cg20237610 | 2.15E-09 | LAMA2 | TSS1500 | Open Sea |
| cg20238308 | 5.94E-08 | PTPRQ | TSS1500 | Open Sea |
| cg20248204 | 5.57E-07 | KCTD12 | TSS1500 | S_Shore |
| cg20248822 | 7.00E-07 | C1orf151 | TSS1500 | N_Shore |
| cg20249277 | 3.67E-08 | C7orf53 | TSS200 | Open Sea |
| cg20249566 | 5.13E-08 | NWD1 | TSS200 | Open Sea |
| cg20250935 | 1.35E-07 | CALM1 | TSS1500 | N_Shore |
| cg20254361 | 9.09E-08 | MYOM3 | TSS200 | Open Sea |
| cg20266715 | 1.69E-07 | CAPN2 | TSS1500 | Open Sea |
| cg20269537 | 2.99E-07 | ATXN10 | TSS1500 | N_Shore |
| cg20288341 | 3.99E-07 | LY6G6E | TSS1500 | Open Sea |
| cg20289911 | 2.35E-07 | FAM110A | TSS1500 | S_Shore |
| cg20303399 | 2.05E-08 | ADRA1A | TSS200 | Island |
| cg20317748 | 5.30E-07 | C15orf52 | 1stExon | Open Sea |
| cg20318845 | 4.59E-07 | SIX2 | TSS1500 | Island |
| cg20322886 | 7.22E-08 | C14orf180 | TSS200 | Open Sea |
| cg20333727 | 6.56E-08 | LSP1 | TSS200 | N_Shelf |
| cg20335213 | 2.22E-07 | ARHGAP29 | TSS1500 | S_Shore |
| cg20337103 | 4.20E-07 | BCL9L | TSS200 | S_Shore |
| cg20352728 | 2.14E-07 | LRP5L | TSS1500 | S_Shelf |
| cg20356482 | 2.28E-07 | FBP2 | TSS200 | Open Sea |
| cg20359360 | 2.65E-07 | PNPLA2 | TSS1500 | N_Shore |
| cg20363347 | 6.52E-07 | MYEOV | TSS200 | Open Sea |
| cg20366832 | 3.62E-07 | LLGL2 | TSS1500 | N_Shore |
| cg20370281 | 8.97E-07 | VPS18 | TSS1500 | N_Shore |
| cg20377447 | 8.38E-11 | TSPO2 | TSS1500 | Open Sea |
| cg20380214 | 6.86E-07 | LOC144571 | TSS200 | Island |
| cg20389981 | 3.37E-07 | CCDC136 | 1stExon | S_Shore |
| cg20398120 | 5.78E-08 | PRKAG3 | TSS200 | Open Sea |
| cg20405384 | 6.95E-07 | TCF23 | TSS200 | N_Shore |
| cg20417424 | 4.97E-09 | ST6GALNAC5 | TSS1500 | N_Shore |
| cg20427865 | 1.53E-07 | CX3CL1 | TSS200 | Open Sea |
| cg20431135 | 1.68E-07 | MFAP4 | TSS1500 | Open Sea |
| cg20442078 | 6.36E-07 | UCN | TSS1500 | Island |
| cg20443501 | 1.34E-07 | PLAGL1 | TSS1500 | S_Shore |
| cg20451680 | 2.67E-07 | ESM1 | 1stExon | Open Sea |
| cg20459022 | 7.44E-08 | IGFL2 | 1stExon | Open Sea |
| cg20471017 | 8.00E-07 | FOLR4 | TSS1500 | Open Sea |
| cg20480642 | 4.93E-07 | PXDNL | TSS200 | Open Sea |
| cg20481340 | 5.13E-07 | GPR20 | TSS1500 | Open Sea |
| cg20491963 | 3.50E-08 | RPL31P11 | TSS200 | Open Sea |
| cg20492912 | 8.26E-08 | UBE4A | TSS1500 | N_Shore |
| cg20494635 | 5.50E-08 | LOC100133545 | TSS1500 | N_Shore |
| cg20494779 | 8.64E-08 | MC2R | TSS1500 | Open Sea |
| cg20511832 | 3.09E-08 | CHRNB4 | TSS1500 | S_Shore |
| cg20516209 | 1.48E-07 | EMILIN1 | 1stExon | N_Shelf |
| cg20521035 | 3.41E-08 | MID1 | TSS200 | Open Sea |
| cg20522675 | 5.18E-08 | TPRG1L | TSS1500 | N_Shore |
| cg20523169 | 3.43E-07 | FLJ45079 | TSS200 | N_Shelf |
| cg20525917 | 8.55E-07 | GATA5 | TSS1500 | S_Shore |
| cg20530056 | 9.04E-08 | IKBKE | TSS1500 | Open Sea |
| cg20555778 | 6.39E-10 | MIR2278 | TSS200 | Open Sea |
| cg20566840 | 4.59E-07 | GPR81 | TSS200 | Open Sea |
| cg20569108 | 9.58E-07 | MLLT10 | TSS1500 | N_Shore |
| cg20582800 | 6.89E-07 | RSPH9 | TSS200 | Island |
| cg20596724 | 1.40E-10 | SDR9C7 | TSS1500 | Open Sea |
| cg20610181 | 5.04E-09 | CA9 | 1stExon | N_Shore |
| cg20629183 | 1.05E-07 | MPP2 | TSS1500 | S_Shore |
| cg20630655 | 7.90E-10 | SNUPN | TSS1500 | S_Shore |
| cg20632224 | 4.62E-08 | MIR320B1 | TSS200 | S_Shelf |
| cg20632887 | 2.03E-07 | ATP1A2 | 1stExon | Open Sea |
| cg20638429 | 1.36E-07 | GGT1 | TSS200 | Open Sea |
| cg20640261 | 6.42E-08 | MSH5 | TSS1500 | N_Shore |
| cg20649716 | 7.30E-08 | PCDHB10 | 1stExon | Island |
| cg20655350 | 5.82E-07 | GPBAR1 | TSS1500 | Open Sea |
| cg20663448 | 5.08E-08 | FILIP1 | TSS1500 | Open Sea |
| cg20669834 | 2.54E-07 | MYLK | 1stExon | Open Sea |
| cg20673840 | 7.46E-10 | ESM1 | 1stExon | Open Sea |
| cg20677436 | 7.01E-07 | SLC26A10 | TSS1500 | Island |
| cg20693209 | 1.06E-07 | SPON1 | 1stExon | Island |
| cg20695297 | 7.65E-07 | S1PR4 | 1stExon | Island |
| cg20700549 | 7.74E-08 | TMEM109 | TSS1500 | N_Shore |
| cg20703614 | 5.01E-07 | YDJC | TSS1500 | S_Shore |
| cg20705812 | 9.63E-07 | DLGAP2 | TSS200 | Open Sea |
| cg20713761 | 1.60E-08 | HVCN1 | TSS1500 | S_Shore |
| cg20718434 | 4.87E-07 | ELFN1 | TSS200 | Open Sea |
| cg20721738 | 3.46E-07 | RAB36 | TSS1500 | N_Shore |
| cg20727217 | 6.75E-07 | SFRP2 | TSS1500 | N_Shore |
| cg20730333 | 1.32E-07 | PNLIPRP1 | TSS1500 | Open Sea |
| cg20738500 | 5.94E-07 | LOC254559 | TSS200 | Island |
| cg20740051 | 7.84E-09 | B4GALT3 | TSS1500 | S_Shore |
| cg20742696 | 3.99E-07 | LEFTY2 | TSS1500 | S_Shelf |
| cg20742981 | 6.69E-07 | LIMK2 | TSS200 | Open Sea |
| cg20747380 | 2.42E-08 | HOXA2 | 1stExon | N_Shore |
| cg20768743 | 1.32E-08 | CD226 | TSS1500 | Open Sea |
| cg20769842 | 3.67E-07 | MKRN3 | TSS1500 | Open Sea |
| cg20777437 | 6.10E-08 | CDCP2 | TSS1500 | Open Sea |
| cg20778688 | 1.43E-08 | ACACA | TSS1500 | Open Sea |
| cg20779964 | 2.29E-08 | CBLN4 | TSS200 | Island |
| cg20780850 | 3.46E-07 | C1orf114 | TSS1500 | S_Shore |
| cg20791593 | 1.15E-07 | NEU4 | TSS1500 | N_Shelf |
| cg20792895 | 1.09E-08 | MKRN3 | TSS200 | Open Sea |
| cg20797216 | 3.16E-07 | FBN3 | 1stExon | N_Shelf |
| cg20817941 | 4.99E-07 | CTSK | 1stExon | Open Sea |
| cg20847668 | 1.15E-07 | NOP10 | TSS1500 | Open Sea |
| cg20862097 | 9.33E-07 | DAAM2 | TSS200 | S_Shore |
| cg20864636 | 7.89E-12 | GARNL3 | TSS1500 | Open Sea |
| cg20886049 | 1.23E-07 | TSKU | TSS1500 | N_Shore |
| cg20889395 | 8.41E-08 | WFIKKN2 | TSS200 | Open Sea |
| cg20893542 | 1.31E-07 | LPP | TSS1500 | Open Sea |
| cg20903900 | 5.02E-08 | MKS1 | TSS1500 | S_Shore |
| cg20911718 | 1.14E-07 | NYNRIN | TSS1500 | N_Shore |
| cg20916578 | 7.54E-07 | LYPLA1 | TSS1500 | S_Shore |
| cg20924286 | 8.21E-07 | CLDN11 | TSS1500 | N_Shore |
| cg20925954 | 1.04E-07 | DUSP5 | TSS1500 | N_Shore |
| cg20938170 | 8.51E-07 | MOGAT2 | TSS200 | Open Sea |
| cg20941110 | 2.90E-07 | BNC1 | TSS1500 | Island |
| cg20941737 | 4.91E-07 | ZBTB20 | 1stExon | Open Sea |
| cg20945085 | 7.93E-08 | C2orf65 | TSS200 | Island |
| cg20947127 | 1.96E-07 | TBC1D26 | TSS200 | Open Sea |
| cg20951444 | 8.71E-09 | FNDC5 | TSS1500 | Open Sea |
| cg20958753 | 2.62E-07 | KCNK6 | TSS1500 | N_Shore |
| cg20972214 | 3.99E-08 | SLC2A3 | 1stExon | Open Sea |
| cg20973931 | 7.91E-07 | CTSD | TSS1500 | S_Shore |
| cg20975835 | 5.98E-07 | CCDC88B | TSS1500 | N_Shelf |
| cg20976581 | 1.88E-07 | GAS2L2 | TSS1500 | Open Sea |
| cg20979153 | 3.05E-07 | ZNF217 | TSS200 | S_Shore |
| cg20988073 | 1.40E-07 | ALDH1L1 | TSS1500 | S_Shore |
| cg20988616 | 3.28E-08 | ENPP3 | TSS1500 | Open Sea |
| cg20995127 | 3.63E-08 | RAB3GAP1 | TSS1500 | Open Sea |
| cg20998539 | 9.90E-10 | ERN1 | TSS1500 | S_Shore |
| cg21002224 | 2.76E-07 | SPTBN2 | TSS200 | Open Sea |
| cg21012874 | 2.41E-08 | SNCG | 1stExon | Open Sea |
| cg21022906 | 5.79E-07 | C14orf182 | 1stExon | S_Shelf |
| cg21032074 | 2.45E-08 | C6orf94 | TSS1500 | Open Sea |
| cg21033861 | 3.79E-07 | NUBPL | TSS1500 | Open Sea |
| cg21035374 | 3.38E-09 | NWD1 | 1stExon | Open Sea |
| cg21037006 | 4.61E-09 | CALD1 | TSS1500 | Open Sea |
| cg21038703 | 2.72E-07 | ASB16 | 1stExon | Open Sea |
| cg21048669 | 1.95E-07 | CLEC3A | TSS200 | Open Sea |
| cg21052415 | 9.91E-07 | FAM123A | TSS1500 | S_Shore |
| cg21058822 | 1.17E-07 | MBOAT4 | TSS200 | Open Sea |
| cg21068293 | 2.70E-07 | STRA6 | TSS1500 | Open Sea |
| cg21091547 | 5.46E-07 | CDKN1A | TSS1500 | N_Shore |
| cg21097733 | 2.04E-07 | HOXB2 | TSS1500 | S_Shelf |
| cg21104276 | 1.19E-07 | FZD1 | 1stExon | Island |
| cg21109038 | 4.68E-07 | ZNF710 | TSS1500 | Island |
| cg21111471 | 2.66E-07 | FLRT1 | TSS1500 | Open Sea |
| cg21116266 | 3.69E-07 | ITGBL1 | 1stExon | N_Shore |
| cg21116457 | 1.39E-07 | SLC14A1 | TSS200 | Open Sea |
| cg21127537 | 1.90E-08 | TTC12 | TSS1500 | N_Shore |
| cg21139496 | 1.23E-07 | DERL1 | TSS1500 | S_Shore |
| cg21144941 | 1.22E-07 | CECR1 | TSS200 | Open Sea |
| cg21146537 | 4.98E-07 | TOM1 | TSS1500 | N_Shore |
| cg21156057 | 8.55E-07 | ABL1 | TSS1500 | N_Shore |
| cg21157923 | 5.96E-07 | LCN10 | TSS1500 | N_Shelf |
| cg21158317 | 3.22E-07 | RNF112 | TSS200 | Open Sea |
| cg21166445 | 7.67E-08 | UBE2E3 | TSS1500 | N_Shore |
| cg21180956 | 9.93E-08 | ESM1 | TSS200 | Open Sea |
| cg21184174 | 2.74E-07 | NGF | TSS1500 | Island |
| cg21197216 | 9.15E-07 | C1orf94 | TSS200 | S_Shore |
| cg21198755 | 7.06E-07 | C14orf180 | TSS200 | Open Sea |
| cg21203643 | 1.29E-07 | DEGS2 | TSS1500 | S_Shore |
| cg21205978 | 4.45E-07 | APOL6 | TSS1500 | Open Sea |
| cg21208744 | 6.58E-07 | MTMR11 | TSS1500 | Open Sea |
| cg21214613 | 5.18E-08 | HSPB7 | 1stExon | Open Sea |
| cg21225804 | 8.92E-07 | FREQ | TSS1500 | Island |
| cg21237418 | 8.90E-07 | RAB34 | TSS200 | Island |
| cg21246872 | 8.53E-07 | CASC3 | TSS1500 | Open Sea |
| cg21247948 | 1.85E-07 | PRKD3 | TSS1500 | Open Sea |
| cg21250100 | 3.29E-07 | CSDC2 | TSS200 | Open Sea |
| cg21250978 | 2.44E-07 | PRKAR2B | TSS1500 | N_Shore |
| cg21251785 | 1.97E-07 | TRPM3 | TSS1500 | Open Sea |
| cg21266845 | 9.21E-08 | DPT | TSS200 | Open Sea |
| cg21275283 | 6.92E-08 | MYO15A | TSS1500 | Open Sea |
| cg21291672 | 7.87E-07 | TMEM200B | TSS1500 | Island |
| cg21295575 | 2.55E-08 | TM7SF4 | 1stExon | Open Sea |
| cg21296602 | 8.60E-07 | TAF1D | TSS1500 | S_Shore |
| cg21304158 | 1.31E-07 | SNED1 | TSS1500 | N_Shore |
| cg21313071 | 2.14E-08 | KCNG1 | TSS1500 | S_Shore |
| cg21320793 | 1.11E-09 | PDE6B | TSS200 | N_Shore |
| cg21336116 | 7.33E-09 | KRT23 | 1stExon | Open Sea |
| cg21340621 | 4.91E-07 | KDM2B | TSS1500 | S_Shore |
| cg21340733 | 1.84E-07 | KRT8 | TSS200 | S_Shore |
| cg21343189 | 1.52E-07 | LDB3 | TSS1500 | Open Sea |
| cg21350115 | 7.96E-11 | CALCRL | 1stExon | Open Sea |
| cg21361134 | 3.35E-08 | CLRN1 | 1stExon | Open Sea |
| cg21366602 | 2.83E-11 | EPS15 | 1stExon | Open Sea |
| cg21370522 | 3.64E-07 | TNF | TSS200 | Open Sea |
| cg21385748 | 1.88E-08 | LIMS1 | TSS1500 | Open Sea |
| cg21396064 | 3.14E-08 | MIR193B | TSS200 | S_Shore |
| cg21424940 | 2.77E-07 | TWIST1 | TSS1500 | S_Shore |
| cg21432842 | 2.66E-07 | CSF3 | 1stExon | Open Sea |
| cg21448423 | 1.85E-07 | ACOT11 | 1stExon | Open Sea |
| cg21451869 | 4.58E-07 | MMP9 | TSS1500 | N_Shelf |
| cg21463380 | 8.85E-10 | SST | TSS1500 | S_Shore |
| cg21466821 | 4.29E-07 | HIPK4 | TSS200 | S_Shore |
| cg21468889 | 6.39E-07 | FUCA2 | TSS1500 | S_Shore |
| cg21475150 | 4.80E-08 | RPL31 | TSS1500 | Island |
| cg21477262 | 8.34E-07 | ETNK1 | TSS1500 | N_Shore |
| cg21480743 | 6.40E-07 | KILLIN | 1stExon | N_Shore |
| cg21481950 | 7.08E-07 | GABBR1 | TSS1500 | S_Shore |
| cg21484964 | 1.12E-07 | PGBD4 | 1stExon | S_Shore |
| cg21488617 | 4.93E-07 | DNALI1 | 1stExon | Island |
| cg21490635 | 2.75E-08 | EMP1 | TSS1500 | Open Sea |
| cg21490751 | 6.29E-07 | MIR551B | TSS1500 | Open Sea |
| cg21491711 | 2.77E-07 | DBN1 | TSS1500 | Open Sea |
| cg21493505 | 9.42E-07 | ZNF542 | TSS1500 | Island |
| cg21499175 | 5.14E-08 | HERV-FRD | TSS1500 | Open Sea |
| cg21500895 | 2.31E-11 | BTBD11 | TSS1500 | N_Shore |
| cg21502048 | 7.03E-07 | PTGER2 | TSS1500 | N_Shore |
| cg21504353 | 8.69E-07 | SUSD2 | TSS200 | Open Sea |
| cg21506509 | 3.04E-07 | MN1 | 1stExon | Island |
| cg21514857 | 3.01E-08 | PXDNL | 1stExon | Open Sea |
| cg21534931 | 2.96E-08 | SDHB | TSS1500 | S_Shore |
| cg21535366 | 7.95E-07 | BTBD11 | TSS200 | N_Shore |
| cg21543918 | 2.43E-07 | NHSL1 | 1stExon | Open Sea |
| cg21548950 | 2.27E-07 | RHCG | TSS1500 | S_Shore |
| cg21558752 | 4.24E-07 | KRT4 | TSS1500 | Open Sea |
| cg21560076 | 2.03E-07 | EXPH5 | TSS200 | Open Sea |
| cg21561492 | 2.18E-07 | KCNE4 | 1stExon | N_Shore |
| cg21572722 | 1.94E-07 | ELOVL2 | TSS1500 | Island |
| cg21572731 | 3.06E-09 | C3orf52 | TSS200 | Island |
| cg21580089 | 4.63E-07 | LNX1 | TSS200 | Open Sea |
| cg21601837 | 3.09E-07 | ALDH1L1 | TSS1500 | S_Shore |
| cg21601845 | 8.15E-11 | GFAP | TSS200 | S_Shelf |
| cg21609024 | 5.61E-08 | LRP8 | TSS1500 | S_Shore |
| cg21615397 | 3.05E-09 | NEDD4L | TSS200 | S_Shore |
| cg21619325 | 1.79E-09 | OSR1 | TSS1500 | N_Shore |
| cg21620540 | 4.10E-08 | DKK3 | TSS1500 | S_Shore |
| cg21622977 | 7.92E-08 | RBPMS | TSS1500 | N_Shore |
| cg21626848 | 4.94E-07 | SC65 | TSS1500 | S_Shore |
| cg21630608 | 1.42E-08 | SFRP2 | TSS1500 | N_Shore |
| cg21631754 | 1.85E-08 | EXOC3L2 | TSS200 | Island |
| cg21640432 | 2.22E-07 | FFAR2 | TSS1500 | Open Sea |
| cg21642076 | 2.02E-07 | TMEM191A | TSS1500 | N_Shore |
| cg21655830 | 4.70E-07 | C21orf84 | TSS1500 | Open Sea |
| cg21657059 | 3.24E-07 | SFRP2 | TSS1500 | N_Shore |
| cg21671476 | 8.32E-07 | MYL9 | TSS1500 | S_Shore |
| cg21686694 | 1.04E-08 | RNF220 | TSS1500 | N_Shore |
| cg21703322 | 8.76E-07 | LAYN | TSS200 | Island |
| cg21711132 | 4.29E-07 | GABRG3 | TSS1500 | Island |
| cg21714061 | 2.31E-07 | ATXN7L3 | TSS200 | N_Shore |
| cg21721340 | 5.93E-10 | SOX18 | TSS1500 | S_Shore |
| cg21722785 | 4.29E-07 | C17orf44 | TSS200 | S_Shore |
| cg21723903 | 1.28E-08 | EFCAB4B | TSS1500 | Island |
| cg21733927 | 9.99E-07 | SEPT9 | 1stExon | Open Sea |
| cg21735670 | 2.67E-08 | CRYGC | TSS1500 | Open Sea |
| cg21752601 | 4.43E-07 | UCN | TSS200 | Island |
| cg21757872 | 2.19E-09 | BTBD11 | TSS200 | N_Shore |
| cg21766592 | 1.31E-07 | SLC1A5 | 1stExon | N_Shelf |
| cg21769117 | 8.11E-07 | CLIC1 | TSS1500 | N_Shelf |
| cg21801984 | 4.91E-08 | DDX53 | TSS1500 | Open Sea |
| cg21806985 | 1.25E-08 | MCC | TSS200 | S_Shore |
| cg21813101 | 2.63E-08 | RPL22 | TSS1500 | S_Shore |
| cg21813747 | 6.58E-08 | NBL1 | TSS1500 | N_Shore |
| cg21817179 | 2.40E-08 | SOSTDC1 | 1stExon | Open Sea |
| cg21818141 | 7.42E-09 | OSGEPL1 | TSS1500 | Open Sea |
| cg21821913 | 9.30E-07 | RPRM | TSS1500 | S_Shore |
| cg21830459 | 3.15E-07 | CECR1 | TSS200 | Open Sea |
| cg21834048 | 1.67E-08 | GRK7 | TSS1500 | N_Shore |
| cg21843594 | 8.18E-08 | HORMAD2 | TSS200 | Island |
| cg21858516 | 6.08E-07 | DACT1 | TSS1500 | N_Shore |
| cg21870884 | 1.05E-08 | GPR25 | 1stExon | Island |
| cg21874193 | 1.25E-07 | TAS2R19 | 1stExon | Open Sea |
| cg21880903 | 2.37E-07 | KLB | 1stExon | Open Sea |
| cg21881327 | 1.34E-09 | GGT1 | TSS200 | Open Sea |
| cg21890667 | 2.83E-07 | HORMAD2 | TSS1500 | Island |
| cg21899500 | 3.06E-07 | GRM2 | TSS1500 | Island |
| cg21901307 | 7.50E-07 | PDE3B | TSS1500 | N_Shore |
| cg21908828 | 1.46E-07 | KIAA0922 | TSS1500 | N_Shore |
| cg21911021 | 6.34E-07 | ZIK1 | TSS1500 | Island |
| cg21923770 | 8.02E-07 | TUSC5 | 1stExon | Open Sea |
| cg21931680 | 8.69E-12 | AMPD1 | TSS1500 | Open Sea |
| cg21936959 | 1.15E-07 | MRGPRF | TSS1500 | S_Shelf |
| cg21940640 | 3.10E-07 | PHKG1 | TSS1500 | Open Sea |
| cg21944455 | 2.42E-08 | GFAP | TSS1500 | Open Sea |
| cg21950720 | 2.92E-07 | EXTL1 | TSS1500 | Open Sea |
| cg21962791 | 7.27E-08 | PYROXD1 | TSS1500 | N_Shore |
| cg21971206 | 3.89E-07 | PIK3R6 | TSS200 | Open Sea |
| cg21988972 | 5.16E-07 | AFF3 | TSS1500 | Open Sea |
| cg21991616 | 1.26E-07 | HGC6.3 | TSS1500 | Island |
| cg21993406 | 9.83E-07 | CENPH | TSS1500 | N_Shore |
| cg21995347 | 7.69E-08 | SNX18 | 1stExon | Island |
| cg21996245 | 2.03E-07 | B4GALNT4 | TSS1500 | Open Sea |
| cg22004774 | 9.55E-08 | IL22RA2 | TSS1500 | Open Sea |
| cg22008490 | 6.55E-07 | PTPN7 | TSS200 | Open Sea |
| cg22013966 | 5.75E-08 | SERPINA13 | TSS1500 | Open Sea |
| cg22019970 | 1.22E-09 | PAFAH2 | TSS1500 | S_Shore |
| cg22020227 | 1.54E-07 | OLFML3 | TSS1500 | Open Sea |
| cg22027433 | 9.15E-09 | ERN1 | TSS1500 | Island |
| cg22041417 | 3.01E-07 | TPM4 | TSS200 | N_Shore |
| cg22045990 | 7.26E-07 | TSSK3 | 1stExon | S_Shore |
| cg22047282 | 1.01E-08 | EMILIN1 | 1stExon | N_Shelf |
| cg22057050 | 5.10E-07 | KIAA1310 | TSS1500 | Island |
| cg22064048 | 2.83E-07 | TRIM59 | TSS1500 | S_Shore |
| cg22070156 | 3.55E-07 | ITGBL1 | 1stExon | N_Shore |
| cg22077553 | 2.74E-07 | MYOC | 1stExon | Open Sea |
| cg22079747 | 9.74E-07 | WDR69 | TSS200 | N_Shore |
| cg22082456 | 1.76E-08 | SCP2 | TSS1500 | N_Shore |
| cg22083047 | 4.90E-08 | PRICKLE2 | TSS1500 | Open Sea |
| cg22083325 | 7.76E-07 | LOC440461 | TSS200 | Island |
| cg22090713 | 2.79E-07 | RASSF1 | TSS1500 | S_Shore |
| cg22095604 | 6.24E-07 | NOL4 | 1stExon | Island |
| cg22106220 | 1.60E-07 | LRP5L | TSS200 | S_Shelf |
| cg22107533 | 7.53E-08 | TRIM69 | TSS1500 | Open Sea |
| cg22108980 | 7.63E-07 | IGFBP1 | TSS200 | N_Shore |
| cg22111442 | 3.28E-09 | SNORD85 | TSS200 | Open Sea |
| cg22129545 | 5.80E-07 | UCN | TSS200 | Island |
| cg22134923 | 1.40E-07 | AHNAK | TSS1500 | Island |
| cg22158648 | 1.07E-07 | CABLES1 | TSS1500 | N_Shore |
| cg22161115 | 1.62E-07 | UGT2B15 | 1stExon | Open Sea |
| cg22165175 | 9.19E-07 | KCNA2 | TSS1500 | N_Shore |
| cg22168489 | 8.39E-09 | WDR66 | TSS1500 | N_Shore |
| cg22181664 | 2.51E-07 | CLCA1 | TSS200 | Open Sea |
| cg22189286 | 6.32E-07 | HSPB8 | TSS1500 | Open Sea |
| cg22197830 | 1.52E-07 | TXNDC15 | 1stExon | N_Shore |
| cg22198132 | 1.99E-13 | HOXC4 | TSS1500 | N_Shore |
| cg22210627 | 5.66E-07 | LXN | TSS200 | Open Sea |
| cg22214889 | 9.70E-07 | BCAS4 | TSS1500 | N_Shore |
| cg22221074 | 7.62E-08 | EFCAB4B | TSS1500 | S_Shore |
| cg22222663 | 4.08E-08 | TMPRSS13 | TSS1500 | Open Sea |
| cg22229016 | 2.89E-09 | SEMA3B | TSS1500 | Open Sea |
| cg22239334 | 5.65E-07 | CNTD1 | TSS1500 | N_Shore |
| cg22262544 | 9.95E-07 | MIR508 | TSS200 | Open Sea |
| cg22262704 | 2.10E-07 | CST5 | TSS1500 | Open Sea |
| cg22264436 | 3.14E-08 | SOST | TSS1500 | S_Shelf |
| cg22283643 | 5.28E-07 | TUSC5 | 1stExon | Open Sea |
| cg22284422 | 5.19E-07 | LOC284276 | TSS200 | Open Sea |
| cg22289837 | 7.30E-07 | CA3 | TSS1500 | N_Shore |
| cg22292345 | 4.39E-08 | LOC100129354 | TSS1500 | Island |
| cg22302929 | 5.47E-07 | SCAND3 | 1stExon | N_Shore |
| cg22304662 | 2.72E-08 | ZBTB47 | TSS1500 | N_Shore |
| cg22315192 | 3.48E-07 | SLC22A18 | TSS200 | N_Shelf |
| cg22317989 | 4.43E-07 | TNNT3 | 1stExon | Open Sea |
| cg22325294 | 6.30E-07 | RNF39 | TSS1500 | S_Shore |
| cg22328208 | 5.41E-09 | TSPYL5 | 1stExon | Island |
| cg22331159 | 8.74E-07 | INPP5D | TSS1500 | N_Shore |
| cg22331349 | 4.30E-07 | ZNF577 | TSS200 | Island |
| cg22331862 | 7.66E-07 | SLC6A11 | TSS200 | Island |
| cg22332666 | 8.08E-07 | OSGEPL1 | TSS1500 | Open Sea |
| cg22333888 | 1.36E-07 | ADAM33 | TSS1500 | S_Shore |
| cg22344162 | 7.85E-08 | CREG1 | TSS1500 | S_Shore |
| cg22363327 | 2.76E-09 | SFRS13B | TSS1500 | Island |
| cg22365906 | 6.25E-07 | ANKS1B | TSS200 | Open Sea |
| cg22375610 | 1.94E-08 | APOBEC2 | 1stExon | Open Sea |
| cg22377963 | 3.45E-07 | BCL7C | TSS1500 | S_Shore |
| cg22378252 | 1.81E-08 | GCNT2 | TSS200 | Open Sea |
| cg22378459 | 1.11E-07 | TNNT3 | TSS200 | Open Sea |
| cg22392708 | 2.29E-09 | KRT23 | 1stExon | Open Sea |
| cg22431093 | 4.90E-07 | FGD4 | TSS200 | Open Sea |
| cg22436963 | 5.13E-08 | C1orf201 | 1stExon | Open Sea |
| cg22454769 | 5.49E-08 | FHL2 | TSS200 | Island |
| cg22469388 | 1.72E-07 | WFIKKN2 | 1stExon | Open Sea |
| cg22481448 | 1.62E-07 | SLC9A1 | 1stExon | N_Shore |
| cg22488259 | 2.07E-07 | NAV1 | TSS1500 | N_Shore |
| cg22493877 | 4.36E-07 | SPSB1 | TSS1500 | Open Sea |
| cg22505395 | 1.26E-07 | SLC6A13 | TSS200 | Open Sea |
| cg22505962 | 4.48E-07 | CLEC3B | TSS200 | Open Sea |
| cg22505977 | 2.15E-09 | SLC36A2 | TSS1500 | Open Sea |
| cg22507723 | 5.90E-07 | AGAP2 | 1stExon | Island |
| cg22516643 | 6.49E-07 | NTNG1 | TSS1500 | N_Shore |
| cg22517740 | 3.24E-07 | RHOU | TSS1500 | N_Shore |
| cg22534509 | 5.53E-07 | GPR81 | 1stExon | Open Sea |
| cg22534898 | 4.37E-07 | ITIH4 | TSS1500 | Open Sea |
| cg22542731 | 3.91E-09 | LIMS2 | TSS1500 | Open Sea |
| cg22546122 | 2.85E-07 | TPM4 | TSS200 | N_Shore |
| cg22549408 | 4.43E-07 | PMAIP1 | TSS1500 | N_Shore |
| cg22564695 | 7.61E-08 | DAND5 | 1stExon | Open Sea |
| cg22588640 | 6.76E-08 | KCNIP3 | 1stExon | Open Sea |
| cg22595230 | 5.70E-08 | CENPB | 1stExon | Island |
| cg22603393 | 1.10E-07 | KIAA0754 | 1stExon | S_Shelf |
| cg22614400 | 4.25E-07 | GIGYF1 | TSS1500 | S_Shore |
| cg22621652 | 1.19E-07 | FAM180A | TSS200 | Open Sea |
| cg22628873 | 8.81E-09 | GGT6 | TSS1500 | Open Sea |
| cg22660207 | 3.05E-09 | SCRG1 | TSS200 | Open Sea |
| cg22664064 | 3.43E-07 | NMI | TSS1500 | S_Shore |
| cg22675922 | 4.48E-08 | KCNQ1DN | TSS1500 | N_Shore |
| cg22677858 | 5.65E-08 | IPO9 | TSS1500 | N_Shore |
| cg22682201 | 4.77E-10 | APOBEC2 | TSS200 | Open Sea |
| cg22684041 | 5.90E-07 | SCAMP4 | TSS1500 | N_Shore |
| cg22688160 | 1.63E-10 | SDHB | TSS1500 | S_Shore |
| cg22689909 | 1.17E-07 | GLRX | TSS1500 | Open Sea |
| cg22690720 | 2.74E-08 | SAC3D1 | TSS1500 | N_Shore |
| cg22704788 | 8.10E-07 | PRELP | TSS1500 | Open Sea |
| cg22707705 | 1.97E-07 | CYP27A1 | TSS1500 | N_Shore |
| cg22709811 | 2.01E-07 | DNAJC11 | TSS1500 | S_Shore |
| cg22710865 | 1.43E-09 | ZNF669 | TSS1500 | S_Shore |
| cg22713958 | 1.44E-07 | LGALS3BP | TSS200 | Open Sea |
| cg22715761 | 4.53E-08 | LDB3 | TSS1500 | Open Sea |
| cg22775789 | 5.67E-07 | CS | TSS1500 | S_Shore |
| cg22778790 | 3.72E-07 | ESR2 | TSS1500 | S_Shore |
| cg22790835 | 1.43E-07 | C13orf29 | TSS1500 | Open Sea |
| cg22790931 | 1.54E-07 | POU6F2 | TSS200 | S_Shore |
| cg22793065 | 7.84E-07 | PCDHB4 | 1stExon | Island |
| cg22809047 | 2.20E-08 | RPL31 | TSS1500 | Island |
| cg22815409 | 6.50E-09 | TSPO2 | TSS200 | Open Sea |
| cg22819422 | 7.30E-07 | PFKP | TSS1500 | N_Shore |
| cg22825473 | 9.78E-08 | FAM101A | 1stExon | Open Sea |
| cg22829325 | 7.31E-07 | C8orf31 | TSS200 | Open Sea |
| cg22843712 | 4.55E-08 | MYOM2 | TSS1500 | Open Sea |
| cg22857947 | 1.20E-09 | PRKACA | TSS200 | N_Shelf |
| cg22862003 | 7.26E-07 | MX1 | TSS1500 | N_Shore |
| cg22871947 | 5.54E-07 | C10orf71 | TSS1500 | Open Sea |
| cg22885024 | 3.51E-08 | GEM | TSS1500 | S_Shore |
| cg22889914 | 4.16E-08 | ANKRD2 | TSS1500 | S_Shore |
| cg22894896 | 6.52E-08 | MIR193A | TSS200 | Island |
| cg22897522 | 8.36E-07 | MYH10 | TSS1500 | S_Shore |
| cg22900206 | 3.63E-09 | FAM171B | TSS1500 | N_Shore |
| cg22900415 | 6.26E-09 | GJA3 | TSS1500 | Island |
| cg22910462 | 1.27E-07 | CECR1 | TSS200 | Open Sea |
| cg22912610 | 4.50E-09 | GRIP1 | TSS1500 | Open Sea |
| cg22927302 | 3.62E-11 | SEMA3B | TSS1500 | Open Sea |
| cg22932223 | 9.70E-08 | KIAA2013 | TSS1500 | S_Shore |
| cg22942576 | 2.67E-08 | CSRP1 | TSS1500 | S_Shore |
| cg22948288 | 6.03E-07 | MEOX1 | TSS1500 | Open Sea |
| cg22956023 | 1.19E-07 | LOC729234 | TSS1500 | N_Shore |
| cg22957651 | 2.61E-07 | MSN | TSS1500 | N_Shore |
| cg22960869 | 5.69E-09 | ABI3 | TSS200 | Open Sea |
| cg22971501 | 6.76E-07 | LDLR | TSS1500 | N_Shore |
| cg22979807 | 1.14E-07 | TMEM150B | TSS1500 | Open Sea |
| cg22982570 | 1.20E-09 | PCBP4 | TSS200 | Open Sea |
| cg22986999 | 1.15E-07 | MRGPRF | TSS1500 | S_Shelf |
| cg22990888 | 8.65E-12 | GSR | TSS1500 | S_Shore |
| cg22991320 | 2.42E-08 | C20orf85 | TSS200 | Island |
| cg22996440 | 1.17E-09 | GALNT14 | TSS1500 | Island |
| cg22997113 | 1.16E-07 | HOXA4 | 1stExon | Island |
| cg23008153 | 3.36E-07 | RNF180 | TSS1500 | N_Shore |
| cg23010346 | 2.18E-07 | RHOC | TSS1500 | S_Shore |
| cg23013864 | 9.48E-08 | CAP2 | TSS1500 | Open Sea |
| cg23018111 | 2.84E-07 | DKFZp566F0947 | TSS1500 | Open Sea |
| cg23029474 | 6.23E-09 | CDO1 | TSS1500 | S_Shore |
| cg23038349 | 4.72E-10 | LY9 | TSS1500 | Open Sea |
| cg23038594 | 1.26E-07 | LOC284688 | TSS1500 | Open Sea |
| cg23047544 | 4.31E-07 | MIR199A1 | TSS200 | Open Sea |
| cg23050079 | 4.51E-10 | LY9 | TSS1500 | Open Sea |
| cg23051392 | 1.77E-07 | C1QTNF3 | TSS200 | Open Sea |
| cg23083424 | 2.00E-08 | SYNPO2L | TSS200 | Open Sea |
| cg23085666 | 6.94E-08 | SLC41A3 | TSS1500 | Open Sea |
| cg23090046 | 6.84E-08 | KLC1 | TSS1500 | Island |
| cg23090372 | 5.87E-09 | FGF6 | TSS1500 | S_Shore |
| cg23097139 | 1.07E-08 | SNUPN | TSS1500 | S_Shore |
| cg23105827 | 2.93E-07 | ARHGEF19 | TSS200 | Open Sea |
| cg23108728 | 6.02E-08 | TUBA4B | TSS200 | N_Shore |
| cg23109344 | 5.17E-07 | C8orf42 | TSS1500 | S_Shore |
| cg23114616 | 1.52E-08 | TNFRSF25 | TSS200 | S_Shore |
| cg23126152 | 1.13E-08 | SSH1 | TSS200 | Open Sea |
| cg23129930 | 1.61E-09 | HOXA6 | 1stExon | Island |
| cg23131355 | 9.72E-08 | STAB2 | TSS1500 | Open Sea |
| cg23136645 | 1.86E-09 | TPO | TSS200 | Open Sea |
| cg23146998 | 8.57E-08 | F10 | TSS200 | S_Shelf |
| cg23152216 | 9.76E-07 | KRAS | TSS1500 | Open Sea |
| cg23165226 | 1.25E-07 | BAT1 | TSS1500 | S_Shore |
| cg23172400 | 3.59E-07 | TP53INP1 | TSS1500 | Island |
| cg23172892 | 8.11E-07 | RHAG | 1stExon | Open Sea |
| cg23178195 | 1.71E-08 | FXYD1 | TSS200 | N_Shelf |
| cg23181159 | 2.01E-07 | FAM8A1 | TSS1500 | N_Shore |
| cg23189410 | 7.31E-08 | ZIC4 | TSS1500 | N_Shore |
| cg23190719 | 4.12E-08 | CALD1 | TSS1500 | Open Sea |
| cg23201830 | 1.15E-10 | WDR69 | TSS1500 | N_Shore |
| cg23206851 | 5.02E-07 | HOXA2 | TSS1500 | N_Shore |
| cg23216745 | 1.33E-07 | PBXIP1 | TSS1500 | Open Sea |
| cg23228587 | 3.20E-09 | TMEM225 | TSS200 | Open Sea |
| cg23242944 | 5.39E-08 | GFAP | TSS1500 | Open Sea |
| cg23244095 | 3.16E-09 | C1orf70 | TSS1500 | Island |
| cg23251170 | 4.08E-07 | GPD1 | TSS1500 | Open Sea |
| cg23255283 | 6.25E-07 | LOC284276 | TSS200 | Open Sea |
| cg23257184 | 3.04E-08 | ZNF681 | TSS1500 | S_Shore |
| cg23257461 | 6.94E-07 | RHBDL2 | TSS1500 | Open Sea |
| cg23268208 | 5.82E-07 | HORMAD2 | TSS200 | Island |
| cg23272544 | 1.15E-07 | AIRE | TSS1500 | Island |
| cg23279355 | 9.37E-07 | CMYA5 | TSS200 | Open Sea |
| cg23282442 | 7.17E-11 | C8orf12 | TSS200 | Open Sea |
| cg23283187 | 2.02E-07 | CPZ | TSS1500 | N_Shore |
| cg23315423 | 5.20E-07 | SNCG | TSS200 | Open Sea |
| cg23323671 | 2.06E-08 | STMN1 | TSS1500 | Island |
| cg23331484 | 1.16E-07 | SLC7A10 | TSS1500 | S_Shore |
| cg23345084 | 5.22E-08 | TMEM178 | TSS1500 | N_Shore |
| cg23350385 | 6.94E-08 | CCRL2 | TSS1500 | Open Sea |
| cg23361275 | 7.57E-07 | MFHAS1 | 1stExon | Island |
| cg23366166 | 8.85E-08 | FEZ1 | TSS1500 | S_Shore |
| cg23378033 | 4.61E-07 | MSRB3 | TSS1500 | N_Shore |
| cg23396935 | 1.62E-08 | FAM3A | TSS1500 | Island |
| cg23411274 | 4.72E-07 | BTN2A3 | TSS1500 | Open Sea |
| cg23417875 | 3.62E-07 | MAP4K4 | TSS1500 | N_Shore |
| cg23427912 | 6.22E-07 | TRIML1 | TSS1500 | Open Sea |
| cg23447569 | 2.54E-07 | AHNAK | TSS1500 | S_Shore |
| cg23449659 | 2.27E-07 | TRPV6 | 1stExon | Open Sea |
| cg23449696 | 2.37E-08 | ZIC1 | TSS1500 | N_Shore |
| cg23460950 | 1.80E-07 | CLU | TSS200 | Island |
| cg23477967 | 8.69E-13 | TFPI | 1stExon | Open Sea |
| cg23479922 | 4.89E-07 |  | 1stExon | Island |
| cg23482427 | 4.17E-08 | C2orf70 | TSS1500 | Island |
| cg23491841 | 3.55E-07 | C2orf85 | TSS1500 | N_Shelf |
| cg23494957 | 3.45E-07 | ESR2 | TSS1500 | S_Shore |
| cg23499955 | 4.50E-07 | C10orf128 | TSS200 | Open Sea |
| cg23502162 | 8.59E-07 | COL3A1 | TSS200 | Open Sea |
| cg23504215 | 3.29E-07 | TDRD1 | TSS200 | Island |
| cg23512558 | 7.20E-07 | MAP1LC3B2 | TSS200 | Open Sea |
| cg23522522 | 8.69E-07 | DLK1 | TSS1500 | N_Shore |
| cg23523215 | 5.02E-08 | ALS2CR4 | TSS1500 | S_Shore |
| cg23523368 | 1.73E-11 | AMPD1 | TSS1500 | Open Sea |
| cg23533926 | 1.04E-09 | MYL2 | TSS1500 | Open Sea |
| cg23534216 | 7.67E-09 | KAZALD1 | 1stExon | Island |
| cg23584647 | 2.07E-07 | BEST1 | TSS200 | Open Sea |
| cg23595055 | 3.72E-08 | DUSP27 | TSS1500 | Open Sea |
| cg23596673 | 3.82E-09 | GPLD1 | TSS1500 | N_Shelf |
| cg23605991 | 1.74E-07 | PDE6B | TSS1500 | N_Shore |
| cg23616212 | 7.84E-07 | SORT1 | TSS1500 | S_Shore |
| cg23620639 | 3.29E-08 | COL13A1 | TSS1500 | N_Shore |
| cg23621013 | 5.78E-08 | FAM180A | 1stExon | Open Sea |
| cg23621496 | 1.83E-09 | RD3 | TSS1500 | Open Sea |
| cg23634451 | 2.63E-09 | ABI3BP | TSS1500 | Open Sea |
| cg23639734 | 1.59E-07 | SOD3 | TSS1500 | Open Sea |
| cg23646820 | 1.83E-07 | GPR137B | TSS1500 | N_Shore |
| cg23651356 | 9.64E-10 | RGS17 | TSS1500 | S_Shore |
| cg23653457 | 2.50E-09 | LIMCH1 | TSS1500 | N_Shore |
| cg23659307 | 1.58E-07 | HBE1 | TSS1500 | Open Sea |
| cg23665568 | 4.06E-07 | BCL10 | TSS1500 | S_Shelf |
| cg23666844 | 1.20E-07 | CRYGN | TSS1500 | S_Shore |
| cg23669287 | 1.99E-13 | NR2F2 | TSS1500 | N_Shore |
| cg23674169 | 2.70E-07 | MUM1 | TSS200 | S_Shore |
| cg23676369 | 4.72E-08 | CASS4 | TSS1500 | Open Sea |
| cg23681095 | 3.58E-07 | TSSK4 | TSS1500 | Open Sea |
| cg23685969 | 6.92E-08 | ASCC1 | TSS1500 | S_Shore |
| cg23699648 | 2.85E-08 | GJA1 | TSS200 | Open Sea |
| cg23704362 | 2.87E-10 | C8orf46 | 1stExon | Open Sea |
| cg23705113 | 4.68E-07 | THRSP | TSS200 | Open Sea |
| cg23705224 | 1.68E-07 | CTXN2 | 1stExon | Open Sea |
| cg23712462 | 1.34E-07 | FAM135B | TSS1500 | S_Shore |
| cg23717187 | 4.43E-08 | MED24 | TSS1500 | S_Shore |
| cg23725321 | 4.17E-08 | SEC31B | TSS200 | Island |
| cg23728588 | 8.97E-10 | PNLIP | TSS1500 | Open Sea |
| cg23733123 | 1.54E-07 | MARVELD1 | TSS1500 | N_Shore |
| cg23734137 | 6.13E-10 | NR2F2 | TSS1500 | S_Shore |
| cg23741520 | 1.68E-09 | BNC1 | TSS1500 | S_Shore |
| cg23762359 | 5.82E-07 | KCNQ1DN | TSS1500 | N_Shore |
| cg23764129 | 6.44E-07 | HTR3A | 1stExon | Open Sea |
| cg23793500 | 6.08E-07 | GPT | TSS1500 | S_Shore |
| cg23800435 | 1.90E-07 | C1orf210 | TSS200 | Open Sea |
| cg23816737 | 4.07E-07 | TULP4 | 1stExon | Open Sea |
| cg23820560 | 3.37E-08 | MYO18B | TSS1500 | Open Sea |
| cg23827284 | 1.38E-07 | MYLK2 | TSS200 | N_Shelf |
| cg23846443 | 1.65E-09 | NOX3 | TSS1500 | Open Sea |
| cg23849812 | 4.35E-09 | PEG3 | TSS1500 | Open Sea |
| cg23859635 | 5.70E-07 | MTA3 | TSS1500 | N_Shore |
| cg23865597 | 5.73E-08 | DYM | TSS1500 | S_Shore |
| cg23869158 | 2.44E-09 | LRCH1 | TSS1500 | N_Shore |
| cg23884241 | 8.86E-08 | HOXA4 | 1stExon | Island |
| cg23893332 | 1.51E-10 | IFI27 | TSS1500 | Open Sea |
| cg23901857 | 1.57E-08 | HOXD8 | TSS1500 | N_Shore |
| cg23908269 | 4.79E-08 | PANK2 | TSS1500 | N_Shore |
| cg23919118 | 9.94E-07 | RFX4 | TSS1500 | N_Shore |
| cg23924737 | 6.36E-08 | MRPS23 | TSS1500 | S_Shore |
| cg23933044 | 2.15E-07 | C5orf42 | TSS1500 | S_Shore |
| cg23936031 | 5.72E-10 | HOXA5 | 1stExon | Island |
| cg23941394 | 7.04E-09 | PPP2R2D | TSS1500 | Open Sea |
| cg23956385 | 7.42E-07 | C10orf72 | TSS1500 | S_Shore |
| cg23963591 | 4.25E-08 | TAS1R1 | TSS200 | S_Shore |
| cg23973000 | 8.43E-09 | TSSK1B | 1stExon | Open Sea |
| cg23979954 | 8.30E-07 | IFLTD1 | TSS200 | Open Sea |
| cg23981354 | 7.78E-07 | HECA | TSS1500 | N_Shore |
| cg23983449 | 5.95E-07 | GABARAP | TSS1500 | S_Shore |
| cg24002010 | 5.50E-07 | TMEM86B | TSS1500 | Open Sea |
| cg24004007 | 1.85E-07 | FBXL22 | TSS1500 | N_Shelf |
| cg24006253 | 8.67E-07 | VTCN1 | TSS200 | Open Sea |
| cg24033957 | 7.72E-07 | CASP4 | TSS1500 | Open Sea |
| cg24035545 | 1.16E-07 | MYO18B | TSS200 | Open Sea |
| cg24035928 | 2.83E-07 | NRTN | TSS1500 | Island |
| cg24036830 | 8.49E-09 | ZNF389 | TSS1500 | Open Sea |
| cg24040989 | 5.67E-07 | SYNPO | TSS1500 | Open Sea |
| cg24041556 | 7.79E-07 | SLC44A2 | TSS200 | Island |
| cg24049621 | 3.87E-09 | ZNF532 | TSS1500 | N_Shore |
| cg24058132 | 4.64E-07 | GALC | 1stExon | S_Shore |
| cg24059075 | 2.98E-07 | PRPH | TSS1500 | N_Shore |
| cg24074477 | 1.64E-07 | CECR2 | TSS200 | Open Sea |
| cg24074594 | 2.05E-07 | FCRLA | TSS200 | Open Sea |
| cg24076083 | 2.82E-07 | RTBDN | 1stExon | S_Shore |
| cg24079702 | 1.16E-07 | FHL2 | TSS200 | Island |
| cg24082215 | 1.24E-08 | MKS1 | TSS1500 | S_Shore |
| cg24092253 | 3.09E-08 | YTHDF1 | TSS1500 | Island |
| cg24096415 | 9.90E-07 | RILP | TSS200 | S_Shore |
| cg24103040 | 2.80E-08 | GRIP1 | TSS200 | Open Sea |
| cg24105407 | 5.32E-07 | TAS1R1 | 1stExon | S_Shore |
| cg24126851 | 1.62E-07 | DCHS1 | TSS1500 | S_Shore |
| cg24129390 | 1.56E-07 | STARD13 | TSS200 | Open Sea |
| cg24146090 | 1.67E-07 | FAM189A2 | TSS200 | N_Shore |
| cg24147596 | 5.50E-07 | ARL14 | 1stExon | Open Sea |
| cg24149658 | 8.51E-07 | CAPN6 | TSS200 | Open Sea |
| cg24155190 | 1.91E-08 | CSRP1 | TSS1500 | S_Shore |
| cg24166463 | 1.46E-07 | OR51T1 | 1stExon | Open Sea |
| cg24169915 | 2.97E-07 | C14orf177 | 1stExon | Open Sea |
| cg24185852 | 9.45E-08 | MIR585 | TSS200 | Open Sea |
| cg24189734 | 1.76E-07 | LOC255167 | TSS1500 | N_Shore |
| cg24192805 | 1.41E-07 | UTS2R | 1stExon | Island |
| cg24200501 | 1.13E-07 | CYBRD1 | TSS1500 | N_Shore |
| cg24210813 | 4.33E-07 | LOC404266 | TSS200 | Island |
| cg24222083 | 2.36E-07 | KRT222 | TSS1500 | Open Sea |
| cg24222546 | 3.22E-10 | STRA6 | TSS1500 | Open Sea |
| cg24237813 | 1.59E-07 | ATP9A | TSS1500 | S_Shore |
| cg24240870 | 7.50E-07 | GOSR1 | TSS1500 | N_Shore |
| cg24254937 | 7.50E-08 | E2F2 | TSS1500 | S_Shore |
| cg24255269 | 6.57E-08 | PISRT1 | TSS200 | Open Sea |
| cg24257776 | 6.45E-07 | LOC100129354 | TSS1500 | Island |
| cg24262376 | 4.53E-08 | SCNM1 | TSS1500 | Open Sea |
| cg24277586 | 2.91E-07 | C3orf74 | TSS1500 | Open Sea |
| cg24280945 | 6.92E-11 | CSGALNACT1 | TSS1500 | S_Shore |
| cg24302646 | 3.83E-07 | CPN1 | TSS1500 | Open Sea |
| cg24306962 | 1.01E-07 | HSPB7 | TSS1500 | Open Sea |
| cg24309555 | 3.77E-07 | APOB | TSS200 | Island |
| cg24310500 | 2.01E-07 | CPNE5 | TSS1500 | Island |
| cg24315340 | 3.74E-07 | EPM2A | TSS1500 | S_Shore |
| cg24324441 | 2.25E-07 | MFSD1 | TSS1500 | N_Shore |
| cg24332577 | 6.15E-07 | SALL4 | TSS1500 | S_Shore |
| cg24340657 | 3.85E-08 | KRT24 | 1stExon | Open Sea |
| cg24348495 | 6.25E-08 | TCEA2 | TSS200 | Island |
| cg24353535 | 3.39E-07 | ASCL2 | TSS1500 | S_Shore |
| cg24390871 | 7.68E-09 | ART4 | TSS200 | Open Sea |
| cg24391460 | 2.50E-07 | MAGI2 | TSS1500 | S_Shore |
| cg24394097 | 1.31E-07 | KCNAB1 | 1stExon | Open Sea |
| cg24414325 | 2.85E-08 | IKZF4 | TSS1500 | Open Sea |
| cg24427376 | 4.65E-08 | NEXN | TSS200 | N_Shore |
| cg24430528 | 4.19E-09 | G3BP2 | TSS1500 | S_Shore |
| cg24433189 | 1.72E-07 | SSTR5 | TSS200 | Island |
| cg24437523 | 2.30E-07 | ATP1A2 | 1stExon | Open Sea |
| cg24446178 | 3.79E-07 | SLC17A8 | TSS200 | Open Sea |
| cg24448013 | 3.42E-07 | GALE | TSS200 | N_Shore |
| cg24454695 | 6.35E-07 | GNG4 | TSS1500 | Island |
| cg24476103 | 4.49E-07 | RUNX1T1 | TSS1500 | Open Sea |
| cg24477401 | 4.76E-07 | ARHGAP27 | TSS1500 | S_Shore |
| cg24495017 | 8.65E-07 | PITX1 | TSS1500 | S_Shore |
| cg24495528 | 5.29E-07 | RBM20 | TSS1500 | N_Shore |
| cg24498180 | 5.11E-07 | C1orf223 | TSS1500 | S_Shelf |
| cg24508208 | 7.61E-08 | CALD1 | TSS1500 | Open Sea |
| cg24509168 | 2.69E-07 | KCNJ9 | TSS200 | N_Shelf |
| cg24517066 | 4.58E-08 | RSAD1 | TSS1500 | N_Shore |
| cg24520070 | 6.52E-08 | C14orf48 | TSS1500 | Open Sea |
| cg24529771 | 9.35E-07 | KCNMB3 | TSS1500 | N_Shore |
| cg24539599 | 3.54E-07 | MCC | TSS1500 | S_Shore |
| cg24541550 | 4.65E-07 | MRVI1 | TSS1500 | Open Sea |
| cg24557917 | 1.35E-09 | TRIM75 | TSS200 | Open Sea |
| cg24568468 | 5.41E-07 | GLRB | TSS1500 | N_Shore |
| cg24569018 | 2.80E-11 | TMEM89 | TSS200 | Open Sea |
| cg24571875 | 4.12E-10 | PNLIP | TSS1500 | Open Sea |
| cg24578857 | 5.43E-07 | PLD6 | TSS1500 | Island |
| cg24580782 | 1.56E-11 | NR0B2 | 1stExon | Open Sea |
| cg24583987 | 8.33E-07 | GUCA2B | TSS1500 | Open Sea |
| cg24587552 | 4.50E-07 | C2orf83 | TSS1500 | Open Sea |
| cg24598094 | 5.37E-07 | SLC30A1 | TSS1500 | S_Shore |
| cg24601030 | 4.38E-07 | DIXDC1 | TSS1500 | N_Shore |
| cg24603803 | 6.60E-07 | PALMD | 1stExon | Open Sea |
| cg24607398 | 3.70E-08 | EPM2AIP1 | 1stExon | N_Shore |
| cg24612707 | 4.49E-07 | C7orf68 | TSS1500 | N_Shore |
| cg24612990 | 8.67E-08 | LYZL6 | TSS1500 | Open Sea |
| cg24640065 | 2.68E-08 | TARBP1 | TSS1500 | S_Shore |
| cg24645868 | 4.74E-09 | MOXD1 | TSS1500 | S_Shore |
| cg24653728 | 3.37E-07 | OR8B4 | TSS200 | Open Sea |
| cg24657523 | 8.10E-08 | MYEF2 | TSS200 | S_Shore |
| cg24670552 | 8.87E-07 | IRS2 | 1stExon | Island |
| cg24673101 | 9.95E-07 | CCDC85C | 1stExon | Island |
| cg24673765 | 7.00E-09 | HSPB6 | 1stExon | Island |
| cg24676384 | 4.94E-07 | PBXIP1 | TSS1500 | Open Sea |
| cg24681208 | 4.39E-07 | REM2 | TSS1500 | N_Shelf |
| cg24683623 | 8.08E-07 | FGF2 | TSS1500 | N_Shore |
| cg24685778 | 9.67E-08 | TRIM63 | TSS200 | Open Sea |
| cg24691835 | 8.49E-08 | ACTG2 | TSS1500 | Open Sea |
| cg24694549 | 4.66E-09 | GRIP1 | 1stExon | Open Sea |
| cg24701772 | 1.19E-08 | EPS15 | TSS1500 | Open Sea |
| cg24708308 | 1.49E-09 | SCARNA11 | TSS1500 | Open Sea |
| cg24724587 | 5.05E-07 | DDAH1 | TSS200 | S_Shore |
| cg24725079 | 6.71E-07 | PLXNA4 | TSS1500 | S_Shore |
| cg24735937 | 5.31E-07 | CLTB | TSS1500 | S_Shore |
| cg24739098 | 3.39E-07 | RASGRF1 | 1stExon | Open Sea |
| cg24743156 | 6.62E-07 | CTAGE5 | TSS1500 | N_Shore |
| cg24747122 | 4.38E-08 | GNA13 | TSS1500 | S_Shore |
| cg24751551 | 9.48E-10 | KCNJ15 | TSS1500 | Open Sea |
| cg24781100 | 1.14E-07 | MYBPC1 | TSS1500 | Open Sea |
| cg24782497 | 9.86E-08 | KIAA1683 | TSS200 | Open Sea |
| cg24786986 | 3.02E-07 | FOXP2 | 1stExon | Open Sea |
| cg24804106 | 4.75E-08 | PSD3 | TSS1500 | S_Shore |
| cg24814117 | 2.46E-07 | AP3B1 | TSS1500 | S_Shore |
| cg24818566 | 3.18E-08 | DBC1 | TSS1500 | Island |
| cg24865110 | 1.76E-07 | YPEL2 | TSS1500 | N_Shore |
| cg24876187 | 4.78E-07 | CCHCR1 | TSS1500 | S_Shore |
| cg24876897 | 3.15E-09 | DDOST | TSS1500 | S_Shore |
| cg24886219 | 3.48E-08 | DUSP27 | TSS200 | Open Sea |
| cg24886563 | 8.07E-07 | P2RX1 | TSS200 | Open Sea |
| cg24901474 | 6.76E-07 | RGS5 | TSS200 | Open Sea |
| cg24901707 | 2.86E-09 | C8orf12 | TSS200 | Open Sea |
| cg24909309 | 9.69E-08 | C8orf86 | TSS1500 | Open Sea |
| cg24922045 | 7.07E-07 | GAS2L2 | TSS200 | Open Sea |
| cg24922596 | 7.09E-10 | MC2R | TSS200 | Open Sea |
| cg24927890 | 1.52E-07 | SYNPO | TSS200 | Open Sea |
| cg24927974 | 9.35E-07 | DPY19L1 | TSS1500 | S_Shore |
| cg24936980 | 5.78E-08 | SAC3D1 | TSS1500 | N_Shore |
| cg24940601 | 9.61E-07 | KCNE4 | 1stExon | N_Shore |
| cg24946253 | 2.40E-07 | PXDNL | TSS200 | Open Sea |
| cg24966958 | 2.74E-07 | POPDC2 | TSS1500 | Open Sea |
| cg24968721 | 3.24E-07 | SFRP2 | TSS1500 | N_Shore |
| cg24974611 | 4.30E-11 | SEC16B | TSS200 | Open Sea |
| cg24977886 | 6.80E-09 | FAM109B | TSS200 | Island |
| cg24990238 | 2.63E-07 | KCNMB1 | TSS1500 | Open Sea |
| cg24994249 | 6.79E-07 | UBE4A | TSS1500 | N_Shore |
| cg25000555 | 4.70E-07 | DNA2 | TSS1500 | S_Shore |
| cg25008263 | 6.67E-07 | FOXN3 | TSS1500 | S_Shore |
| cg25023291 | 1.65E-08 | TAS2R42 | TSS1500 | Open Sea |
| cg25023761 | 2.22E-08 | MIR320B1 | TSS1500 | S_Shelf |
| cg25030832 | 3.72E-07 | NEU4 | TSS200 | N_Shelf |
| cg25067242 | 8.77E-07 | NGF | TSS1500 | Island |
| cg25087851 | 5.31E-08 | GPR44 | TSS1500 | S_Shelf |
| cg25096142 | 9.36E-11 | SLC9A11 | 1stExon | Open Sea |
| cg25100722 | 4.22E-07 | LOC284688 | TSS200 | Open Sea |
| cg25101936 | 6.03E-09 | ZBTB16 | TSS1500 | N_Shore |
| cg25106036 | 1.10E-07 | HERV-FRD | TSS1500 | Open Sea |
| cg25137841 | 4.82E-07 | PLA2G3 | TSS200 | Open Sea |
| cg25141490 | 6.80E-07 | IL17B | TSS1500 | Open Sea |
| cg25145360 | 1.79E-07 | ALDH3A1 | TSS200 | S_Shelf |
| cg25148589 | 7.48E-07 | GRIA2 | 1stExon | N_Shore |
| cg25153824 | 8.54E-08 | CCDC19 | TSS200 | Island |
| cg25164777 | 5.39E-07 | PXDNL | TSS1500 | Open Sea |
| cg25177139 | 3.43E-10 | SLC10A6 | 1stExon | Open Sea |
| cg25178683 | 1.66E-07 | LGALS3BP | TSS1500 | Open Sea |
| cg25190001 | 7.76E-08 | WNT1 | TSS1500 | N_Shore |
| cg25190824 | 8.24E-07 | BMP8B | TSS1500 | S_Shore |
| cg25206536 | 1.86E-07 | MIR572 | TSS1500 | N_Shore |
| cg25209353 | 7.79E-07 | LDLRAP1 | TSS1500 | N_Shore |
| cg25210134 | 1.35E-07 | PLCB2 | TSS200 | Open Sea |
| cg25212814 | 1.51E-07 | C6orf164 | TSS1500 | Open Sea |
| cg25216196 | 4.07E-08 | MFAP4 | TSS200 | Open Sea |
| cg25228625 | 1.13E-07 | C17orf44 | TSS200 | S_Shore |
| cg25236446 | 4.99E-07 | FOXJ1 | TSS200 | Island |
| cg25241038 | 2.31E-11 | PROM2 | 1stExon | Open Sea |
| cg25245118 | 3.11E-07 | SPDYE4 | TSS200 | Open Sea |
| cg25263238 | 3.98E-07 | BLNK | TSS1500 | Open Sea |
| cg25274320 | 5.47E-07 | ZNHIT2 | TSS1500 | S_Shore |
| cg25279586 | 3.81E-07 | PTPRM | TSS1500 | N_Shore |
| cg25281616 | 3.05E-07 | H19 | TSS1500 | S_Shore |
| cg25283486 | 2.99E-07 | RADIL | TSS1500 | S_Shore |
| cg25292098 | 7.26E-07 | EFCAB8 | TSS1500 | Open Sea |
| cg25298235 | 9.85E-07 | COG5 | TSS1500 | S_Shore |
| cg25302646 | 9.76E-07 | PRDX6 | TSS1500 | N_Shore |
| cg25302985 | 2.95E-07 | LOC400804 | TSS200 | Open Sea |
| cg25306939 | 1.85E-08 | KCNQ1OT1 | TSS1500 | Island |
| cg25312054 | 7.71E-07 | IRS2 | 1stExon | Island |
| cg25313204 | 1.63E-07 | SLC22A3 | TSS1500 | Island |
| cg25330016 | 4.38E-08 | C1QTNF1 | TSS200 | Open Sea |
| cg25340361 | 4.10E-08 | COMMD1 | TSS1500 | Open Sea |
| cg25342125 | 9.00E-12 | CCDC50 | TSS1500 | N_Shore |
| cg25355803 | 4.57E-07 | MARVELD1 | TSS1500 | N_Shore |
| cg25358853 | 2.66E-08 | CCRL2 | TSS1500 | Open Sea |
| cg25362709 | 4.70E-07 | OR8B12 | TSS200 | Open Sea |
| cg25364822 | 8.44E-09 | FNDC5 | TSS1500 | Open Sea |
| cg25365006 | 3.76E-07 | SLC41A3 | TSS1500 | Open Sea |
| cg25371036 | 1.28E-07 | AMOTL1 | TSS1500 | N_Shore |
| cg25371169 | 3.70E-07 | PLEKHA2 | TSS1500 | N_Shore |
| cg25386426 | 3.98E-08 | PANK4 | TSS1500 | N_Shore |
| cg25391117 | 7.90E-07 | C1QTNF3 | 1stExon | Open Sea |
| cg25397191 | 5.15E-09 | PRKACA | TSS200 | N_Shelf |
| cg25400253 | 7.52E-07 | ITGA2B | TSS200 | S_Shelf |
| cg25402083 | 1.89E-09 | GPR20 | TSS1500 | Open Sea |
| cg25407077 | 3.95E-09 | ALPK2 | TSS1500 | Open Sea |
| cg25414605 | 5.69E-07 | ATPGD1 | TSS1500 | N_Shore |
| cg25415932 | 3.00E-07 | OGG1 | TSS1500 | N_Shore |
| cg25422089 | 6.86E-07 | MGC16275 | TSS1500 | S_Shore |
| cg25423004 | 1.36E-08 | HOXD8 | TSS1500 | N_Shore |
| cg25426350 | 2.55E-07 | HPX | TSS200 | Open Sea |
| cg25440680 | 6.04E-07 | STBD1 | TSS1500 | N_Shore |
| cg25451120 | 2.50E-07 | ABI3 | TSS200 | Open Sea |
| cg25461865 | 2.34E-08 | ENPP3 | 1stExon | Open Sea |
| cg25462303 | 3.90E-07 | GCET2 | TSS200 | Open Sea |
| cg25477769 | 9.65E-09 | HNF1A | 1stExon | Island |
| cg25479708 | 3.32E-07 | VAMP2 | TSS1500 | S_Shore |
| cg25499537 | 1.91E-07 | IGSF10 | TSS1500 | N_Shore |
| cg25500444 | 8.91E-07 | CCDC81 | 1stExon | S_Shore |
| cg25509184 | 4.30E-07 | CFTR | TSS1500 | Open Sea |
| cg25518276 | 8.81E-07 | C11orf85 | TSS1500 | Open Sea |
| cg25518824 | 2.15E-07 | LOC284023 | TSS1500 | S_Shore |
| cg25528786 | 6.16E-07 | CEACAM21 | TSS1500 | Open Sea |
| cg25533211 | 2.80E-10 | TSPO2 | TSS1500 | Open Sea |
| cg25533774 | 7.63E-08 | SOSTDC1 | 1stExon | Open Sea |
| cg25540142 | 5.04E-07 | NANOG | TSS200 | Open Sea |
| cg25541621 | 4.22E-07 | FMO6P | TSS200 | Open Sea |
| cg25547939 | 1.14E-07 | VEGFB | TSS1500 | N_Shore |
| cg25548834 | 1.61E-09 | LOC402644 | TSS200 | Open Sea |
| cg25560398 | 2.32E-07 | ECEL1P2 | TSS1500 | Island |
| cg25562031 | 2.44E-07 | MOG | 1stExon | Open Sea |
| cg25567448 | 5.66E-07 | NEURL3 | TSS1500 | S_Shore |
| cg25570676 | 3.98E-07 | SLC8A2 | TSS1500 | Open Sea |
| cg25580198 | 8.99E-07 | GPIHBP1 | TSS200 | Open Sea |
| cg25583503 | 1.47E-11 | UGT2B7 | 1stExon | Open Sea |
| cg25585129 | 4.00E-08 | SMTNL2 | TSS1500 | N_Shore |
| cg25588680 | 1.98E-07 | AOX2P | TSS200 | Open Sea |
| cg25590527 | 3.02E-11 | GGT1 | TSS1500 | Open Sea |
| cg25592910 | 1.04E-07 | PCDH15 | TSS200 | Open Sea |
| cg25595793 | 2.55E-08 | SLC44A2 | TSS200 | Island |
| cg25600446 | 8.92E-07 | GPT | TSS1500 | N_Shore |
| cg25607249 | 2.43E-07 | SLC1A5 | 1stExon | N_Shelf |
| cg25608041 | 2.06E-08 | TBC1D1 | TSS1500 | Island |
| cg25611723 | 5.89E-08 | APCDD1 | TSS1500 | N_Shore |
| cg25623524 | 4.51E-09 | NCRNA00181 | TSS1500 | Island |
| cg25623886 | 4.82E-07 | KRTAP10-6 | 1stExon | Open Sea |
| cg25635500 | 3.26E-07 | RASL10A | TSS1500 | S_Shore |
| cg25647415 | 1.49E-08 | SEMA3B | TSS1500 | Open Sea |
| cg25647583 | 1.11E-07 | FES | TSS1500 | N_Shore |
| cg25658983 | 2.09E-08 | SIAH3 | TSS200 | Open Sea |
| cg25666403 | 7.94E-07 | OSM | TSS200 | Open Sea |
| cg25677394 | 6.90E-08 | HRH1 | TSS200 | Open Sea |
| cg25678231 | 9.17E-07 | ETV3L | TSS200 | Open Sea |
| cg25686172 | 1.01E-09 | LOC150622 | TSS200 | Open Sea |
| cg25692621 | 7.51E-07 | ASB4 | TSS200 | Open Sea |
| cg25710107 | 1.99E-10 | A2LD1 | TSS1500 | S_Shelf |
| cg25716415 | 2.70E-09 | C14orf183 | 1stExon | Open Sea |
| cg25718383 | 1.32E-07 | CPA1 | TSS200 | N_Shore |
| cg25725843 | 6.39E-08 | ST6GAL2 | TSS1500 | Island |
| cg25735482 | 3.09E-07 | TENC1 | TSS1500 | N_Shore |
| cg25738301 | 1.53E-08 | NFYB | TSS1500 | Island |
| cg25740652 | 8.62E-09 | LIMCH1 | TSS1500 | N_Shore |
| cg25742464 | 6.60E-08 | ELF3 | TSS1500 | Open Sea |
| cg25743345 | 6.65E-07 | CASKIN2 | TSS1500 | Open Sea |
| cg25744056 | 5.20E-07 | FAM163B | TSS1500 | S_Shore |
| cg25744957 | 1.64E-08 | FLJ36777 | TSS1500 | Open Sea |
| cg25747783 | 2.93E-07 | FLJ23834 | TSS200 | Open Sea |
| cg25754958 | 9.15E-07 | PTPN7 | TSS1500 | Open Sea |
| cg25758167 | 2.88E-09 | AGL | TSS1500 | N_Shore |
| cg25759381 | 2.03E-07 | TSSK2 | 1stExon | N_Shore |
| cg25767433 | 6.29E-09 | CUTA | TSS1500 | S_Shore |
| cg25771195 | 5.50E-07 | C16orf80 | TSS1500 | S_Shore |
| cg25774643 | 5.58E-07 | SCT | TSS200 | Island |
| cg25779645 | 9.60E-08 | R3HDML | TSS1500 | Open Sea |
| cg25781162 | 4.91E-10 | ABCG8 | TSS200 | Open Sea |
| cg25803107 | 1.51E-08 | LOC150622 | TSS200 | Open Sea |
| cg25804357 | 1.69E-08 | MIR604 | TSS1500 | Open Sea |
| cg25815024 | 2.01E-07 | PRKAG3 | TSS200 | Open Sea |
| cg25816184 | 3.71E-07 | PTPRO | TSS1500 | N_Shore |
| cg25832084 | 2.08E-07 | AQP6 | TSS1500 | S_Shelf |
| cg25840926 | 3.45E-08 | RHOB | 1stExon | Island |
| cg25844605 | 1.09E-07 | LOC149837 | TSS1500 | Open Sea |
| cg25850808 | 7.93E-07 | CD109 | TSS1500 | N_Shore |
| cg25853078 | 4.22E-10 | OPCML | TSS1500 | Open Sea |
| cg25865570 | 2.14E-08 | CUGBP2 | TSS1500 | Open Sea |
| cg25874123 | 1.62E-07 | MYOM1 | TSS200 | Open Sea |
| cg25878131 | 1.73E-08 | ELF1 | TSS1500 | Open Sea |
| cg25879745 | 6.48E-08 | SIGLEC1 | TSS200 | Open Sea |
| cg25881170 | 3.73E-08 | CD47 | TSS1500 | Island |
| cg25882256 | 3.23E-07 | RUNX3 | TSS1500 | Island |
| cg25885803 | 1.90E-08 | MADD | TSS1500 | N_Shore |
| cg25898146 | 1.02E-07 | MYOM2 | TSS1500 | Open Sea |
| cg25906442 | 5.54E-08 | C20orf85 | TSS200 | Island |
| cg25929139 | 1.18E-07 | ELFN1 | TSS1500 | Open Sea |
| cg25936595 | 9.39E-07 | RAX2 | TSS200 | S_Shore |
| cg25953688 | 9.33E-07 | CUGBP1 | TSS1500 | S_Shore |
| cg25959149 | 1.17E-07 | BANF2 | TSS200 | Open Sea |
| cg25960038 | 1.03E-07 | LOC404266 | TSS200 | N_Shore |
| cg25960403 | 2.09E-07 | MIR572 | TSS1500 | N_Shore |
| cg25982743 | 4.80E-07 | TIMP4 | 1stExon | Island |
| cg25985347 | 6.27E-07 | PALLD | TSS200 | Open Sea |
| cg25987804 | 7.62E-08 | RBMXL3 | TSS1500 | N_Shore |
| cg26005232 | 6.13E-08 | NUDC | TSS1500 | N_Shore |
| cg26029547 | 1.83E-07 | STK19 | TSS1500 | N_Shore |
| cg26034769 | 1.07E-08 | NEDD4L | 1stExon | S_Shelf |
| cg26037142 | 3.69E-07 | ATF6B | TSS1500 | N_Shore |
| cg26037821 | 1.97E-07 | BLOC1S1 | TSS1500 | N_Shore |
| cg26051413 | 5.46E-07 | ASCL2 | TSS1500 | Island |
| cg26051750 | 4.22E-08 | LUZP1 | TSS1500 | Open Sea |
| cg26065909 | 4.55E-07 | SELENBP1 | TSS200 | Open Sea |
| cg26069745 | 2.68E-07 | HOXA2 | 1stExon | N_Shore |
| cg26078436 | 2.09E-11 | HBBP1 | TSS1500 | Open Sea |
| cg26090072 | 1.89E-08 | RTKN | TSS1500 | Island |
| cg26094482 | 4.41E-10 | KCNQ1OT1 | TSS1500 | Island |
| cg26095658 | 3.07E-07 | FIGLA | TSS200 | Island |
| cg26108719 | 2.02E-08 | MXRA8 | TSS1500 | Open Sea |
| cg26116551 | 8.49E-09 | ZNF536 | TSS200 | N_Shelf |
| cg26122129 | 1.89E-07 | RBPMS | TSS1500 | N_Shore |
| cg26125600 | 6.62E-07 | PF4V1 | TSS200 | N_Shore |
| cg26129220 | 8.67E-08 | DNAH12 | TSS1500 | Open Sea |
| cg26143719 | 5.41E-07 | C1QTNF6 | 1stExon | Open Sea |
| cg26145670 | 5.06E-07 | SCAND3 | 1stExon | N_Shore |
| cg26152983 | 5.82E-08 | MAGEF1 | 1stExon | N_Shore |
| cg26160945 | 7.20E-11 | STK38L | TSS1500 | N_Shore |
| cg26162108 | 1.38E-10 | HOXC4 | TSS1500 | N_Shore |
| cg26171815 | 1.31E-10 | MIR580 | TSS1500 | N_Shore |
| cg26172016 | 6.65E-09 | ZNF532 | TSS1500 | N_Shore |
| cg26184741 | 2.77E-07 | SRGAP3 | TSS1500 | Open Sea |
| cg26187962 | 3.30E-09 | EPHX1 | TSS1500 | Open Sea |
| cg26190476 | 6.15E-08 | FAM19A3 | TSS1500 | Island |
| cg26191586 | 1.26E-07 | LRP8 | TSS1500 | S_Shore |
| cg26196882 | 9.17E-07 | SV2A | TSS1500 | Open Sea |
| cg26201811 | 3.85E-07 | SHANK3 | TSS1500 | N_Shore |
| cg26206133 | 1.69E-08 | TSSK1B | 1stExon | Open Sea |
| cg26214765 | 6.52E-08 | BRP44L | TSS1500 | S_Shore |
| cg26215428 | 7.24E-08 | PKD2 | TSS1500 | N_Shore |
| cg26218823 | 1.11E-07 | MIR508 | TSS200 | Open Sea |
| cg26220985 | 2.58E-07 | DPT | 1stExon | Open Sea |
| cg26233209 | 3.23E-07 | ATG12 | TSS1500 | S_Shore |
| cg26241462 | 8.36E-08 | DCHS1 | TSS1500 | S_Shore |
| cg26245256 | 1.09E-07 | LOC388428 | TSS200 | N_Shore |
| cg26247508 | 1.78E-07 | SH2D4B | TSS1500 | S_Shelf |
| cg26259363 | 3.37E-07 | DES | TSS1500 | N_Shore |
| cg26265787 | 3.92E-07 | TYK2 | TSS1500 | S_Shore |
| cg26278699 | 5.01E-07 | COL6A3 | TSS200 | Open Sea |
| cg26279021 | 4.40E-07 | TWIST1 | TSS1500 | S_Shore |
| cg26281051 | 4.36E-07 | DEFB129 | TSS200 | Open Sea |
| cg26283426 | 7.11E-08 | SOST | 1stExon | S_Shelf |
| cg26286961 | 6.07E-08 | CSGALNACT1 | TSS200 | S_Shore |
| cg26293512 | 2.26E-07 | TEPP | 1stExon | Open Sea |
| cg26293681 | 4.64E-07 | CCDC67 | TSS1500 | N_Shore |
| cg26306869 | 2.56E-07 | S100A3 | TSS1500 | Open Sea |
| cg26313247 | 3.08E-07 | GPX5 | TSS1500 | Open Sea |
| cg26317834 | 3.96E-08 | SMTNL2 | TSS1500 | N_Shore |
| cg26324978 | 1.48E-08 | S100Z | 1stExon | Open Sea |
| cg26325174 | 7.09E-07 | MEG8 | TSS1500 | Open Sea |
| cg26326633 | 1.64E-07 | ANK1 | 1stExon | Island |
| cg26331326 | 1.81E-08 | KCTD6 | TSS1500 | Open Sea |
| cg26333393 | 3.35E-09 | CPXM2 | TSS1500 | S_Shore |
| cg26334023 | 8.40E-09 | ABI3 | TSS200 | Open Sea |
| cg26337868 | 9.11E-07 | RND3 | TSS1500 | S_Shelf |
| cg26338757 | 4.21E-08 | LDB3 | TSS200 | Open Sea |
| cg26355894 | 3.06E-07 | TNNT3 | TSS200 | Open Sea |
| cg26365553 | 8.83E-09 | MADD | TSS1500 | N_Shore |
| cg26365668 | 1.17E-08 | MRPS18C | TSS1500 | N_Shore |
| cg26376241 | 2.07E-07 | SPRED2 | TSS200 | Open Sea |
| cg26377880 | 2.33E-08 | C5orf49 | TSS1500 | S_Shore |
| cg26389381 | 6.80E-11 | SCEL | TSS1500 | Open Sea |
| cg26392737 | 1.35E-08 | EPB49 | 1stExon | S_Shelf |
| cg26397662 | 1.22E-07 | SNORD115-14 | TSS200 | Open Sea |
| cg26399035 | 4.39E-07 | THUMPD1 | TSS1500 | S_Shore |
| cg26405020 | 5.39E-07 | FES | TSS1500 | N_Shore |
| cg26421140 | 1.03E-07 | HSPA2 | TSS1500 | N_Shore |
| cg26429925 | 2.25E-07 | BNC1 | TSS1500 | Island |
| cg26444282 | 1.61E-09 | SYNPO | 1stExon | Open Sea |
| cg26450387 | 5.37E-10 | TM9SF4 | TSS1500 | Open Sea |
| cg26453588 | 3.77E-07 | BIK | TSS1500 | Island |
| cg26456021 | 5.88E-07 | CEMP1 | 1stExon | N_Shore |
| cg26458072 | 3.19E-07 | SEC31B | TSS200 | Island |
| cg26460678 | 2.37E-07 | HYAL2 | TSS1500 | S_Shore |
| cg26467279 | 1.09E-07 | PMEPA1 | TSS1500 | S_Shore |
| cg26476156 | 9.57E-07 | 8-Mar | TSS1500 | S_Shore |
| cg26479099 | 7.71E-07 | ACTA2 | TSS1500 | Open Sea |
| cg26484631 | 2.66E-07 | ULBP1 | TSS1500 | N_Shore |
| cg26491425 | 7.33E-07 | RHOV | TSS200 | S_Shore |
| cg26497399 | 1.65E-07 | HDAC7 | TSS1500 | S_Shore |
| cg26499232 | 2.33E-07 | HSD17B7P2 | TSS1500 | N_Shore |
| cg26512226 | 4.75E-07 | DES | TSS1500 | N_Shore |
| cg26516004 | 7.47E-07 | CYP1A1 | TSS1500 | S_Shore |
| cg26518431 | 8.68E-07 | MGC12982 | TSS1500 | N_Shore |
| cg26521448 | 2.92E-07 | ZC3H7A | TSS200 | Open Sea |
| cg26529864 | 1.07E-08 | BOLA3 | TSS1500 | S_Shore |
| cg26534847 | 4.40E-07 | BPI | TSS200 | Open Sea |
| cg26539232 | 6.51E-07 | ARID3A | TSS1500 | Island |
| cg26539631 | 7.41E-08 | ATF6B | TSS1500 | N_Shore |
| cg26541780 | 1.10E-07 | PAQR6 | TSS200 | S_Shelf |
| cg26542283 | 1.21E-07 | C5orf39 | TSS200 | Island |
| cg26557179 | 6.07E-07 | RTP3 | TSS1500 | Open Sea |
| cg26557270 | 1.43E-07 | FRK | TSS1500 | Open Sea |
| cg26565099 | 4.63E-07 | TADA1 | TSS1500 | S_Shore |
| cg26572973 | 1.61E-07 | GPT | TSS1500 | N_Shore |
| cg26577320 | 4.84E-07 | KIAA1026 | TSS200 | Open Sea |
| cg26580095 | 5.40E-07 | BTBD8 | TSS1500 | N_Shore |
| cg26585644 | 3.12E-07 | DCI | TSS1500 | S_Shore |
| cg26588076 | 1.02E-07 | SREBF1 | TSS1500 | N_Shore |
| cg26593946 | 7.07E-08 | NR2F2 | TSS200 | S_Shelf |
| cg26599373 | 2.82E-07 | IL17B | TSS1500 | Open Sea |
| cg26600802 | 1.04E-07 | TDRD1 | 1stExon | Island |
| cg26605164 | 1.11E-08 | KAZALD1 | 1stExon | Island |
| cg26613742 | 4.62E-08 | PRKACA | TSS200 | N_Shelf |
| cg26614815 | 1.21E-07 | ART4 | TSS1500 | Open Sea |
| cg26620147 | 1.03E-07 | AVIL | TSS1500 | Open Sea |
| cg26632897 | 5.67E-08 | C1QTNF1 | 1stExon | Open Sea |
| cg26638505 | 9.31E-07 | SRD5A2 | TSS200 | Island |
| cg26643476 | 1.37E-07 | GPR81 | TSS1500 | Open Sea |
| cg26644853 | 9.81E-07 | CX3CL1 | TSS200 | Open Sea |
| cg26648818 | 1.45E-07 | TOX3 | TSS200 | S_Shore |
| cg26651122 | 4.44E-07 | MT2A | TSS1500 | N_Shore |
| cg26654798 | 3.33E-07 | BNC1 | TSS1500 | Island |
| cg26661623 | 4.53E-08 | ASGR2 | TSS1500 | Open Sea |
| cg26670636 | 1.49E-07 | C7orf61 | TSS1500 | Open Sea |
| cg26670875 | 3.16E-09 | MYH7 | TSS1500 | Open Sea |
| cg26674558 | 8.97E-10 | C8orf46 | TSS1500 | Open Sea |
| cg26680502 | 9.65E-08 | PPP1R16B | TSS1500 | N_Shore |
| cg26700277 | 5.58E-07 | ZNF469 | 1stExon | N_Shore |
| cg26703873 | 5.31E-09 | UNG | TSS1500 | N_Shore |
| cg26705561 | 6.93E-07 | SEC31B | TSS200 | S_Shore |
| cg26711820 | 6.92E-07 | MYF6 | 1stExon | N_Shore |
| cg26734350 | 5.92E-07 | GPR56 | TSS1500 | Open Sea |
| cg26738568 | 5.55E-07 | MIR125A | TSS200 | Open Sea |
| cg26740494 | 1.86E-10 | MMP23A | TSS1500 | Island |
| cg26746469 | 2.36E-07 | KIAA0406 | TSS1500 | S_Shore |
| cg26754510 | 1.41E-07 | C6orf57 | TSS1500 | N_Shore |
| cg26758396 | 3.58E-07 | FERMT1 | TSS200 | S_Shore |
| cg26781466 | 1.21E-07 | GCNT2 | 1stExon | Open Sea |
| cg26785230 | 1.50E-07 | FAM96B | TSS1500 | Island |
| cg26785896 | 6.94E-07 | SLC26A10 | TSS1500 | Island |
| cg26797898 | 1.74E-07 | GPR133 | TSS200 | Open Sea |
| cg26810908 | 8.21E-07 | ZP4 | TSS1500 | Open Sea |
| cg26811852 | 3.48E-09 | HNRNPH1 | TSS1500 | S_Shore |
| cg26813693 | 3.68E-07 | ZNF507 | TSS1500 | N_Shore |
| cg26814075 | 3.93E-08 | LEP | TSS200 | Island |
| cg26814712 | 2.44E-07 | SHC4 | 1stExon | Island |
| cg26816748 | 2.60E-07 | CALD1 | TSS1500 | Open Sea |
| cg26826183 | 3.40E-07 | EIF3G | TSS1500 | S_Shore |
| cg26827653 | 1.22E-07 | FEZ1 | TSS1500 | S_Shore |
| cg26832686 | 6.86E-07 | WWP2 | TSS1500 | Open Sea |
| cg26833120 | 9.12E-08 | COL16A1 | 1stExon | Island |
| cg26837372 | 3.79E-07 | STK19 | TSS200 | N_Shore |
| cg26842034 | 9.65E-07 | SCGBL | TSS200 | Open Sea |
| cg26852159 | 4.13E-08 | ASB16 | TSS1500 | Open Sea |
| cg26859016 | 4.76E-07 | IKBKE | TSS1500 | Open Sea |
| cg26862286 | 6.09E-07 | NCL | TSS1500 | S_Shore |
| cg26864526 | 2.13E-07 | HRH1 | TSS1500 | Open Sea |
| cg26872305 | 3.83E-07 | STK39 | TSS1500 | S_Shore |
| cg26877596 | 5.17E-07 | NUAK1 | TSS1500 | S_Shore |
| cg26882204 | 1.43E-07 | CTBS | TSS1500 | S_Shore |
| cg26884581 | 9.14E-08 | PYGM | 1stExon | Open Sea |
| cg26887212 | 3.74E-07 | ZBTB22 | TSS1500 | S_Shore |
| cg26893134 | 2.08E-07 | FRK | 1stExon | Open Sea |
| cg26900724 | 2.28E-08 | DBN1 | TSS1500 | Open Sea |
| cg26908388 | 8.35E-07 | KIFC1 | TSS1500 | N_Shore |
| cg26908876 | 2.81E-09 | KCNQ1OT1 | TSS1500 | Island |
| cg26915370 | 2.46E-07 | CALD1 | TSS1500 | Open Sea |
| cg26917754 | 2.88E-08 | TXLNA | TSS1500 | N_Shore |
| cg26919186 | 2.43E-07 | LOC390858 | TSS1500 | Open Sea |
| cg26919618 | 2.03E-07 | ZBTB22 | TSS1500 | N_Shore |
| cg26923754 | 1.61E-08 | SLC25A34 | 1stExon | Open Sea |
| cg26924127 | 5.50E-07 | RD3 | TSS1500 | Open Sea |
| cg26924223 | 1.82E-07 | ZNF563 | TSS1500 | S_Shore |
| cg26925717 | 7.63E-07 | SLC39A2 | TSS200 | Open Sea |
| cg26927427 | 5.01E-07 | KIFC3 | TSS1500 | N_Shelf |
| cg26935102 | 2.20E-07 | POLR3GL | TSS1500 | S_Shore |
| cg26937038 | 2.89E-07 | S100A9 | TSS1500 | Open Sea |
| cg26939946 | 7.93E-07 | RNF112 | TSS200 | Open Sea |
| cg26946836 | 7.63E-08 | CD207 | TSS1500 | Open Sea |
| cg26950715 | 7.23E-09 | MGMT | TSS1500 | N_Shore |
| cg26952618 | 2.49E-08 | FAM18A | 1stExon | Island |
| cg26958741 | 4.38E-10 | MYL2 | TSS200 | Open Sea |
| cg26970800 | 1.02E-08 | GIF | TSS1500 | Open Sea |
| cg26971420 | 2.33E-07 | ORMDL3 | TSS1500 | S_Shore |
| cg26972554 | 3.84E-07 | FLJ44606 | TSS1500 | S_Shore |
| cg26983326 | 3.42E-07 | ARHGEF5L | TSS1500 | S_Shore |
| cg27013931 | 3.43E-07 | FBRS | TSS1500 | S_Shelf |
| cg27021512 | 3.22E-07 | ESD | TSS1500 | S_Shore |
| cg27026695 | 4.87E-08 | HSPA2 | TSS1500 | N_Shore |
| cg27036341 | 8.28E-07 | GAPDHS | TSS200 | Island |
| cg27063540 | 7.64E-07 | C16orf89 | TSS200 | Open Sea |
| cg27063986 | 1.69E-07 | NDST4 | TSS1500 | Open Sea |
| cg27066989 | 2.31E-07 | GUCY1B2 | TSS200 | Open Sea |
| cg27068143 | 8.46E-08 | HTR2A | TSS200 | Open Sea |
| cg27076046 | 7.25E-07 | DNMT3L | TSS1500 | Open Sea |
| cg27086690 | 5.09E-09 | ZNF280D | TSS1500 | Island |
| cg27091787 | 3.55E-07 | HYAL2 | TSS1500 | S_Shore |
| cg27106909 | 1.41E-07 | YPEL3 | 1stExon | N_Shore |
| cg27112809 | 1.55E-09 | LOC400578 | TSS1500 | Open Sea |
| cg27116510 | 5.67E-07 | IPO5 | TSS200 | Open Sea |
| cg27123522 | 1.36E-08 | GPX5 | TSS1500 | Open Sea |
| cg27124370 | 5.00E-07 | WDR88 | TSS200 | Island |
| cg27131953 | 1.40E-07 | FASTK | TSS1500 | Island |
| cg27147785 | 6.75E-07 | OAS2 | TSS200 | Open Sea |
| cg27149093 | 4.44E-07 | SLC41A2 | 1stExon | Open Sea |
| cg27150412 | 3.22E-09 | PRSS36 | TSS200 | S_Shore |
| cg27169020 | 2.29E-09 | BNC1 | TSS1500 | S_Shore |
| cg27174787 | 1.02E-07 | TPM4 | TSS200 | N_Shore |
| cg27177839 | 2.14E-07 | JAG2 | TSS1500 | Open Sea |
| cg27180443 | 4.93E-07 | SCARB1 | TSS1500 | S_Shore |
| cg27182527 | 7.62E-07 | WIPF3 | TSS200 | Open Sea |
| cg27190239 | 6.62E-07 | CLEC2A | TSS200 | Open Sea |
| cg27195719 | 8.05E-07 | DYNLL2 | TSS1500 | N_Shore |
| cg27196842 | 2.04E-07 | LOC572558 | TSS200 | S_Shore |
| cg27210863 | 1.62E-07 | SH2D4B | TSS1500 | S_Shelf |
| cg27212940 | 6.11E-10 | LOC399959 | TSS1500 | Open Sea |
| cg27222669 | 6.26E-07 | ABCC3 | TSS1500 | N_Shore |
| cg27223827 | 7.53E-07 | FHL2 | 1stExon | Open Sea |
| cg27239280 | 2.38E-07 | CTF1 | TSS1500 | S_Shore |
| cg27243140 | 4.60E-07 | OTOP3 | TSS1500 | N_Shore |
| cg27244773 | 9.58E-08 | ARHGAP27 | 1stExon | Island |
| cg27255239 | 8.43E-07 | RFX8 | TSS1500 | S_Shore |
| cg27263998 | 8.03E-07 | IGF2 | TSS200 | Open Sea |
| cg27270684 | 4.22E-07 | FKBP9L | TSS200 | Open Sea |
| cg27277403 | 5.79E-07 | KMO | TSS1500 | Open Sea |
| cg27291464 | 6.81E-07 | CYP4X1 | TSS1500 | N_Shore |
| cg27299616 | 1.98E-07 | DYNC1H1 | TSS1500 | N_Shore |
| cg27300742 | 3.88E-07 | H19 | TSS1500 | S_Shore |
| cg27303733 | 6.74E-09 | EIF3F | TSS1500 | N_Shore |
| cg27310255 | 1.42E-07 | OLA1 | TSS1500 | S_Shore |
| cg27312338 | 1.73E-08 | KIFC3 | TSS1500 | N_Shelf |
| cg27312626 | 4.79E-07 | KCNJ15 | TSS1500 | Open Sea |
| cg27313021 | 1.13E-07 | GPR37L1 | TSS200 | Open Sea |
| cg27314482 | 1.22E-07 | BTN3A1 | TSS1500 | Open Sea |
| cg27316026 | 8.56E-07 | KRT24 | 1stExon | Open Sea |
| cg27319216 | 3.03E-08 | XKR5 | TSS1500 | S_Shore |
| cg27324619 | 8.88E-07 | INPP5J | TSS200 | Open Sea |
| cg27329371 | 2.93E-07 | ALDH3A1 | TSS200 | S_Shelf |
| cg27334919 | 2.19E-07 | TWIST1 | TSS1500 | S_Shore |
| cg27342837 | 1.16E-07 | TFPI | TSS1500 | Open Sea |
| cg27346545 | 3.12E-07 | RAD21L1 | TSS1500 | N_Shore |
| cg27365208 | 7.71E-07 | ZNF101 | TSS1500 | N_Shore |
| cg27367045 | 7.43E-08 | MGC3771 | TSS200 | N_Shelf |
| cg27372994 | 2.43E-09 | UGT2B11 | TSS200 | Open Sea |
| cg27383865 | 2.70E-08 | SLC38A8 | TSS200 | Open Sea |
| cg27384002 | 4.87E-12 | TMEM200A | TSS200 | Open Sea |
| cg27406618 | 2.71E-10 | FAM170B | 1stExon | S_Shore |
| cg27408345 | 1.19E-10 | LOC282997 | TSS200 | N_Shore |
| cg27417316 | 5.19E-07 | FLJ35024 | TSS1500 | Island |
| cg27420922 | 2.12E-08 | C13orf26 | TSS200 | Open Sea |
| cg27428414 | 3.94E-07 | DHDPSL | TSS200 | Open Sea |
| cg27428856 | 5.58E-10 | TRAF3IP2 | TSS200 | Open Sea |
| cg27447599 | 5.44E-07 | IGFBP1 | TSS200 | N_Shore |
| cg27454842 | 5.90E-09 | PISD | TSS1500 | S_Shore |
| cg27462160 | 3.90E-08 | SPATA2L | TSS1500 | Island |
| cg27464144 | 7.28E-07 | ABCA8 | TSS200 | Open Sea |
| cg27467076 | 4.30E-07 | HTA | TSS1500 | Open Sea |
| cg27492220 | 5.38E-09 | PAGE2B | TSS200 | Open Sea |
| cg27496267 | 3.00E-07 | FZD1 | 1stExon | Island |
| cg27497781 | 9.62E-08 | CHIA | TSS200 | Open Sea |
| cg27498387 | 5.23E-07 | ANK1 | TSS1500 | Island |
| cg27502912 | 9.74E-07 | CPT1B | 1stExon | Island |
| cg27508071 | 6.33E-07 | C7orf26 | TSS1500 | Island |
| cg27514608 | 4.12E-07 | PPARGC1A | TSS1500 | Open Sea |
| cg27518898 | 2.40E-07 | IL10RB | TSS1500 | N_Shore |
| cg27519140 | 4.99E-08 | RPP21 | TSS1500 | N_Shore |
| cg27520549 | 2.95E-08 | SLC22A14 | TSS1500 | Open Sea |
| cg27531553 | 2.86E-08 | TAS1R1 | 1stExon | S_Shore |
| cg27547703 | 1.03E-07 | TDRD1 | TSS200 | Island |
| cg27550918 | 2.19E-07 | SYNPO2L | 1stExon | S_Shelf |
| cg27552218 | 3.25E-09 | LRRC17 | TSS1500 | Open Sea |
| cg27553890 | 1.92E-07 | SSH1 | TSS200 | Open Sea |
| cg27554954 | 2.03E-08 | ANXA2 | TSS1500 | S_Shore |
| cg27555092 | 1.12E-08 | ALPK2 | TSS200 | Open Sea |
| cg27560555 | 2.00E-07 | CNO | TSS1500 | N_Shore |
| cg27560864 | 1.05E-07 | SCN4B | TSS1500 | S_Shore |
| cg27563423 | 9.04E-08 | PALMD | TSS200 | Open Sea |
| cg27565645 | 1.43E-07 | BAX | TSS1500 | N_Shore |
| cg27570256 | 2.25E-08 | LOC100270710 | TSS200 | S_Shelf |
| cg27582124 | 1.78E-07 | GALNT5 | TSS200 | Open Sea |
| cg27582986 | 2.10E-08 | C19orf34 | TSS1500 | N_Shore |
| cg27584762 | 4.69E-07 | CCNH | TSS1500 | S_Shore |
| cg27600794 | 1.48E-10 | PNLIPRP1 | TSS1500 | Open Sea |
| cg27608032 | 1.50E-07 | FASTK | TSS1500 | Island |
| cg27611263 | 4.80E-07 | THUMPD2 | TSS1500 | S_Shore |
| cg27614534 | 2.17E-09 | ALPK2 | TSS1500 | Open Sea |
| cg27616227 | 2.66E-09 | ANGPT1 | TSS200 | Open Sea |
| cg27625491 | 8.21E-07 | CD36 | TSS1500 | Open Sea |
| cg27641317 | 4.90E-08 | FIGLA | TSS1500 | S_Shore |
| cg27650171 | 9.29E-07 | VARS2 | TSS1500 | Island |
| cg27663476 | 4.88E-07 | FAH | TSS1500 | Open Sea |
| cg27664018 | 1.04E-07 | CX3CL1 | TSS1500 | Open Sea |
