# Supplementary material for: Integrated Analysis of Gene Expression and Methylation Data to Identify Potential Biomarkers Related to Atherosclerosis Onset
Source: Oxid Med Cell Longev. 2022 Jul 22;2022:5493051. doi: 10.1155/2022/5493051 (PMC9338736; doi:10.1155/2022/5493051)
Supplement: Supplementary 6 — Table S3: detailed 132 DEGs. [file 5493051.f6.docx]

Table S3. Detailed 132 DEGs.

| logFC | AveExpr | t | P.Value | adj.P.Val | B |  | updown |
| --- | --- | --- | --- | --- | --- | --- | --- |
| FABP4 | 2.454460938 | 5.571159531 | 6.14180231 | 5.31E-08 | 1.74E-05 | 8.215648644 | Up-regulated |
| IGJ | 1.893149375 | 9.890205938 | 4.886670278 | 6.90E-06 | 0.000136912 | 3.632643866 | Up-regulated |
| IGKV1D-33 | 1.8847375 | 8.945788438 | 5.608036985 | 4.39E-07 | 3.36E-05 | 6.223825334 | Up-regulated |
| IGHV3-52 | 1.867600313 | 10.76898578 | 5.486464828 | 7.04E-07 | 4.10E-05 | 5.77793031 | Up-regulated |
| IGKV3D-11 | 1.85104375 | 7.890862813 | 5.194009519 | 2.17E-06 | 6.81E-05 | 4.719664784 | Up-regulated |
| MMP7 | 1.840230625 | 7.951310938 | 4.457119288 | 3.31E-05 | 0.00040518 | 2.164795439 | Up-regulated |
| MMP9 | 1.817804063 | 9.363930781 | 4.911545996 | 6.29E-06 | 0.000129527 | 3.719568465 | Up-regulated |
| CD36 | 1.802205312 | 9.758264531 | 4.880665429 | 7.05E-06 | 0.000138472 | 3.6116902 | Up-regulated |
| IBSP | 1.794982188 | 7.909742344 | 6.584515084 | 8.93E-09 | 1.26E-05 | 9.900075136 | Up-regulated |
| IGKV1D-27 | 1.751979063 | 6.150596094 | 4.717865196 | 1.29E-05 | 0.000205696 | 3.048104023 | Up-regulated |
| IGHV4-59 | 1.739774688 | 8.551526094 | 5.236462049 | 1.84E-06 | 6.26E-05 | 4.871927518 | Up-regulated |
| IGHV3-43 | 1.731189688 | 10.73705828 | 5.32283459 | 1.32E-06 | 5.33E-05 | 5.183179674 | Up-regulated |
| IGKV1OR2-3 | 1.674665313 | 7.156892969 | 5.055109749 | 3.67E-06 | 9.31E-05 | 4.224957171 | Up-regulated |
| IGKC | 1.672011563 | 8.593926094 | 5.350199001 | 1.19E-06 | 5.23E-05 | 5.282187635 | Up-regulated |
| IGKV3D-20 | 1.667174063 | 7.917380469 | 5.118525857 | 2.89E-06 | 8.00E-05 | 4.450145947 | Up-regulated |
| IGKV2D-26 | 1.654989063 | 6.202410781 | 4.866093555 | 7.44E-06 | 0.000143798 | 3.56089017 | Up-regulated |
| IGKV1D-43 | 1.612422813 | 7.858346719 | 5.158106299 | 2.48E-06 | 7.35E-05 | 4.591273487 | Up-regulated |
| DPP4 | 1.610872813 | 7.597484531 | 5.809102921 | 1.99E-07 | 2.60E-05 | 6.96807584 | Up-regulated |
| IGHV3-74 | 1.58550375 | 9.801395 | 5.442582771 | 8.35E-07 | 4.52E-05 | 5.617802677 | Up-regulated |
| MMP12 | 1.574105625 | 9.645882188 | 3.754129816 | 0.000370675 | 0.002614809 | -0.076646193 | Up-regulated |
| TM4SF19 | 1.569003125 | 8.488513125 | 5.04252716 | 3.85E-06 | 9.52E-05 | 4.180415238 | Up-regulated |
| IGHV3-7 | 1.566328333 | 10.9419274 | 5.326174624 | 1.31E-06 | 5.33E-05 | 5.195254268 | Up-regulated |
| IGKV1-13 | 1.553635938 | 9.231854531 | 5.411454138 | 9.42E-07 | 4.74E-05 | 5.50448729 | Up-regulated |
| MME | 1.485000313 | 6.636423906 | 5.288486451 | 1.51E-06 | 5.66E-05 | 5.059171784 | Up-regulated |
| FABP5 | 1.464088906 | 9.090030703 | 4.921956358 | 6.05E-06 | 0.000126964 | 3.756003832 | Up-regulated |
| IGKV1D-16 | 1.442508438 | 9.450381719 | 5.409765771 | 9.48E-07 | 4.74E-05 | 5.498347857 | Up-regulated |
| IGHV3-73 | 1.436434375 | 9.7747525 | 5.12012704 | 2.87E-06 | 7.98E-05 | 4.455846576 | Up-regulated |
| ATP6V0D2 | 1.425070313 | 6.536292344 | 4.484191346 | 3.00E-05 | 0.000374415 | 2.255328722 | Up-regulated |
| HMOX1 | 1.419502188 | 8.488010469 | 5.625004557 | 4.11E-07 | 3.31E-05 | 6.286313782 | Up-regulated |
| MMP8 | 1.417320313 | 5.547593594 | 4.115883473 | 0.000109729 | 0.001009255 | 1.049143429 | Up-regulated |
| IGHV3OR16-7 | 1.40885875 | 9.191685313 | 5.161093255 | 2.46E-06 | 7.31E-05 | 4.601941448 | Up-regulated |
| IL1RN | 1.394489375 | 6.931544688 | 5.526077021 | 6.04E-07 | 3.88E-05 | 5.92285692 | Up-regulated |
| IGKV1D-42 | 1.389536875 | 10.50234188 | 5.323866545 | 1.32E-06 | 5.33E-05 | 5.186910005 | Up-regulated |
| IGKV1OR9-2 | 1.383067188 | 6.309356719 | 5.308036575 | 1.40E-06 | 5.47E-05 | 5.129717172 | Up-regulated |
| IGKV2D-28 | 1.344009375 | 9.36077 | 4.813781192 | 9.04E-06 | 0.000162498 | 3.379086128 | Up-regulated |
| LOC102724453 | 1.303029375 | 9.294813125 | 4.833131463 | 8.41E-06 | 0.000155252 | 3.446231261 | Up-regulated |
| PLA2G7 | 1.299555 | 9.253657813 | 4.680023535 | 1.48E-05 | 0.000227158 | 2.918387231 | Up-regulated |
| IGHV3-35 | 1.2955975 | 9.47369 | 5.465555924 | 7.64E-07 | 4.30E-05 | 5.701576914 | Up-regulated |
| CHI3L1 | 1.28963875 | 9.221257188 | 3.632612596 | 0.000550433 | 0.003565576 | -0.440235791 | Up-regulated |
| CD52 | 1.263186563 | 8.427867344 | 5.049655346 | 3.75E-06 | 9.39E-05 | 4.205643072 | Up-regulated |
| AQP9 | 1.2529975 | 9.121391875 | 4.017623491 | 0.000153603 | 0.001311363 | 0.737225218 | Up-regulated |
| IGKV2D-24 | 1.222532813 | 7.168715156 | 4.278607837 | 6.23E-05 | 0.000653332 | 1.57509958 | Up-regulated |
| PLIN2 | 1.18298 | 9.322913438 | 5.045769825 | 3.80E-06 | 9.46E-05 | 4.191889721 | Up-regulated |
| CCR1 | 1.179454375 | 7.918391875 | 5.740922261 | 2.61E-07 | 2.76E-05 | 6.714805826 | Up-regulated |
| ANPEP | 1.173833125 | 7.817300938 | 5.264605781 | 1.66E-06 | 5.96E-05 | 4.973133585 | Up-regulated |
| SELE | 1.157189375 | 6.310250938 | 4.803337122 | 9.39E-06 | 0.000166388 | 3.342896589 | Up-regulated |
| IGKV3OR2-268 | 1.15536 | 7.38236875 | 4.850996295 | 7.87E-06 | 0.00014908 | 3.508330542 | Up-regulated |
| MMRN1 | 1.154273438 | 5.718870469 | 5.076693155 | 3.38E-06 | 8.82E-05 | 4.301469252 | Up-regulated |
| TDO2 | 1.150757813 | 4.960949844 | 5.371599509 | 1.10E-06 | 5.06E-05 | 5.359747356 | Up-regulated |
| LOC642131 | 1.147373438 | 6.563629219 | 4.889577471 | 6.82E-06 | 0.000135902 | 3.642792517 | Up-regulated |
| ADAMDEC1 | 1.138717813 | 6.736103281 | 3.832569471 | 0.000286111 | 0.002141951 | 0.162077362 | Up-regulated |
| SLC28A3 | 1.13202375 | 6.122057813 | 4.756930878 | 1.11E-05 | 0.000186468 | 3.182534816 | Up-regulated |
| CD163 | 1.130550938 | 10.83334328 | 5.766682762 | 2.36E-07 | 2.70E-05 | 6.810393615 | Up-regulated |
| KMO | 1.1243625 | 6.01270125 | 5.240659184 | 1.81E-06 | 6.23E-05 | 4.88700735 | Up-regulated |
| PLEK | 1.104736563 | 9.405280156 | 5.652165836 | 3.69E-07 | 3.22E-05 | 6.386469744 | Up-regulated |
| CYTIP | 1.099515 | 8.560687813 | 5.096490438 | 3.14E-06 | 8.44E-05 | 4.371768 | Up-regulated |
| MOP-1 | 1.099231875 | 6.961554688 | 3.640266981 | 0.000537007 | 0.003496097 | -0.417561966 | Up-regulated |
| RGS1 | 1.094815 | 8.554976875 | 3.588517775 | 0.000634219 | 0.003987521 | -0.570243245 | Up-regulated |
| IGHV1OR15-1 | 1.077120313 | 5.110156719 | 4.254924196 | 6.77E-05 | 0.000692439 | 1.497843234 | Up-regulated |
| ITGAX | 1.070598438 | 8.703302969 | 4.900019795 | 6.56E-06 | 0.000132761 | 3.679267497 | Up-regulated |
| IGHV3OR16-8 | 1.068210313 | 7.191508906 | 5.008291789 | 4.38E-06 | 0.000102706 | 4.059459789 | Up-regulated |
| NPL | 1.066364063 | 8.649446094 | 5.526379645 | 6.03E-07 | 3.88E-05 | 5.923965475 | Up-regulated |
| MIR8071-1 | 1.064760313 | 7.402199844 | 5.426276748 | 8.89E-07 | 4.67E-05 | 5.558416256 | Up-regulated |
| CXCL10 | 1.051734063 | 7.565688906 | 4.489465891 | 2.95E-05 | 0.000369371 | 2.273000451 | Up-regulated |
| CD180 | 1.048369063 | 7.681407344 | 5.098417284 | 3.12E-06 | 8.40E-05 | 4.378616098 | Up-regulated |
| KYNU | 1.047220625 | 7.449657813 | 4.753720026 | 1.13E-05 | 0.000187915 | 3.171466172 | Up-regulated |
| SEL1L3 | 1.043348438 | 7.956952656 | 5.906582654 | 1.36E-07 | 2.23E-05 | 7.331678531 | Up-regulated |
| VAV3 | 1.026725938 | 7.028524219 | 5.801496857 | 2.05E-07 | 2.63E-05 | 6.939777963 | Up-regulated |
| C7 | 1.024251875 | 5.943591563 | 4.367149893 | 4.56E-05 | 0.000517426 | 1.865984552 | Up-regulated |
| TM4SF18 | 1.0232675 | 6.987580938 | 5.464108439 | 7.68E-07 | 4.30E-05 | 5.696294854 | Up-regulated |
| EMCN | 1.01551375 | 6.61350125 | 4.978811298 | 4.89E-06 | 0.000109102 | 3.955584494 | Up-regulated |
| CR1 | 1.011271875 | 7.64321625 | 4.356703078 | 4.73E-05 | 0.000531859 | 1.831497158 | Up-regulated |
| DCSTAMP | 1.005814063 | 5.575719531 | 5.558096078 | 5.33E-07 | 3.73E-05 | 6.040260363 | Up-regulated |
| PRDM1 | 1.005105938 | 7.457313594 | 5.787842456 | 2.17E-07 | 2.66E-05 | 6.889004877 | Up-regulated |
| C2 | 1.002994167 | 8.31613125 | 5.342825416 | 1.23E-06 | 5.24E-05 | 5.255490604 | Up-regulated |
| ACP5 | 1.001420313 | 8.354723906 | 4.539075997 | 2.47E-05 | 0.000326913 | 2.439730586 | Up-regulated |
| FREM1 | -1.005310625 | 6.781046875 | -4.972983079 | 5.00E-06 | 0.00011059 | 3.93507975 | Down-regulated |
| SLC2A12 | -1.007498125 | 5.962895938 | -5.854886149 | 1.66E-07 | 2.52E-05 | 7.138635342 | Down-regulated |
| MYOM1 | -1.009213438 | 7.292944531 | -5.372189879 | 1.10E-06 | 5.06E-05 | 5.361888575 | Down-regulated |
| FRK | -1.014270938 | 7.816624531 | -4.59551981 | 2.01E-05 | 0.000282281 | 2.630546594 | Down-regulated |
| GRIA1 | -1.025623125 | 7.69117 | -4.697359803 | 1.39E-05 | 0.000217399 | 2.97775205 | Down-regulated |
| TTLL7 | -1.026067188 | 9.218687344 | -5.393006138 | 1.01E-06 | 4.92E-05 | 5.43744181 | Down-regulated |
| NEXN | -1.02671125 | 9.910222188 | -5.947293745 | 1.15E-07 | 2.10E-05 | 7.484028794 | Down-regulated |
| TCEAL2 | -1.031026563 | 8.255096406 | -5.881618558 | 1.50E-07 | 2.39E-05 | 7.238399349 | Down-regulated |
| THRB | -1.036804375 | 8.209507188 | -5.120056262 | 2.87E-06 | 7.98E-05 | 4.455594573 | Down-regulated |
| NLGN1 | -1.0380175 | 6.134227188 | -5.453442549 | 8.00E-07 | 4.41E-05 | 5.657388705 | Down-regulated |
| SBSPON | -1.04225625 | 7.780391875 | -5.342838727 | 1.23E-06 | 5.24E-05 | 5.255538786 | Down-regulated |
| MPP6 | -1.057385 | 7.451604375 | -6.904210668 | 2.43E-09 | 1.13E-05 | 11.12879068 | Down-regulated |
| PLN | -1.061111875 | 10.74383531 | -5.202825734 | 2.10E-06 | 6.75E-05 | 4.751245671 | Down-regulated |
| NPNT | -1.065333438 | 9.491100469 | -5.55104664 | 5.48E-07 | 3.75E-05 | 6.014392881 | Down-regulated |
| LPHN3 | -1.066818438 | 7.243796719 | -4.628353298 | 1.78E-05 | 0.000259292 | 2.742081461 | Down-regulated |
| PRUNE2 | -1.070696094 | 9.179668672 | -5.770111742 | 2.32E-07 | 2.69E-05 | 6.823126958 | Down-regulated |
| PDZRN3 | -1.071817813 | 8.116894844 | -6.094346956 | 6.42E-08 | 1.86E-05 | 8.036609707 | Down-regulated |
| ACTC1 | -1.07237375 | 8.756990313 | -4.715351733 | 1.30E-05 | 0.000206871 | 3.039472752 | Down-regulated |
| NPR1 | -1.076765625 | 7.211579688 | -5.251330302 | 1.74E-06 | 6.08E-05 | 4.925368433 | Down-regulated |
| GRIA2 | -1.089658125 | 8.229319063 | -4.112403182 | 0.000111052 | 0.001018207 | 1.038020919 | Down-regulated |
| SEMA3D | -1.092117188 | 6.590017969 | -4.902274132 | 6.51E-06 | 0.00013188 | 3.687146412 | Down-regulated |
| SNORD116-21 | -1.098605938 | 7.970831094 | -4.702092235 | 1.36E-05 | 0.000214265 | 2.993975605 | Down-regulated |
| NEGR1 | -1.100617188 | 7.792942031 | -6.358357374 | 2.23E-08 | 1.42E-05 | 9.036639802 | Down-regulated |
| PLD5 | -1.111381563 | 6.282228281 | -5.826184154 | 1.86E-07 | 2.58E-05 | 7.031664711 | Down-regulated |
| PCDH20 | -1.1142475 | 6.014171875 | -5.977619278 | 1.02E-07 | 2.05E-05 | 7.59769647 | Down-regulated |
| UNC13C | -1.121798438 | 5.503821406 | -5.580767925 | 4.88E-07 | 3.53E-05 | 6.1235271 | Down-regulated |
| SLC22A3 | -1.128239375 | 8.969953125 | -6.390722117 | 1.96E-08 | 1.38E-05 | 9.159853816 | Down-regulated |
| ANGPTL1 | -1.133249688 | 6.118383594 | -5.756057984 | 2.46E-07 | 2.73E-05 | 6.770953393 | Down-regulated |
| CNN1 | -1.140570938 | 9.062172656 | -6.50187113 | 1.25E-08 | 1.26E-05 | 9.583915782 | Down-regulated |
| TMEM56 | -1.143576563 | 7.253480469 | -6.202809613 | 4.16E-08 | 1.73E-05 | 8.446296118 | Down-regulated |
| LGI1 | -1.156440938 | 5.174031406 | -5.41861982 | 9.16E-07 | 4.70E-05 | 5.530551568 | Down-regulated |
| MRAP2 | -1.158247188 | 9.253291094 | -5.428434596 | 8.82E-07 | 4.65E-05 | 5.566271505 | Down-regulated |
| SCRG1 | -1.160169688 | 7.099733281 | -5.687594389 | 3.21E-07 | 3.02E-05 | 6.517339999 | Down-regulated |
| FIBIN | -1.161312188 | 9.241323906 | -6.171023183 | 4.73E-08 | 1.73E-05 | 8.326056667 | Down-regulated |
| IL31RA | -1.175403438 | 5.550028594 | -6.452364595 | 1.52E-08 | 1.37E-05 | 9.394866239 | Down-regulated |
| ITLN1 | -1.182176875 | 6.441940625 | -3.598544356 | 0.000614163 | 0.003874647 | -0.540773046 | Down-regulated |
| PLCB4 | -1.194507813 | 6.370274531 | -5.865862494 | 1.59E-07 | 2.43E-05 | 7.179583027 | Down-regulated |
| ACADL | -1.197929375 | 7.002552188 | -5.911917249 | 1.33E-07 | 2.23E-05 | 7.351625561 | Down-regulated |
| PDE8B | -1.232035313 | 6.816950156 | -5.512140774 | 6.37E-07 | 3.96E-05 | 5.871828731 | Down-regulated |
| LINC00670 | -1.236636875 | 6.500943438 | -5.541655289 | 5.68E-07 | 3.75E-05 | 5.979948934 | Down-regulated |
| RYR2 | -1.241439688 | 7.064771719 | -5.549895203 | 5.50E-07 | 3.75E-05 | 6.010168794 | Down-regulated |
| NPR3 | -1.261192344 | 7.598900234 | -5.691493722 | 3.17E-07 | 3.01E-05 | 6.531759442 | Down-regulated |
| LRRN1 | -1.272334688 | 5.443577656 | -5.96181792 | 1.09E-07 | 2.09E-05 | 7.538449842 | Down-regulated |
| NPY1R | -1.27438375 | 6.75722 | -5.261742393 | 1.67E-06 | 5.97E-05 | 4.962827217 | Down-regulated |
| CNTN3 | -1.361736563 | 6.962632344 | -5.760228916 | 2.42E-07 | 2.71E-05 | 6.786433711 | Down-regulated |
| PCDH11X | -1.366003281 | 6.149847422 | -6.051467129 | 7.62E-08 | 1.94E-05 | 7.875125744 | Down-regulated |
| RPS6KA6 | -1.3692 | 6.725065625 | -5.947327813 | 1.15E-07 | 2.10E-05 | 7.484156401 | Down-regulated |
| ATRNL1 | -1.4374975 | 8.247135313 | -5.177874154 | 2.31E-06 | 7.04E-05 | 4.661920407 | Down-regulated |
| CARTPT | -1.470486875 | 6.49031 | -3.450712244 | 0.000981312 | 0.005658681 | -0.969696282 | Down-regulated |
| HAND2-AS1 | -1.504355313 | 6.310014219 | -5.931125638 | 1.23E-07 | 2.16E-05 | 7.423490013 | Down-regulated |
| FHL5 | -1.564652188 | 8.027710781 | -6.552992831 | 1.01E-08 | 1.26E-05 | 9.779403832 | Down-regulated |
| MYOCD | -1.62844875 | 8.156160625 | -6.139981097 | 5.35E-08 | 1.74E-05 | 8.208771467 | Down-regulated |
| CASQ2 | -1.667664063 | 8.549835781 | -6.399743552 | 1.89E-08 | 1.38E-05 | 9.194220878 | Down-regulated |
| CNTN4 | -1.792331875 | 7.343392188 | -6.84701888 | 3.07E-09 | 1.19E-05 | 10.90840099 | Down-regulated |
| TPH1 | -1.88662625 | 8.72227125 | -5.505213307 | 6.55E-07 | 4.01E-05 | 5.846479813 | Down-regulated |
| CNTN1 | -1.911032188 | 6.989837656 | -6.317621477 | 2.63E-08 | 1.42E-05 | 8.881736849 | Down-regulated |
