# Supplementary material for: Integrated Analysis of Gene Expression and Methylation Data to Identify Potential Biomarkers Related to Atherosclerosis Onset
Source: Oxid Med Cell Longev. 2022 Jul 22;2022:5493051. doi: 10.1155/2022/5493051 (PMC9338736; doi:10.1155/2022/5493051)
Supplement: Supplementary 7 — Table S4: the detailed functional enrichment results of the 2860 genes. [file 5493051.f7.docx]

**Table S4** The detailed functional enrichment results of the 2860 genes.

| ID | Description | GeneRatio | BgRatio | pvalue | p.adjust | qvalue | geneID | Count |
| --- | --- | --- | --- | --- | --- | --- | --- | --- |
| GO:0006936 | muscle contraction | 86/2213 | 352/17653 | 5.08E-10 | 2.99E-06 | 2.66E-06 | OBSL1/GLRA1/EDNRA/ALDOA/MYLK2/NPY2R/MYL2/MYH7/ATP1A2/P2RX1/PRKACA/CHRNE/CHRM1/CHGA/MYL10/SSPN/ACTG2/CALD1/FLNA/ANK2/TRIM63/ACTA2/RYR2/PDE4B/MYOM2/DAPK3/PRKG1/CTNNA3/ADRA2C/CNN1/SMPX/UCN/DMPK/TCAP/ITGA2/CALCA/TBXA2R/MYH4/CASQ1/MYL3/ACTN2/MYH14/MYL9/MYOM3/DYSF/ADRA1A/C12orf57/GJA5/TNNI1/KCNA5/MYOCD/TPM4/HTR2A/SCN2B/MYOM1/SORBS1/SLMAP/FXYD1/GSTM2/DRD2/HSPB6/PLCE1/CACNA2D1/TNNI2/RCSD1/MYLK/SGCA/KCNE3/CTGF/PABPN1/EDN3/CALM1/CSRP3/TNNT2/DES/P2RX6/CHRNB4/CALCRL/NEDD4L/TNNT3/SLC9A1/ANKRD2/GJA1/MYBPC1/VEGFB/SCN4B | 86 |
| GO:0050878 | regulation of body fluid levels | 110/2213 | 499/17653 | 1.41E-09 | 4.14E-06 | 3.68E-06 | APLNR/OAS2/F2RL2/DGKG/AQP1/ADCY4/GNA15/SCUBE1/VEGFA/HEG1/HIST1H3A/P2RX1/PRKACA/STMN1/CHRM1/PROC/SERPIND1/HIST1H3C/FLNA/GP9/F10/PLEK/TSPAN8/AQP5/C1QTNF1/HIST1H3E/GP1BB/ALOX12/S100A9/FAP/CLIC1/METAP1/ZFPM1/PRKG1/ADRA2C/UCN/AKR1B1/CDO1/GBA/PRLR/ITGA2/TP63/F2RL3/TBXA2R/DGKZ/AKT1/ADAMTS18/MICAL1/SERPINF2/MYL9/PAFAH2/SLC22A2/LCK/HSPB1/GJA5/MAPK3/ADCY5/SELP/VKORC1/CFTR/XDH/LCP2/MFN2/COL1A2/CSRP1/PDGFA/GP1BA/PLAT/NOS3/DRD2/ADORA2A/SCNN1A/KRT16/DDR1/TRPV5/AK3/ENTPD2/APOE/CEL/MERTK/ADCY6/SERPING1/CSN3/FLI1/RHOB/HBE1/PRKAR2A/AQP4/PLAUR/PAM/PDPN/CD36/GATA5/P2RX6/UTS2R/PRKAR2B/PIK3R6/TFPI/COL3A1/GJA1/GUCA2B/GNA13/AP3B1/ITGA2B/SCT/PTPRO/PF4V1/HYAL2/STK39/ANXA2 | 110 |
| GO:0003012 | muscle system process | 100/2213 | 450/17653 | 5.09E-09 | 9.97E-06 | 8.88E-06 | OBSL1/GLRA1/MIR25/MIR23B/EDNRA/IGFBP5/ALDOA/MYLK2/NPY2R/MYL2/MYH7/ATP1A2/P2RX1/HAND2/PRKACA/CHRNE/CHRM1/CHGA/MYL10/SSPN/ACTG2/CALD1/PDLIM5/MIR199A1/FLNA/ANK2/MAP2K4/TRIM63/ACTA2/RYR2/PDE4B/MYOM2/DAPK3/PRKG1/CTNNA3/ADRA2C/CNN1/SMPX/UCN/DMPK/TCAP/ITGA2/CALCA/TBXA2R/MYH4/CASQ1/MYL3/ERRFI1/ACTN2/MYH14/MYL9/MYOM3/DYSF/LEP/ADRA1A/C12orf57/GJA5/TNNI1/KCNA5/MYOCD/TPM4/HTR2A/SCN2B/MYOM1/SORBS1/MIR195/SLMAP/FXYD1/NOS3/GSTM2/DRD2/HSPB6/PLCE1/CACNA2D1/TNNI2/RCSD1/MYLK/SGCA/KCNE3/CTGF/PABPN1/EDN3/CALM1/IL1B/CSRP3/TNNT2/DES/P2RX6/CHRNB4/CALCRL/NEDD4L/MYOC/TNNT3/SLC9A1/ANKRD2/GJA1/MYBPC1/VEGFB/PPARGC1A/SCN4B | 100 |
| GO:0060537 | muscle tissue development | 86/2213 | 396/17653 | 1.76E-07 | 0.000258497 | 0.00023019 | OBSL1/FHL2/COL11A1/MIR25/HDAC9/MIR23B/KCNAB1/IGFBP5/HNRNPU/SMYD1/MYLK2/MYL2/MYH7/FOXC2/VEGFA/HEG1/FOXP2/BCL9L/PDLIM5/MIR199A1/OSR1/POPDC2/MAP2K4/RARA/NR2F2/SVIL/ASB2/RYR2/MYOM2/ZFPM1/PAX7/BVES/NUPR1/TCAP/TWIST1/FRS2/TP63/SOX6/CASQ1/MYL3/FLOT1/HOXD10/STRA6/HEYL/ACTN2/MYH14/MYOM3/SOX15/BMP5/S100B/JPH2/GJA5/KEL/BMP4/TNNI1/MYOCD/SIX4/MYOM1/FGF20/MIR195/FGF2/CAV2/CDON/KCNK2/ALDH1A2/GTF3C5/TBX5/HOXD9/MIR548C/MYLK/NKX2-6/HIVEP3/MYF6/SOX11/CSRP3/TNNT2/RB1/NOX4/SLC9A1/ANKRD2/COL3A1/GJA1/NEXN/PITX1/PKD2/PPARGC1A | 86 |
| GO:0001501 | skeletal system development | 101/2213 | 493/17653 | 3.09E-07 | 0.000307013 | 0.000273394 | WFIKKN2/HOXD8/COL11A1/CMKLR1/HOXA3/HOXA7/PKDCC/HOXA5/SMAD9/PLXNB1/FOXC2/CER1/HAND2/SFRP2/HOXB2/DLX2/JAG2/GPLD1/COL5A2/HOXC4/OSR1/RARA/SNAI1/NDST1/SFRP1/IGF2/CSGALNACT1/ZFPM1/PTH1R/PAX7/RUNX3/LRP5/HOXA6/EXTL1/PAX1/ALX4/TWIST1/RASSF2/TP63/LOXL2/GDF6/SOX6/PRELP/MTHFD1L/HOXD10/CLEC3A/BMP5/NPR2/CHI3L1/LEP/MKS1/WNT9B/GJA5/MAPK3/TGFB3/LRRC17/VKORC1/BMP4/TGFBI/ZBTB16/SIX4/COL1A2/SPNS2/COL2A1/FGF2/HAPLN4/TULP3/MMP14/HES5/DCHS1/MDFI/CYR61/TJP2/HOXC8/HOXD4/HOXD9/TNFSF11/EXT1/FLI1/NPPC/PPARGC1B/HOXA10/CTGF/SOX11/DSCAML1/MUSTN1/SFRP4/SIX2/MMP9/MYOC/FGF6/COL3A1/COL13A1/DYM/PITX1/FOXN3/WNT1/BMP8B/HYAL2/SRD5A2/ANXA2 | 101 |
| GO:0043062 | extracellular structure organization | 85/2213 | 395/17653 | 3.13E-07 | 0.000307013 | 0.000273394 | LOXL1/COL11A1/MFAP4/ABI3BP/SPOCK2/CAPN2/DDR2/TNXB/SCUBE1/FOXC2/TNF/ITGA7/PRKACA/CETP/LIPC/GFAP/PXDN/SFRP2/ITGAX/ELANE/COL18A1/ADAM12/COL5A2/TTR/APOB/FAP/CSGALNACT1/PLA2G7/NR2E1/LAMB3/LOXL4/ITGA2/LOXL2/B4GALT1/FLOT1/LRP1/SERPINF2/ELF3/ELN/KAZALD1/PHLDB1/ABCG1/VIT/FERMT1/TGFBI/DPT/COL6A2/ETS1/COL1A2/ITGAM/COL2A1/ITGA6/IBSP/PDGFA/FGF2/OPTC/CYP1B1/MMP14/DDR1/POMT1/CYR61/APOE/GPIHBP1/FBLN5/ITGA10/EGFLAM/LAMA3/MATN4/CTGF/PDPN/CD36/APOA1/SMOC2/LAMA2/CTSK/ABL1/MMP9/COL3A1/COL13A1/ITGA2B/CD47/COL6A3/COL16A1/SCARB1/ANXA2 | 85 |
| GO:0048568 | embryonic organ development | 90/2213 | 428/17653 | 4.16E-07 | 0.000307751 | 0.00027405 | TMIE/COL11A1/EPN1/HOXA3/HOXA7/PKDCC/BCR/HOXA5/TTPA/CLRN1/FOXC2/VEGFA/TNF/HAND2/CHRNA9/USH1C/PAX6/HOXB2/DLX2/PCDH12/CSF2/HOXC4/OSR1/RARA/NR2F2/SNAI1/NDST1/ASCL2/KRT8/RYR2/KDM2B/KRT19/IGF2/MBD3/FOLR1/PLCD1/ZFPM1/NAGLU/BBS5/HOXA6/ABR/HMX2/ALX4/TCAP/TWIST1/FRS2/AKT1/MTHFD1L/HOXD10/STRA6/MYO7A/BMP5/KCNQ4/MKS1/WNT9B/GJA5/MAPK3/TH/TGFB3/ZIC1/BMP4/TPO/ID3/SIX4/VANGL2/COL2A1/MYO15A/PDGFA/TCF7/NES/TULP3/BBS7/MMP14/ALDH1A2/MDFI/CYR61/HOXD4/HOXD9/ERCC1/NKX2-6/EGFR/SETDB2/SOX11/DSCAML1/SIX2/SOX18/GJA1/WNT1/SCT/PKD2 | 90 |
| GO:0051146 | striated muscle cell differentiation | 65/2213 | 279/17653 | 4.19E-07 | 0.000307751 | 0.00027405 | WFIKKN2/OBSL1/FHL2/HDAC9/MIR23B/IGFBP5/LDB3/HNRNPU/SMYD1/CAPN2/MYLK2/MYL2/VEGFA/MAMSTR/ADAM12/PDLIM5/MIR199A1/POPDC2/MAP2K4/RARA/CD53/KRT8/KRT19/MYOM2/BVES/MYOZ3/DMPK/TCAP/FRS2/MYEF2/SOX6/CASQ1/AKT1/OBSCN/FLOT1/ACTN2/MYOM3/FLNC/MYOZ2/KEL/BMP4/MYOCD/SIX4/MYOM1/AVPR1A/MIR195/CAV2/CDON/MMP14/RBM38/TBX5/NKX2-6/MYF6/CSRP3/TNNT2/RB1/NOX4/TNNT3/SLC9A1/ANKRD2/KRAS/SORT1/NEXN/MYBPC1/WNT1 | 65 |
| GO:0003018 | vascular process in circulatory system | 44/2213 | 164/17653 | 5.60E-07 | 0.00035967 | 0.000320285 | HTR1B/MIR23B/EDNRA/BCR/WDR35/FOXC2/ATP1A2/P2RX1/CHRM1/CHGA/ANGPT1/PRKG1/ABR/ADRA2C/UCN/CALCA/TBXA2R/AKT1/NOS2/SERPINF2/HRH2/EPHX2/LEP/ADRA1A/KCNMB1/GJA5/HRH1/NTS/KEL/KCNA5/PDE2A/HTR2A/DDAH1/AVPR1A/GCLC/NOS3/ADORA2A/APOE/ADCY6/NPPC/EGFR/EDN3/UTS2R/GJA1 | 44 |
| GO:0001503 | ossification | 80/2213 | 371/17653 | 6.34E-07 | 0.00035967 | 0.000320285 | FHL2/COL11A1/IGFBP5/MN1/HNRNPU/PKDCC/DDR2/ECM1/PLXNB1/FOXC2/CER1/FZD1/TNF/HAND2/PRKACA/SFRP2/SOST/GPLD1/COL5A2/OSR1/LRP4/SNAI1/SFRP1/IGF2/CLIC1/CSGALNACT1/PTH1R/RUNX3/LRP5/TWIST1/CALCA/RASSF2/TP63/AKT1/PDLIM7/CLEC3A/BMP5/OSTN/NPR2/LEP/RRBP1/KAZALD1/TPH1/MAPK3/TGFB3/BMP4/FZD9/ZBTB16/ID3/TPM4/CLEC3B/COL2A1/IBSP/DUOX2/DHH/SMAD6/MMP14/TOB1/DCHS1/CYR61/TNFSF11/EXT1/NPPC/EGFR/PPARGC1B/GPNMB/CTGF/RPS15/SOX11/SIX2/CTSK/MMP9/MYOC/SORT1/COL13A1/GJA1/BMP8B/IGSF10/HDAC7/ASGR2 | 80 |
| GO:0006937 | regulation of muscle contraction | 44/2213 | 165/17653 | 6.73E-07 | 0.00035967 | 0.000320285 | MYLK2/NPY2R/MYL2/MYH7/ATP1A2/P2RX1/PRKACA/CHRM1/CHGA/FLNA/ANK2/RYR2/PDE4B/DAPK3/PRKG1/CTNNA3/ADRA2C/CNN1/UCN/DMPK/ITGA2/CALCA/TBXA2R/CASQ1/MYL3/MYL9/ADRA1A/C12orf57/GJA5/TNNI1/MYOCD/GSTM2/HSPB6/PLCE1/TNNI2/RANGRF/KCNE3/CTGF/CALM1/TNNT2/CHRNB4/CALCRL/TNNT3/SLC9A1 | 44 |
| GO:0035150 | regulation of tube size | 39/2213 | 140/17653 | 8.66E-07 | 0.000424671 | 0.000378168 | HTR1B/EDNRA/WDR35/FOXC2/ATP1A2/P2RX1/CHRM1/CHGA/PRKG1/ADRA2C/UCN/CALCA/TBXA2R/AKT1/NOS2/SERPINF2/HRH2/EPHX2/LEP/ADRA1A/KCNMB1/WNT9B/GJA5/HRH1/NTS/KEL/KCNA5/HTR2A/AVPR1A/GCLC/NOS3/ADORA2A/APOE/ADCY6/NPPC/EGFR/EDN3/UTS2R/GJA1 | 39 |
| GO:0006941 | striated muscle contraction | 45/2213 | 173/17653 | 1.07E-06 | 0.000484489 | 0.000431435 | OBSL1/ALDOA/MYLK2/MYL2/MYH7/ATP1A2/PRKACA/CHGA/FLNA/ANK2/RYR2/PDE4B/MYOM2/CTNNA3/SMPX/UCN/DMPK/TCAP/CASQ1/MYL3/MYH14/MYOM3/ADRA1A/C12orf57/GJA5/TNNI1/KCNA5/SCN2B/MYOM1/GSTM2/CACNA2D1/TNNI2/RCSD1/KCNE3/CTGF/CALM1/CSRP3/TNNT2/NEDD4L/TNNT3/SLC9A1/GJA1/MYBPC1/VEGFB/SCN4B | 45 |
| GO:0030198 | extracellular matrix organization | 74/2213 | 341/17653 | 1.29E-06 | 0.000540274 | 0.000481111 | LOXL1/COL11A1/MFAP4/ABI3BP/SPOCK2/CAPN2/DDR2/TNXB/SCUBE1/FOXC2/TNF/ITGA7/GFAP/PXDN/SFRP2/ITGAX/ELANE/COL18A1/ADAM12/COL5A2/TTR/FAP/CSGALNACT1/NR2E1/LAMB3/LOXL4/ITGA2/LOXL2/B4GALT1/FLOT1/LRP1/SERPINF2/ELF3/ELN/KAZALD1/PHLDB1/VIT/FERMT1/TGFBI/DPT/COL6A2/ETS1/COL1A2/ITGAM/COL2A1/ITGA6/IBSP/PDGFA/FGF2/OPTC/CYP1B1/MMP14/DDR1/POMT1/CYR61/FBLN5/ITGA10/EGFLAM/LAMA3/MATN4/CTGF/PDPN/SMOC2/LAMA2/CTSK/ABL1/MMP9/COL3A1/COL13A1/ITGA2B/CD47/COL6A3/COL16A1/ANXA2 | 74 |
| GO:0050880 | regulation of blood vessel size | 38/2213 | 139/17653 | 1.97E-06 | 0.000758752 | 0.000675665 | HTR1B/EDNRA/WDR35/FOXC2/ATP1A2/P2RX1/CHRM1/CHGA/PRKG1/ADRA2C/UCN/CALCA/TBXA2R/AKT1/NOS2/SERPINF2/HRH2/EPHX2/LEP/ADRA1A/KCNMB1/GJA5/HRH1/NTS/KEL/KCNA5/HTR2A/AVPR1A/GCLC/NOS3/ADORA2A/APOE/ADCY6/NPPC/EGFR/EDN3/UTS2R/GJA1 | 38 |
| GO:0030048 | actin filament-based movement | 37/2213 | 134/17653 | 2.06E-06 | 0.000758752 | 0.000675665 | MYO1C/MYLK2/MYL2/MYH7/ATP1A2/FLNA/ANK2/RYR2/PDE4B/CTNNA3/TCAP/MYH4/MYL3/ACTN2/MYH14/MYO7A/FRMD6/GJA5/TNNI1/KCNA5/TPM4/SCN2B/WIPF3/CACNA2D1/MYO1B/TNNI2/KCNE3/PDPN/TNNT2/DES/NEDD4L/TNNT3/SLC9A1/MYH10/GJA1/MYBPC1/SCN4B | 37 |
| GO:1903522 | regulation of blood circulation | 64/2213 | 290/17653 | 3.66E-06 | 0.001264959 | 0.00112644 | HSPB7/MYL2/MYH7/ATP1A2/P2RX1/PRKACA/CHRM1/CHGA/FLNA/ANK2/POPDC2/RYR2/PDE4B/FXYD6/CTNNA3/ADRA2C/BVES/UCN/DMPK/CALCA/TBXA2R/CASQ1/MYL3/AKT1/KCNK3/ATP1A4/HRH2/NPR2/LEP/ADRA1A/GJA5/TH/HRH1/AHCYL1/KCNA5/KCNJ14/HTR2A/SCN2B/AVPR1A/CACNA1B/SREBF1/FXYD1/NOS3/GSTM2/DRD2/CACNA2D1/NPPC/RANGRF/EGFR/KCNE3/CTGF/EDN3/CALM1/KCNIP4/CSRP3/TNNT2/DES/UTS2R/KCNK6/SLC9A1/KCNIP3/GJA1/SLC8A2/SCN4B | 64 |
| GO:0007599 | hemostasis | 73/2213 | 346/17653 | 4.44E-06 | 0.001452501 | 0.001293446 | F2RL2/DGKG/GNA15/SCUBE1/HIST1H3A/P2RX1/PRKACA/PROC/SERPIND1/HIST1H3C/FLNA/GP9/F10/PLEK/TSPAN8/C1QTNF1/HIST1H3E/GP1BB/ALOX12/S100A9/FAP/CLIC1/METAP1/ZFPM1/PRKG1/ADRA2C/ITGA2/F2RL3/TBXA2R/DGKZ/AKT1/ADAMTS18/MICAL1/SERPINF2/MYL9/PAFAH2/LCK/HSPB1/MAPK3/SELP/VKORC1/LCP2/MFN2/COL1A2/CSRP1/PDGFA/GP1BA/PLAT/NOS3/ADORA2A/AK3/ENTPD2/APOE/MERTK/SERPING1/FLI1/RHOB/HBE1/PRKAR2A/PLAUR/PDPN/CD36/GATA5/P2RX6/PRKAR2B/PIK3R6/TFPI/COL3A1/GNA13/AP3B1/ITGA2B/PF4V1/ANXA2 | 73 |
| GO:0007596 | blood coagulation | 72/2213 | 341/17653 | 5.01E-06 | 0.001521262 | 0.001354677 | F2RL2/DGKG/GNA15/SCUBE1/HIST1H3A/P2RX1/PRKACA/PROC/SERPIND1/HIST1H3C/FLNA/GP9/F10/PLEK/TSPAN8/C1QTNF1/HIST1H3E/GP1BB/ALOX12/S100A9/FAP/CLIC1/METAP1/ZFPM1/PRKG1/ADRA2C/ITGA2/F2RL3/TBXA2R/DGKZ/AKT1/ADAMTS18/MICAL1/SERPINF2/MYL9/PAFAH2/LCK/HSPB1/MAPK3/SELP/VKORC1/LCP2/MFN2/COL1A2/CSRP1/PDGFA/GP1BA/PLAT/NOS3/ADORA2A/AK3/ENTPD2/APOE/MERTK/SERPING1/RHOB/HBE1/PRKAR2A/PLAUR/PDPN/CD36/GATA5/P2RX6/PRKAR2B/PIK3R6/TFPI/COL3A1/GNA13/AP3B1/ITGA2B/PF4V1/ANXA2 | 72 |
| GO:0062013 | positive regulation of small molecule metabolic process | 42/2213 | 166/17653 | 5.17E-06 | 0.001521262 | 0.001354677 | RCVRN/RAMP1/TNF/TSHR/CHGA/EPM2AIP1/GPLD1/GPHA2/IGF2/PTH1R/TWIST1/C1QTNF2/BEND3/AKT1/NOS2/OSTN/HRH1/ABCG1/CD244/ADNP/HTR2A/AVPR1A/SORBS1/EGF/PMAIP1/SREBF1/NOS3/APOE/COMT/NPPC/PPARGC1B/CALM1/IL1B/APOA1/INSR/GPD1/GUCA2B/IRS2/LDLRAP1/SCT/GAPDHS/PPARGC1A | 42 |
| GO:0014706 | striated muscle tissue development | 78/2213 | 379/17653 | 5.63E-06 | 0.001560688 | 0.001389785 | OBSL1/FHL2/COL11A1/MIR25/HDAC9/MIR23B/KCNAB1/HNRNPU/SMYD1/MYLK2/MYL2/MYH7/FOXC2/VEGFA/HEG1/FOXP2/BCL9L/PDLIM5/MIR199A1/POPDC2/MAP2K4/RARA/NR2F2/SVIL/ASB2/RYR2/MYOM2/ZFPM1/PAX7/BVES/NUPR1/TCAP/TWIST1/FRS2/SOX6/CASQ1/MYL3/FLOT1/HOXD10/HEYL/ACTN2/MYH14/MYOM3/SOX15/BMP5/S100B/JPH2/GJA5/KEL/BMP4/TNNI1/MYOCD/SIX4/MYOM1/FGF20/MIR195/FGF2/CAV2/CDON/KCNK2/ALDH1A2/GTF3C5/TBX5/HOXD9/MIR548C/NKX2-6/HIVEP3/MYF6/SOX11/CSRP3/TNNT2/RB1/NOX4/SLC9A1/ANKRD2/GJA1/NEXN/PITX1 | 78 |
| GO:0007517 | muscle organ development | 81/2213 | 398/17653 | 5.84E-06 | 0.001560688 | 0.001389785 | COL11A1/MIR25/HDAC9/MIR23B/KCNAB1/SMYD1/MAPK12/MYLK2/MYL2/MYH7/FOXC2/HEG1/FZD1/ITGA7/FOXP2/BCL9L/MIR199A1/POPDC2/NR2F2/SVIL/TCF23/ASB2/RYR2/MYOM2/ZFPM1/USP2/PAX7/BVES/NUPR1/ALX4/TCAP/TWIST1/FRS2/SOX6/CASQ1/MYL3/FLOT1/HOXD10/STRA6/HEYL/CTF1/MYH14/MYOM3/SOX15/S100B/JPH2/KEL/BMP4/TNNI1/MYOCD/ID3/SIX4/VANGL2/MYOM1/FGF20/MIR195/FGF2/CAV2/TAGLN/CDON/KCNK2/GTF3C5/SPEG/CNTFR/TBX5/HOXD9/MIR548C/MYLK/SGCA/HIVEP3/MYF6/SOX11/CSRP3/TNNT2/RB1/LAMA2/ANKRD2/COL3A1/GJA1/PITX1/COL6A3 | 81 |
| GO:0010959 | regulation of metal ion transport | 75/2213 | 363/17653 | 7.31E-06 | 0.001816689 | 0.001617753 | WWP2/RCVRN/APLNR/KCNAB1/KCNC1/RRAD/ATP1A2/P2RX1/GNAI2/PRKACA/GJC2/OSR1/FLNA/ANK2/ORAI1/CCL5/RYR2/PDE4B/FXYD6/GLRX/UCN/TRPV2/DMPK/KCNS1/F2RL3/VAMP2/CASQ1/AKT1/ACTN2/TESC/REM2/GEM/JPH2/KCNMB1/KEL/BEST1/CXCL12/AHCYL1/KCNA5/HTR2A/NEDD4/SCN2B/SLMAP/EGF/CACNA1B/HSPA2/FXYD1/NOS3/GSTM2/CCL2/DRD2/ADORA2A/CACNA2D1/TRPV3/MYLK/RANGRF/KCNE3/HOMER3/CALM1/TF/KCNIP4/LILRB2/AHNAK/ABL1/KCNG1/NEDD4L/SLC9A1/KCNIP3/GJA1/SLC30A1/COMMD1/PKD2/STK39/SCN4B/BAX | 75 |
| GO:0007266 | Rho protein signal transduction | 48/2213 | 202/17653 | 7.41E-06 | 0.001816689 | 0.001617753 | EPS8L1/F2RL2/RTKN/BCR/DLC1/STMN1/RHOD/ARHGEF17/ABR/PLEKHG5/F2RL3/CDC42EP3/CTNNAL1/FGD4/ARHGEF7/OBSCN/FLOT1/ABRA/PLEKHG6/ADRA1A/ARHGEF10L/ARHGEF3/COL1A2/VANGL2/ARAP3/PLEKHG3/PLEKHG7/NET1/RHOU/RHOBTB2/PREX2/APOE/RHOB/CNKSR1/PDPN/APOA1/NGEF/ARHGAP29/GPR20/ABL1/MYOC/RHOC/ARHGEF19/COL3A1/RASGRF1/GNA13/RND3/RHOV | 48 |
| GO:0070252 | actin-mediated cell contraction | 31/2213 | 110/17653 | 8.58E-06 | 0.001870419 | 0.0016656 | MYLK2/MYL2/MYH7/ATP1A2/FLNA/ANK2/RYR2/PDE4B/CTNNA3/TCAP/MYH4/MYL3/ACTN2/FRMD6/GJA5/TNNI1/KCNA5/TPM4/SCN2B/CACNA2D1/TNNI2/KCNE3/PDPN/TNNT2/DES/NEDD4L/TNNT3/SLC9A1/GJA1/MYBPC1/SCN4B | 31 |
| GO:0035296 | regulation of tube diameter | 35/2213 | 131/17653 | 8.59E-06 | 0.001870419 | 0.0016656 | HTR1B/EDNRA/WDR35/ATP1A2/P2RX1/CHRM1/CHGA/PRKG1/ADRA2C/UCN/CALCA/TBXA2R/AKT1/NOS2/SERPINF2/HRH2/EPHX2/LEP/ADRA1A/KCNMB1/GJA5/HRH1/KEL/KCNA5/HTR2A/AVPR1A/NOS3/ADORA2A/APOE/ADCY6/NPPC/EGFR/EDN3/UTS2R/GJA1 | 35 |
| GO:0097746 | regulation of blood vessel diameter | 35/2213 | 131/17653 | 8.59E-06 | 0.001870419 | 0.0016656 | HTR1B/EDNRA/WDR35/ATP1A2/P2RX1/CHRM1/CHGA/PRKG1/ADRA2C/UCN/CALCA/TBXA2R/AKT1/NOS2/SERPINF2/HRH2/EPHX2/LEP/ADRA1A/KCNMB1/GJA5/HRH1/KEL/KCNA5/HTR2A/AVPR1A/NOS3/ADORA2A/APOE/ADCY6/NPPC/EGFR/EDN3/UTS2R/GJA1 | 35 |
| GO:0050817 | coagulation | 72/2213 | 347/17653 | 9.48E-06 | 0.001992147 | 0.001773998 | F2RL2/DGKG/GNA15/SCUBE1/HIST1H3A/P2RX1/PRKACA/PROC/SERPIND1/HIST1H3C/FLNA/GP9/F10/PLEK/TSPAN8/C1QTNF1/HIST1H3E/GP1BB/ALOX12/S100A9/FAP/CLIC1/METAP1/ZFPM1/PRKG1/ADRA2C/ITGA2/F2RL3/TBXA2R/DGKZ/AKT1/ADAMTS18/MICAL1/SERPINF2/MYL9/PAFAH2/LCK/HSPB1/MAPK3/SELP/VKORC1/LCP2/MFN2/COL1A2/CSRP1/PDGFA/GP1BA/PLAT/NOS3/ADORA2A/AK3/ENTPD2/APOE/MERTK/SERPING1/RHOB/HBE1/PRKAR2A/PLAUR/PDPN/CD36/GATA5/P2RX6/PRKAR2B/PIK3R6/TFPI/COL3A1/GNA13/AP3B1/ITGA2B/PF4V1/ANXA2 | 72 |
| GO:0060047 | heart contraction | 60/2213 | 275/17653 | 1.06E-05 | 0.002140175 | 0.001905816 | HSPB7/MYLK2/MYL2/MYH7/ATP1A2/PRKACA/CHGA/FLNA/ANK2/POPDC2/RYR2/PDE4B/FXYD6/CTNNA3/BVES/UCN/DMPK/TCAP/CALCA/CASQ1/MYL3/KCNK3/ATP1A4/NPR2/ADRA1A/GJA5/TH/AHCYL1/TNNI1/KCNA5/KCNJ14/SCN2B/AVPR1A/CACNA1B/SREBF1/FXYD1/NOS3/GSTM2/DRD2/CACNA2D1/TNNI2/NPPC/RANGRF/KCNE3/CTGF/EDN3/CALM1/KCNIP4/CSRP3/TNNT2/DES/KCNK6/NEDD4L/TNNT3/SLC9A1/KCNIP3/GJA1/VEGFB/SLC8A2/SCN4B | 60 |
| GO:0034329 | cell junction assembly | 51/2213 | 223/17653 | 1.23E-05 | 0.002410075 | 0.002146161 | CDH5/MACF1/MYO1C/DLC1/VEGFA/HEG1/DSG1/TNF/PRKACA/SH3BP1/LIMS1/FLNA/ANK2/SNAI1/SFRP1/RHOD/CDH20/DAPK3/LAMB3/ITGA2/CRB3/CDH3/ARHGEF7/FLOT1/ACTN2/S100A10/FLNC/FBLIM1/GJA5/PARD6B/ITGA6/SORBS1/UGT8/MMP14/CDH19/LAMA3/MPP7/LDB1/TNS1/MARVELD3/CDH18/GPBAR1/ABL1/MYOC/SLC9A1/LIMS2/RHOC/GJA1/PTPRO/HDAC7/COL16A1 | 51 |
| GO:0003015 | heart process | 60/2213 | 278/17653 | 1.50E-05 | 0.002837562 | 0.002526836 | HSPB7/MYLK2/MYL2/MYH7/ATP1A2/PRKACA/CHGA/FLNA/ANK2/POPDC2/RYR2/PDE4B/FXYD6/CTNNA3/BVES/UCN/DMPK/TCAP/CALCA/CASQ1/MYL3/KCNK3/ATP1A4/NPR2/ADRA1A/GJA5/TH/AHCYL1/TNNI1/KCNA5/KCNJ14/SCN2B/AVPR1A/CACNA1B/SREBF1/FXYD1/NOS3/GSTM2/DRD2/CACNA2D1/TNNI2/NPPC/RANGRF/KCNE3/CTGF/EDN3/CALM1/KCNIP4/CSRP3/TNNT2/DES/KCNK6/NEDD4L/TNNT3/SLC9A1/KCNIP3/GJA1/VEGFB/SLC8A2/SCN4B | 60 |
| GO:0060562 | epithelial tube morphogenesis | 66/2213 | 316/17653 | 1.76E-05 | 0.003167124 | 0.00282031 | PLXNB2/EDNRA/DLC1/HOXA5/FOXC2/VEGFA/FZD1/TNF/HAND2/SFRP2/CSF1R/IRX3/RALA/OSR1/RARA/SFRP1/CCL11/RYR2/KDM2B/FOLR1/BBS5/ESR1/LRP5/SALL4/STARD13/TCAP/TWIST1/B4GALT1/MTHFD1L/SOSTDC1/BMP5/MKS1/WNT9B/KLHL3/BMP4/CTSZ/SIX4/BTRC/VANGL2/TIMELESS/FGF2/EGF/GZF1/TULP3/BBS7/PPP1CA/MMP14/HES5/DCHS1/DDR1/AR/RHOB/TACSTD2/ZEB2/SETDB2/SOX11/SIX2/ABL1/SOX18/KRAS/BCL10/GJA1/GNA13/WNT1/LUZP1/PKD2 | 66 |
| GO:0048704 | embryonic skeletal system morphogenesis | 27/2213 | 93/17653 | 1.78E-05 | 0.003167124 | 0.00282031 | COL11A1/HOXA3/HOXA7/HOXA5/FOXC2/HOXB2/DLX2/OSR1/NDST1/HOXA6/ALX4/TWIST1/MTHFD1L/HOXD10/WNT9B/TGFB3/BMP4/SIX4/COL2A1/TULP3/MMP14/MDFI/HOXD4/HOXD9/SOX11/DSCAML1/SIX2 | 27 |
| GO:0042692 | muscle cell differentiation | 74/2213 | 366/17653 | 1.84E-05 | 0.003183045 | 0.002834488 | WFIKKN2/OBSL1/FHL2/HDAC9/MIR23B/IGFBP5/LDB3/HNRNPU/SMYD1/MAPK12/CAPN2/MYLK2/MYL2/VEGFA/MAMSTR/ADAM12/PDLIM5/MIR199A1/ANK2/POPDC2/MAP2K4/FBXO40/RARA/CD53/KRT8/KRT19/IGF2/MYOM2/BVES/MYOZ3/DMPK/TCAP/FRS2/MYEF2/SOX6/CASQ1/AKT1/OBSCN/FLOT1/ACTN2/MYOM3/FLNC/MIR125B1/MYOZ2/TCF3/KEL/BMP4/MYOCD/RORA/SIX4/MYOM1/AVPR1A/MIR195/CAV2/CDON/MMP14/RBM38/SPEG/TBX5/NKX2-6/MYF6/CSRP3/TNNT2/RB1/NOX4/ABL1/TNNT3/SLC9A1/ANKRD2/KRAS/SORT1/NEXN/MYBPC1/WNT1 | 74 |
| GO:0030168 | platelet activation | 39/2213 | 158/17653 | 2.05E-05 | 0.0034407 | 0.003063928 | F2RL2/DGKG/GNA15/P2RX1/FLNA/GP9/PLEK/C1QTNF1/GP1BB/ALOX12/CLIC1/METAP1/PRKG1/ADRA2C/F2RL3/DGKZ/AKT1/ADAMTS18/MYL9/LCK/HSPB1/MAPK3/SELP/LCP2/COL1A2/CSRP1/PDGFA/GP1BA/NOS3/ENTPD2/APOE/MERTK/RHOB/PDPN/PIK3R6/COL3A1/GNA13/ITGA2B/PF4V1 | 39 |
| GO:0015837 | amine transport | 25/2213 | 84/17653 | 2.25E-05 | 0.00368123 | 0.003278119 | GRM7/HTR1B/SLC18A2/NPY2R/CARTPT/ATP1A2/CHGA/OSR1/SNCG/SV2A/ADRA2C/CHRNA6/LEP/RAB3GAP1/TH/CXCL12/HTR2A/AVPR1A/FGF20/DRD2/ADORA2A/CCK/RHCG/KCNA2/STK39 | 25 |
| GO:0031032 | actomyosin structure organization | 44/2213 | 188/17653 | 2.60E-05 | 0.004140342 | 0.003686956 | OBSL1/LDB3/RTKN/MYLK2/DLC1/MYL2/STMN1/PPM1E/SFRP1/PPP1R9A/KRT8/KRT19/MYOM2/CNN1/MYOZ3/TCAP/PHACTR1/CASQ1/OBSCN/ACTN2/MYH14/SERPINF2/MYOM3/S100A10/MYOZ2/FRMD6/TGFB3/SIX4/ARHGEF10L/MYOM1/SORBS1/TACSTD2/CTGF/CSRP3/TNNT2/APOA1/NOX4/ABL1/MYOC/TNNT3/SLC9A1/MYH10/LIMCH1/MYBPC1 | 44 |
| GO:0015711 | organic anion transport | 88/2213 | 460/17653 | 2.90E-05 | 0.004488889 | 0.003997335 | LCN1/CPT1B/GRM7/CA14/SLC16A9/AQP1/GRM2/TMEM30B/SLC1A5/ATP1A2/SLC6A13/CETP/ACACA/MFSD2A/SLC25A18/OSR1/ABCG8/SLC26A10/FOLR1/SLC2A2/CYB5R2/SLC6A9/SV2A/SLC26A9/SLC38A8/VAMP2/SLCO2A1/AKT1/PLA2G3/SLC25A2/STRA6/NOS2/SLC16A5/SLC22A2/SFXN5/LEP/SLC19A1/ACSL1/LDLR/RAB3GAP1/ABCG1/SLC6A6/ABCC11/CFTR/BEST1/SLC22A17/CA12/AVPR1A/STARD10/DRD2/ADORA2A/SLC10A6/SLC22A1/SLC17A8/APOE/SLC16A11/CROT/TNFSF11/TRPC4/SLC26A8/AGXT/CA1/SLC16A4/ATP10A/ATP8B1/SLC17A3/CD36/IL1B/CCK/CA9/APOA1/SLC36A2/SLC7A7/SLC2A3/SCP2/CA3/SLC6A11/RHAG/SLC7A10/GJA1/THRSP/ATP9A/IRS2/SLC22A3/STK39/SCARB1/ABCC3/SLC22A14 | 88 |
| GO:0090257 | regulation of muscle system process | 53/2213 | 242/17653 | 3.02E-05 | 0.004556297 | 0.004057362 | MIR25/IGFBP5/MYLK2/NPY2R/MYL2/MYH7/ATP1A2/P2RX1/HAND2/PRKACA/CHRM1/CHGA/MIR199A1/FLNA/ANK2/TRIM63/RYR2/PDE4B/DAPK3/PRKG1/CTNNA3/ADRA2C/CNN1/UCN/DMPK/ITGA2/CALCA/TBXA2R/CASQ1/MYL3/ERRFI1/MYL9/ADRA1A/C12orf57/GJA5/TNNI1/MYOCD/NOS3/GSTM2/HSPB6/PLCE1/TNNI2/SGCA/RANGRF/KCNE3/CTGF/CALM1/TNNT2/CHRNB4/CALCRL/TNNT3/SLC9A1/PPARGC1A | 53 |
| GO:0051017 | actin filament bundle assembly | 36/2213 | 144/17653 | 3.13E-05 | 0.004600085 | 0.004096355 | PLS1/DLC1/PFN3/FSCN2/USH1C/STMN1/PPM1E/PLEK/SFRP1/RHOD/PPP1R9A/PHACTR1/SYNPO/SERPINF2/S100A10/SPIRE2/TGFB3/SYNPO2L/ARHGEF10L/SORBS1/PAWR/RHOBTB2/MYO1B/SHROOM1/RHOB/FSCN3/TACSTD2/CTGF/APOA1/NOX4/ABL1/MYOC/SLC9A1/RHOC/SHANK3/RND3 | 36 |
| GO:0008016 | regulation of heart contraction | 53/2213 | 243/17653 | 3.40E-05 | 0.004879165 | 0.004344875 | HSPB7/MYL2/MYH7/ATP1A2/PRKACA/CHGA/FLNA/ANK2/POPDC2/RYR2/PDE4B/FXYD6/CTNNA3/BVES/UCN/DMPK/CALCA/CASQ1/MYL3/KCNK3/ATP1A4/NPR2/ADRA1A/GJA5/TH/AHCYL1/KCNA5/KCNJ14/SCN2B/AVPR1A/CACNA1B/SREBF1/FXYD1/NOS3/GSTM2/DRD2/CACNA2D1/NPPC/RANGRF/KCNE3/CTGF/EDN3/CALM1/KCNIP4/CSRP3/TNNT2/DES/KCNK6/SLC9A1/KCNIP3/GJA1/SLC8A2/SCN4B | 53 |
| GO:0042391 | regulation of membrane potential | 84/2213 | 438/17653 | 3.98E-05 | 0.005576635 | 0.004965969 | WWP2/GLRA1/GRIA4/NPY2R/BCO2/ATP1A2/P2RX1/CHRNA9/CHRNE/SCN9A/GABRG3/FLNA/ANK2/POPDC2/PRKCZ/PPP1R9A/SLC26A10/RYR2/CLIC1/KCNMB3/GLRX/CTNNA3/SLC26A9/BVES/CATSPER1/DMPK/GBA/AKT1/KCNK3/ACTN2/MYH14/GPR88/HCN1/CHRNA6/HTR3B/KCNK18/CNGB3/ATP1A4/GRIK1/GLRB/ADRA1A/GJA5/RAB3GAP1/NTSR2/CFTR/FZD9/RIMS2/KCNA5/NEDD4/SCN2B/SLMAP/PMAIP1/GCLC/CACNA1B/FXYD1/DRD2/ADORA2A/KCNK2/DLD/PXK/CACNA2D1/SLC26A8/RANGRF/KCNE3/CCK/CNIH2/P2RX6/CNGA3/CHRNB4/MPP2/KCNK6/ABL1/NEDD4L/MYOC/KCNA2/SLC9A1/SSH1/GJA1/HTR3A/PANK2/GRIA2/SHANK3/SCN4B/BAX | 84 |
| GO:1901615 | organic hydroxy compound metabolic process | 93/2213 | 497/17653 | 4.20E-05 | 0.005745633 | 0.00511646 | CYP7B1/DPM2/ISYNA1/TTPA/TNF/HAND2/DIO3/CETP/LIPC/ACACA/ACADL/ALDH1A1/NR0B2/FDX1L/PLEK/SNAI1/TTR/GPD2/ITPKB/APOB/ALOX12/PLCD1/CYB5R2/INPP5J/PTH1R/LRP5/AKR1B1/MTMR7/GBA/DKK3/PLCB2/CDH3/BMP5/FDPS/CYP27C1/CYP26C1/EPHX2/GC/LEP/HSD17B7/RNF180/LDLR/DEGS2/TPH1/TH/HRH1/ABCG1/CFTR/TPO/CD244/ITGAM/HSD3B7/NFYB/FGF2/DUOX2/ITPKC/SREBF1/DRD2/CYP1B1/PNMT/PLCH1/ALDH1A2/MOXD1/PLCE1/APOE/INPP5E/CEL/LBR/INPP5A/PNKD/COMT/SULT1C4/RDH5/ZEB2/ACAT2/ATP8B1/CALM1/HMGCS2/CYB5R3/RDH13/IL1B/AOC2/APOA1/SERPINA12/MOGAT2/SCP2/INPP5D/CYP27A1/PANK2/LDLRAP1/CYP1A1/SRD5A2/SCARB1 | 93 |
| GO:0008360 | regulation of cell shape | 39/2213 | 163/17653 | 4.35E-05 | 0.00581041 | 0.005174144 | PLXNB2/ALDOA/PALMD/DLC1/PLXNB1/VEGFA/ITGA7/PALM/CSF1R/RHOD/CCL11/DAPK3/BVES/CDC42EP3/CCL13/FGD4/CSNK1G3/MYH14/S100B/FBLIM1/CSNK1D/ARAP3/RHOU/CCL2/RHOBTB2/WIPF3/RHOB/PDPN/ATP10A/PARVB/PLXNA1/MYH10/MSN/RHOC/PLXNA4/GNA13/FES/RND3/RHOV | 39 |
| GO:0034765 | regulation of ion transmembrane transport | 85/2213 | 446/17653 | 4.53E-05 | 0.005926508 | 0.005277529 | WWP2/APLNR/KCNAB1/KCNC1/KCNE4/RRAD/ATP1A2/PRKACA/GJC2/SCN9A/OSR1/FLNA/ANK2/KCNA6/RYR2/PDE4B/CLIC1/FXYD6/GLRX/CATSPER1/TWIST1/KCNS1/F2RL3/VAMP2/CASQ1/AKT1/KCNA7/ACTN2/CLCNKB/HCN1/TESC/REM2/KCNQ4/GEM/JPH2/KCNMB1/KCNA3/GJA5/KEL/CFTR/AHCYL1/KCNA5/KCNJ14/NEDD4/SCN2B/TMEM109/SLMAP/CACNA1B/HSPA2/FXYD1/GSTM2/DRD2/CLCNKA/CACNA2D1/CLIC6/RANGRF/KCNE3/SHISA6/CALM1/KCNIP4/CNIH2/AHNAK/KCNJ9/HVCN1/KCNK6/ABL1/KCNG1/MMP9/NEDD4L/KCNA2/SLC9A1/KCNIP3/GJA1/HTR3A/SLC30A1/IRS2/RASGRF1/KCNJ15/COMMD1/SHANK3/PKD2/STK39/PPARGC1A/SCN4B/BAX | 85 |
| GO:0034330 | cell junction organization | 57/2213 | 270/17653 | 4.68E-05 | 0.005980518 | 0.005325624 | CDH5/MACF1/MYO1C/DLC1/VEGFA/HEG1/DSG1/TNF/PRKACA/SH3BP1/CSF1R/LIMS1/FLNA/ANK2/SNAI1/SFRP1/RHOD/CDH20/DAPK3/LAMB3/ITGA2/CRB3/CLDN9/CDH3/ARHGEF7/PTPN23/FLOT1/ACTN2/S100A10/FLNC/FBLIM1/GJA5/TGFB3/CADM3/PARD6B/ITGA6/SORBS1/UGT8/MMP14/CDH19/LAMA3/MPP7/LDB1/TNS1/MARVELD3/CDH18/GPBAR1/ABL1/MYOC/SLC9A1/LIMS2/RHOC/GJA1/PTPRO/HDAC7/COL16A1/KIFC3 | 57 |
| GO:0030193 | regulation of blood coagulation | 23/2213 | 78/17653 | 5.56E-05 | 0.006807251 | 0.006061826 | PROC/PLEK/TSPAN8/C1QTNF1/ALOX12/S100A9/FAP/PRKG1/TBXA2R/ADAMTS18/SERPINF2/SELP/PDGFA/GP1BA/PLAT/NOS3/APOE/SERPING1/PLAUR/PDPN/CD36/TFPI/ANXA2 | 23 |
| GO:1900046 | regulation of hemostasis | 23/2213 | 78/17653 | 5.56E-05 | 0.006807251 | 0.006061826 | PROC/PLEK/TSPAN8/C1QTNF1/ALOX12/S100A9/FAP/PRKG1/TBXA2R/ADAMTS18/SERPINF2/SELP/PDGFA/GP1BA/PLAT/NOS3/APOE/SERPING1/PLAUR/PDPN/CD36/TFPI/ANXA2 | 23 |
| GO:0061572 | actin filament bundle organization | 36/2213 | 148/17653 | 5.83E-05 | 0.006993791 | 0.00622794 | PLS1/DLC1/PFN3/FSCN2/USH1C/STMN1/PPM1E/PLEK/SFRP1/RHOD/PPP1R9A/PHACTR1/SYNPO/SERPINF2/S100A10/SPIRE2/TGFB3/SYNPO2L/ARHGEF10L/SORBS1/PAWR/RHOBTB2/MYO1B/SHROOM1/RHOB/FSCN3/TACSTD2/CTGF/APOA1/NOX4/ABL1/MYOC/SLC9A1/RHOC/SHANK3/RND3 | 36 |
| GO:0060048 | cardiac muscle contraction | 33/2213 | 132/17653 | 6.56E-05 | 0.007720687 | 0.006875237 | MYLK2/MYL2/MYH7/ATP1A2/PRKACA/CHGA/FLNA/ANK2/RYR2/PDE4B/CTNNA3/UCN/TCAP/MYL3/ADRA1A/GJA5/TNNI1/KCNA5/SCN2B/GSTM2/CACNA2D1/TNNI2/KCNE3/CTGF/CALM1/CSRP3/TNNT2/NEDD4L/TNNT3/SLC9A1/GJA1/VEGFB/SCN4B | 33 |
| GO:0007015 | actin filament organization | 75/2213 | 388/17653 | 7.76E-05 | 0.008945053 | 0.00796553 | OBSL1/PLS1/SPTBN5/BCAR1/EPS8L1/ALDOA/MYO1C/DLC1/FCHSD1/PFN3/CLRN1/AVIL/FSCN2/USH1C/SH3BP1/KANK2/STMN1/DBN1/PPM1E/SPTBN1/FLNA/PLEK/SFRP1/RHOD/PPP1R9A/CCL11/MYOM2/TCAP/PHACTR1/CDC42EP3/SYNPO/MICAL1/ACTN2/SERPINF2/MYOM3/S100A10/EFS/CCL21/SPIRE2/TGFB3/CXCL12/SYNPO2L/TPM4/ARHGEF10L/MYOM1/SORBS1/PAWR/PYCARD/RHOU/RHOBTB2/DIAPH2/WIPF3/MYO1B/SHROOM1/CASS4/RHOB/FSCN3/PPARGC1B/TACSTD2/CTGF/TNNT2/APOA1/NOX4/SPTBN2/ABL1/CSF3/MYOC/SLC9A1/RHOC/CAP2/SSH1/MYBPC1/SHANK3/RND3/RHOV | 75 |
| GO:0048562 | embryonic organ morphogenesis | 59/2213 | 288/17653 | 8.50E-05 | 0.009429219 | 0.008396677 | TMIE/COL11A1/HOXA3/HOXA7/BCR/HOXA5/CLRN1/FOXC2/HAND2/CHRNA9/USH1C/PAX6/HOXB2/DLX2/HOXC4/OSR1/NDST1/RYR2/KDM2B/FOLR1/NAGLU/BBS5/HOXA6/ABR/HMX2/ALX4/TCAP/TWIST1/FRS2/MTHFD1L/HOXD10/STRA6/MYO7A/KCNQ4/WNT9B/MAPK3/TH/TGFB3/ZIC1/BMP4/SIX4/VANGL2/COL2A1/MYO15A/TCF7/TULP3/BBS7/MMP14/MDFI/HOXD4/HOXD9/SETDB2/SOX11/DSCAML1/SIX2/SOX18/GJA1/WNT1/PKD2 | 59 |
| GO:0055002 | striated muscle cell development | 38/2213 | 162/17653 | 8.52E-05 | 0.009429219 | 0.008396677 | WFIKKN2/OBSL1/FHL2/HDAC9/MIR23B/LDB3/HNRNPU/MYLK2/MYL2/VEGFA/PDLIM5/MIR199A1/MAP2K4/KRT8/KRT19/MYOM2/BVES/MYOZ3/TCAP/CASQ1/OBSCN/ACTN2/MYOM3/FLNC/MYOZ2/KEL/BMP4/SIX4/MYOM1/MIR195/CAV2/NKX2-6/MYF6/CSRP3/TNNT2/TNNT3/NEXN/MYBPC1 | 38 |
| GO:0009612 | response to mechanical stimulus | 46/2213 | 209/17653 | 8.66E-05 | 0.009429219 | 0.008396677 | COL11A1/PKD2L2/KCNC1/AQP1/CHEK1/ATP1A2/CHRNA9/SOST/FOXP2/MAP2K4/RYR2/UCN/DENND5B/TCAP/ITGA2/CLCN6/AKT1/PKD2L1/STRA6/CHI3L1/MAPK3/CXCL12/KCNA5/PDE2A/ETS1/HTR2A/PPL/PKDREJ/MAG/ANKRD23/GCLC/DRD2/NRXN2/MMP14/KCNK2/EGFR/IL1B/CSRP3/LTBR/SLC9A1/COL3A1/BCL10/GJA1/SHANK3/PKD2/SCEL | 46 |
| GO:0007512 | adult heart development | 8/2213 | 14/17653 | 8.96E-05 | 0.009586279 | 0.008536539 | APLNR/MYH7/SCUBE1/HAND2/TCAP/ADRA1A/MNAT1/GJA1 | 8 |
| GO:0022604 | regulation of cell morphogenesis | 87/2213 | 468/17653 | 9.31E-05 | 0.009710286 | 0.008646966 | OBSL1/PLXNB2/MACF1/ALDOA/PALMD/DLC1/PLXNB1/DSCAM/VEGFA/TRAK1/ITGA7/PALM/CSF1R/LIMS1/BCL9L/CRABP2/PDLIM5/ZNF135/FLNA/LRP4/RHOD/PPP1R9A/CCL11/DAPK3/NR2E1/BVES/TRPV2/CDC42EP3/CCL13/FGD4/ARHGEF7/CSNK1G3/MYH14/SRCIN1/S100A10/DAB1/OSTN/S100B/SEMA3B/FBLIM1/CSNK1D/KEL/GRIP1/CXCL12/RIMS2/ADNP/NEDD4/ARAP3/MAG/NET1/RHOU/CCL2/GOLGA4/RHOBTB2/CPNE6/APOE/WIPF3/KNDC1/ANAPC2/BARHL2/RHOB/TACSTD2/LRP8/ZEB2/PDPN/ATP10A/PARVB/PLXNA1/APOA1/NGEF/SIPA1L1/ABL1/NGF/NEDD4L/MYOC/MYH10/MSN/RHOC/SSH1/CPNE5/PLXNA4/GNA13/FES/PTPRO/SHANK3/RND3/RHOV | 87 |
| GO:0030195 | negative regulation of blood coagulation | 17/2213 | 51/17653 | 9.57E-05 | 0.009710286 | 0.008646966 | PROC/TSPAN8/C1QTNF1/ALOX12/FAP/PRKG1/ADAMTS18/SERPINF2/PDGFA/GP1BA/PLAT/NOS3/APOE/SERPING1/PLAUR/TFPI/ANXA2 | 17 |
| GO:1900047 | negative regulation of hemostasis | 17/2213 | 51/17653 | 9.57E-05 | 0.009710286 | 0.008646966 | PROC/TSPAN8/C1QTNF1/ALOX12/FAP/PRKG1/ADAMTS18/SERPINF2/PDGFA/GP1BA/PLAT/NOS3/APOE/SERPING1/PLAUR/TFPI/ANXA2 | 17 |
| GO:0003014 | renal system process | 29/2213 | 113/17653 | 0.000107854 | 0.010752516 | 0.009575068 | AQP1/BCR/ADCY4/HNF1A/PRKACA/AKR1B1/SERPINF2/ADRA1A/SGK1/GJA5/ADCY5/KLHL3/MCAM/BMP4/AVPR1A/DRD2/ADORA2A/TRPV5/ADCY6/COMT/PRKAR2A/AQP4/PKN1/UTS2R/CHRNB4/PRKAR2B/GJA1/PTPRO/HYAL2 | 29 |
| GO:0030199 | collagen fibril organization | 16/2213 | 47/17653 | 0.000115037 | 0.011199727 | 0.009973307 | LOXL1/COL11A1/DDR2/TNXB/FOXC2/SFRP2/COL5A2/LOXL4/LOXL2/SERPINF2/DPT/COL1A2/COL2A1/CYP1B1/COL3A1/ANXA2 | 16 |
| GO:0009187 | cyclic nucleotide metabolic process | 35/2213 | 147/17653 | 0.000116148 | 0.011199727 | 0.009973307 | RCVRN/AQP1/GRM2/ADCY4/RAMP1/TBL1XR1/VEGFA/GNAI2/TSHR/PALM/CHGA/PDE7A/GPHA2/PDE4B/PDE3B/NOS2/OSTN/NPR2/ADCY5/RORA/PDE2A/ADNP/NOS3/DRD2/ADORA2A/APOE/SSTR4/ADCY6/NPPC/CALM1/GABBR1/CAP2/GUCA2B/SCT/PKD2 | 35 |
| GO:0006942 | regulation of striated muscle contraction | 25/2213 | 92/17653 | 0.000118111 | 0.011205316 | 0.009978284 | MYL2/MYH7/ATP1A2/PRKACA/CHGA/FLNA/ANK2/RYR2/PDE4B/CTNNA3/UCN/DMPK/CASQ1/MYL3/ADRA1A/C12orf57/GJA5/TNNI1/GSTM2/RANGRF/KCNE3/CTGF/CALM1/TNNT3/SLC9A1 | 25 |
| GO:0051952 | regulation of amine transport | 22/2213 | 77/17653 | 0.000134002 | 0.012357714 | 0.011004489 | GRM7/HTR1B/NPY2R/CARTPT/ATP1A2/CHGA/OSR1/SNCG/SV2A/ADRA2C/CHRNA6/LEP/RAB3GAP1/CXCL12/HTR2A/AVPR1A/FGF20/DRD2/ADORA2A/CCK/KCNA2/STK39 | 22 |
| GO:0045807 | positive regulation of endocytosis | 32/2213 | 131/17653 | 0.00013446 | 0.012357714 | 0.011004489 | CLIP3/BCR/VEGFA/TNF/TFR2/ANGPT1/ABR/ITGA2/FLOT1/LRP1/CCL21/TBC1D5/LDLR/TSPAN1/EGF/PYCARD/CCL2/DRD2/APOE/MERTK/TF/CD36/IL1B/MBL2/SGIP1/CLU/SFRP4/NEDD4L/MAGI2/LDLRAP1/CD47/ANXA2 | 32 |
| GO:0061045 | negative regulation of wound healing | 21/2213 | 72/17653 | 0.000136958 | 0.012393643 | 0.011036484 | PROC/TSPAN8/C1QTNF1/ALOX12/FAP/PRKG1/EPPK1/ADAMTS18/SERPINF2/PDGFA/FGF2/GP1BA/PLAT/NOS3/APOE/SERPING1/PLAUR/TFPI/GJA1/CD109/ANXA2 | 21 |
| GO:0031589 | cell-substrate adhesion | 63/2213 | 319/17653 | 0.000150542 | 0.013416457 | 0.011947295 | MACF1/ABI3BP/HOXA7/SPOCK2/DLC1/NPY2R/PLEKHA2/VEGFA/ITGA7/LIMS1/ANGPT1/FLNA/EMILIN1/PRKCZ/SFRP1/RHOD/DAPK3/SNED1/CD96/TMEM8B/LYPD3/BVES/ITGA2/ARHGEF7/ACTN2/SRCIN1/S100A10/MYO1G/CCL21/PARVG/VIT/FERMT1/ITGA6/SORBS1/RADIL/NET1/SMAD6/MMP14/GCNT2/DDR1/SIGLEC1/CYR61/MERTK/FBLN5/ITGA10/EGFLAM/TACSTD2/CTGF/PDPN/LDB1/CD36/PARVB/APOA1/SMOC2/ABL1/MYOC/SLC9A1/COL3A1/COL13A1/WNT1/ITGA2B/PTPRO/COL16A1 | 63 |
| GO:0030049 | muscle filament sliding | 14/2213 | 39/17653 | 0.000160145 | 0.013796907 | 0.012286084 | MYLK2/MYL2/MYH7/TCAP/MYH4/MYL3/ACTN2/TNNI1/TPM4/TNNI2/TNNT2/DES/TNNT3/MYBPC1 | 14 |
| GO:0033275 | actin-myosin filament sliding | 14/2213 | 39/17653 | 0.000160145 | 0.013796907 | 0.012286084 | MYLK2/MYL2/MYH7/TCAP/MYH4/MYL3/ACTN2/TNNI1/TPM4/TNNI2/TNNT2/DES/TNNT3/MYBPC1 | 14 |
| GO:0003231 | cardiac ventricle development | 30/2213 | 121/17653 | 0.000161847 | 0.013796907 | 0.012286084 | COL11A1/NPY2R/MYL2/MYH7/FOXC2/HEG1/FZD1/HAND2/SFRP2/RYR2/ZFPM1/SALL4/FRS2/MYL3/STRA6/HEYL/GJA5/BMP4/TNNI1/MYOCD/VANGL2/SMAD6/KCNK2/CYR61/LTBP1/TBX5/DAND5/SOX11/TNNT2/LUZP1 | 30 |
| GO:0051260 | protein homooligomerization | 63/2213 | 320/17653 | 0.000164974 | 0.013862555 | 0.012344543 | IKZF4/GLRA1/SPTBN5/KCNC1/ALDOA/SCUBE1/P2RX1/ACACA/ACADL/EMILIN1/C1QTNF1/TMEM120A/KCNA6/SCARA5/ALDH5A1/CRYZ/GBA/TP63/KCNS1/C1QTNF2/BEND3/B4GALT1/AQP11/FLOT1/PKD2L1/KCNA7/ACTN2/HCN1/KCTD11/KCNA3/CD79B/ATXN10/DPYS/KCNA5/MGST1/LY6G5B/ATL1/SLC22A1/LNX1/TRPV5/LETM1/ALAD/LGI1/APOE/TNFSF11/KCTD12/CCDC88C/IKBKE/PAM/RIOK3/CLU/P2RX6/ALOX5AP/MPP2/DERL1/KCNG1/KCNA2/RHOC/BCL10/PKD2/KCTD6/BIK/BAX | 63 |
| GO:0060538 | skeletal muscle organ development | 39/2213 | 173/17653 | 0.000170507 | 0.014125698 | 0.012578871 | HDAC9/KCNAB1/SMYD1/MYLK2/FOXP2/BCL9L/POPDC2/NR2F2/SVIL/ASB2/MYOM2/PAX7/BVES/NUPR1/TWIST1/CASQ1/MYL3/FLOT1/HOXD10/STRA6/HEYL/MYH14/S100B/KEL/MYOCD/SIX4/MYOM1/CAV2/CDON/GTF3C5/CNTFR/HOXD9/HIVEP3/MYF6/SOX11/CSRP3/RB1/ANKRD2/PITX1 | 39 |
| GO:0048738 | cardiac muscle tissue development | 47/2213 | 221/17653 | 0.000173286 | 0.014156519 | 0.012606317 | OBSL1/FHL2/COL11A1/MIR25/MIR23B/HNRNPU/MYLK2/MYL2/MYH7/FOXC2/VEGFA/HEG1/PDLIM5/MIR199A1/MAP2K4/RARA/RYR2/MYOM2/ZFPM1/BVES/TCAP/FRS2/SOX6/MYL3/ACTN2/MYOM3/BMP5/JPH2/GJA5/BMP4/TNNI1/MYOCD/MYOM1/FGF20/MIR195/FGF2/KCNK2/ALDH1A2/TBX5/MIR548C/NKX2-6/CSRP3/TNNT2/NOX4/SLC9A1/GJA1/NEXN | 47 |
| GO:0035023 | regulation of Rho protein signal transduction | 32/2213 | 133/17653 | 0.000181688 | 0.014284383 | 0.012720179 | EPS8L1/F2RL2/BCR/DLC1/STMN1/ARHGEF17/ABR/PLEKHG5/F2RL3/FGD4/ARHGEF7/OBSCN/FLOT1/ABRA/PLEKHG6/ADRA1A/ARHGEF10L/ARHGEF3/ARAP3/PLEKHG3/PLEKHG7/NET1/PREX2/APOE/APOA1/NGEF/GPR20/ABL1/MYOC/ARHGEF19/COL3A1/RASGRF1 | 32 |
| GO:0048565 | digestive tract development | 32/2213 | 133/17653 | 0.000181688 | 0.014284383 | 0.012720179 | PKDCC/FOXL1/HOXA5/TNF/SFRP2/SFRP1/ALX4/TP63/CDKN1A/STRA6/DAB1/HRH2/TGFB3/BMP4/MYOCD/NKX2-3/VANGL2/ITGA6/TCF7/BBS7/DCHS1/ALDH1A2/NKX2-6/EGFR/SOX11/GATA5/RB1/SIX2/DACT1/COL3A1/SCT/CYP1A1 | 32 |
| GO:0050892 | intestinal absorption | 13/2213 | 35/17653 | 0.000184087 | 0.014284383 | 0.012720179 | PLS1/ABCG8/SLC2A2/LEP/SLC2A5/LDLR/PNLIP/TJP2/CEL/CD36/APOA1/MOGAT2/SCARB1 | 13 |
| GO:0043270 | positive regulation of ion transport | 50/2213 | 240/17653 | 0.00018627 | 0.014284383 | 0.012720179 | APLNR/KCNC1/NPY2R/P2RX1/GJC2/CHRM1/FLNA/ANK2/ORAI1/CCL5/RYR2/GLRX/UCN/TRPV2/F2RL3/CASQ1/AKT1/ACTN2/TESC/LEP/JPH2/KCNMB1/RAB3GAP1/CFTR/CXCL12/AHCYL1/AVPR1A/HSPA2/FXYD1/GSTM2/CCL2/DRD2/ADORA2A/APOE/CACNA2D1/TNFSF11/TRPV3/MYLK/KCNE3/CALM1/IL1B/CCK/ABL1/SLC9A1/HTR3A/SHANK3/PKD2/STK39/SCN4B/BAX | 50 |
| GO:0050818 | regulation of coagulation | 23/2213 | 84/17653 | 0.000192501 | 0.014284383 | 0.012720179 | PROC/PLEK/TSPAN8/C1QTNF1/ALOX12/S100A9/FAP/PRKG1/TBXA2R/ADAMTS18/SERPINF2/SELP/PDGFA/GP1BA/PLAT/NOS3/APOE/SERPING1/PLAUR/PDPN/CD36/TFPI/ANXA2 | 23 |
| GO:0097756 | negative regulation of blood vessel diameter | 23/2213 | 84/17653 | 0.000192501 | 0.014284383 | 0.012720179 | HTR1B/EDNRA/WDR35/ATP1A2/P2RX1/CHRM1/CHGA/ADRA2C/TBXA2R/AKT1/HRH2/LEP/ADRA1A/GJA5/HRH1/KEL/KCNA5/HTR2A/AVPR1A/EGFR/EDN3/UTS2R/GJA1 | 23 |
| GO:0090066 | regulation of anatomical structure size | 89/2213 | 491/17653 | 0.000193264 | 0.014284383 | 0.012720179 | PLS1/SPTBN5/HTR1B/EPS8L1/EDNRA/MACF1/AQP1/MYO1C/WDR35/FCHSD1/DSCAM/PFN3/FOXC2/VEGFA/AVIL/ATP1A2/P2RX1/USH1C/SH3BP1/KANK2/CHRM1/CHGA/CRABP2/SPTBN1/PLEK/PPP1R9A/CCL11/PRKG1/ADRA2C/UCN/TRPV2/CALCA/CLCN6/CDC42EP3/TBXA2R/RILP/AKT1/ACTN2/NOS2/SERPINF2/HRH2/CCL21/EPHX2/LEP/SEMA3B/ADRA1A/KCNMB1/SPIRE2/WNT9B/GJA5/AKT1S1/HRH1/NTS/KEL/SLC12A4/CXCL12/E2F4/KCNA5/ADNP/HTR2A/AVPR1A/SLC12A6/MAG/GCLC/PYCARD/NOS3/ADORA2A/GOLGA4/PPP1CA/APOE/WIPF3/SLC12A8/ANAPC2/ADCY6/BARHL2/NPPC/EGFR/EDN3/PLXNA1/UTS2R/SPTBN2/ABL1/NGF/CSF3/MSN/SSH1/GJA1/PLXNA4/SHANK3 | 89 |
| GO:0071804 | cellular potassium ion transport | 45/2213 | 210/17653 | 0.000196708 | 0.014284383 | 0.012720179 | WWP2/KCNAB1/KCNC1/AQP1/KCNE4/ATP1A2/KCNN3/FLNA/ANK2/KCNA6/KCNMB3/KCNS1/VAMP2/KCNK3/PKD2L1/KCNA7/ACTN2/HCN1/KCNK18/CNGB3/KCNQ4/ATP1A4/KCNMB1/KCNA3/KEL/SLC12A4/KCNA5/KCNJ14/NEDD4/SLC12A6/KCNK2/SLC12A8/KCNE3/KCNIP4/CNGA3/KCNJ9/KCNK6/KCNG1/NEDD4L/KCNA2/SLC9A1/KCNIP3/KCNJ15/PKD2/STK39 | 45 |
| GO:0071805 | potassium ion transmembrane transport | 45/2213 | 210/17653 | 0.000196708 | 0.014284383 | 0.012720179 | WWP2/KCNAB1/KCNC1/AQP1/KCNE4/ATP1A2/KCNN3/FLNA/ANK2/KCNA6/KCNMB3/KCNS1/VAMP2/KCNK3/PKD2L1/KCNA7/ACTN2/HCN1/KCNK18/CNGB3/KCNQ4/ATP1A4/KCNMB1/KCNA3/KEL/SLC12A4/KCNA5/KCNJ14/NEDD4/SLC12A6/KCNK2/SLC12A8/KCNE3/KCNIP4/CNGA3/KCNJ9/KCNK6/KCNG1/NEDD4L/KCNA2/SLC9A1/KCNIP3/KCNJ15/PKD2/STK39 | 45 |
| GO:0002576 | platelet degranulation | 31/2213 | 128/17653 | 0.000200464 | 0.014379604 | 0.012804973 | PHACTR2/ALDOA/ECM1/VEGFA/P2RX1/APLP2/FLNA/PLEK/IGF2/LGALS3BP/ACTN2/SERPINF2/ITIH4/TGFB3/SELP/CLEC3B/PDGFA/EGF/SERPING1/LHFPL2/CALM1/TF/CD36/VTI1B/APOA1/CLU/LEFTY2/ITGA2B/VEGFB/CD109/OLA1 | 31 |
| GO:0061041 | regulation of wound healing | 32/2213 | 134/17653 | 0.00021046 | 0.014707812 | 0.013097241 | INSL3/FOXC2/PROC/MIR199A1/PLEK/TSPAN8/C1QTNF1/ALOX12/S100A9/FAP/PRKG1/EPPK1/TBXA2R/ADAMTS18/SERPINF2/SOX15/SELP/PDGFA/FGF2/GP1BA/PLAT/NOS3/APOE/SERPING1/MYLK/PLAUR/PDPN/CD36/TFPI/GJA1/CD109/ANXA2 | 32 |
| GO:0060749 | mammary gland alveolus development | 9/2213 | 19/17653 | 0.000212867 | 0.014707812 | 0.013097241 | HOXA5/VEGFA/ESR1/PRLR/TPH1/EGF/DDR1/TNFSF11/AR | 9 |
| GO:0061377 | mammary gland lobule development | 9/2213 | 19/17653 | 0.000212867 | 0.014707812 | 0.013097241 | HOXA5/VEGFA/ESR1/PRLR/TPH1/EGF/DDR1/TNFSF11/AR | 9 |
| GO:0030239 | myofibril assembly | 20/2213 | 69/17653 | 0.000215041 | 0.014707812 | 0.013097241 | OBSL1/LDB3/MYLK2/MYL2/KRT8/KRT19/MYOM2/MYOZ3/TCAP/CASQ1/OBSCN/ACTN2/MYOM3/MYOZ2/SIX4/MYOM1/CSRP3/TNNT2/TNNT3/MYBPC1 | 20 |
| GO:0048706 | embryonic skeletal system development | 30/2213 | 123/17653 | 0.000220788 | 0.014927329 | 0.01329272 | COL11A1/HOXA3/HOXA7/HOXA5/FOXC2/HOXB2/DLX2/OSR1/NDST1/PAX7/HOXA6/ALX4/TWIST1/MTHFD1L/HOXD10/MKS1/WNT9B/TGFB3/BMP4/SIX4/COL2A1/TULP3/MMP14/MDFI/HOXD4/HOXD9/EXT1/SOX11/DSCAML1/SIX2 | 30 |
| GO:0006813 | potassium ion transport | 50/2213 | 242/17653 | 0.000229709 | 0.015353957 | 0.01367263 | WWP2/KCNAB1/KCNC1/AQP1/KCNE4/ATP1A2/KCNN3/FLNA/ANK2/KCNA6/KCNMB3/KCNS1/VAMP2/KCNK3/PKD2L1/KCNA7/ACTN2/HCN1/KCNK18/CNGB3/KCNQ4/ATP1A4/KCNMB1/KCNA3/GJA5/KEL/SLC12A4/KCNA5/KCNJ14/HTR2A/NEDD4/SLC12A6/FXYD1/NOS3/DRD2/KCNK2/SLC12A8/KCNE3/KCNIP4/CNGA3/KCNJ9/KCNK6/KCNG1/NEDD4L/KCNA2/SLC9A1/KCNIP3/KCNJ15/PKD2/STK39 | 50 |
| GO:0019229 | regulation of vasoconstriction | 18/2213 | 60/17653 | 0.000273896 | 0.017903266 | 0.015942778 | ATP1A2/P2RX1/CHRM1/ADRA2C/TBXA2R/AKT1/HRH2/LEP/ADRA1A/GJA5/HRH1/KCNA5/HTR2A/AVPR1A/EGFR/EDN3/UTS2R/GJA1 | 18 |
| GO:0007519 | skeletal muscle tissue development | 37/2213 | 165/17653 | 0.000276031 | 0.017903266 | 0.015942778 | HDAC9/KCNAB1/SMYD1/MYLK2/FOXP2/BCL9L/POPDC2/NR2F2/SVIL/ASB2/MYOM2/PAX7/BVES/NUPR1/TWIST1/CASQ1/MYL3/FLOT1/HOXD10/HEYL/MYH14/S100B/KEL/MYOCD/SIX4/MYOM1/CAV2/CDON/GTF3C5/HOXD9/HIVEP3/MYF6/SOX11/CSRP3/RB1/ANKRD2/PITX1 | 37 |
| GO:0055001 | muscle cell development | 39/2213 | 177/17653 | 0.000280932 | 0.017903266 | 0.015942778 | WFIKKN2/OBSL1/FHL2/HDAC9/MIR23B/LDB3/HNRNPU/MYLK2/MYL2/VEGFA/PDLIM5/MIR199A1/ANK2/MAP2K4/KRT8/KRT19/MYOM2/BVES/MYOZ3/TCAP/CASQ1/OBSCN/ACTN2/MYOM3/FLNC/MYOZ2/KEL/BMP4/SIX4/MYOM1/MIR195/CAV2/NKX2-6/MYF6/CSRP3/TNNT2/TNNT3/NEXN/MYBPC1 | 39 |
| GO:0015893 | drug transport | 42/2213 | 195/17653 | 0.000281284 | 0.017903266 | 0.015942778 | SLC47A2/HTR1B/SLC18A2/AQP1/NPY2R/SLC18A3/SLC1A5/ATP1A2/SLC15A2/NGB/SLC25A18/AQP5/SNCG/FOLR1/SLC6A9/SLC25A2/STRA6/CHRNA6/SLC22A2/SFXN5/SLC19A1/CXCL12/HTR2A/FGF20/DRD2/ADORA2A/SLC22A1/SLC17A8/HBE1/ATP8B1/SLC17A3/SLC36A2/KCNA2/SLC22A18/RHAG/SLC7A10/GJA1/C2orf83/SLC22A3/GIF/ABCC3/ABCA8 | 42 |
| GO:0022612 | gland morphogenesis | 29/2213 | 119/17653 | 0.000283068 | 0.017903266 | 0.015942778 | CYP7B1/IGFBP5/TNF/PAX6/CSF1R/SFRP1/CCL11/FEM1B/ESR1/LRP5/FRS2/TP63/B4GALT1/SOSTDC1/CRIP1/ELF3/TGFB3/BMP4/NKX2-3/BTRC/PDGFA/DUOX2/DDR1/AR/EGFR/PLXNA1/LIMS2/MSN/BAX | 29 |
| GO:0007389 | pattern specification process | 78/2213 | 424/17653 | 0.000293539 | 0.018368022 | 0.016356641 | HOXD8/HOXA3/HOXA7/HOXA5/FOXC2/CER1/HAND2/SFRP2/PAX6/HOXB2/IRX3/SOST/DAAM2/DLX2/HOXC4/OSR1/RFX8/NR2F2/LRP4/SNAI1/SFRP1/KDM2B/FOLR1/BBS5/PAX7/LRP5/HOXA6/PAX1/ALX4/TCAP/RFX4/FRS2/TP63/FOXJ1/HOXD10/SOSTDC1/BMP5/CYP26C1/PGAP1/MKS1/PCDH8/ZIC1/BMP4/ZBTB16/VANGL2/APC2/EGF/CDON/SMAD6/TULP3/BBS7/HES5/DCHS1/ALDH1A2/MDFI/TBX5/HOXC8/HOXD4/HOXD9/DAND5/AR/HOXA10/ZEB2/MYF6/SETDB2/LDB1/DSCAML1/RING1/SOX1/SIX2/MID1/SOX18/NBL1/MEOX1/GJA1/PLD6/WNT1/PKD2 | 78 |
| GO:0060415 | muscle tissue morphogenesis | 22/2213 | 81/17653 | 0.000297995 | 0.018450574 | 0.016430154 | COL11A1/MYLK2/MYL2/MYH7/FOXC2/HEG1/FZD1/RYR2/MYOM2/ZFPM1/PAX7/TCAP/MYL3/MYOM3/TNNI1/VANGL2/MYOM1/MIR195/MYLK/MYF6/TNNT2/COL3A1 | 22 |
| GO:0042310 | vasoconstriction | 21/2213 | 76/17653 | 0.000313729 | 0.019024266 | 0.016941024 | HTR1B/EDNRA/ATP1A2/P2RX1/CHRM1/ADRA2C/TBXA2R/AKT1/HRH2/LEP/ADRA1A/GJA5/HRH1/KEL/KCNA5/HTR2A/AVPR1A/EGFR/EDN3/UTS2R/GJA1 | 21 |
| GO:0055013 | cardiac muscle cell development | 21/2213 | 76/17653 | 0.000313729 | 0.019024266 | 0.016941024 | OBSL1/FHL2/MIR23B/HNRNPU/MYLK2/MYL2/VEGFA/PDLIM5/MIR199A1/MAP2K4/MYOM2/BVES/TCAP/ACTN2/MYOM3/BMP4/MYOM1/MIR195/NKX2-6/CSRP3/NEXN | 21 |
| GO:0051216 | cartilage development | 40/2213 | 184/17653 | 0.00031741 | 0.019051055 | 0.016964879 | COL11A1/HOXA3/PKDCC/HOXA5/SMAD9/CER1/HAND2/SFRP2/DLX2/GPLD1/HOXC4/OSR1/RARA/SNAI1/CSGALNACT1/PTH1R/PAX7/RUNX3/LOXL2/GDF6/SOX6/BMP5/CHI3L1/LEP/MAPK3/BMP4/TGFBI/ZBTB16/COL2A1/FGF2/HES5/CYR61/NPPC/CTGF/MUSTN1/SIX2/FGF6/PITX1/BMP8B/HYAL2 | 40 |
| GO:0050819 | negative regulation of coagulation | 17/2213 | 56/17653 | 0.000343697 | 0.019762078 | 0.017598043 | PROC/TSPAN8/C1QTNF1/ALOX12/FAP/PRKG1/ADAMTS18/SERPINF2/PDGFA/GP1BA/PLAT/NOS3/APOE/SERPING1/PLAUR/TFPI/ANXA2 | 17 |
| GO:0015696 | ammonium transport | 25/2213 | 98/17653 | 0.000345286 | 0.019762078 | 0.017598043 | HTR1B/SLC18A2/AQP1/NPY2R/SLC18A3/P2RX1/CSF2/SNCG/OAZ3/SLC25A2/CHRNA6/SLC22A2/RHBG/SLC44A2/CXCL12/HTR2A/FGF20/DRD2/ADORA2A/SLC22A1/SLC5A7/RHCG/KCNA2/RHAG/SLC22A3 | 25 |
| GO:0048705 | skeletal system morphogenesis | 44/2213 | 209/17653 | 0.000346247 | 0.019762078 | 0.017598043 | HOXD8/COL11A1/HOXA3/HOXA7/HOXA5/FOXC2/CER1/SFRP2/HOXB2/DLX2/OSR1/RARA/NDST1/SFRP1/CSGALNACT1/LRP5/HOXA6/PAX1/ALX4/TWIST1/MTHFD1L/HOXD10/WNT9B/TGFB3/BMP4/SIX4/COL2A1/TULP3/MMP14/MDFI/HOXC8/HOXD4/HOXD9/NPPC/PPARGC1B/CTGF/SOX11/DSCAML1/SFRP4/SIX2/FGF6/COL13A1/FOXN3/HYAL2 | 44 |
| GO:0050953 | sensory perception of light stimulus | 44/2213 | 209/17653 | 0.000346247 | 0.019762078 | 0.017598043 | NRL/RCVRN/GLRA1/COL11A1/OPN1SW/RD3/CLRN1/FSCN2/USH1C/PAX6/ZNF513/COL18A1/PCDH15/NR2E1/BBS5/SOX14/CRYZ/CDH3/GPR179/MYO7A/CNGB3/GLRB/CRYGC/TH/BEST1/TGFBI/BBS1/COL2A1/CRYBA4/CYP1B1/BBS7/USH2A/OPN4/TACSTD2/RDH5/NYX/GRK7/AOC2/CNGA3/PDE6B/POU6F2/GJA3/RAX2/KIFC3 | 44 |
| GO:0050804 | modulation of chemical synaptic transmission | 64/2213 | 335/17653 | 0.000350474 | 0.019762078 | 0.017598043 | GRM7/HTR1B/PLCL1/GRIA4/GRM2/PAIP2/NPY2R/PREPL/ATP1A2/GNAI2/GFAP/CPLX3/RAPSN/DBN1/USP46/RARA/PRKCZ/PPP1R9A/SNCG/NR2E1/SLC6A9/UCN/DMPK/VAMP2/SYNPO/FLOT1/HRH2/GRIK1/S100B/ADRA1A/RAB3GAP1/HRH1/RIMS2/ADNP/HTR2A/CNTN4/DRD2/ADORA2A/SCGN/PXK/LGI1/APOE/PCDH17/CEL/ANAPC2/PNKD/EGFR/LRP8/SHISA6/IL1B/LILRB2/SIPA1L1/LAMA2/CHRNB4/MPP2/ABL1/NGF/SSH1/KRAS/SLC30A1/RASGRF1/GRIA2/SLC8A2/SHANK3 | 64 |
| GO:0099177 | regulation of trans-synaptic signaling | 64/2213 | 335/17653 | 0.000350474 | 0.019762078 | 0.017598043 | GRM7/HTR1B/PLCL1/GRIA4/GRM2/PAIP2/NPY2R/PREPL/ATP1A2/GNAI2/GFAP/CPLX3/RAPSN/DBN1/USP46/RARA/PRKCZ/PPP1R9A/SNCG/NR2E1/SLC6A9/UCN/DMPK/VAMP2/SYNPO/FLOT1/HRH2/GRIK1/S100B/ADRA1A/RAB3GAP1/HRH1/RIMS2/ADNP/HTR2A/CNTN4/DRD2/ADORA2A/SCGN/PXK/LGI1/APOE/PCDH17/CEL/ANAPC2/PNKD/EGFR/LRP8/SHISA6/IL1B/LILRB2/SIPA1L1/LAMA2/CHRNB4/MPP2/ABL1/NGF/SSH1/KRAS/SLC30A1/RASGRF1/GRIA2/SLC8A2/SHANK3 | 64 |
| GO:0022898 | regulation of transmembrane transporter activity | 49/2213 | 240/17653 | 0.000352774 | 0.019762078 | 0.017598043 | WWP2/KCNAB1/KCNC1/RRAD/ATP1A2/PRKACA/OSR1/ANK2/RYR2/PDE4B/FXYD6/GLRX/TWIST1/KCNS1/VAMP2/CASQ1/ACTN2/TESC/REM2/GEM/JPH2/CFTR/AHCYL1/NEDD4/SCN2B/SLMAP/HSPA2/FXYD1/GSTM2/DRD2/CACNA2D1/RANGRF/KCNE3/SHISA6/CALM1/CNIH2/AHNAK/KCNG1/MMP9/NEDD4L/SLC9A1/GJA1/HTR3A/RASGRF1/SHANK3/PKD2/STK39/PPARGC1A/SCN4B | 49 |
| GO:0034754 | cellular hormone metabolic process | 28/2213 | 115/17653 | 0.000363053 | 0.020146027 | 0.017939947 | AKR1C2/BCO2/ALDH1A1/CRABP2/FDX1L/TTR/ESR1/UGT2B7/AKR1B1/DKK3/STRA6/BMP5/CYP27C1/CYP26C1/HSD17B7/RBP1/CYP1B1/ALDH1A2/AKR1B15/HSD17B8/COMT/RDH5/RDH13/CYP1A1/SRD5A2/SCARB1/UGT2B11/PPARGC1A | 28 |
| GO:0034763 | negative regulation of transmembrane transport | 29/2213 | 121/17653 | 0.000382072 | 0.02100325 | 0.018703301 | KCNAB1/RRAD/ATP1A2/TNF/OSR1/TWIST1/OAZ3/AKT1/ACTN2/REM2/OSTN/GEM/LEP/KEL/NEDD4/GSTM2/DRD2/GRB10/KCNE3/OAZ2/CALM1/IL1B/MMP9/NEDD4L/SLC30A1/IRS2/COMMD1/PKD2/STK39 | 29 |
| GO:0035051 | cardiocyte differentiation | 34/2213 | 150/17653 | 0.000387847 | 0.021036929 | 0.018733291 | OBSL1/FHL2/MIR23B/HNRNPU/MYLK2/MYL2/VEGFA/HAND2/PDLIM5/MIR199A1/MAP2K4/RARA/MYOM2/FOLR1/BVES/TCAP/TWIST1/FRS2/SOX6/ACTN2/MYOM3/MAPK3/BMP4/MYOCD/MYOM1/MIR195/TBX5/NKX2-6/EGFR/CSRP3/NOX4/SOX18/SLC9A1/NEXN | 34 |
| GO:0015695 | organic cation transport | 14/2213 | 42/17653 | 0.000389838 | 0.021036929 | 0.018733291 | SLC18A3/OAZ3/SLC25A2/SLC22A2/RHBG/SLC44A2/ADORA2A/SLC22A1/SLC5A7/RHCG/SLC22A18/RHAG/SLC22A3/SLC22A14 | 14 |
| GO:0001763 | morphogenesis of a branching structure | 42/2213 | 198/17653 | 0.000394985 | 0.021109999 | 0.018798359 | EDNRA/HOXA5/FOXC2/VEGFA/TNF/SFRP2/DLX2/SFRP1/CCL11/FEM1B/ESR1/LRP5/FRS2/TP63/B4GALT1/MKS1/WNT9B/BMP4/CTSZ/SIX4/BTRC/VANGL2/TIMELESS/PDGFA/FGF2/EGF/GZF1/DRD2/PPP1CA/MMP14/DCHS1/DDR1/AR/TACSTD2/PLXNA1/SIX2/ABL1/KRAS/COL13A1/GNA13/WNT1/PKD2 | 42 |
| GO:0008202 | steroid metabolic process | 60/2213 | 311/17653 | 0.00040569 | 0.021109999 | 0.018798359 | CYP7B1/AKR1C2/TNF/CETP/LIPC/ACACA/ACADL/NR0B2/FDX1L/RORC/SNAI1/APOB/CYB5R2/ESR1/LRP5/UGT2B7/AKR1B1/STUB1/PRLR/DKK3/BMP5/FDPS/CYP26C1/EPHX2/GC/LEP/HSD17B7/LDLR/ABCG1/CFTR/RORA/HSD3B7/SULT4A1/NFYB/DHH/SREBF1/CYP1B1/APOE/CEL/LBR/AKR1B15/HSD17B8/COMT/ACAT2/ATP8B1/HMGCS2/CYB5R3/IL1B/APOA1/SERPINA12/SCP2/UGT2B15/CYP27A1/PANK2/LDLRAP1/CYP1A1/SRD5A2/SCARB1/UGT2B11/PPARGC1A | 60 |
| GO:0045907 | positive regulation of vasoconstriction | 12/2213 | 33/17653 | 0.000407634 | 0.021109999 | 0.018798359 | ADRA2C/TBXA2R/AKT1/HRH2/ADRA1A/GJA5/HRH1/HTR2A/AVPR1A/EGFR/UTS2R/GJA1 | 12 |
| GO:0006066 | alcohol metabolic process | 62/2213 | 324/17653 | 0.000410862 | 0.021109999 | 0.018798359 | CYP7B1/DPM2/ISYNA1/TNF/CETP/LIPC/ACACA/ACADL/ALDH1A1/NR0B2/PLEK/TTR/GPD2/ITPKB/APOB/PLCD1/INPP5J/PTH1R/LRP5/AKR1B1/MTMR7/GBA/PLCB2/FDPS/CYP27C1/EPHX2/LEP/HSD17B7/LDLR/DEGS2/HRH1/ABCG1/CFTR/CD244/NFYB/FGF2/ITPKC/SREBF1/CYP1B1/PLCH1/ALDH1A2/MOXD1/PLCE1/APOE/INPP5E/CEL/LBR/INPP5A/SULT1C4/RDH5/ACAT2/CALM1/HMGCS2/CYB5R3/RDH13/IL1B/APOA1/SERPINA12/MOGAT2/INPP5D/LDLRAP1/SCARB1 | 62 |
| GO:0009415 | response to water | 7/2213 | 13/17653 | 0.000414711 | 0.021109999 | 0.018798359 | COL18A1/KRT8/AKR1B1/SIPA1/TH/AVPR1A/PKD2 | 7 |
| GO:0048739 | cardiac muscle fiber development | 7/2213 | 13/17653 | 0.000414711 | 0.021109999 | 0.018798359 | VEGFA/MYOM2/TCAP/MYOM3/BMP4/MYOM1/NEXN | 7 |
| GO:0016339 | calcium-dependent cell-cell adhesion via plasma membrane cell adhesion molecules | 15/2213 | 47/17653 | 0.000416314 | 0.021109999 | 0.018798359 | CDH5/PCDHB4/DSG1/PCDH12/CDH20/PCDHB13/CDH3/PCDHB9/SELP/DCHS1/CDH16/CDH19/PCDHB14/CDH18/PCDHB10 | 15 |
| GO:0060193 | positive regulation of lipase activity | 18/2213 | 62/17653 | 0.000428113 | 0.021522724 | 0.01916589 | EDNRA/GNA15/CCL5/PRKCZ/ESR1/PLCB2/ADRA1A/HTR2A/AVPR1A/FGF2/PNLIP/CYR61/GPIHBP1/EGFR/S1PR4/APOA1/RHOC/GNA13 | 18 |
| GO:0055123 | digestive system development | 33/2213 | 145/17653 | 0.000433956 | 0.021631596 | 0.019262839 | PKDCC/FOXL1/HOXA5/TNF/SFRP2/SFRP1/ALX4/TP63/CDKN1A/STRA6/DAB1/HRH2/TGFB3/BMP4/MYOCD/NKX2-3/VANGL2/ITGA6/TCF7/BBS7/DCHS1/ALDH1A2/NKX2-6/EGFR/SOX11/GATA5/INSR/RB1/SIX2/DACT1/COL3A1/SCT/CYP1A1 | 33 |
| GO:0035637 | multicellular organismal signaling | 42/2213 | 199/17653 | 0.000441127 | 0.021804287 | 0.01941662 | GLRA1/CARTPT/ATP1A2/P2RX1/PRKACA/SCN9A/FLNA/ANK2/RYR2/KCNMB3/FXYD6/CTNNA3/DMPK/GBA/ITGA2/CASQ1/KCNK3/MYH14/GPR88/ATP1A4/NPR2/GJA5/AHCYL1/KCNA5/KCNJ14/SCN2B/AVPR1A/FXYD1/CACNA2D1/NPPC/RANGRF/KCNE3/CALM1/KCNIP4/CHRNB4/KCNK6/KCNA2/SLC9A1/KCNIP3/GJA1/SLC8A2/SCN4B | 42 |
| GO:0032970 | regulation of actin filament-based process | 70/2213 | 377/17653 | 0.000446839 | 0.021902582 | 0.019504151 | SPTBN5/EPS8L1/MYO1C/MYLK2/DLC1/FCHSD1/PFN3/AVIL/ATP1A2/SH3BP1/KANK2/STMN1/CSF1R/PPM1E/SPTBN1/FLNA/ANK2/PLEK/SFRP1/RHOD/PPP1R9A/CCL11/RYR2/PDE4B/DAPK3/CTNNA3/CDC42EP3/DIXDC1/SYNPO/LRP1/ACTN2/SERPINF2/S100A10/ABRA/CCL21/FRMD6/SPIRE2/GJA5/TGFB3/CXCL12/SYNPO2L/ARHGEF10L/VANGL2/PDGFA/PYCARD/RHOU/RHOBTB2/WIPF3/RHOB/RANGRF/KCNE3/TACSTD2/CTGF/PAM/PDPN/TNNT2/APOA1/NOX4/SPTBN2/ABL1/CSF3/MYOC/SLC9A1/RHOC/ARHGEF19/SSH1/FES/SHANK3/RND3/RHOV | 70 |
| GO:0048771 | tissue remodeling | 37/2213 | 169/17653 | 0.000452204 | 0.02198236 | 0.019575193 | HTR1B/IGFBP5/HOXA3/BCR/CARTPT/FOXC2/HAND2/CSF1R/MC4R/CSPG4/MIR199A1/SFRP1/PTH1R/LRP5/ABR/CALCA/RASSF2/B4GALT1/ELF3/LEP/GJA5/TPH1/TGFB3/MIR195/NOS3/MMP14/MITF/MERTK/TNFSF11/EGFR/PPARGC1B/GPNMB/NOX4/CTSK/INPP5D/GJA1/BAX | 37 |
| GO:0003007 | heart morphogenesis | 49/2213 | 243/17653 | 0.00047513 | 0.022907518 | 0.020399043 | FHL2/COL11A1/MYLK2/DLC1/NPY2R/TMEM100/MYL2/MYH7/FOXC2/VEGFA/HEG1/FZD1/HAND2/SFRP2/RARA/SNAI1/RYR2/MYOM2/FOLR1/ZFPM1/BBS5/TCAP/TWIST1/MYL3/HEYL/MYOM3/BMP5/GJA5/TH/BMP4/TNNI1/VANGL2/NEDD4/COL2A1/MYOM1/MIR195/SMAD6/BBS7/DCHS1/ALDH1A2/CYR61/TBX5/SETDB2/SOX11/TNNT2/INSR/SOX18/GJA1/PKD2 | 49 |
| GO:0051056 | regulation of small GTPase mediated signal transduction | 64/2213 | 339/17653 | 0.00048785 | 0.023329536 | 0.020774848 | EPS8L1/F2RL2/SYDE1/BCR/DLC1/DAB2IP/AGAP2/SH3BP1/STMN1/TNK1/CHN2/RHOD/ITPKB/ARHGEF17/SMAP1/ABR/PLEKHG5/STARD13/F2RL3/FGD4/ARHGEF7/OBSCN/FLOT1/SRGAP1/ARHGAP20/SIPA1/ABRA/PLEKHG6/ADRA1A/PSD2/MFN2/ARHGEF10L/ARHGEF3/RALGPS2/ARAP3/PLEKHG3/PLEKHG7/NET1/RHOU/CDON/RHOBTB2/PREX2/GARNL3/PLCE1/APOE/RHOB/APOA1/NGEF/SIPA1L1/ARHGAP29/GPR20/ABL1/NGF/MYOC/RHOC/ARHGEF19/KRAS/COL3A1/RASGRF1/GNA13/PSD3/MADD/SRGAP3/RHOV | 64 |
| GO:0003205 | cardiac chamber development | 35/2213 | 158/17653 | 0.00050596 | 0.023880637 | 0.021265601 | FHL2/COL11A1/NPY2R/MYL2/MYH7/FOXC2/HEG1/FZD1/HAND2/SFRP2/ANK2/RARA/NDST1/RYR2/ZFPM1/SALL4/FRS2/MYL3/STRA6/HEYL/BMP5/GJA5/BMP4/TNNI1/MYOCD/VANGL2/SMAD6/KCNK2/CYR61/LTBP1/TBX5/DAND5/SOX11/TNNT2/LUZP1 | 35 |
| GO:0003002 | regionalization | 63/2213 | 333/17653 | 0.000507494 | 0.023880637 | 0.021265601 | HOXD8/HOXA3/HOXA7/HOXA5/FOXC2/CER1/SFRP2/PAX6/HOXB2/IRX3/SOST/DLX2/HOXC4/OSR1/RFX8/NR2F2/LRP4/SNAI1/SFRP1/KDM2B/PAX7/LRP5/HOXA6/PAX1/ALX4/TCAP/RFX4/FRS2/TP63/FOXJ1/HOXD10/SOSTDC1/CYP26C1/PGAP1/MKS1/PCDH8/BMP4/ZBTB16/VANGL2/EGF/CDON/SMAD6/TULP3/HES5/ALDH1A2/MDFI/HOXC8/HOXD4/HOXD9/AR/HOXA10/ZEB2/MYF6/SETDB2/LDB1/DSCAML1/RING1/SOX1/SIX2/NBL1/MEOX1/PLD6/WNT1 | 63 |
| GO:0009950 | dorsal/ventral axis specification | 8/2213 | 17/17653 | 0.000511846 | 0.023894248 | 0.021277722 | PAX6/SOST/LRP4/SFRP1/LRP5/EGF/SMAD6/MDFI | 8 |
| GO:0060191 | regulation of lipase activity | 22/2213 | 84/17653 | 0.000517472 | 0.023966685 | 0.021342226 | EDNRA/GNA15/LIPC/CCL5/PRKCZ/ESR1/PLCB2/LRP1/ADRA1A/HTR2A/AVPR1A/FGF2/PNLIP/CYR61/GPIHBP1/EGFR/S1PR4/APOA1/ABL1/RHOC/SORT1/GNA13 | 22 |
| GO:0045214 | sarcomere organization | 15/2213 | 48/17653 | 0.000536162 | 0.024499085 | 0.021816326 | OBSL1/LDB3/KRT8/KRT19/MYOM2/TCAP/CASQ1/OBSCN/ACTN2/MYOM3/SIX4/MYOM1/TNNT2/TNNT3/MYBPC1 | 15 |
| GO:0045216 | cell-cell junction organization | 48/2213 | 238/17653 | 0.000537297 | 0.024499085 | 0.021816326 | CDH5/MACF1/MYO1C/DLC1/VEGFA/HEG1/DSG1/TNF/PRKACA/CSF1R/LIMS1/ANK2/SNAI1/SFRP1/RHOD/CDH20/DAPK3/ITGA2/CRB3/CLDN9/CDH3/ARHGEF7/PTPN23/ACTN2/S100A10/GJA5/TGFB3/CADM3/PARD6B/SORBS1/UGT8/MMP14/CDH19/MPP7/LDB1/MARVELD3/CDH18/GPBAR1/ABL1/MYOC/SLC9A1/LIMS2/RHOC/GJA1/PTPRO/HDAC7/COL16A1/KIFC3 | 48 |
| GO:0046068 | cGMP metabolic process | 16/2213 | 53/17653 | 0.000545205 | 0.024668414 | 0.021967112 | RCVRN/AQP1/ADCY4/VEGFA/NOS2/OSTN/NPR2/ADCY5/RORA/PDE2A/ADNP/NOS3/APOE/ADCY6/NPPC/GUCA2B | 16 |
| GO:0090207 | regulation of triglyceride metabolic process | 12/2213 | 34/17653 | 0.000558742 | 0.024983342 | 0.022247554 | TBL1XR1/GPLD1/LDLR/SREBF1/FGF21/APOE/APOA1/SERPINA12/PNPLA2/THRSP/PANK2/SCARB1 | 12 |
| GO:0090596 | sensory organ morphogenesis | 50/2213 | 251/17653 | 0.00056066 | 0.024983342 | 0.022247554 | NRL/TMIE/COL11A1/BCR/DSCAM/CLRN1/VEGFA/FSCN2/CHRNA9/USH1C/DIO3/PAX6/COL5A2/OSR1/AQP5/FJX1/KDM2B/NAGLU/LRP5/ABR/HMX2/TCAP/TWIST1/FRS2/STRA6/MYO7A/HCN1/KCNQ4/C12orf57/MAPK3/TH/ZIC1/BMP4/MFN2/SIX4/VANGL2/COL2A1/MYO15A/EGF/CDON/KRT13/SOX11/RDH13/RING1/SOX1/SIX2/TSKU/WNT1/PTPRM/BAX | 50 |
| GO:0008217 | regulation of blood pressure | 38/2213 | 177/17653 | 0.000570974 | 0.025251637 | 0.02248647 | EDNRA/QRFP/CARTPT/ATP1A2/P2RX1/CHGA/NAV2/MIR199A1/NR2F2/LRP5/UCN/CALCA/TBXA2R/NOS2/SERPINF2/LVRN/NPR2/EPHX2/LEP/ADRA1A/SLC2A5/SGK1/GJA5/CTSZ/COL1A2/DDAH1/AVPR1A/CACNA1B/NOS3/DRD2/AR/EDN3/UTS2R/KCNK6/GJA1/GUCA2B/PTPRO/STK39 | 38 |
| GO:0001101 | response to acid chemical | 62/2213 | 329/17653 | 0.000621403 | 0.027172998 | 0.024197433 | GLRA1/AQP1/CAPN2/AKR1C2/VEGFA/TNF/ACACA/COL18A1/MGMT/COL5A2/OSR1/OGG1/RARA/SFRP1/CLK2/KRT8/APOB/HLCS/FOLR1/AKR1B1/CDO1/ITGA2/ADNP2/AKT1/TESC/CPEB4/SERPINF1/SIPA1/GLRB/CCL21/LEP/KCNMB1/ACSL1/LDLR/WNT9B/IPO5/TH/ZNF354A/ID3/COL1A2/AVPR1A/LANCL2/GCLC/SREBF1/KRT13/TIE1/ALDH1A2/FGF21/ALAD/UCP1/ADCY6/EGFR/CTGF/CD36/SST/PTGER2/FFAR2/COL3A1/GJA1/PKD2/COL16A1/PPARGC1A | 62 |
| GO:0006970 | response to osmotic stress | 19/2213 | 69/17653 | 0.000623658 | 0.027172998 | 0.024197433 | AQP1/KMO/TNF/OSR1/AKR1B1/ITGA2/ERRFI1/TH/MLC1/SLC12A6/RCSD1/MYLK/EGFR/PKN1/MARVELD3/SST/PKD2/STK39/BAX | 19 |
| GO:0016331 | morphogenesis of embryonic epithelium | 33/2213 | 148/17653 | 0.000638116 | 0.027598538 | 0.024576375 | PLXNB2/DLC1/FZD1/SFRP2/IRX3/JAG2/RALA/OSR1/RARA/TGFB1I1/SFRP1/KDM2B/FOLR1/SALL4/TWIST1/TP63/MTHFD1L/BMP5/MKS1/WNT9B/PCDH8/BMP4/SIX4/VANGL2/TULP3/HES5/ALDH1A2/AR/ZEB2/SOX11/ABL1/BCL10/LUZP1 | 33 |
| GO:0030851 | granulocyte differentiation | 11/2213 | 30/17653 | 0.00065191 | 0.027793394 | 0.024749894 | TRIB1/CSF2/RARA/ZFPM1/TESC/SPI1/L3MBTL3/CUL4A/C1QC/CSF3/INPP5D | 11 |
| GO:0061448 | connective tissue development | 48/2213 | 240/17653 | 0.000652072 | 0.027793394 | 0.024749894 | COL11A1/HOXA3/PKDCC/HOXA5/SMAD9/TBL1XR1/FOXC2/CER1/HAND2/SFRP2/DLX2/GPLD1/OXCT1/HOXC4/OSR1/RARA/RORC/SNAI1/ACTA2/CSGALNACT1/PTH1R/PAX7/RUNX3/LRP5/LOXL2/GDF6/SOX6/CRIP1/BMP5/CHI3L1/LEP/MAPK3/BMP4/TGFBI/ZBTB16/COL2A1/FGF2/HES5/CYR61/NPPC/CTGF/MUSTN1/SIX2/FGF6/PITX1/BMP8B/HYAL2/PPARGC1A | 48 |
| GO:0032412 | regulation of ion transmembrane transporter activity | 47/2213 | 234/17653 | 0.000669832 | 0.02834497 | 0.02524107 | WWP2/KCNAB1/KCNC1/RRAD/ATP1A2/PRKACA/OSR1/ANK2/RYR2/PDE4B/FXYD6/GLRX/KCNS1/VAMP2/CASQ1/ACTN2/TESC/REM2/GEM/JPH2/CFTR/AHCYL1/NEDD4/SCN2B/SLMAP/HSPA2/FXYD1/GSTM2/DRD2/CACNA2D1/RANGRF/KCNE3/SHISA6/CALM1/CNIH2/AHNAK/KCNG1/MMP9/NEDD4L/SLC9A1/HTR3A/RASGRF1/SHANK3/PKD2/STK39/PPARGC1A/SCN4B | 47 |
| GO:0015850 | organic hydroxy compound transport | 46/2213 | 228/17653 | 0.000687292 | 0.028876071 | 0.025714012 | HTR1B/SLC18A2/AQP1/NPY2R/CARTPT/P2RX1/SLC2A13/CETP/LIPC/CHGA/C1QTNF1/ABCG8/APOB/SNCG/ADRA2C/STRA6/LRP1/CHRNA6/SLC22A2/LEP/PGAP1/LDLR/ABCG1/CFTR/CXCL12/HTR2A/FGF20/EGF/PNLIP/DRD2/ADORA2A/SLC10A6/SLC22A1/APOE/CEL/ATP8B1/CD36/APOA1/CLU/SCP2/KCNA2/LDLRAP1/SLC22A3/SCARB1/ABCC3/ANXA2 | 46 |
| GO:0048593 | camera-type eye morphogenesis | 26/2213 | 108/17653 | 0.000695269 | 0.02900405 | 0.025827977 | DSCAM/VEGFA/USH1C/DIO3/PAX6/AQP5/FJX1/KDM2B/LRP5/TWIST1/FRS2/STRA6/HCN1/C12orf57/TH/BMP4/MFN2/EGF/CDON/SOX11/RDH13/RING1/SOX1/TSKU/PTPRM/BAX | 26 |
| GO:0061138 | morphogenesis of a branching epithelium | 39/2213 | 185/17653 | 0.000708531 | 0.029349156 | 0.026135293 | EDNRA/HOXA5/FOXC2/VEGFA/TNF/SFRP2/SFRP1/CCL11/FEM1B/ESR1/LRP5/FRS2/TP63/B4GALT1/MKS1/WNT9B/BMP4/CTSZ/SIX4/BTRC/VANGL2/TIMELESS/PDGFA/FGF2/EGF/GZF1/PPP1CA/MMP14/DCHS1/DDR1/AR/TACSTD2/PLXNA1/SIX2/ABL1/KRAS/GNA13/WNT1/PKD2 | 39 |
| GO:0030299 | intestinal cholesterol absorption | 7/2213 | 14/17653 | 0.000739785 | 0.030218169 | 0.026909145 | ABCG8/LEP/LDLR/PNLIP/CEL/CD36/APOA1 | 7 |
| GO:0072488 | ammonium transmembrane transport | 7/2213 | 14/17653 | 0.000739785 | 0.030218169 | 0.026909145 | AQP1/SLC18A3/SLC22A2/RHBG/SLC22A1/RHCG/RHAG | 7 |
| GO:0052652 | cyclic purine nucleotide metabolic process | 25/2213 | 103/17653 | 0.000768014 | 0.031154872 | 0.027743274 | RCVRN/AQP1/GRM2/ADCY4/RAMP1/GNAI2/TSHR/PALM/GPHA2/NOS2/OSTN/NPR2/ADCY5/ADNP/NOS3/DRD2/ADORA2A/APOE/ADCY6/NPPC/CALM1/GABBR1/CAP2/GUCA2B/SCT | 25 |
| GO:1901888 | regulation of cell junction assembly | 21/2213 | 81/17653 | 0.000792854 | 0.031942259 | 0.028444439 | MACF1/MYO1C/DLC1/VEGFA/TNF/PRKACA/LIMS1/SNAI1/SFRP1/RHOD/DAPK3/FLOT1/S100A10/MMP14/LDB1/GPBAR1/ABL1/MYOC/SLC9A1/GJA1/COL16A1 | 21 |
| GO:0030879 | mammary gland development | 31/2213 | 138/17653 | 0.000803613 | 0.032035976 | 0.028527894 | OAS2/IGFBP5/HOXA5/VEGFA/CSF1R/MGMT/CCL11/ESR1/LRP5/CDO1/PRLR/ITGA2/AKT1/B4GALT1/SOSTDC1/ELF3/TPH1/TGFB3/BMP4/XDH/BTRC/EGF/DDR1/HOXD9/TNFSF11/CSN3/AR/PAM/GJA1/IRS2/BAX | 31 |
| GO:0010927 | cellular component assembly involved in morphogenesis | 26/2213 | 109/17653 | 0.000806074 | 0.032035976 | 0.028527894 | OBSL1/LDB3/MYLK2/MYL2/ANK2/KRT8/KRT19/MYOM2/MYOZ3/TCAP/CASQ1/OBSCN/PLA2G3/ACTN2/MYOM3/MYOZ2/PHLDB1/ZPBP2/CCDC136/SIX4/MYOM1/UGT8/CSRP3/TNNT2/TNNT3/MYBPC1 | 26 |
| GO:0032409 | regulation of transporter activity | 50/2213 | 255/17653 | 0.000812537 | 0.032076142 | 0.028563661 | WWP2/KCNAB1/KCNC1/RRAD/ATP1A2/PRKACA/OSR1/ANK2/RYR2/PDE4B/FXYD6/GLRX/TWIST1/KCNS1/VAMP2/CASQ1/ACTN2/TESC/REM2/GEM/JPH2/SGK1/CFTR/AHCYL1/NEDD4/SCN2B/SLMAP/HSPA2/FXYD1/GSTM2/DRD2/CACNA2D1/RANGRF/KCNE3/SHISA6/CALM1/CNIH2/AHNAK/KCNG1/MMP9/NEDD4L/SLC9A1/GJA1/HTR3A/RASGRF1/SHANK3/PKD2/STK39/PPARGC1A/SCN4B | 50 |
| GO:0070633 | transepithelial transport | 8/2213 | 18/17653 | 0.000820494 | 0.0321743 | 0.028651071 | AQP1/CSF2/GPLD1/RHBG/CFTR/BEST1/AHCYL1/RHCG | 8 |
| GO:0007601 | visual perception | 42/2213 | 205/17653 | 0.000833179 | 0.032241842 | 0.028711216 | NRL/RCVRN/GLRA1/COL11A1/OPN1SW/RD3/CLRN1/FSCN2/PAX6/ZNF513/COL18A1/NR2E1/BBS5/SOX14/CRYZ/CDH3/GPR179/MYO7A/CNGB3/GLRB/CRYGC/TH/BEST1/TGFBI/BBS1/COL2A1/CRYBA4/CYP1B1/BBS7/USH2A/OPN4/TACSTD2/RDH5/NYX/GRK7/AOC2/CNGA3/PDE6B/POU6F2/GJA3/RAX2/KIFC3 | 42 |
| GO:0009952 | anterior/posterior pattern specification | 42/2213 | 205/17653 | 0.000833179 | 0.032241842 | 0.028711216 | HOXD8/HOXA3/HOXA7/HOXA5/FOXC2/CER1/SFRP2/PAX6/HOXB2/HOXC4/OSR1/NR2F2/SFRP1/KDM2B/LRP5/HOXA6/PAX1/ALX4/TCAP/FRS2/HOXD10/CYP26C1/PGAP1/PCDH8/BMP4/ZBTB16/VANGL2/CDON/TULP3/ALDH1A2/HOXC8/HOXD4/HOXD9/HOXA10/ZEB2/MYF6/LDB1/RING1/SIX2/MEOX1/PLD6/WNT1 | 42 |
| GO:0055007 | cardiac muscle cell differentiation | 28/2213 | 121/17653 | 0.000864443 | 0.033076396 | 0.029454383 | OBSL1/FHL2/MIR23B/HNRNPU/MYLK2/MYL2/VEGFA/PDLIM5/MIR199A1/MAP2K4/RARA/MYOM2/BVES/TCAP/FRS2/SOX6/ACTN2/MYOM3/BMP4/MYOCD/MYOM1/MIR195/TBX5/NKX2-6/CSRP3/NOX4/SLC9A1/NEXN | 28 |
| GO:0048644 | muscle organ morphogenesis | 22/2213 | 87/17653 | 0.000865992 | 0.033076396 | 0.029454383 | COL11A1/MYLK2/MYL2/MYH7/FOXC2/HEG1/FZD1/RYR2/MYOM2/ZFPM1/PAX7/TCAP/MYL3/MYOM3/TNNI1/VANGL2/MYOM1/MIR195/MYLK/MYF6/TNNT2/COL3A1 | 22 |
| GO:0009190 | cyclic nucleotide biosynthetic process | 25/2213 | 104/17653 | 0.000892777 | 0.033879458 | 0.030169506 | RCVRN/AQP1/GRM2/ADCY4/RAMP1/GNAI2/TSHR/PALM/GPHA2/NOS2/OSTN/NPR2/ADCY5/ADNP/NOS3/DRD2/ADORA2A/APOE/ADCY6/NPPC/CALM1/GABBR1/CAP2/GUCA2B/SCT | 25 |
| GO:0055006 | cardiac cell development | 21/2213 | 82/17653 | 0.000941964 | 0.03530619 | 0.031440005 | OBSL1/FHL2/MIR23B/HNRNPU/MYLK2/MYL2/VEGFA/PDLIM5/MIR199A1/MAP2K4/MYOM2/BVES/TCAP/ACTN2/MYOM3/BMP4/MYOM1/MIR195/NKX2-6/CSRP3/NEXN | 21 |
| GO:0071229 | cellular response to acid chemical | 41/2213 | 200/17653 | 0.000942379 | 0.03530619 | 0.031440005 | GLRA1/AQP1/CAPN2/AKR1C2/VEGFA/TNF/ACACA/COL5A2/OSR1/RARA/SFRP1/APOB/FOLR1/ADNP2/AKT1/TESC/CPEB4/SERPINF1/SIPA1/LEP/KCNMB1/LDLR/WNT9B/IPO5/ID3/COL1A2/AVPR1A/LANCL2/GCLC/SREBF1/KRT13/ALDH1A2/UCP1/ADCY6/EGFR/PTGER2/FFAR2/COL3A1/PKD2/COL16A1/PPARGC1A | 41 |
| GO:1904062 | regulation of cation transmembrane transport | 57/2213 | 302/17653 | 0.000968108 | 0.035901908 | 0.031970489 | WWP2/APLNR/KCNAB1/KCNC1/RRAD/ATP1A2/PRKACA/GJC2/OSR1/FLNA/ANK2/RYR2/PDE4B/FXYD6/GLRX/TWIST1/KCNS1/F2RL3/VAMP2/CASQ1/ACTN2/TESC/REM2/GEM/JPH2/KCNMB1/KEL/NEDD4/SCN2B/SLMAP/HSPA2/FXYD1/GSTM2/DRD2/CACNA2D1/RANGRF/KCNE3/SHISA6/CALM1/KCNIP4/CNIH2/AHNAK/ABL1/KCNG1/MMP9/NEDD4L/SLC9A1/KCNIP3/SLC30A1/RASGRF1/COMMD1/SHANK3/PKD2/STK39/PPARGC1A/SCN4B/BAX | 57 |
| GO:0050795 | regulation of behavior | 18/2213 | 66/17653 | 0.000970487 | 0.035901908 | 0.031970489 | HTR1B/NPY2R/QRFP/MC4R/CSF2/UCN/STRA6/RAG1/MDK/HTR2A/PER3/DRD2/ADORA2A/APOE/SGIP1/UTS2R/KCNA2/GJA1 | 18 |
| GO:0007187 | G-protein coupled receptor signaling pathway, coupled to cyclic nucleotide second messenger | 47/2213 | 238/17653 | 0.000978826 | 0.035984095 | 0.032043676 | GRM7/HTR1B/EDNRA/GRM2/GPR37L1/ADCY4/NPY2R/GNA15/RAMP1/GNAI2/TSHR/SENP7/PALM/CHRM1/CHGA/MC4R/FLNA/SSTR3/MC2R/SSTR5/RXFP1/PTH1R/MC1R/ADRA2C/CALCA/TBXA2R/CYSLTR2/HRH2/ADRA1A/ADCY5/HRH1/HTR2A/GNAL/CCL2/DRD2/ADORA2A/GLP2R/SSTR4/ADCY6/CCR3/S1PR4/GPR149/TSKU/CALCRL/GABBR1/PTGER2/GNA13 | 47 |
| GO:0015849 | organic acid transport | 58/2213 | 309/17653 | 0.001008294 | 0.03660979 | 0.032600854 | LCN1/CPT1B/GRM7/SLC16A9/GRM2/SLC1A5/ATP1A2/SLC6A13/ACACA/MFSD2A/SLC25A18/OSR1/SLC26A10/FOLR1/SLC6A9/SV2A/SLC26A9/SLC38A8/VAMP2/SLCO2A1/AKT1/PLA2G3/SLC25A2/STRA6/NOS2/SLC16A5/SFXN5/LEP/SLC19A1/ACSL1/RAB3GAP1/SLC6A6/AVPR1A/STARD10/DRD2/ADORA2A/SLC10A6/SLC17A8/APOE/SLC16A11/CROT/TNFSF11/TRPC4/SLC26A8/AGXT/SLC16A4/ATP8B1/CD36/IL1B/CCK/SLC36A2/SLC7A7/SLC6A11/SLC7A10/THRSP/IRS2/STK39/ABCC3 | 58 |
| GO:0046942 | carboxylic acid transport | 58/2213 | 309/17653 | 0.001008294 | 0.03660979 | 0.032600854 | LCN1/CPT1B/GRM7/SLC16A9/GRM2/SLC1A5/ATP1A2/SLC6A13/ACACA/MFSD2A/SLC25A18/OSR1/SLC26A10/FOLR1/SLC6A9/SV2A/SLC26A9/SLC38A8/VAMP2/SLCO2A1/AKT1/PLA2G3/SLC25A2/STRA6/NOS2/SLC16A5/SFXN5/LEP/SLC19A1/ACSL1/RAB3GAP1/SLC6A6/AVPR1A/STARD10/DRD2/ADORA2A/SLC10A6/SLC17A8/APOE/SLC16A11/CROT/TNFSF11/TRPC4/SLC26A8/AGXT/SLC16A4/ATP8B1/CD36/IL1B/CCK/SLC36A2/SLC7A7/SLC6A11/SLC7A10/THRSP/IRS2/STK39/ABCC3 | 58 |
| GO:0002026 | regulation of the force of heart contraction | 10/2213 | 27/17653 | 0.00104451 | 0.037692059 | 0.03356461 | MYL2/MYH7/ATP1A2/CHGA/RYR2/MYL3/ADRA1A/NOS3/CSRP3/SLC9A1 | 10 |
| GO:0006182 | cGMP biosynthetic process | 13/2213 | 41/17653 | 0.001065291 | 0.038207578 | 0.034023678 | RCVRN/AQP1/ADCY4/NOS2/OSTN/NPR2/ADCY5/ADNP/NOS3/APOE/ADCY6/NPPC/GUCA2B | 13 |
| GO:0005976 | polysaccharide metabolic process | 26/2213 | 111/17653 | 0.001074503 | 0.038271198 | 0.034080331 | PPP1R3A/PRKAG3/EPM2AIP1/PCDH12/PYGM/NDST1/IGF2/CSGALNACT1/PPP1CC/GLT8D2/C1QTNF2/AKT1/MANBA/B3GNT7/AGL/SORBS1/EGF/PPP1CA/EXT1/PPP1R1A/CALM1/INSR/PHKG1/IRS2/STBD1/CHIA | 26 |
| GO:0071214 | cellular response to abiotic stimulus | 58/2213 | 310/17653 | 0.001092205 | 0.038271198 | 0.034080331 | TSPYL5/MFAP4/AQP1/OPN1SW/CHEK1/USP47/ATP1A2/PALM/SFRP2/MGMT/GPLD1/OSR1/MAP2K4/SFRP1/METAP1/MC1R/AKR1B1/ITGA2/TP63/CDKN1A/AKT1/PKD2L1/ERRFI1/CRIP1/KCNK18/SIPA1/MAPK3/TP53INP1/PDE2A/NEDD4/TMEM109/AVPR1A/SLC12A6/MAG/NET1/GCLC/IMPACT/EEF1D/RCSD1/ERCC1/MYLK/RHOB/OPN4/EGFR/CALM1/IL1B/GRK7/LTBR/NOX4/HVCN1/PDE6B/SLC9A1/BCL10/GJA1/PKD2/HYAL2/STK39/BAX | 58 |
| GO:0104004 | cellular response to environmental stimulus | 58/2213 | 310/17653 | 0.001092205 | 0.038271198 | 0.034080331 | TSPYL5/MFAP4/AQP1/OPN1SW/CHEK1/USP47/ATP1A2/PALM/SFRP2/MGMT/GPLD1/OSR1/MAP2K4/SFRP1/METAP1/MC1R/AKR1B1/ITGA2/TP63/CDKN1A/AKT1/PKD2L1/ERRFI1/CRIP1/KCNK18/SIPA1/MAPK3/TP53INP1/PDE2A/NEDD4/TMEM109/AVPR1A/SLC12A6/MAG/NET1/GCLC/IMPACT/EEF1D/RCSD1/ERCC1/MYLK/RHOB/OPN4/EGFR/CALM1/IL1B/GRK7/LTBR/NOX4/HVCN1/PDE6B/SLC9A1/BCL10/GJA1/PKD2/HYAL2/STK39/BAX | 58 |
| GO:0043010 | camera-type eye development | 56/2213 | 297/17653 | 0.001093091 | 0.038271198 | 0.034080331 | LIMK2/RD3/DSCAM/FOXC2/VEGFA/USH1C/DIO3/PAX6/DLX2/ZNF513/FOXP2/RARA/AQP5/GPD2/FJX1/KDM2B/MYOM2/NR2E1/BFSP1/LRP5/TWIST1/FRS2/STRA6/HCN1/SERPINF1/WNT9B/PFDN5/C12orf57/RAB3GAP1/TH/BMP4/MFN2/MYOM1/EGF/CRYBA4/CDON/NES/TULP3/CYP1B1/HES5/ALDH1A2/MITF/SLC17A8/MERTK/EGFR/ZEB2/SOX11/RDH13/RING1/SOX1/TSKU/PDE6B/GJA1/PTPRM/CYP1A1/BAX | 56 |
| GO:0048754 | branching morphogenesis of an epithelial tube | 33/2213 | 153/17653 | 0.001168878 | 0.040618695 | 0.036170767 | EDNRA/HOXA5/FOXC2/VEGFA/TNF/SFRP2/CCL11/ESR1/LRP5/B4GALT1/MKS1/WNT9B/BMP4/CTSZ/SIX4/BTRC/VANGL2/TIMELESS/FGF2/EGF/GZF1/PPP1CA/MMP14/DCHS1/DDR1/AR/TACSTD2/SIX2/ABL1/KRAS/GNA13/WNT1/PKD2 | 33 |
| GO:0043647 | inositol phosphate metabolic process | 18/2213 | 67/17653 | 0.001173951 | 0.040618695 | 0.036170767 | ISYNA1/PLEK/ITPKB/PLCD1/INPP5J/PTH1R/MTMR7/PLCB2/HRH1/CD244/FGF2/ITPKC/PLCH1/PLCE1/INPP5E/INPP5A/CALM1/INPP5D | 18 |
| GO:0007044 | cell-substrate junction assembly | 22/2213 | 89/17653 | 0.001197637 | 0.041011554 | 0.036520606 | MACF1/DLC1/VEGFA/LIMS1/SFRP1/RHOD/DAPK3/LAMB3/ITGA2/ARHGEF7/ACTN2/S100A10/ITGA6/SORBS1/MMP14/LAMA3/LDB1/TNS1/ABL1/MYOC/SLC9A1/COL16A1 | 22 |
| GO:0001654 | eye development | 63/2213 | 344/17653 | 0.00119925 | 0.041011554 | 0.036520606 | NRL/LIMK2/RD3/DSCAM/FOXC2/VEGFA/FSCN2/USH1C/DIO3/PAX6/DLX2/ZNF513/FOXP2/COL5A2/RARA/AQP5/GPD2/FJX1/KDM2B/MYOM2/NR2E1/NAGLU/BFSP1/LRP5/TWIST1/FRS2/ADAMTS18/STRA6/MYO7A/HCN1/SERPINF1/WNT9B/PFDN5/C12orf57/RAB3GAP1/TH/BMP4/MFN2/MYOM1/EGF/CRYBA4/CDON/NES/TULP3/CYP1B1/BBS7/HES5/ALDH1A2/MITF/SLC17A8/MERTK/EGFR/ZEB2/SOX11/RDH13/RING1/SOX1/TSKU/PDE6B/GJA1/PTPRM/CYP1A1/BAX | 63 |
| GO:0042462 | eye photoreceptor cell development | 11/2213 | 32/17653 | 0.001213724 | 0.041266609 | 0.036747731 | NRL/VEGFA/FSCN2/USH1C/DIO3/PAX6/NAGLU/MYO7A/HCN1/TH/RDH13 | 11 |
| GO:0031033 | myosin filament organization | 7/2213 | 15/17653 | 0.001237519 | 0.041358431 | 0.036829498 | OBSL1/MYOM2/DAPK3/TCAP/MYOM3/MYOM1/MYBPC1 | 7 |
| GO:0045725 | positive regulation of glycogen biosynthetic process | 7/2213 | 15/17653 | 0.001237519 | 0.041358431 | 0.036829498 | EPM2AIP1/IGF2/C1QTNF2/AKT1/SORBS1/INSR/IRS2 | 7 |
| GO:0098856 | intestinal lipid absorption | 7/2213 | 15/17653 | 0.001237519 | 0.041358431 | 0.036829498 | ABCG8/LEP/LDLR/PNLIP/CEL/CD36/APOA1 | 7 |
| GO:0048545 | response to steroid hormone | 70/2213 | 391/17653 | 0.001247452 | 0.041454874 | 0.03691538 | FHL2/CYP7B1/HTR1B/AQP1/HNRNPU/PMEPA1/DSG1/TRIP4/ATP1A2/TNF/PAQR6/KANK2/NR0B2/TRIM63/RARA/RORC/NR2F2/TGFB1I1/SFRP1/SSTR3/IL1RN/SSTR5/NR2E1/ESR1/UCN/CDO1/CATSPER1/GBA/NR2C1/TP63/CDKN1A/HEYL/ERRFI1/SERPINF1/MDK/S100B/TH/TGFB3/GRIP1/BMP4/RORA/SOX30/NEDD4/AGL/AVPR1A/SREBF1/FOXO3/FIBIN/ALAD/SSTR4/CPN1/AR/EGFR/PPARGC1B/AGXT/CTGF/PAM/PADI2/RB1/CNGA3/UCP3/SST/PTGER2/ESR2/KRAS/TFPI/MED24/KCTD6/SRD5A2/PPARGC1A | 70 |
| GO:0030828 | positive regulation of cGMP biosynthetic process | 8/2213 | 19/17653 | 0.001262404 | 0.041716081 | 0.037147984 | RCVRN/NOS2/OSTN/ADNP/NOS3/APOE/NPPC/GUCA2B | 8 |
| GO:0051291 | protein heterooligomerization | 28/2213 | 124/17653 | 0.001290193 | 0.042396187 | 0.037753615 | IKZF4/GLRA1/IKZF1/HIST1H3A/P2RX1/LIMS1/HIST1H3C/PRKCZ/C1QTNF1/LRP4/TMEM120A/HIST1H3E/HIST1H4B/C1QTNF2/S100A10/GLRB/COL6A2/COL1A2/HSD17B8/HBE1/SEPT9/TNNT2/INSR/CHRNB4/BCL10/MAGI2/C1QTNF6/ANXA2 | 28 |
| GO:0045785 | positive regulation of cell adhesion | 70/2213 | 392/17653 | 0.001336767 | 0.04368259 | 0.038899152 | ABI3BP/SPOCK2/HLA-DMB/NPY2R/PLEKHA2/FOXC2/VEGFA/TNF/SFRP2/CX3CL1/LIMS1/ELANE/ANGPT1/FLNA/CCL5/RARA/PRKCZ/SFRP1/RHOD/ITPKB/JAK3/IGF2/CD46/VTCN1/ITGA2/CD1D/AKT1/ARHGEF7/PTPN23/TNFSF18/FLOT1/RAG1/CD86/SERPINF2/S100A10/CCL21/LEP/LCK/VIT/CXCL12/FERMT1/ZBTB16/ETS1/ITGA6/TNFSF13B/IBSP/NET1/CXCL13/PYCARD/CCL2/GCNT2/CYR61/TNFSF11/EGFLAM/PDPN/LDB1/CD36/IL1B/LILRB2/APOA1/SMOC2/CCDC88B/ABL1/PIK3R6/MYOC/BCL10/AP3B1/CD47/ZP4/COL16A1 | 70 |
| GO:0015872 | dopamine transport | 13/2213 | 42/17653 | 0.001368081 | 0.044214564 | 0.039372872 | HTR1B/SLC18A2/NPY2R/SNCG/CHRNA6/SLC22A2/CXCL12/HTR2A/FGF20/DRD2/SLC22A1/KCNA2/SLC22A3 | 13 |
| GO:0030804 | positive regulation of cyclic nucleotide biosynthetic process | 13/2213 | 42/17653 | 0.001368081 | 0.044214564 | 0.039372872 | RCVRN/RAMP1/TSHR/GPHA2/NOS2/OSTN/ADNP/NOS3/APOE/NPPC/CALM1/GUCA2B/SCT | 13 |
| GO:0030104 | water homeostasis | 18/2213 | 68/17653 | 0.001412563 | 0.044911853 | 0.039993805 | AQP1/ADCY4/PRKACA/STMN1/ALOX12/AKR1B1/GBA/TP63/ADCY5/CFTR/SCNN1A/KRT16/ADCY6/PRKAR2A/AQP4/PRKAR2B/NEDD4L/HYAL2 | 18 |
| GO:0030810 | positive regulation of nucleotide biosynthetic process | 18/2213 | 68/17653 | 0.001412563 | 0.044911853 | 0.039993805 | RCVRN/RAMP1/TSHR/GPHA2/NOS2/OSTN/ADNP/HTR2A/NOS3/APOE/NPPC/CALM1/INSR/GPD1/GUCA2B/SCT/GAPDHS/PPARGC1A | 18 |
| GO:1900373 | positive regulation of purine nucleotide biosynthetic process | 18/2213 | 68/17653 | 0.001412563 | 0.044911853 | 0.039993805 | RCVRN/RAMP1/TSHR/GPHA2/NOS2/OSTN/ADNP/HTR2A/NOS3/APOE/NPPC/CALM1/INSR/GPD1/GUCA2B/SCT/GAPDHS/PPARGC1A | 18 |
| GO:0072606 | interleukin-8 secretion | 10/2213 | 28/17653 | 0.001444069 | 0.04566673 | 0.040666019 | TLR6/MAPKBP1/NOS2/CHI3L1/LEP/CD244/PYCARD/NLRP10/FFAR2/HYAL2 | 10 |
| GO:0048660 | regulation of smooth muscle cell proliferation | 31/2213 | 143/17653 | 0.0014869 | 0.046769766 | 0.041648267 | HTR1B/TRIB1/IGFBP5/TNF/ELANE/CCL5/PRKG1/CNN1/NDRG2/AKR1B1/ITGA2/AKT1/SERPINF2/VIPR2/TGFB3/BMP4/MYOCD/MFN2/FGF2/TERT/ERN1/HES5/MNAT1/COMT/MIR130A/NPPC/EGFR/NOTCH3/CALCRL/MMP9/PPARGC1A | 31 |
| GO:0044262 | cellular carbohydrate metabolic process | 50/2213 | 262/17653 | 0.001501131 | 0.046966243 | 0.041823229 | IGFBP5/ISYNA1/FBP2/PPP1R3A/PRKAG3/EPM2AIP1/PCDH12/PYGM/GPLD1/PLEK/RORC/C1QTNF1/NDST1/CLK2/GPD2/IGF2/CSGALNACT1/PTH1R/PPP1CC/AKR1B1/MTMR7/HKDC1/C1QTNF2/AKT1/B4GALT1/LEP/MANBA/HRH1/B3GNT7/RORA/CD244/AGL/SORBS1/GBA3/PMAIP1/PPP1CA/INPP5E/C1QTNF3/INPP5A/EXT1/HK1/PPP1R1A/CALM1/INSR/PHKG1/SERPINA12/MOGAT2/IRS2/STBD1/PPARGC1A | 50 |
| GO:1903035 | negative regulation of response to wounding | 21/2213 | 85/17653 | 0.001542245 | 0.047997264 | 0.042741349 | PROC/TSPAN8/C1QTNF1/ALOX12/FAP/PRKG1/EPPK1/ADAMTS18/SERPINF2/PDGFA/FGF2/GP1BA/PLAT/NOS3/APOE/SERPING1/PLAUR/TFPI/GJA1/CD109/ANXA2 | 21 |
| GO:0019218 | regulation of steroid metabolic process | 25/2213 | 108/17653 | 0.001583783 | 0.048924507 | 0.043567055 | TNF/ACACA/ACADL/RORC/SNAI1/APOB/STUB1/DKK3/BMP5/FDPS/EPHX2/LEP/LDLR/ABCG1/RORA/NFYB/DHH/SREBF1/APOE/IL1B/APOA1/SERPINA12/PANK2/LDLRAP1/PPARGC1A | 25 |
| GO:0007188 | adenylate cyclase-modulating G-protein coupled receptor signaling pathway | 40/2213 | 199/17653 | 0.001591625 | 0.048924507 | 0.043567055 | GRM7/HTR1B/EDNRA/GRM2/GPR37L1/ADCY4/NPY2R/GNA15/RAMP1/GNAI2/TSHR/SENP7/PALM/CHRM1/CHGA/MC4R/FLNA/MC2R/RXFP1/PTH1R/MC1R/ADRA2C/CALCA/TBXA2R/CYSLTR2/ADRA1A/ADCY5/HRH1/GNAL/DRD2/ADORA2A/GLP2R/ADCY6/CCR3/S1PR4/TSKU/CALCRL/GABBR1/PTGER2/GNA13 | 40 |
| GO:0010517 | regulation of phospholipase activity | 16/2213 | 58/17653 | 0.001596992 | 0.048924507 | 0.043567055 | EDNRA/GNA15/CCL5/PRKCZ/ESR1/PLCB2/LRP1/ADRA1A/HTR2A/AVPR1A/FGF2/CYR61/EGFR/S1PR4/ABL1/GNA13 | 16 |
| GO:1901021 | positive regulation of calcium ion transmembrane transporter activity | 11/2213 | 33/17653 | 0.001616769 | 0.049273748 | 0.043878053 | ANK2/RYR2/CASQ1/JPH2/HSPA2/GSTM2/CACNA2D1/KCNE3/CALM1/SLC9A1/PKD2 | 11 |
| GO:0009791 | post-embryonic development | 22/2213 | 91/17653 | 0.001632772 | 0.049397947 | 0.043988652 | ETNK2/SLC18A2/MYL2/SCUBE1/DSCAM/VEGFA/HEG1/SCN9A/FOXP2/PYGO1/APOB/ALDH5A1/ALX4/SOX6/MYO7A/C12orf57/BMP4/NKX2-3/ERCC1/NPPC/ABL1/BAX | 22 |
| GO:0048871 | multicellular organismal homeostasis | 61/2213 | 335/17653 | 0.00163764 | 0.049397947 | 0.043988652 | LCN1/AQP1/ADCY4/CARTPT/CLRN1/VEGFA/TNF/PRKACA/USH1C/ACACA/STMN1/ACADL/CSF1R/MC4R/ZG16B/PCDH15/ALOX12/IL1RN/PTH1R/AKR1B1/GBA/CALCA/TP63/CDH3/MUC2/HSPB1/MKS1/ADCY5/CFTR/BBS1/HTR2A/COL2A1/DRD2/SCNN1A/KRT16/GCNT2/SLC40A1/ADCY6/TNFSF11/USH2A/PRKAR2A/AQP4/EGFR/PPARGC1B/CTGF/LDB1/TF/IL1B/TEX15/RB1/NOX4/CTSK/PRKAR2B/INPP5D/KRAS/RHAG/NOX3/HYAL2/STK39/PPARGC1A/BAX | 61 |
| GO:0043271 | negative regulation of ion transport | 31/2213 | 144/17653 | 0.001671973 | 0.049921539 | 0.044454907 | GRM7/HTR1B/KCNAB1/RRAD/ATP1A2/OSR1/TWIST1/AKT1/ACTN2/REM2/GEM/LEP/KEL/KCNA5/HTR2A/NEDD4/NOS3/GSTM2/DRD2/PXK/KCNE3/CALM1/LILRB2/SFRP4/MMP9/NEDD4L/SLC30A1/IRS2/COMMD1/PKD2/STK39 | 31 |
| GO:0048592 | eye morphogenesis | 31/2213 | 144/17653 | 0.001671973 | 0.049921539 | 0.044454907 | NRL/DSCAM/VEGFA/FSCN2/USH1C/DIO3/PAX6/COL5A2/AQP5/FJX1/KDM2B/NAGLU/LRP5/TWIST1/FRS2/STRA6/MYO7A/HCN1/C12orf57/TH/BMP4/MFN2/EGF/CDON/SOX11/RDH13/RING1/SOX1/TSKU/PTPRM/BAX | 31 |

| ID | Description | GeneRatio | BgRatio | pvalue | p.adjust | qvalue | geneID | Count |
| --- | --- | --- | --- | --- | --- | --- | --- | --- |
| GO:0030017 | sarcomere | 60/2309 | 197/18698 | 1.15E-11 | 3.55E-09 | 3.11E-09 | OBSL1/FHL2/DUSP27/KY/LDB3/ALDOA/MYLK2/MYL2/FBP2/MYH7/FBXL22/SPTBN1/FLNA/ANK2/TRIM63/KRT8/RYR2/PDE4B/CMYA5/KRT19/MYOM2/MYO18B/ANK1/SMPX/SYNC/MYOZ3/STUB1/TCAP/MYH4/CASQ1/SYNPO/MYL3/OBSCN/ACTN2/MYOM3/FLNC/ABRA/HSPB1/MYOZ2/ADRA1A/JPH2/SYNPO2L/TNNI1/KCNA5/TPM4/MYOM1/TNNI2/CALM1/PALLD/PARVB/CSRP3/TNNT2/DES/PYROXD1/TNNT3/ANKRD2/NEXN/MYBPC1/TIMP4/KCTD6 | 60 |
| GO:0030016 | myofibril | 64/2309 | 219/18698 | 1.79E-11 | 3.55E-09 | 3.11E-09 | OBSL1/FHL2/DUSP27/KY/LDB3/ALDOA/MYLK2/MYL2/FBP2/MYH7/FBXL22/CALD1/SPTBN1/FLNA/ANK2/TRIM63/SVIL/KRT8/RYR2/PDE4B/CMYA5/KRT19/MYOM2/MYO18B/ANK1/SMPX/SYNC/MYOZ3/STUB1/TCAP/MYH4/CASQ1/SYNPO/MYL3/OBSCN/ACTN2/MYOM3/FLNC/ABRA/HSPB1/MYOZ2/ADRA1A/JPH2/SYNPO2L/TNNI1/KCNA5/TPM4/MYOM1/ANKRD23/TNNI2/CALM1/PALLD/PARVB/CSRP3/TNNT2/DES/AHNAK/PYROXD1/TNNT3/ANKRD2/NEXN/MYBPC1/TIMP4/KCTD6 | 64 |
| GO:0044449 | contractile fiber part | 63/2309 | 214/18698 | 1.82E-11 | 3.55E-09 | 3.11E-09 | OBSL1/FHL2/DUSP27/KY/LDB3/ALDOA/MYLK2/MYL2/FBP2/MYH7/FBXL22/SPTBN1/FLNA/ANK2/TRIM63/SVIL/KRT8/RYR2/PDE4B/CMYA5/KRT19/MYOM2/MYO18B/ANK1/SMPX/SYNC/MYOZ3/STUB1/TCAP/MYH4/CASQ1/SYNPO/MYL3/OBSCN/ACTN2/MYL9/MYOM3/FLNC/ABRA/HSPB1/MYOZ2/ADRA1A/JPH2/SYNPO2L/TNNI1/KCNA5/TPM4/MYOM1/TNNI2/CALM1/PALLD/PARVB/CSRP3/TNNT2/DES/AHNAK/PYROXD1/TNNT3/ANKRD2/NEXN/MYBPC1/TIMP4/KCTD6 | 63 |
| GO:0043292 | contractile fiber | 66/2309 | 230/18698 | 2.12E-11 | 3.55E-09 | 3.11E-09 | OBSL1/FHL2/DUSP27/KY/LDB3/ALDOA/MYLK2/MYL2/FBP2/MYH7/FBXL22/CALD1/SPTBN1/FLNA/ANK2/TRIM63/SVIL/KRT8/RYR2/PDE4B/CMYA5/KRT19/MYOM2/MYO18B/ANK1/SMPX/SYNC/MYOZ3/STUB1/TCAP/MYH4/CASQ1/SYNPO/MYL3/OBSCN/ACTN2/MYL9/MYOM3/FLNC/ABRA/HSPB1/MYOZ2/ADRA1A/JPH2/SYNPO2L/TNNI1/KCNA5/TPM4/MYOM1/ANKRD23/TNNI2/CALM1/PALLD/PARVB/CSRP3/TNNT2/DES/AHNAK/PYROXD1/TNNT3/ANKRD2/GJA1/NEXN/MYBPC1/TIMP4/KCTD6 | 66 |
| GO:0031674 | I band | 42/2309 | 138/18698 | 1.39E-08 | 1.87E-06 | 1.64E-06 | OBSL1/FHL2/KY/LDB3/ALDOA/FBP2/MYH7/FBXL22/FLNA/ANK2/TRIM63/KRT8/RYR2/PDE4B/KRT19/MYOM2/ANK1/SYNC/MYOZ3/STUB1/TCAP/CASQ1/SYNPO/MYL3/OBSCN/ACTN2/MYOM3/FLNC/HSPB1/MYOZ2/ADRA1A/JPH2/SYNPO2L/KCNA5/MYOM1/PALLD/PARVB/CSRP3/DES/ANKRD2/NEXN/MYBPC1 | 42 |
| GO:0030018 | Z disc | 39/2309 | 125/18698 | 2.12E-08 | 2.38E-06 | 2.08E-06 | OBSL1/FHL2/KY/LDB3/FBP2/MYH7/FBXL22/FLNA/ANK2/TRIM63/KRT8/RYR2/PDE4B/KRT19/MYOM2/ANK1/SYNC/MYOZ3/STUB1/TCAP/CASQ1/SYNPO/OBSCN/ACTN2/MYOM3/FLNC/HSPB1/MYOZ2/ADRA1A/JPH2/SYNPO2L/KCNA5/MYOM1/PALLD/PARVB/CSRP3/DES/NEXN/MYBPC1 | 39 |
| GO:0031012 | extracellular matrix | 99/2309 | 479/18698 | 1.31E-07 | 1.26E-05 | 1.10E-05 | LOXL1/COL11A1/MFAP4/WISP2/EMILIN3/CD248/ABI3BP/SPOCK2/ECM1/CPZ/TNXB/VEGFA/PXDN/SFRP2/SOST/COL18A1/GPLD1/CSPG4/NAV2/COL5A2/WISP1/EMILIN1/TGFB1I1/SFRP1/S100A9/CPXM2/SPOCK3/LAMB3/CILP/LGALS3BP/SPARCL1/LOXL2/LRRN2/ADAMTS18/PRELP/ADAM11/SERPINF1/CHI3L1/MMRN2/ELN/WNT9B/SOD3/KAZALD1/LTBP2/TGFB3/FLRT1/LTBP3/VIT/BMP4/TGFBI/DPT/MATN2/COL6A2/SPON1/CLEC3B/COL1A2/COL2A1/ITGA6/IBSP/OPTC/LRFN3/HAPLN4/CDON/MMP14/LINGO4/MAMDC2/CYR61/ENTPD2/LTBP1/APOE/FBN3/CLEC14A/FBLN5/TINAGL1/CTSD/CRIP2/USH2A/EGFLAM/LAMA3/ADAMTSL1/CTGF/NYX/ADAMTSL3/APOA1/CLU/SMOC2/LAMA2/LEFTY2/MMP9/MYOC/COL3A1/RTBDN/WNT1/TIMP4/COL6A3/ZP4/COL16A1/PRSS36/ANXA2 | 99 |
| GO:0016323 | basolateral plasma membrane | 53/2309 | 210/18698 | 2.18E-07 | 1.83E-05 | 1.60E-05 | AQP1/MYO1C/RAB17/TSHR/PALM/EPS15/OTOF/CD81/ANK2/AQP5/FOLR1/PTH1R/ANK1/EPPK1/CD1D/DLG2/B4GALT1/FLOT1/LRP1/KCNQ4/SLC19A1/RHBG/LDLR/MLC1/CD300LG/BEST1/VANGL2/ITGA6/SLC12A6/SLC14A1/SLC22A1/SLC40A1/GPIHBP1/CDH16/TRPC4/AQP4/EGFR/TACSTD2/PDPN/TF/CNNM2/CA9/SLC7A7/RHCG/SLC9A1/MSN/LDLRAP1/PROM2/SLC8A2/PKD2/STK39/ABCC3/ANXA2 | 53 |
| GO:0015629 | actin cytoskeleton | 99/2309 | 487/18698 | 2.97E-07 | 2.22E-05 | 1.94E-05 | OBSL1/PLS1/SPTBN5/HSPB7/BCAR1/ALDOA/MYO1C/RTKN/CAPN2/DDR2/DLC1/MYL2/MYH7/AVIL/FSCN2/ACACA/DBN1/ACTG2/CALD1/PDLIM5/SPTBN1/FLNA/RARA/PRKCZ/SVIL/PPP1R9A/CORO6/KRT19/MYOM2/MYO18B/DAPK3/MYOZ3/STK38L/CDC42EP3/MYH4/SYNPO/MYL3/FLOT1/DCTN4/PDLIM7/ACTN2/MYH14/SRCIN1/MYO7A/MYL9/MYOM3/MYO1G/ABRA/DCDC2/MYOZ2/MTSS1L/FBLIM1/PARVG/SEPT12/SYNPO2L/FERMT1/TNNI1/MYO7B/TPM4/VANGL2/MYO15A/MYOM1/SORBS1/PAWR/APC2/SCNN1D/LSP1/ANKRD23/LANCL2/RHOU/WIPF3/DCTN1/ULBP1/MYO1B/TNNI2/RCSD1/SHROOM1/MYLK/SEPT9/FSCN3/PALLD/PARVB/TNNT2/NOTCH3/AHNAK/ACTL7B/VPS18/NOX4/LLGL2/FILIP1/SPTBN2/ABL1/TNNT3/MYH10/CAP2/GABARAP/MYBPC1/PKD2/DYNLL2 | 99 |
| GO:0031430 | M band | 15/2309 | 31/18698 | 9.74E-07 | 6.55E-05 | 5.72E-05 | OBSL1/FHL2/ALDOA/SPTBN1/ANK2/TRIM63/CMYA5/MYOM2/ANK1/SMPX/OBSCN/MYOM3/MYOM1/MYBPC1/KCTD6 | 15 |
| GO:0005578 | proteinaceous extracellular matrix | 78/2309 | 375/18698 | 2.14E-06 | 0.000130456 | 0.000114027 | LOXL1/COL11A1/MFAP4/WISP2/EMILIN3/CD248/ABI3BP/SPOCK2/ECM1/CPZ/TNXB/VEGFA/PXDN/SOST/COL18A1/GPLD1/NAV2/COL5A2/WISP1/EMILIN1/SFRP1/SPOCK3/LAMB3/CILP/LGALS3BP/SPARCL1/LOXL2/LRRN2/ADAMTS18/PRELP/SERPINF1/CHI3L1/MMRN2/ELN/WNT9B/KAZALD1/LTBP2/FLRT1/VIT/BMP4/TGFBI/DPT/MATN2/COL6A2/SPON1/COL1A2/COL2A1/ITGA6/OPTC/LRFN3/HAPLN4/LINGO4/MAMDC2/CYR61/ENTPD2/LTBP1/FBN3/FBLN5/USH2A/EGFLAM/LAMA3/ADAMTSL1/CTGF/NYX/ADAMTSL3/SMOC2/LAMA2/MMP9/MYOC/COL3A1/RTBDN/WNT1/TIMP4/COL6A3/ZP4/COL16A1/PRSS36/ANXA2 | 78 |
| GO:0042383 | sarcolemma | 35/2309 | 128/18698 | 3.50E-06 | 0.000196255 | 0.000171539 | AQP1/ATP1A2/SSPN/ANK2/POPDC2/KRT8/ALOX12/RYR2/KRT19/ANK1/BVES/SYNC/CASQ1/OBSCN/FLOT1/FLNC/DYSF/ADRA1A/SLC2A5/TGFB3/COL6A2/SLMAP/CAV2/FXYD1/CACNA2D1/SGCA/DES/AHNAK/LAMA2/SLC9A1/FGF6/SLC30A1/STBD1/COL6A3/ANXA2 | 35 |
| GO:0031672 | A band | 17/2309 | 43/18698 | 5.98E-06 | 0.000308919 | 0.000270014 | OBSL1/FHL2/ALDOA/MYL2/SPTBN1/ANK2/TRIM63/CMYA5/MYOM2/ANK1/SMPX/MYL3/OBSCN/MYOM3/MYOM1/MYBPC1/KCTD6 | 17 |
| GO:0005938 | cell cortex | 63/2309 | 296/18698 | 9.22E-06 | 0.000432076 | 0.000377661 | GRM7/PLS1/SPTBN5/EXOC3L2/RTKN/CAPN2/DLC1/SEPT8/SH3BP1/DBN1/CALD1/SPTBN1/FLNA/PRKCZ/RHOD/PPP1R9A/KRT19/SEPT10/USP2/RAI14/BFSP1/ARHGEF7/FLOT1/ACTN2/NOS2/MYO7A/DCDC2/MTSS1L/FBLIM1/SPIRE2/GYPC/PHLDB1/SEPT12/PARD6B/CTSZ/RIMS2/TPM4/NEDD4/LANCL2/RHOU/CLTB/RHOBTB2/GPSM1/WIPF3/DCTN1/FAM110C/CRIP2/TRPC4/SHROOM1/RHOB/SEPT9/CNKSR1/CTGF/LLGL2/SPTBN2/MYH10/RHOC/CAP2/PKD2/RND3/RHOV/NCL/ANXA2 | 63 |
| GO:1902495 | transmembrane transporter complex | 67/2309 | 321/18698 | 9.64E-06 | 0.000432076 | 0.000377661 | GLRA1/KCNAB1/KCNC1/CPT1C/GRIA4/LRRC8B/ATP1A2/PRKACA/CHRNA9/CHRNE/SCN9A/GABRG3/ABCG8/KCNA6/RYR2/PDE4B/CLIC1/KCNMB3/CATSPER1/KCNS1/DLG2/VAMP2/PKD2L1/KCNA7/CLCNKB/HCN1/CHRNA6/HTR3B/LRRC8C/CNGB3/KCNQ4/ATP1A4/GRIK1/GLRB/KCNMB1/KCNA3/CFTR/BEST1/KCNA5/KCNJ14/SCN2B/CACNA1B/HSPA2/FXYD1/KCNK2/SCNN1A/CLCNKA/CACNA2D1/TRPC4/CLIC6/KCNE3/SHISA6/CALM1/KCNIP4/CNIH2/VWC2L/CNGA3/CHRNB4/KCNK6/KCNG1/KCNA2/SLC9A1/KCNIP3/HTR3A/GRIA2/SHANK3/SCN4B | 67 |
| GO:0005911 | cell-cell junction | 85/2309 | 440/18698 | 1.58E-05 | 0.000665663 | 0.00058183 | OBSL1/CDH5/HEG1/DSG1/ATP1A2/SH3BP1/GJC2/LIMS1/PCDH12/DBN1/CLDN11/PDLIM5/FLNA/ANK2/CLDN22/PRKCZ/CD53/PMP22/CDH20/KRT8/SLC2A2/CTNNA3/SV2A/BVES/AKR1B1/PLEKHG5/EPPK1/FRS2/CRB3/CLDN9/CDH3/SYNPO/AKT1/B4GALT1/FLOT1/PDLIM7/CLDN25/FRMD6/GYPC/GJA5/CADM3/MLC1/FLRT1/UBN1/PARD6B/LCP2/KCNA5/PPL/VANGL2/ITGA6/STARD10/SORBS1/PANX3/ANKRD23/FXYD1/PPP1CA/TJP2/DSC3/TRPC4/CDH19/SHROOM1/SGCA/RANGRF/TACSTD2/CNKSR1/MPP7/ESAM/DES/MARVELD3/CDH18/AHNAK/CD226/KCNA2/SLC9A1/GJA3/COL13A1/GJA1/MAGI2/CCDC85C/PTPRM/AMOTL1/PKD2/KIFC3/ANXA2/SCN4B | 85 |
| GO:0098589 | membrane region | 65/2309 | 314/18698 | 1.71E-05 | 0.000667038 | 0.000583032 | MYO1C/CAPN2/CLIP3/DLC1/ATP1A2/P2RX1/GNAI2/TNF/PRKACA/PLVAP/TLR6/ANGPT1/ANK2/PRKCZ/LRP4/DAPK3/CD46/BVES/FLOT1/S100A10/LCK/ADRA1A/KCNA3/MAPK3/CD8A/MLC1/GRIP1/LCP2/KCNA5/HTR2A/ITGAM/SORBS1/MAG/CAV2/FXYD1/NOS3/FAM170B/TUBA1B/CTSD/TRPC4/HK1/PRKAR2A/SGCA/RANGRF/EGFR/KCNE3/LRP8/PDPN/CD36/INSR/AHNAK/CD226/PRKAR2B/INPP5D/SLC9A1/KRAS/TFPI/BCL10/GJA1/MARVELD1/PROM2/HYAL2/ARID3A/SCARB1/ANXA2 | 65 |
| GO:0045121 | membrane raft | 63/2309 | 302/18698 | 1.79E-05 | 0.000667038 | 0.000583032 | MYO1C/CAPN2/CLIP3/DLC1/ATP1A2/P2RX1/GNAI2/TNF/PRKACA/PLVAP/TLR6/ANGPT1/ANK2/PRKCZ/LRP4/DAPK3/BVES/FLOT1/S100A10/LCK/ADRA1A/KCNA3/MAPK3/CD8A/MLC1/GRIP1/LCP2/KCNA5/HTR2A/ITGAM/SORBS1/MAG/CAV2/FXYD1/NOS3/TUBA1B/CTSD/TRPC4/HK1/PRKAR2A/SGCA/RANGRF/EGFR/KCNE3/LRP8/PDPN/CD36/INSR/AHNAK/CD226/PRKAR2B/INPP5D/SLC9A1/KRAS/TFPI/BCL10/GJA1/MARVELD1/PROM2/HYAL2/ARID3A/SCARB1/ANXA2 | 63 |
| GO:0099568 | cytoplasmic region | 89/2309 | 468/18698 | 1.89E-05 | 0.000668234 | 0.000584077 | GRM7/PLS1/SPTBN5/KCNAB1/EXOC3L2/RTKN/CAPN2/DLC1/WDR35/SEPT8/TRAK1/PRKACA/SH3BP1/WDR66/DBN1/CALD1/SPTBN1/FLNA/MAP2K4/PRKCZ/RHOD/PPP1R9A/KRT19/SEPT10/USP2/DNAH7/RAI14/BBS5/BFSP1/DNHD1/AKR1B1/CCDC63/ARHGEF7/FLOT1/ACTN2/NOS2/TTLL8/MYO7A/DCDC2/HSPB1/MTSS1L/FBLIM1/SPIRE2/GYPC/PHLDB1/SEPT12/PARD6B/CTSZ/RIMS2/TPM4/BBS1/NEDD4/DNAH6/LANCL2/RHOU/CLTB/TULP3/BBS7/RHOBTB2/GPSM1/INPP5E/WIPF3/DCTN1/DNALI1/FAM110C/CRIP2/TRPC4/SHROOM1/RHOB/SEPT9/PRKAR2A/BLOC1S1/KIF19/CNKSR1/CTGF/AP3M2/LLGL2/RSPH9/SPTBN2/MYH10/RHOC/CAP2/GABARAP/AP3B1/PKD2/RND3/RHOV/NCL/ANXA2 | 89 |
| GO:0098857 | membrane microdomain | 63/2309 | 303/18698 | 1.99E-05 | 0.000668234 | 0.000584077 | MYO1C/CAPN2/CLIP3/DLC1/ATP1A2/P2RX1/GNAI2/TNF/PRKACA/PLVAP/TLR6/ANGPT1/ANK2/PRKCZ/LRP4/DAPK3/BVES/FLOT1/S100A10/LCK/ADRA1A/KCNA3/MAPK3/CD8A/MLC1/GRIP1/LCP2/KCNA5/HTR2A/ITGAM/SORBS1/MAG/CAV2/FXYD1/NOS3/TUBA1B/CTSD/TRPC4/HK1/PRKAR2A/SGCA/RANGRF/EGFR/KCNE3/LRP8/PDPN/CD36/INSR/AHNAK/CD226/PRKAR2B/INPP5D/SLC9A1/KRAS/TFPI/BCL10/GJA1/MARVELD1/PROM2/HYAL2/ARID3A/SCARB1/ANXA2 | 63 |
| GO:1990351 | transporter complex | 67/2309 | 329/18698 | 2.22E-05 | 0.000711644 | 0.00062202 | GLRA1/KCNAB1/KCNC1/CPT1C/GRIA4/LRRC8B/ATP1A2/PRKACA/CHRNA9/CHRNE/SCN9A/GABRG3/ABCG8/KCNA6/RYR2/PDE4B/CLIC1/KCNMB3/CATSPER1/KCNS1/DLG2/VAMP2/PKD2L1/KCNA7/CLCNKB/HCN1/CHRNA6/HTR3B/LRRC8C/CNGB3/KCNQ4/ATP1A4/GRIK1/GLRB/KCNMB1/KCNA3/CFTR/BEST1/KCNA5/KCNJ14/SCN2B/CACNA1B/HSPA2/FXYD1/KCNK2/SCNN1A/CLCNKA/CACNA2D1/TRPC4/CLIC6/KCNE3/SHISA6/CALM1/KCNIP4/CNIH2/VWC2L/CNGA3/CHRNB4/KCNK6/KCNG1/KCNA2/SLC9A1/KCNIP3/HTR3A/GRIA2/SHANK3/SCN4B | 67 |
| GO:0016459 | myosin complex | 22/2309 | 71/18698 | 2.74E-05 | 0.00083768 | 0.000732183 | OBSL1/MYO1C/MYL2/MYH7/ACTG2/MYOM2/MYO18B/MYH4/MYL3/MYH14/MYO7A/MYL9/MYOM3/MYO1G/MYO7B/MYO15A/MYOM1/MYO1B/SHROOM1/MYH10/MYBPC1/DYNLL2 | 22 |
| GO:0034702 | ion channel complex | 60/2309 | 296/18698 | 6.69E-05 | 0.001954203 | 0.001708091 | GLRA1/KCNAB1/KCNC1/CPT1C/GRIA4/LRRC8B/PRKACA/CHRNA9/CHRNE/SCN9A/GABRG3/KCNA6/RYR2/PDE4B/CLIC1/KCNMB3/CATSPER1/KCNS1/DLG2/VAMP2/PKD2L1/KCNA7/CLCNKB/HCN1/CHRNA6/HTR3B/LRRC8C/KCNQ4/GRIK1/GLRB/KCNMB1/KCNA3/CFTR/BEST1/KCNA5/KCNJ14/SCN2B/CACNA1B/HSPA2/KCNK2/SCNN1A/CLCNKA/CACNA2D1/TRPC4/CLIC6/KCNE3/SHISA6/CALM1/KCNIP4/CNIH2/VWC2L/CHRNB4/KCNK6/KCNG1/KCNA2/KCNIP3/HTR3A/GRIA2/SHANK3/SCN4B | 60 |
| GO:0032153 | cell division site | 23/2309 | 81/18698 | 8.40E-05 | 0.002351813 | 0.002055626 | RTKN/SEPT8/RAB11FIP3/RALA/RHOD/SVIL/SEPT10/PPP1CC/TUBGCP4/PLEKHG6/PSD2/SPIRE2/SEPT12/RHOBTB2/MYLK/RHOB/SEPT9/PKN1/MYH10/RHOC/SSH1/HTR3A/RND3 | 23 |
| GO:0016324 | apical plasma membrane | 60/2309 | 300/18698 | 9.93E-05 | 0.002668103 | 0.002332083 | ATP6V0D2/F2RL2/AQP1/KCNE4/UPK2/DDR2/RAB17/DSG1/EPS15/CSPG4/ANK2/PRKCZ/AQP5/ABCG8/FOLR1/SLC2A2/PTH1R/SLC26A9/CRB3/MYO7A/SLC19A1/SLC2A5/MLC1/CD300LG/PARD6B/SLC34A2/CFTR/AHCYL1/KCNA5/VANGL2/DUOX2/FXYD1/KCNK2/SCNN1A/TRPV5/CNTFR/GPIHBP1/USH2A/SHROOM1/EGFR/PLD1/PDPN/ATP8B1/TF/SLC17A3/CD36/NOX4/SPTBN2/RHCG/SLC22A18/SLC9A1/MSN/GJA1/PROM2/AMOTL1/PTPRO/AQP6/HYAL2/STK39/GIF | 60 |
| GO:0044448 | cell cortex part | 38/2309 | 166/18698 | 0.000107636 | 0.002781974 | 0.002431612 | PLS1/SPTBN5/EXOC3L2/RTKN/CAPN2/DLC1/SEPT8/SH3BP1/DBN1/CALD1/SPTBN1/FLNA/PRKCZ/PPP1R9A/KRT19/SEPT10/FLOT1/ACTN2/NOS2/DCDC2/MTSS1L/GYPC/PHLDB1/SEPT12/CTSZ/RIMS2/TPM4/LANCL2/CLTB/WIPF3/DCTN1/TRPC4/SHROOM1/SEPT9/LLGL2/SPTBN2/CAP2/PKD2 | 38 |
| GO:0005581 | collagen trimer | 24/2309 | 88/18698 | 0.000119511 | 0.002974491 | 0.002599884 | COL11A1/C1QTNF7/C1QTNF8/TNXB/COL18A1/COL5A2/EMILIN1/C1QTNF1/C1QTNF2/COL6A2/COL1A2/COL2A1/C1QA/SCARA3/C1QTNF3/C1QC/COL23A1/MBL2/C1QL1/COL3A1/COL13A1/C1QTNF6/COL6A3/COL16A1 | 24 |
| GO:0045178 | basal part of cell | 17/2309 | 53/18698 | 0.000136185 | 0.003268442 | 0.002856815 | AQP1/MYO1C/CLRN1/EPS15/CLDN11/AQP5/FAP/ITGA2/KCNQ4/PHLDB1/ITGA6/EGFR/HOMER3/TACSTD2/TF/LDLRAP1/PKD2 | 17 |
| GO:0045177 | apical part of cell | 69/2309 | 366/18698 | 0.000204372 | 0.004735803 | 0.004139376 | ATP6V0D2/F2RL2/AQP1/KCNE4/UPK2/DDR2/RAB17/DSG1/USH1C/EPS15/CSPG4/ANK2/PRKCZ/AQP5/ABCG8/FAP/FOLR1/SLC2A2/PTH1R/SLC26A9/CRB3/MYO7A/DAB1/SLC19A1/SLC2A5/LDLR/MLC1/CD300LG/PARD6B/SLC34A2/CFTR/AHCYL1/MYO7B/KCNA5/VANGL2/MGST1/DUOX2/PLAT/FXYD1/KCNK2/SCNN1A/DCHS1/TRPV5/CNTFR/GPIHBP1/MYO1B/USH2A/SHROOM1/EGFR/PLD1/PDPN/ATP8B1/TF/SLC17A3/CD36/NOX4/SPTBN2/RHCG/SLC22A18/SLC9A1/MSN/GJA1/PROM2/AMOTL1/PTPRO/AQP6/HYAL2/STK39/GIF | 69 |
| GO:0098858 | actin-based cell projection | 42/2309 | 197/18698 | 0.000257392 | 0.005723139 | 0.005002368 | NPTX2/MYO1C/CLRN1/FSCN2/USH1C/PALM/ACTG2/ANGPT1/PCDH15/AQP5/ACTA2/PPP1R9A/AKR1B1/FGD4/B4GALT1/ACTN2/SRCIN1/MYO7A/MYO1G/PLEKHG6/AOC3/FZD9/MYO7B/CLCA1/MYO15A/ITGA6/STARD10/PDGFA/DNALI1/MYO1B/USH2A/FSCN3/PDPN/ATP8B1/CA9/MSN/RHOC/PROM2/HYAL2/GIF/SCARB1/DYNC1H1 | 42 |
| GO:0098802 | plasma membrane receptor complex | 39/2309 | 179/18698 | 0.000264014 | 0.005723139 | 0.005002368 | CPT1C/GRIA4/RAMP1/ITGA7/CHRNA9/CHRNE/TRAF5/ITGAX/TLR6/TFR2/EMILIN1/ITGA2/DLG2/CHRNA6/HTR3B/GRIK1/CD79B/CD8A/IL13RA1/ITGAM/ITGA6/SORBS1/CNTFR/ITGA10/SHISA6/TF/CSF2RB/CNIH2/INSR/VWC2L/CHRNB4/CALCRL/GABBR1/BCL10/HTR3A/GRIA2/ITGA2B/SHANK3/IL10RB | 39 |
| GO:0044853 | plasma membrane raft | 25/2309 | 98/18698 | 0.000274408 | 0.005762574 | 0.005036836 | DLC1/ATP1A2/PRKACA/PLVAP/LRP4/BVES/FLOT1/ADRA1A/MAPK3/CD8A/MLC1/LCP2/KCNA5/HTR2A/ITGAM/CAV2/FXYD1/NOS3/TRPC4/PRKAR2A/RANGRF/LRP8/INSR/TFPI/SCARB1 | 25 |
| GO:0030863 | cortical cytoskeleton | 26/2309 | 105/18698 | 0.000347432 | 0.006804399 | 0.005947454 | PLS1/SPTBN5/RTKN/CAPN2/DLC1/DBN1/CALD1/SPTBN1/FLNA/PPP1R9A/KRT19/FLOT1/ACTN2/NOS2/DCDC2/MTSS1L/GYPC/RIMS2/TPM4/LANCL2/WIPF3/TRPC4/SHROOM1/LLGL2/SPTBN2/CAP2 | 26 |
| GO:0005902 | microvillus | 22/2309 | 83/18698 | 0.000350672 | 0.006804399 | 0.005947454 | MYO1C/CLRN1/USH1C/ANGPT1/AQP5/AKR1B1/MYO7A/MYO1G/PLEKHG6/AOC3/MYO7B/CLCA1/STARD10/PDGFA/FSCN3/PDPN/CA9/MSN/PROM2/HYAL2/GIF/SCARB1 | 22 |
| GO:0016460 | myosin II complex | 12/2309 | 33/18698 | 0.000354396 | 0.006804399 | 0.005947454 | OBSL1/MYH7/MYOM2/MYH4/MYL3/MYH14/MYL9/MYOM3/MYOM1/SHROOM1/MYH10/MYBPC1 | 12 |
| GO:0030055 | cell-substrate junction | 73/2309 | 401/18698 | 0.000401937 | 0.007502833 | 0.006557927 | FHL2/BCAR1/CAPN2/DDR2/LPP/DLC1/FZD1/MPZL1/LIMS1/CD81/CSPG4/RALA/FLNA/LAYN/TGFB1I1/SVIL/FAP/CD46/CNN1/PPP1CC/SMPX/TSPAN4/EPPK1/ITGA2/DIXDC1/ARHGEF7/FLOT1/DCTN4/PDLIM7/LRP1/ACTN2/FLNC/HSPB1/FBLIM1/PARVG/MAPK3/FLRT1/MCAM/FERMT1/TPM4/CSRP1/ITGA6/LMO7/SORBS1/CAV2/RHOU/MMP14/GRB7/CASS4/RPS29/RHOB/PRKAR2A/EGFR/PLAUR/RPS15/PALLD/TNS1/PARVB/AHNAK/NOX4/RPL31/RPL22/SLC9A1/LIMS2/MSN/KRAS/GJA1/NEXN/GNA13/ITGA2B/FES/RND3/TADA1 | 73 |
| GO:0043025 | neuronal cell body | 80/2309 | 449/18698 | 0.000434685 | 0.007894813 | 0.006900541 | GLRA1/MBP/KCNAB1/KCNC1/PURA/NPTX2/GRIA4/CPZ/TMEM100/RAB17/DAB2IP/GJC2/KCNN3/NGB/FLNA/MAP2K4/RARA/PRKCZ/LRP4/PPP1R9A/APOB/SNCG/SV2A/UCN/CALCA/TMPRSS5/FEZ1/PGRMC1/SYNPO/ARHGEF7/LRP1/SRCIN1/DAB1/SERPINF1/S100B/PSD2/TH/TGFB3/ATXN10/FLRT1/S100A5/ADNP/KCNJ14/HTR2A/PHAX/CACNA1B/PYCARD/DRD2/ADORA2A/PPP1CA/KCNK2/TOP1/RGS10/CPNE6/MAP1A/SLC17A8/APOE/KNDC1/PDYN/CPN1/KCNE3/TTLL7/PAM/LRP8/SLC5A7/VTI1B/CCK/INSR/CNGA3/SPTBN2/ABL1/PRKAR2B/SST/KCNA2/SORT1/HTR3A/CPNE5/SLC8A2/SRD5A2/PPARGC1A | 80 |
| GO:0005924 | cell-substrate adherens junction | 72/2309 | 396/18698 | 0.000455158 | 0.008049118 | 0.007035413 | FHL2/BCAR1/CAPN2/DDR2/LPP/DLC1/FZD1/MPZL1/LIMS1/CD81/CSPG4/RALA/FLNA/LAYN/TGFB1I1/SVIL/FAP/CD46/CNN1/PPP1CC/SMPX/TSPAN4/ITGA2/DIXDC1/ARHGEF7/FLOT1/DCTN4/PDLIM7/LRP1/ACTN2/FLNC/HSPB1/FBLIM1/PARVG/MAPK3/FLRT1/MCAM/FERMT1/TPM4/CSRP1/ITGA6/LMO7/SORBS1/CAV2/RHOU/MMP14/GRB7/CASS4/RPS29/RHOB/PRKAR2A/EGFR/PLAUR/RPS15/PALLD/TNS1/PARVB/AHNAK/NOX4/RPL31/RPL22/SLC9A1/LIMS2/MSN/KRAS/GJA1/NEXN/GNA13/ITGA2B/FES/RND3/TADA1 | 72 |
| GO:0031253 | cell projection membrane | 62/2309 | 332/18698 | 0.000539882 | 0.009302582 | 0.008131016 | SPTBN5/EPS8L1/KCNC1/MACF1/AQP1/OPN1SW/MYO1C/DLC1/PALM/EPS15/GABRG3/CSPG4/SPTBN1/PLEK/SSTR3/TMEM67/FAP/FOLR1/PTH1R/BBS5/ANK1/BVES/B4GALT1/PKD2L1/TESC/PSD2/MTSS1L/SLC34A2/FZD9/FERMT1/BBS1/APC2/CYBRD1/CLTB/DRD2/ADORA2A/BBS7/SCNN1A/USH2A/OPN4/FSCN3/PLAUR/SHISA6/PDPN/PEX19/ATP8B1/SLC17A3/CD36/CA9/GRK7/INSR/CNGA3/MPP2/PDE6B/KCNA2/MSN/GNA13/PSD3/PROM2/SHANK3/PKD2/SCARB1 | 62 |
| GO:0034703 | cation channel complex | 44/2309 | 217/18698 | 0.000584009 | 0.009790543 | 0.008557523 | KCNAB1/KCNC1/CPT1C/GRIA4/PRKACA/SCN9A/KCNA6/RYR2/PDE4B/KCNMB3/CATSPER1/KCNS1/DLG2/VAMP2/PKD2L1/KCNA7/HCN1/HTR3B/KCNQ4/GRIK1/KCNMB1/KCNA3/KCNA5/KCNJ14/SCN2B/CACNA1B/HSPA2/KCNK2/SCNN1A/CACNA2D1/TRPC4/KCNE3/SHISA6/CALM1/KCNIP4/CNIH2/VWC2L/KCNK6/KCNG1/KCNA2/KCNIP3/HTR3A/GRIA2/SCN4B | 44 |
| GO:0005925 | focal adhesion | 71/2309 | 393/18698 | 0.00059734 | 0.009790543 | 0.008557523 | FHL2/BCAR1/CAPN2/DDR2/LPP/DLC1/FZD1/MPZL1/LIMS1/CD81/CSPG4/RALA/FLNA/LAYN/TGFB1I1/SVIL/FAP/CD46/CNN1/PPP1CC/TSPAN4/ITGA2/DIXDC1/ARHGEF7/FLOT1/DCTN4/PDLIM7/LRP1/ACTN2/FLNC/HSPB1/FBLIM1/PARVG/MAPK3/FLRT1/MCAM/FERMT1/TPM4/CSRP1/ITGA6/LMO7/SORBS1/CAV2/RHOU/MMP14/GRB7/CASS4/RPS29/RHOB/PRKAR2A/EGFR/PLAUR/RPS15/PALLD/TNS1/PARVB/AHNAK/NOX4/RPL31/RPL22/SLC9A1/LIMS2/MSN/KRAS/GJA1/NEXN/GNA13/ITGA2B/FES/RND3/TADA1 | 71 |
| GO:0043235 | receptor complex | 68/2309 | 374/18698 | 0.000644789 | 0.010316625 | 0.009017351 | GRM7/PLXNB2/CPT1C/GRIA4/DDR2/GPR37L1/RAMP1/PLXNB1/ITGA7/CHRNA9/TSHR/CHRNE/TRAF5/CSF1R/ITGAX/GABRG3/TLR6/TFR2/EMILIN1/LRP4/ABCG8/PTH1R/LRP5/LOXL4/ITGA2/DLG2/GPRC5C/PKD2L1/LRP1/CHRNA6/HTR3B/GRIK1/LDLR/CD79B/CD8A/IL13RA1/ITGAM/ITGA6/SORBS1/EGF/DDR1/TIE1/CNTFR/MERTK/ITGA10/TRPV3/EGFR/LRP8/SHISA6/TF/CD36/CSF2RB/CNIH2/PLXNA1/INSR/VWC2L/NOTCH3/GPR20/CHRNB4/CALCRL/GABBR1/BCL10/HTR3A/PLXNA4/GRIA2/ITGA2B/SHANK3/IL10RB | 68 |
| GO:0106003 | amyloid-beta complex | 6/2309 | 11/18698 | 0.000935076 | 0.014613286 | 0.012772891 | PGRMC1/APOE/LILRB2/INSR/CLU/GRIA2 | 6 |
| GO:0005901 | caveola | 20/2309 | 78/18698 | 0.00100635 | 0.015369706 | 0.013434048 | DLC1/ATP1A2/PLVAP/LRP4/BVES/FLOT1/ADRA1A/MAPK3/MLC1/KCNA5/HTR2A/CAV2/FXYD1/NOS3/TRPC4/RANGRF/LRP8/INSR/TFPI/SCARB1 | 20 |
| GO:0044420 | extracellular matrix component | 27/2309 | 119/18698 | 0.001158039 | 0.017293384 | 0.015115458 | LOXL1/COL11A1/MFAP4/TNXB/COL18A1/COL5A2/EMILIN1/LAMB3/LOXL2/SERPINF1/MMRN2/ELN/TGFBI/MATN2/COL1A2/COL2A1/ITGA6/ENTPD2/LTBP1/FBLN5/USH2A/EGFLAM/LAMA3/SMOC2/LAMA2/COL3A1/ANXA2 | 27 |
| GO:0032155 | cell division site part | 17/2309 | 64/18698 | 0.001543648 | 0.022193555 | 0.019398502 | RTKN/RAB11FIP3/RALA/SVIL/PPP1CC/TUBGCP4/PLEKHG6/PSD2/SPIRE2/SEPT12/MYLK/RHOB/PKN1/MYH10/RHOC/SSH1/HTR3A | 17 |
| GO:0030864 | cortical actin cytoskeleton | 19/2309 | 75/18698 | 0.001552228 | 0.022193555 | 0.019398502 | PLS1/SPTBN5/RTKN/CAPN2/DLC1/CALD1/SPTBN1/PPP1R9A/KRT19/FLOT1/ACTN2/DCDC2/MTSS1L/LANCL2/WIPF3/SHROOM1/LLGL2/SPTBN2/CAP2 | 19 |
| GO:0005583 | fibrillar collagen trimer | 6/2309 | 12/18698 | 0.001675143 | 0.022513917 | 0.019678518 | COL11A1/TNXB/COL5A2/COL1A2/COL2A1/COL3A1 | 6 |
| GO:0005614 | interstitial matrix | 6/2309 | 12/18698 | 0.001675143 | 0.022513917 | 0.019678518 | ABI3BP/NAV2/KAZALD1/VIT/EGFLAM/SMOC2 | 6 |
| GO:0098643 | banded collagen fibril | 6/2309 | 12/18698 | 0.001675143 | 0.022513917 | 0.019678518 | COL11A1/TNXB/COL5A2/COL1A2/COL2A1/COL3A1 | 6 |
| GO:0032154 | cleavage furrow | 15/2309 | 54/18698 | 0.00176382 | 0.023240927 | 0.020313968 | RAB11FIP3/RALA/SVIL/PPP1CC/PLEKHG6/PSD2/SPIRE2/SEPT12/MYLK/RHOB/PKN1/MYH10/RHOC/SSH1/HTR3A | 15 |
| GO:0030027 | lamellipodium | 37/2309 | 187/18698 | 0.002390362 | 0.030890838 | 0.027000451 | BCAR1/ABI3/CLRN1/SH3BP1/ACTG2/CSPG4/ACTA2/PPP1R9A/NME2/FAP/CTNNA3/PLEKHG5/DGKZ/FGD4/ARHGEF7/FLOT1/SRCIN1/TESC/MYO1G/DYSF/MTSS1L/MCC/ARAP3/APC2/MYLK/FSCN3/PDPN/PALLD/PARVB/KCNA2/SLC9A1/MYH10/SSH1/PTPRM/AMOTL1/PTPRO/PKD2 | 37 |
| GO:0009925 | basal plasma membrane | 11/2309 | 35/18698 | 0.002438916 | 0.03092362 | 0.027029104 | AQP1/MYO1C/EPS15/AQP5/KCNQ4/ITGA6/EGFR/TACSTD2/TF/LDLRAP1/PKD2 | 11 |
| GO:0031091 | platelet alpha granule | 21/2309 | 90/18698 | 0.00272166 | 0.033363695 | 0.029161876 | PHACTR2/ALDOA/VEGFA/APLP2/IGF2/ACTN2/SERPINF2/TGFB3/SELP/PDGFA/EGF/SERPING1/LHFPL2/CD36/VTI1B/CLU/LEFTY2/ITGA2B/VEGFB/CD109/OLA1 | 21 |
| GO:0031092 | platelet alpha granule membrane | 7/2309 | 17/18698 | 0.00273066 | 0.033363695 | 0.029161876 | PHACTR2/APLP2/SELP/LHFPL2/CD36/ITGA2B/CD109 | 7 |
| GO:0032982 | myosin filament | 8/2309 | 22/18698 | 0.003438778 | 0.041265336 | 0.036068386 | MYH7/ACTG2/MYOM2/MYH4/MYH14/MYOM1/MYH10/MYBPC1 | 8 |
| GO:0008076 | voltage-gated potassium channel complex | 21/2309 | 92/18698 | 0.003599387 | 0.042434878 | 0.037090635 | KCNAB1/KCNC1/KCNA6/KCNMB3/KCNS1/DLG2/VAMP2/KCNA7/HCN1/KCNQ4/KCNMB1/KCNA3/KCNA5/KCNJ14/KCNK2/KCNE3/KCNIP4/KCNK6/KCNG1/KCNA2/KCNIP3 | 21 |
| GO:0005859 | muscle myosin complex | 9/2309 | 27/18698 | 0.003828638 | 0.044359394 | 0.038772778 | OBSL1/MYH7/MYOM2/MYH4/MYL3/MYL9/MYOM3/MYOM1/MYBPC1 | 9 |
| GO:0034705 | potassium channel complex | 22/2309 | 99/18698 | 0.004142272 | 0.047179777 | 0.041237963 | KCNAB1/KCNC1/KCNA6/KCNMB3/KCNS1/DLG2/VAMP2/KCNA7/HCN1/KCNQ4/GRIK1/KCNMB1/KCNA3/KCNA5/KCNJ14/KCNK2/KCNE3/KCNIP4/KCNK6/KCNG1/KCNA2/KCNIP3 | 22 |
| GO:0030122 | AP-2 adaptor complex | 6/2309 | 14/18698 | 0.004372206 | 0.048968707 | 0.042801595 | SLC18A3/EPS15/TBC1D5/EGFR/SGIP1/LDLRAP1 | 6 |

| ID | Description | GeneRatio | BgRatio | pvalue | p.adjust | qvalue | geneID | Count |
| --- | --- | --- | --- | --- | --- | --- | --- | --- |
| GO:0003779 | actin binding | 96/2191 | 410/17548 | 4.73E-10 | 5.28E-07 | 4.74E-07 | OBSL1/PLS1/SPTBN5/EPS8L1/PHACTR2/MACF1/HNRNPU/ALDOA/MYO1C/MYL2/DNASE1/MYH7/PFN3/AVIL/FSCN2/USH1C/DAAM2/INF2/DBN1/CALD1/PDLIM5/SPTBN1/FLNA/KLHL4/SVIL/PPP1R9A/CORO6/MYOM2/MYO18B/CTNNA3/CNN1/MYOZ3/PHACTR1/STK38L/DIXDC1/CTNNAL1/MYH4/FGD4/SYNPO/MYL3/MICAL1/PACRG/ACTN2/MYH14/MYO7A/MYOM3/FLNC/MYO1G/ABRA/MIB2/GC/MYOZ2/MTSS1L/SPIRE2/PARVG/KLHL3/SYNPO2L/TNNI1/MYO7B/TPM4/MYO15A/MYOM1/SORBS1/PAWR/LSP1/TAGLN/NOS3/DIAPH2/PXK/IMPACT/MAP1A/WIPF3/MYO1B/TNNI2/RCSD1/SHROOM1/MYLK/FSCN3/EGFR/PALLD/TNS1/PARVB/CSRP3/TNNT2/VPS18/SPTBN2/ABL1/TNNT3/MYH10/MSN/CAP2/SSH1/LIMCH1/NEXN/MYBPC1/SHANK3 | 96 |
| GO:0015267 | channel activity | 98/2191 | 463/17548 | 7.57E-08 | 3.15E-05 | 2.83E-05 | GLRA1/PKD2L2/KCNAB1/KCNC1/AQP1/GRIA4/KCNE4/LRRC8B/P2RX1/CHRNA9/CHRNE/GJC2/KCNN3/SCN9A/GABRG3/ORAI1/AQP5/KCNA6/SLC26A10/RYR2/CLIC1/KCNMB3/FXYD6/SLC26A9/CATSPER1/TRPV2/DENND5B/KCNS1/CLCN6/FAM155A/KCNK3/AQP11/PKD2L1/KCNA7/CLCNKB/TRPV6/HCN1/CHRNA6/HTR3B/KCNK18/LRRC8C/CNGB3/KCNQ4/GRIK1/GLRB/JPH2/KCNMB1/KCNA3/GJA5/CFTR/BEST1/KCNA5/CLCA1/PDE2A/KCNJ14/SCN2B/TMEM109/SCNN1D/PKDREJ/PANX3/CACNA1B/FXYD1/KCNK2/SCNN1A/SLC14A1/TRPV5/SLC40A1/CLCNKA/CACNA2D1/TRPC4/SLC26A8/TRPV3/CLIC6/AQP4/KCNE3/CALM1/KCNIP4/SLC17A3/P2RX6/CNGA3/KCNJ9/CHRNB4/HVCN1/KCNK6/TRPM3/KCNG1/KCNA2/KCNIP3/GJA3/RHAG/GJA1/HTR3A/KCNJ15/GRIA2/AQP6/PKD2/SCN4B/BAX | 98 |
| GO:0022803 | passive transmembrane transporter activity | 98/2191 | 464/17548 | 8.45E-08 | 3.15E-05 | 2.83E-05 | GLRA1/PKD2L2/KCNAB1/KCNC1/AQP1/GRIA4/KCNE4/LRRC8B/P2RX1/CHRNA9/CHRNE/GJC2/KCNN3/SCN9A/GABRG3/ORAI1/AQP5/KCNA6/SLC26A10/RYR2/CLIC1/KCNMB3/FXYD6/SLC26A9/CATSPER1/TRPV2/DENND5B/KCNS1/CLCN6/FAM155A/KCNK3/AQP11/PKD2L1/KCNA7/CLCNKB/TRPV6/HCN1/CHRNA6/HTR3B/KCNK18/LRRC8C/CNGB3/KCNQ4/GRIK1/GLRB/JPH2/KCNMB1/KCNA3/GJA5/CFTR/BEST1/KCNA5/CLCA1/PDE2A/KCNJ14/SCN2B/TMEM109/SCNN1D/PKDREJ/PANX3/CACNA1B/FXYD1/KCNK2/SCNN1A/SLC14A1/TRPV5/SLC40A1/CLCNKA/CACNA2D1/TRPC4/SLC26A8/TRPV3/CLIC6/AQP4/KCNE3/CALM1/KCNIP4/SLC17A3/P2RX6/CNGA3/KCNJ9/CHRNB4/HVCN1/KCNK6/TRPM3/KCNG1/KCNA2/KCNIP3/GJA3/RHAG/GJA1/HTR3A/KCNJ15/GRIA2/AQP6/PKD2/SCN4B/BAX | 98 |
| GO:0022838 | substrate-specific channel activity | 90/2191 | 437/17548 | 8.96E-07 | 0.000250218 | 0.000224717 | GLRA1/PKD2L2/KCNAB1/KCNC1/AQP1/GRIA4/KCNE4/LRRC8B/P2RX1/CHRNA9/CHRNE/KCNN3/SCN9A/GABRG3/ORAI1/AQP5/KCNA6/SLC26A10/RYR2/CLIC1/KCNMB3/FXYD6/SLC26A9/CATSPER1/TRPV2/DENND5B/KCNS1/CLCN6/FAM155A/KCNK3/AQP11/PKD2L1/KCNA7/CLCNKB/TRPV6/HCN1/CHRNA6/HTR3B/KCNK18/LRRC8C/CNGB3/KCNQ4/GRIK1/GLRB/JPH2/KCNMB1/KCNA3/CFTR/BEST1/KCNA5/CLCA1/PDE2A/KCNJ14/SCN2B/SCNN1D/PKDREJ/CACNA1B/FXYD1/KCNK2/SCNN1A/SLC14A1/TRPV5/SLC40A1/CLCNKA/CACNA2D1/TRPC4/SLC26A8/TRPV3/CLIC6/AQP4/KCNE3/CALM1/KCNIP4/SLC17A3/P2RX6/CNGA3/KCNJ9/CHRNB4/HVCN1/KCNK6/TRPM3/KCNG1/KCNA2/KCNIP3/HTR3A/KCNJ15/GRIA2/AQP6/PKD2/SCN4B | 90 |
| GO:0005216 | ion channel activity | 86/2191 | 424/17548 | 2.97E-06 | 0.000664063 | 0.000596383 | GLRA1/PKD2L2/KCNAB1/KCNC1/AQP1/GRIA4/KCNE4/LRRC8B/P2RX1/CHRNA9/CHRNE/KCNN3/SCN9A/GABRG3/ORAI1/KCNA6/SLC26A10/RYR2/CLIC1/KCNMB3/FXYD6/SLC26A9/CATSPER1/TRPV2/DENND5B/KCNS1/CLCN6/FAM155A/KCNK3/PKD2L1/KCNA7/CLCNKB/TRPV6/HCN1/CHRNA6/HTR3B/KCNK18/LRRC8C/CNGB3/KCNQ4/GRIK1/GLRB/JPH2/KCNMB1/KCNA3/CFTR/BEST1/KCNA5/CLCA1/PDE2A/KCNJ14/SCN2B/SCNN1D/PKDREJ/CACNA1B/FXYD1/KCNK2/SCNN1A/TRPV5/SLC40A1/CLCNKA/CACNA2D1/TRPC4/SLC26A8/TRPV3/CLIC6/KCNE3/CALM1/KCNIP4/SLC17A3/P2RX6/CNGA3/KCNJ9/CHRNB4/HVCN1/KCNK6/TRPM3/KCNG1/KCNA2/KCNIP3/HTR3A/KCNJ15/GRIA2/AQP6/PKD2/SCN4B | 86 |
| GO:0005520 | insulin-like growth factor binding | 13/2191 | 29/17548 | 1.68E-05 | 0.003121379 | 0.002803255 | IGFBP1/WISP2/IGFBP5/CRIM1/WISP1/HTRA4/IGFALS/ESM1/KAZALD1/ITGA6/CYR61/CTGF/INSR | 13 |
| GO:0005261 | cation channel activity | 65/2191 | 316/17548 | 2.95E-05 | 0.004254023 | 0.003820463 | PKD2L2/KCNAB1/KCNC1/AQP1/GRIA4/KCNE4/P2RX1/CHRNA9/CHRNE/KCNN3/SCN9A/ORAI1/KCNA6/RYR2/KCNMB3/CATSPER1/TRPV2/DENND5B/KCNS1/FAM155A/KCNK3/PKD2L1/KCNA7/TRPV6/HCN1/CHRNA6/HTR3B/KCNK18/CNGB3/KCNQ4/GRIK1/JPH2/KCNMB1/KCNA3/KCNA5/PDE2A/KCNJ14/SCN2B/SCNN1D/PKDREJ/CACNA1B/KCNK2/SCNN1A/TRPV5/SLC40A1/CACNA2D1/TRPC4/TRPV3/KCNE3/KCNIP4/P2RX6/CNGA3/KCNJ9/CHRNB4/HVCN1/KCNK6/TRPM3/KCNG1/KCNA2/KCNIP3/HTR3A/KCNJ15/GRIA2/PKD2/SCN4B | 65 |
| GO:0022839 | ion gated channel activity | 68/2191 | 335/17548 | 3.05E-05 | 0.004254023 | 0.003820463 | GLRA1/KCNAB1/KCNC1/AQP1/GRIA4/KCNE4/P2RX1/CHRNA9/CHRNE/KCNN3/SCN9A/GABRG3/KCNA6/RYR2/CLIC1/KCNMB3/CATSPER1/KCNS1/CLCN6/FAM155A/KCNK3/PKD2L1/KCNA7/CLCNKB/HCN1/CHRNA6/HTR3B/KCNK18/CNGB3/KCNQ4/GRIK1/GLRB/JPH2/KCNMB1/KCNA3/CFTR/BEST1/KCNA5/CLCA1/KCNJ14/SCN2B/TMEM109/SCNN1D/CACNA1B/KCNK2/SCNN1A/CLCNKA/CACNA2D1/CLIC6/KCNE3/CALM1/KCNIP4/SLC17A3/P2RX6/CNGA3/KCNJ9/CHRNB4/HVCN1/KCNK6/TRPM3/KCNG1/KCNA2/KCNIP3/HTR3A/KCNJ15/GRIA2/PKD2/SCN4B | 68 |
| GO:0008519 | ammonium transmembrane transporter activity | 13/2191 | 31/17548 | 4.00E-05 | 0.004565919 | 0.004100571 | SLC18A2/AQP1/SLC18A3/OAZ3/SLC25A2/SLC22A2/RHBG/SLC44A2/SLC22A1/SLC5A7/RHCG/RHAG/SLC22A3 | 13 |
| GO:0022836 | gated channel activity | 68/2191 | 338/17548 | 4.09E-05 | 0.004565919 | 0.004100571 | GLRA1/KCNAB1/KCNC1/AQP1/GRIA4/KCNE4/P2RX1/CHRNA9/CHRNE/KCNN3/SCN9A/GABRG3/KCNA6/RYR2/CLIC1/KCNMB3/CATSPER1/KCNS1/CLCN6/FAM155A/KCNK3/PKD2L1/KCNA7/CLCNKB/HCN1/CHRNA6/HTR3B/KCNK18/CNGB3/KCNQ4/GRIK1/GLRB/JPH2/KCNMB1/KCNA3/CFTR/BEST1/KCNA5/CLCA1/KCNJ14/SCN2B/TMEM109/SCNN1D/CACNA1B/KCNK2/SCNN1A/CLCNKA/CACNA2D1/CLIC6/KCNE3/CALM1/KCNIP4/SLC17A3/P2RX6/CNGA3/KCNJ9/CHRNB4/HVCN1/KCNK6/TRPM3/KCNG1/KCNA2/KCNIP3/HTR3A/KCNJ15/GRIA2/PKD2/SCN4B | 68 |
| GO:0019838 | growth factor binding | 33/2191 | 133/17548 | 7.11E-05 | 0.007215387 | 0.006480011 | WFIKKN2/IGFBP1/WISP2/IGFBP5/CRIM1/PXDN/CSF1R/VASN/WISP1/IL1RN/HTRA4/IGFALS/ESM1/KAZALD1/LTBP2/TGFB3/LTBP3/COL1A2/COL2A1/ITGA6/PDGFA/CXCL13/CYR61/LTBP1/EGFR/CTGF/CD36/INSR/KLB/COL3A1/SORT1/CD109/HYAL2 | 33 |
| GO:0008307 | structural constituent of muscle | 16/2191 | 46/17548 | 8.17E-05 | 0.007376574 | 0.006624771 | OBSL1/MYL2/KRT19/MYOM2/TCAP/MYL3/OBSCN/ACTN2/MYL9/MYOM3/TPM4/MYOM1/CSRP3/ANKRD2/NEXN/MYBPC1 | 16 |
| GO:0031432 | titin binding | 8/2191 | 14/17548 | 8.70E-05 | 0.007376574 | 0.006624771 | TRIM63/TCAP/OBSCN/ACTN2/ANKRD23/CALM1/ANKRD2/MYBPC1 | 8 |
| GO:0005178 | integrin binding | 30/2191 | 118/17548 | 9.25E-05 | 0.007376574 | 0.006624771 | WISP2/TNXB/GFAP/SFRP2/CX3CL1/WISP1/EMILIN1/TSPAN8/FAP/LGALS8/TSPAN4/ITGA2/ACTN2/ADAM11/ESM1/CXCL12/TGFBI/ITGA6/IBSP/FGF2/MMP14/CYR61/FBLN5/EGFR/GPNMB/CTGF/IL1B/CD226/COL3A1/COL16A1 | 30 |
| GO:0051371 | muscle alpha-actinin binding | 9/2191 | 18/17548 | 0.000122088 | 0.009091518 | 0.008164931 | OBSL1/LDB3/MYOM2/PKD2L1/MYOM3/MYOM1/PALLD/MYBPC1/PKD2 | 9 |
| GO:0001653 | peptide receptor activity | 32/2191 | 134/17548 | 0.00019535 | 0.013618657 | 0.012230674 | CMKLR1/EDNRA/F2RL2/GPR37L1/NPY2R/RAMP1/GPR75/TSHR/MC4R/PRLHR/SSTR3/MC2R/SSTR5/MC1R/F2RL3/CCRL2/CYSLTR2/NPFFR1/NPR2/NTSR2/CCR9/AVPR1A/GP1BA/CCR10/CXCR5/GLP2R/SSTR4/CCR3/PROKR1/CXCR6/UTS2R/CALCRL | 32 |
| GO:0005267 | potassium channel activity | 31/2191 | 129/17548 | 0.000216392 | 0.013618657 | 0.012230674 | KCNAB1/KCNC1/AQP1/KCNE4/KCNN3/KCNA6/KCNMB3/KCNS1/KCNK3/PKD2L1/KCNA7/HCN1/KCNK18/CNGB3/KCNQ4/GRIK1/KCNMB1/KCNA3/KCNA5/KCNJ14/KCNK2/KCNE3/KCNIP4/CNGA3/KCNJ9/KCNK6/KCNG1/KCNA2/KCNIP3/KCNJ15/PKD2 | 31 |
| GO:0038024 | cargo receptor activity | 23/2191 | 85/17548 | 0.000219459 | 0.013618657 | 0.012230674 | TFR2/SUSD2/FOLR1/SCARA5/LOXL4/LGALS3BP/TMPRSS5/TMPRSS13/ASGR1/LOXL2/CRB3/LRP1/LDLR/STAB2/CD5L/SCARA3/TINAGL1/LRP8/CD36/ENPP3/LDLRAP1/ASGR2/SCARB1 | 23 |
| GO:0015079 | potassium ion transmembrane transporter activity | 37/2191 | 166/17548 | 0.000289015 | 0.016991068 | 0.015259377 | KCNAB1/KCNC1/AQP1/KCNE4/ATP1A2/KCNN3/KCNA6/KCNMB3/KCNS1/KCNK3/PKD2L1/KCNA7/HCN1/KCNK18/CNGB3/KCNQ4/ATP1A4/GRIK1/KCNMB1/KCNA3/SLC12A4/KCNA5/KCNJ14/SLC12A6/KCNK2/SLC12A8/KCNE3/KCNIP4/CNGA3/KCNJ9/KCNK6/KCNG1/KCNA2/SLC9A1/KCNIP3/KCNJ15/PKD2 | 37 |
| GO:0042805 | actinin binding | 14/2191 | 42/17548 | 0.000373787 | 0.020456786 | 0.018371876 | OBSL1/LDB3/PDLIM5/RARA/MYOM2/PKD2L1/MYOM3/KCNA5/MYOM1/ADORA2A/PALLD/CSRP3/MYBPC1/PKD2 | 14 |
| GO:0008528 | G-protein coupled peptide receptor activity | 31/2191 | 133/17548 | 0.000384595 | 0.020456786 | 0.018371876 | CMKLR1/EDNRA/F2RL2/GPR37L1/NPY2R/RAMP1/GPR75/TSHR/MC4R/PRLHR/SSTR3/MC2R/SSTR5/MC1R/F2RL3/CCRL2/CYSLTR2/NPFFR1/NTSR2/CCR9/AVPR1A/GP1BA/CCR10/CXCR5/GLP2R/SSTR4/CCR3/PROKR1/CXCR6/UTS2R/CALCRL | 31 |
| GO:0051393 | alpha-actinin binding | 12/2191 | 34/17548 | 0.000538344 | 0.027333175 | 0.02454744 | OBSL1/LDB3/RARA/MYOM2/PKD2L1/MYOM3/KCNA5/MYOM1/ADORA2A/PALLD/MYBPC1/PKD2 | 12 |
| GO:0005539 | glycosaminoglycan binding | 44/2191 | 215/17548 | 0.000598251 | 0.029054183 | 0.026093047 | FSTL1/WISP2/ABI3BP/SPOCK2/TNXB/VEGFA/LIPC/APLP2/SOST/ELANE/NAV2/SERPIND1/WISP1/LAYN/SFRP1/APOB/SPOCK3/PRELP/MDK/SOD3/LTBP2/SELP/VIT/BMP4/CLEC3B/STAB2/FGF2/CXCL13/HAPLN4/CYR61/APOE/CEL/EGFLAM/HK1/GPNMB/CTGF/PGLYRP2/SMOC2/RPL22/LXN/COL13A1/VEGFB/PF4V1/HYAL2 | 44 |
| GO:0005044 | scavenger receptor activity | 16/2191 | 54/17548 | 0.000655704 | 0.030517553 | 0.027407273 | SUSD2/SCARA5/LOXL4/LGALS3BP/TMPRSS5/TMPRSS13/LOXL2/CRB3/LRP1/STAB2/CD5L/SCARA3/TINAGL1/CD36/ENPP3/SCARB1 | 16 |
| GO:0005080 | protein kinase C binding | 15/2191 | 50/17548 | 0.0008297 | 0.037070983 | 0.033292792 | HDAC9/LDB3/ADCY4/PDLIM5/PLEK/FEZ1/AKT1/HSPB1/PARD6B/AVPR1A/ADCY6/PKN1/ABL1/DACT1/HDAC7 | 15 |
| GO:0008201 | heparin binding | 34/2191 | 158/17548 | 0.000965486 | 0.04109076 | 0.036902883 | FSTL1/WISP2/ABI3BP/TNXB/VEGFA/LIPC/APLP2/SOST/ELANE/NAV2/SERPIND1/WISP1/SFRP1/APOB/PRELP/MDK/SOD3/LTBP2/SELP/BMP4/CLEC3B/FGF2/CXCL13/CYR61/APOE/CEL/GPNMB/CTGF/SMOC2/RPL22/LXN/COL13A1/VEGFB/PF4V1 | 34 |
| GO:0046873 | metal ion transmembrane transporter activity | 80/2191 | 457/17548 | 0.001025618 | 0.04109076 | 0.036902883 | PKD2L2/KCNAB1/KCNC1/AQP1/KCNE4/ATP1A2/SLC6A13/CHRNA9/KCNN3/SCN9A/TFR2/SLC25A18/ORAI1/KCNA6/RYR2/KCNMB3/SLC6A9/SLC39A10/CATSPER1/TRPV2/DENND5B/KCNS1/FAM155A/KCNK3/PKD2L1/KCNA7/TRPV6/HCN1/KCNK18/CNGB3/KCNQ4/ATP1A4/GRIK1/JPH2/KCNMB1/KCNA3/SLC28A1/SLC6A6/SLC12A4/SLC34A2/KCNA5/PDE2A/KCNJ14/SCN2B/SCNN1D/SLC12A6/PKDREJ/CACNA1B/KCNK2/SLC10A6/SCNN1A/TRPV5/LETM1/SLC12A8/SLC40A1/CACNA2D1/TRPC4/TRPV3/KCNE3/SLC5A7/TF/KCNIP4/SLC17A3/CNNM2/CNGA3/KCNJ9/KCNK6/TRPM3/KCNG1/KCNA2/SLC6A11/SLC9A1/KCNIP3/SLC30A1/KCNJ15/SLC8A2/PKD2/SLC39A2/SLC41A2/SCN4B | 80 |
| GO:0005249 | voltage-gated potassium channel activity | 23/2191 | 94/17548 | 0.001030028 | 0.04109076 | 0.036902883 | KCNAB1/KCNC1/KCNE4/KCNA6/KCNS1/KCNK3/KCNA7/HCN1/KCNK18/CNGB3/KCNQ4/KCNA3/KCNA5/KCNJ14/KCNK2/KCNE3/CNGA3/KCNJ9/KCNK6/KCNG1/KCNA2/KCNJ15/PKD2 | 23 |
| GO:0099106 | ion channel regulator activity | 23/2191 | 95/17548 | 0.001201348 | 0.044281748 | 0.039768653 | GRM7/KCNAB1/GRM2/NPY2R/FLNA/PRKCZ/KCNMB3/PRKG1/KCNS1/KCNMB1/SGK1/LYNX1/CFTR/KCNA5/NEDD4/NRXN2/KCNE3/CALM1/KCNIP4/VTI1B/NEDD4L/KCNIP3/SLC30A1 | 23 |
| GO:1901681 | sulfur compound binding | 46/2191 | 235/17548 | 0.00122401 | 0.044281748 | 0.039768653 | GLRA1/FSTL1/WISP2/ABI3BP/TNXB/VEGFA/LIPC/APLP2/ACADL/SOST/ELANE/NAV2/SERPIND1/WISP1/SFRP1/APOB/HLCS/RYR2/ITGA2/PRELP/LRP1/MDK/SOD3/LTBP2/SELP/BMP4/CLEC3B/MGST1/FGF20/FGF2/ACBD7/CXCL13/GSTM2/DLD/CYR61/APOE/CEL/GPNMB/CTGF/SMOC2/RPL22/SCP2/LXN/COL13A1/VEGFB/PF4V1 | 46 |
| GO:0050431 | transforming growth factor beta binding | 8/2191 | 19/17548 | 0.001228947 | 0.044281748 | 0.039768653 | WFIKKN2/VASN/TGFB3/LTBP3/LTBP1/CD36/CD109/HYAL2 | 8 |
| GO:0050840 | extracellular matrix binding | 15/2191 | 52/17548 | 0.001297304 | 0.045284005 | 0.040668762 | COL11A1/CD248/SPOCK2/PLEKHA2/ECM1/VEGFA/LYPD3/ITGA2/SPARCL1/TGFBI/ITGA6/CYR61/TINAGL1/ITGA2B/ANXA2 | 15 |

| ID | Description | GeneRatio | BgRatio | pvalue | p.adjust | qvalue | geneID | Count |
| --- | --- | --- | --- | --- | --- | --- | --- | --- |
| hsa04371 | Apelin signaling pathway | 36/1038 | 137/8106 | 1.40E-05 | 0.004563234 | 0.00403956 | 187/91860/85366/196883/4633/53632/2771/5566/94235/59/6262/5140/5330/4634/207/4843/5595/111/5346/5327/4846/2790/7350/112/4638/1490/801/4854/146850/6548/3845/11337/2786/10672/6543/10891 | 36 |
| hsa04270 | Vascular smooth muscle contraction | 34/1038 | 133/8106 | 4.45E-05 | 0.007275435 | 0.006440511 | 1909/91860/85366/196883/10267/5566/72/800/10335/59/27094/5592/5501/796/5330/50487/79784/10398/4882/148/3779/5595/111/552/135/5499/112/4638/4880/1908/801/10203/4628/10672 | 34 |
| hsa04080 | Neuroactive ligand-receptor interaction | 66/1038 | 341/8106 | 0.000302111 | 0.022857726 | 0.020234588 | 3640/2917/2741/187/3351/1909/2151/2893/2912/4887/283869/5023/55584/7253/1145/1128/4160/2567/2834/6753/4295/4158/6755/59350/5745/4157/7201/152/7349/5618/796/9002/6915/57105/64106/8973/57152/3274/2897/2743/3952/148/319100/7434/3269/4922/23620/3356/552/1813/135/9340/6754/5173/8698/1908/885/9127/2837/1143/10203/6750/2550/5732/2891/6343 | 66 |
| hsa04926 | Relaxin signaling pathway | 31/1038 | 129/8106 | 0.000318571 | 0.022857726 | 0.020234588 | 3640/6300/196883/2769/7422/2771/5566/94235/9586/6416/5590/59/59350/1388/5330/207/4843/5595/111/1278/5602/4846/2790/112/1956/4318/3845/1281/2786/7423/399694 | 31 |
| hsa04611 | Platelet activation | 30/1038 | 124/8106 | 0.000349507 | 0.022857726 | 0.020234588 | 6300/85366/196883/5023/2771/5566/2815/84876/5590/2812/5592/5501/3673/9002/6915/5330/207/5595/111/3937/1278/2811/4846/5499/112/4638/146850/1281/10672/3674 | 30 |
| hsa04915 | Estrogen signaling pathway | 32/1038 | 138/8106 | 0.000506804 | 0.027620808 | 0.024451062 | 91860/3310/196883/2771/5566/9586/3884/5914/3880/2099/1388/5330/207/5595/3866/111/3306/4846/3868/3860/25984/112/1509/1956/801/3765/4318/2550/2100/3845/192666/399694 | 32 |
| hsa04976 | Bile secretion | 23/1038 | 90/8106 | 0.000738751 | 0.034510218 | 0.030549847 | 79799/358/196883/477/5566/8431/64241/7364/480/3949/111/1080/6580/57552/112/361/7366/6548/6343/2052/949/8714/10720 | 23 |
| hsa04022 | cGMP-PKG signaling pathway | 36/1038 | 167/8106 | 0.001001498 | 0.037289643 | 0.033010307 | 1909/91860/85366/196883/4625/477/2771/9586/10335/5140/27094/5592/1388/152/5501/5330/207/10398/480/4882/148/3779/5595/111/5138/4846/5499/112/4638/4880/801/3643/146850/8660/10672/6543 | 36 |
| hsa04024 | cAMP signaling pathway | 44/1038 | 216/8106 | 0.00102632 | 0.037289643 | 0.033010307 | 3351/1909/91860/2893/196883/477/2771/5566/7253/1128/9586/84876/6262/5142/5140/4158/6755/5501/207/10398/54714/480/5595/111/7434/1080/64411/5348/5602/1813/135/5499/51196/112/5337/1908/801/1261/6750/2550/5732/2867/6548/2891 | 44 |
