# Supplementary material for: Integrated Analysis of Gene Expression and Methylation Data to Identify Potential Biomarkers Related to Atherosclerosis Onset
Source: Oxid Med Cell Longev. 2022 Jul 22;2022:5493051. doi: 10.1155/2022/5493051 (PMC9338736; doi:10.1155/2022/5493051)
Supplement: Supplementary 8 — Table S5: the detailed functional enrichment results of the 132 DEGs. [file 5493051.f8.docx]

**Table S5** The detailed functional enrichment results of the 132 DEGs.

| ID | Description | | | GeneRatio | | BgRatio | | | pvalue | p.adjust | qvalue | | geneID | Count |  |
| --- | --- | --- | --- | --- | --- | --- | --- | --- | --- | --- | --- | --- | --- | --- | --- |
| GO:0006958 | complement activation, classical pathway | | | 14/107 | | 128/17653 | | | 3.70E-14 | 7.32E-11 | 6.30E-11 | | IGKV1D-33/IGHV3-74/IGKC/C2/IGHV3-7/IGHV3-43/IGHV4-59/IGKV3D-11/IGHV3-73/IGKV3D-20/IGKV2D-28/C7/CR1/IGHV1OR15-1 | 14 |  |
| GO:0002455 | humoral immune response mediated by circulating immunoglobulin | | | 14/107 | | 139/17653 | | | 1.18E-13 | 1.17E-10 | 1.00E-10 | | IGKV1D-33/IGHV3-74/IGKC/C2/IGHV3-7/IGHV3-43/IGHV4-59/IGKV3D-11/IGHV3-73/IGKV3D-20/IGKV2D-28/C7/CR1/IGHV1OR15-1 | 14 |  |
| GO:0006956 | complement activation | | | 14/107 | | 159/17653 | | | 7.66E-13 | 5.06E-10 | 4.35E-10 | | IGKV1D-33/IGHV3-74/IGKC/C2/IGHV3-7/IGHV3-43/IGHV4-59/IGKV3D-11/IGHV3-73/IGKV3D-20/IGKV2D-28/C7/CR1/IGHV1OR15-1 | 14 |  |
| GO:0072376 | protein activation cascade | | | 14/107 | | 182/17653 | | | 4.89E-12 | 2.42E-09 | 2.08E-09 | | IGKV1D-33/IGHV3-74/IGKC/C2/IGHV3-7/IGHV3-43/IGHV4-59/IGKV3D-11/IGHV3-73/IGKV3D-20/IGKV2D-28/C7/CR1/IGHV1OR15-1 | 14 |  |
| GO:0016064 | immunoglobulin mediated immune response | | | 14/107 | | 197/17653 | | | 1.43E-11 | 5.06E-09 | 4.35E-09 | | IGKV1D-33/IGHV3-74/IGKC/C2/IGHV3-7/IGHV3-43/IGHV4-59/IGKV3D-11/IGHV3-73/IGKV3D-20/IGKV2D-28/C7/CR1/IGHV1OR15-1 | 14 |  |
| GO:0019724 | B cell mediated immunity | | | 14/107 | | 198/17653 | | | 1.53E-11 | 5.06E-09 | 4.35E-09 | | IGKV1D-33/IGHV3-74/IGKC/C2/IGHV3-7/IGHV3-43/IGHV4-59/IGKV3D-11/IGHV3-73/IGKV3D-20/IGKV2D-28/C7/CR1/IGHV1OR15-1 | 14 |  |
| GO:0030449 | regulation of complement activation | | | 11/107 | | 107/17653 | | | 4.58E-11 | 1.12E-08 | 9.61E-09 | | IGKV1D-33/IGKC/C2/IGHV3-7/IGHV4-59/IGKV3D-11/IGKV3D-20/IGKV2D-28/C7/CR1/IGHV1OR15-1 | 11 |  |
| GO:0002526 | acute inflammatory response | | | 14/107 | | 215/17653 | | | 4.64E-11 | 1.12E-08 | 9.61E-09 | | IL31RA/CD163/IGKV1D-33/IL1RN/IGKC/C2/IGHV3-7/IGHV4-59/IGKV3D-11/IGKV3D-20/IGKV2D-28/C7/CR1/IGHV1OR15-1 | 14 |  |
| GO:2000257 | regulation of protein activation cascade | | | 11/107 | | 108/17653 | | | 5.08E-11 | 1.12E-08 | 9.61E-09 | | IGKV1D-33/IGKC/C2/IGHV3-7/IGHV4-59/IGKV3D-11/IGKV3D-20/IGKV2D-28/C7/CR1/IGHV1OR15-1 | 11 |  |
| GO:0002920 | regulation of humoral immune response | | | 11/107 | | 125/17653 | | | 2.51E-10 | 4.96E-08 | 4.27E-08 | | IGKV1D-33/IGKC/C2/IGHV3-7/IGHV4-59/IGKV3D-11/IGKV3D-20/IGKV2D-28/C7/CR1/IGHV1OR15-1 | 11 |  |
| GO:0002460 | adaptive immune response based on somatic recombination of immune receptors built from immunoglobulin superfamily domains | | | 15/107 | | 314/17653 | | | 6.99E-10 | 1.21E-07 | 1.04E-07 | | IL31RA/IGKV1D-33/IGHV3-74/IGKC/C2/IGHV3-7/IGHV3-43/IGHV4-59/IGKV3D-11/IGHV3-73/IGKV3D-20/IGKV2D-28/C7/CR1/IGHV1OR15-1 | 15 |  |
| GO:0002449 | lymphocyte mediated immunity | | | 15/107 | | 315/17653 | | | 7.30E-10 | 1.21E-07 | 1.04E-07 | | IL31RA/IGKV1D-33/IGHV3-74/IGKC/C2/IGHV3-7/IGHV3-43/IGHV4-59/IGKV3D-11/IGHV3-73/IGKV3D-20/IGKV2D-28/C7/CR1/IGHV1OR15-1 | 15 |  |
| GO:0006959 | humoral immune response | | | 15/107 | | 329/17653 | | | 1.33E-09 | 2.02E-07 | 1.74E-07 | | IGKV1D-33/IGHV3-74/IGKC/C2/IGHV3-7/IGHV3-43/IGHV4-59/IGKV3D-11/IGHV3-73/IGKV3D-20/IGKV2D-28/C7/CR1/IGHV1OR15-1/ITLN1 | 15 |  |
| GO:0002673 | regulation of acute inflammatory response | | | 11/107 | | 149/17653 | | | 1.65E-09 | 2.34E-07 | 2.01E-07 | | IGKV1D-33/IGKC/C2/IGHV3-7/IGHV4-59/IGKV3D-11/IGKV3D-20/IGKV2D-28/C7/CR1/IGHV1OR15-1 | 11 |  |
| GO:0070613 | regulation of protein processing | | | 11/107 | | 167/17653 | | | 5.52E-09 | 7.29E-07 | 6.27E-07 | | IGKV1D-33/IGKC/C2/IGHV3-7/IGHV4-59/IGKV3D-11/IGKV3D-20/IGKV2D-28/C7/CR1/IGHV1OR15-1 | 11 |  |
| GO:1903317 | regulation of protein maturation | | | 11/107 | | 169/17653 | | | 6.26E-09 | 7.75E-07 | 6.66E-07 | | IGKV1D-33/IGKC/C2/IGHV3-7/IGHV4-59/IGKV3D-11/IGKV3D-20/IGKV2D-28/C7/CR1/IGHV1OR15-1 | 11 |  |
| GO:0006909 | phagocytosis | | | 14/107 | | 330/17653 | | | 1.23E-08 | 1.43E-06 | 1.23E-06 | | VAV3/IGKV1D-33/IGHV3-74/IGKC/C2/IGHV3-7/IGHV3-43/IGHV4-59/IGKV3D-11/IGHV3-73/IGKV3D-20/CD36/IGKV2D-28/IGHV1OR15-1 | 14 |  |
| GO:0050900 | leukocyte migration | | | 16/107 | | 470/17653 | | | 2.32E-08 | 2.55E-06 | 2.19E-06 | | VAV3/CCR1/HMOX1/IGKV1D-33/IGKC/IGHV3-7/IGHV4-59/IGKV3D-11/IGKV3D-20/MMP9/ITGAX/IGKV2D-28/SELE/PLA2G7/CXCL10/IGHV1OR15-1 | 16 |  |
| GO:0050727 | regulation of inflammatory response | | | 15/107 | | 411/17653 | | | 2.66E-08 | 2.77E-06 | 2.38E-06 | | FABP4/IGKV1D-33/IGKC/C2/IGHV3-7/IGHV4-59/IGKV3D-11/IGKV3D-20/IGKV2D-28/SELE/PLA2G7/ACP5/C7/CR1/IGHV1OR15-1 | 15 |  |
| GO:0006898 | receptor-mediated endocytosis | | | 13/107 | | 330/17653 | | | 1.01E-07 | 9.66E-06 | 8.30E-06 | | CD163/IGKV1D-33/IGKC/SBSPON/IGHV3-7/IGHV4-59/IGKV3D-11/IGKV3D-20/CD36/IGKV2D-28/SELE/GRIA1/IGHV1OR15-1 | 13 |  |
| GO:0002433 | immune response-regulating cell surface receptor signaling pathway involved in phagocytosis | | | 9/107 | | 132/17653 | | | 1.07E-07 | 9.66E-06 | 8.30E-06 | | VAV3/IGKV1D-33/IGKC/IGHV3-7/IGHV4-59/IGKV3D-11/IGKV3D-20/IGKV2D-28/IGHV1OR15-1 | 9 |  |
| GO:0038096 | Fc-gamma receptor signaling pathway involved in phagocytosis | | | 9/107 | | 132/17653 | | | 1.07E-07 | 9.66E-06 | 8.30E-06 | | VAV3/IGKV1D-33/IGKC/IGHV3-7/IGHV4-59/IGKV3D-11/IGKV3D-20/IGKV2D-28/IGHV1OR15-1 | 9 |  |
| GO:0038094 | Fc-gamma receptor signaling pathway | | | 9/107 | | 136/17653 | | | 1.39E-07 | 1.19E-05 | 1.03E-05 | | VAV3/IGKV1D-33/IGKC/IGHV3-7/IGHV4-59/IGKV3D-11/IGKV3D-20/IGKV2D-28/IGHV1OR15-1 | 9 |  |
| GO:0002431 | Fc receptor mediated stimulatory signaling pathway | | | 9/107 | | 137/17653 | | | 1.48E-07 | 1.22E-05 | 1.05E-05 | | VAV3/IGKV1D-33/IGKC/IGHV3-7/IGHV4-59/IGKV3D-11/IGKV3D-20/IGKV2D-28/IGHV1OR15-1 | 9 |  |
| GO:0002697 | regulation of immune effector process | | | 14/107 | | 405/17653 | | | 1.57E-07 | 1.24E-05 | 1.07E-05 | | HMOX1/IGKV1D-33/IGKC/C2/IGHV3-7/IGHV4-59/IGKV3D-11/IGKV3D-20/CD36/IGKV2D-28/C7/CR1/IGHV1OR15-1/MMP12 | 14 |  |
| GO:0016485 | protein processing | | | 12/107 | | 329/17653 | | | 7.31E-07 | 5.57E-05 | 4.79E-05 | | IGKV1D-33/IGKC/C2/IGHV3-7/MME/IGHV4-59/IGKV3D-11/IGKV3D-20/IGKV2D-28/C7/CR1/IGHV1OR15-1 | 12 |  |
| GO:0038095 | Fc-epsilon receptor signaling pathway | | | 9/107 | | 179/17653 | | | 1.42E-06 | 0.000103889 | 8.93E-05 | | VAV3/IGKV1D-33/IGKC/IGHV3-7/IGHV4-59/IGKV3D-11/IGKV3D-20/IGKV2D-28/IGHV1OR15-1 | 9 |  |
| GO:0006586 | indolalkylamine metabolic process | | | 4/107 | | 17/17653 | | | 2.86E-06 | 0.000202158 | 0.000173805 | | TPH1/TDO2/KMO/KYNU | 4 |  |
| GO:0002429 | immune response-activating cell surface receptor signaling pathway | | | 13/107 | | 450/17653 | | | 3.32E-06 | 0.000226499 | 0.000194732 | | VAV3/IGKV1D-33/IGHV3-74/IGKC/IGHV3-7/IGHV3-43/IGHV4-59/IGKV3D-11/IGHV3-73/IGKV3D-20/IGKV2D-28/CR1/IGHV1OR15-1 | 13 |  |
| GO:0051604 | protein maturation | | | 12/107 | | 390/17653 | | | 4.28E-06 | 0.000282616 | 0.000242978 | | IGKV1D-33/IGKC/C2/IGHV3-7/MME/IGHV4-59/IGKV3D-11/IGKV3D-20/IGKV2D-28/C7/CR1/IGHV1OR15-1 | 12 |  |
| GO:0002768 | immune response-regulating cell surface receptor signaling pathway | | | 13/107 | | 482/17653 | | | 6.98E-06 | 0.000446055 | 0.000383494 | | VAV3/IGKV1D-33/IGHV3-74/IGKC/IGHV3-7/IGHV3-43/IGHV4-59/IGKV3D-11/IGHV3-73/IGKV3D-20/IGKV2D-28/CR1/IGHV1OR15-1 | 13 |  |
| GO:0042430 | indole-containing compound metabolic process | | | 4/107 | | 24/17653 | | | 1.23E-05 | 0.000764364 | 0.000657159 | | TPH1/TDO2/KMO/KYNU | 4 |  |
| GO:0038093 | Fc receptor signaling pathway | | | 9/107 | | 246/17653 | | | 1.87E-05 | 0.001124425 | 0.00096672 | | VAV3/IGKV1D-33/IGKC/IGHV3-7/IGHV4-59/IGKV3D-11/IGKV3D-20/IGKV2D-28/IGHV1OR15-1 | 9 |  |
| GO:0048662 | negative regulation of smooth muscle cell proliferation | | | 5/107 | | 55/17653 | | | 2.04E-05 | 0.001186127 | 0.001019769 | | CNN1/MYOCD/NPR3/HMOX1/NPR1 | 5 |  |
| GO:0006569 | tryptophan catabolic process | | | 3/107 | | 10/17653 | | | 2.52E-05 | 0.001313182 | 0.001129004 | | TDO2/KMO/KYNU | 3 |  |
| GO:0042436 | indole-containing compound catabolic process | | | 3/107 | | 10/17653 | | | 2.52E-05 | 0.001313182 | 0.001129004 | | TDO2/KMO/KYNU | 3 |  |
| GO:0046218 | indolalkylamine catabolic process | | | 3/107 | | 10/17653 | | | 2.52E-05 | 0.001313182 | 0.001129004 | | TDO2/KMO/KYNU | 3 |  |
| GO:0070189 | kynurenine metabolic process | | | 3/107 | | 10/17653 | | | 2.52E-05 | 0.001313182 | 0.001129004 | | TDO2/KMO/KYNU | 3 |  |
| GO:0009072 | aromatic amino acid family metabolic process | | | 4/107 | | 29/17653 | | | 2.70E-05 | 0.001369524 | 0.001177443 | | TPH1/TDO2/KMO/KYNU | 4 |  |
| GO:0006910 | phagocytosis, recognition | | | 5/107 | | 60/17653 | | | 3.12E-05 | 0.001545276 | 0.001328545 | | IGHV3-74/IGKC/IGHV3-43/IGHV3-73/CD36 | 5 |  |
| GO:0006568 | tryptophan metabolic process | | | 3/107 | | 12/17653 | | | 4.58E-05 | 0.0022117 | 0.001901502 | | TDO2/KMO/KYNU | 3 |  |
| GO:0002440 | production of molecular mediator of immune response | | | 8/107 | | 223/17653 | | | 6.38E-05 | 0.00300741 | 0.00258561 | | IL31RA/HMOX1/IGKC/IGKV3D-11/IGKV1D-43/IGKV3D-20/CD36/IGKV2D-26 | 8 |  |
| GO:0050871 | positive regulation of B cell activation | | | 6/107 | | 119/17653 | | | 8.58E-05 | 0.003953706 | 0.003399185 | | VAV3/PRDM1/IGHV3-74/IGKC/IGHV3-43/IGHV3-73 | 6 |  |
| GO:0008217 | regulation of blood pressure | | | 7/107 | | 177/17653 | | | 0.000101558 | 0.004572401 | 0.003931106 | | NPR3/HMOX1/MME/ANPEP/NPY1R/NPR1/CARTPT | 7 |  |
| GO:0090026 | positive regulation of monocyte chemotaxis | | | 3/107 | | 17/17653 | | | 0.000138395 | 0.006092465 | 0.005237976 | | CCR1/PLA2G7/CXCL10 | 3 |  |
| GO:0043062 | extracellular structure organization | | | 10/107 | | 395/17653 | | | 0.000143281 | 0.006170433 | 0.005305008 | | IBSP/DPP4/NPNT/MMP9/ITGAX/CD36/PLA2G7/MMP7/MMP8/MMP12 | 10 |  |
| GO:0010038 | response to metal ion | | | 9/107 | | 334/17653 | | | 0.00019669 | 0.00829028 | 0.007127539 | | CASQ2/FIBIN/FABP4/HMOX1/RYR2/NLGN1/PLN/MMP9/AQP9 | 9 |  |
| GO:0006911 | phagocytosis, engulfment | | | 5/107 | | 90/17653 | | | 0.000217438 | 0.008973847 | 0.007715234 | | IGHV3-74/IGKC/IGHV3-43/IGHV3-73/CD36 | 5 |  |
| GO:0048660 | regulation of smooth muscle cell proliferation | | | 6/107 | | 143/17653 | | | 0.000234913 | 0.009497215 | 0.008165197 | | CNN1/MYOCD/NPR3/HMOX1/NPR1/MMP9 | 6 |  |
| GO:0048659 | smooth muscle cell proliferation | | | 6/107 | | 146/17653 | | | 0.000262795 | 0.009958113 | 0.008561453 | | CNN1/MYOCD/NPR3/HMOX1/NPR1/MMP9 | 6 |  |
| GO:0009074 | aromatic amino acid family catabolic process | | | 3/107 | | 21/17653 | | | 0.00026595 | 0.009958113 | 0.008561453 | | TDO2/KMO/KYNU | 3 |  |
| GO:0071677 | positive regulation of mononuclear cell migration | | | 3/107 | | 21/17653 | | | 0.00026595 | 0.009958113 | 0.008561453 | | CCR1/PLA2G7/CXCL10 | 3 |  |
| GO:0022617 | extracellular matrix disassembly | | | 5/107 | | 94/17653 | | | 0.000266421 | 0.009958113 | 0.008561453 | | DPP4/MMP9/MMP7/MMP8/MMP12 | 5 |  |
| GO:0010881 | regulation of cardiac muscle contraction by regulation of the release of sequestered calcium ion | | | 3/107 | | 22/17653 | | | 0.000306587 | 0.010907483 | 0.009377671 | | CASQ2/RYR2/PLN | 3 |  |
| GO:0090025 | regulation of monocyte chemotaxis | | | 3/107 | | 22/17653 | | | 0.000306587 | 0.010907483 | 0.009377671 | | CCR1/PLA2G7/CXCL10 | 3 |  |
| GO:0050853 | B cell receptor signaling pathway | | | 5/107 | | 97/17653 | | | 0.000308339 | 0.010907483 | 0.009377671 | | VAV3/IGHV3-74/IGKC/IGHV3-43/IGHV3-73 | 5 |  |
| GO:0006576 | cellular biogenic amine metabolic process | | | 4/107 | | 54/17653 | | | 0.000319579 | 0.011106763 | 0.009549001 | | TPH1/TDO2/KMO/KYNU | 4 |  |
| GO:0099024 | plasma membrane invagination | | | 5/107 | | 99/17653 | | | 0.00033894 | 0.011576556 | 0.009952904 | | IGHV3-74/IGKC/IGHV3-43/IGHV3-73/CD36 | 5 |  |
| GO:0042402 | cellular biogenic amine catabolic process | | | 3/107 | | 23/17653 | | | 0.000351025 | 0.011589675 | 0.009964183 | | TDO2/KMO/KYNU | 3 |  |
| GO:0042537 | benzene-containing compound metabolic process | | | 3/107 | | 23/17653 | | | 0.000351025 | 0.011589675 | 0.009964183 | | TDO2/KMO/KYNU | 3 |  |
| GO:0050864 | regulation of B cell activation | | | 6/107 | | 158/17653 | | | 0.000401345 | 0.013033829 | 0.011205789 | | VAV3/PRDM1/IGHV3-74/IGKC/IGHV3-43/IGHV3-73 | 6 |  |
| GO:1904705 | regulation of vascular smooth muscle cell proliferation | | | 4/107 | | 59/17653 | | | 0.000449363 | 0.013987009 | 0.012025283 | | CNN1/MYOCD/HMOX1/MMP9 | 4 |  |
| GO:1990874 | vascular smooth muscle cell proliferation | | | 4/107 | | 59/17653 | | | 0.000449363 | 0.013987009 | 0.012025283 | | CNN1/MYOCD/HMOX1/MMP9 | 4 |  |
| GO:0009310 | amine catabolic process | | | 3/107 | | 25/17653 | | | 0.000451877 | 0.013987009 | 0.012025283 | | TDO2/KMO/KYNU | 3 |  |
| GO:0010324 | membrane invagination | | | 5/107 | | 107/17653 | | | 0.000484876 | 0.014777532 | 0.012704932 | | IGHV3-74/IGKC/IGHV3-43/IGHV3-73/CD36 | 5 |  |
| GO:0006937 | regulation of muscle contraction | | | 6/107 | | 165/17653 | | | 0.0005052 | 0.015037056 | 0.012928057 | | CNN1/CASQ2/MYOCD/NPNT/RYR2/PLN | 6 |  |
| GO:1904706 | negative regulation of vascular smooth muscle cell proliferation | | | 3/107 | | 26/17653 | | | 0.000508573 | 0.015037056 | 0.012928057 | | CNN1/MYOCD/HMOX1 | 3 |  |
| GO:0010882 | regulation of cardiac muscle contraction by calcium ion signaling | | | 3/107 | | 27/17653 | | | 0.000569631 | 0.016594685 | 0.014267223 | | CASQ2/RYR2/PLN | 3 |  |
| GO:0043312 | neutrophil degranulation | | | 10/107 | | 485/17653 | | | 0.000726151 | 0.02084791 | 0.017923918 | | MME/ANPEP/FABP5/MMP9/ITGAX/CD36/FRK/CR1/MMP8/CHI3L1 | 10 |  |
| GO:0002283 | neutrophil activation involved in immune response | | | 10/107 | | 488/17653 | | | 0.000761257 | 0.021469004 | 0.018457902 | | MME/ANPEP/FABP5/MMP9/ITGAX/CD36/FRK/CR1/MMP8/CHI3L1 | 10 |  |
| GO:0030574 | collagen catabolic process | | | 4/107 | | 68/17653 | | | 0.000771209 | 0.021469004 | 0.018457902 | | MMP9/MMP7/MMP8/MMP12 | 4 |  |
| GO:0010880 | regulation of release of sequestered calcium ion into cytosol by sarcoplasmic reticulum | | | 3/107 | | 30/17653 | | | 0.000780297 | 0.021469004 | 0.018457902 | | CASQ2/RYR2/PLN | 3 |  |
| GO:0051480 | regulation of cytosolic calcium ion concentration | | | 8/107 | | 325/17653 | | | 0.000813556 | 0.02207745 | 0.018981011 | | CASQ2/CCR1/RYR2/PLN/CD52/CD36/GRIA1/CXCL10 | 8 |  |
| GO:0042119 | neutrophil activation | | | 10/107 | | 498/17653 | | | 0.000888555 | 0.023786871 | 0.02045068 | | MME/ANPEP/FABP5/MMP9/ITGAX/CD36/FRK/CR1/MMP8/CHI3L1 | 10 |  |
| GO:0002446 | neutrophil mediated immunity | | | 10/107 | | 500/17653 | | | 0.000916008 | 0.02419482 | 0.020801412 | | MME/ANPEP/FABP5/MMP9/ITGAX/CD36/FRK/CR1/MMP8/CHI3L1 | 10 |  |
| GO:0008037 | cell recognition | | | 6/107 | | 187/17653 | | | 0.000972466 | 0.025348096 | 0.021792938 | | CNTN4/IGHV3-74/IGKC/IGHV3-43/IGHV3-73/CD36 | 6 |  |
| GO:0030198 | extracellular matrix organization | | | 8/107 | | 341/17653 | | | 0.001108301 | 0.027982213 | 0.024057611 | | IBSP/DPP4/NPNT/MMP9/ITGAX/MMP7/MMP8/MMP12 | 8 |  |
| GO:1903779 | regulation of cardiac conduction | | | 4/107 | | 75/17653 | | | 0.0011143 | 0.027982213 | 0.024057611 | | CASQ2/RYR2/NPR1/PLN | 4 |  |
| GO:0019932 | second-messenger-mediated signaling | | | 8/107 | | 342/17653 | | | 0.001129231 | 0.027982213 | 0.024057611 | | CASQ2/PLEK/RYR2/MRAP2/PLN/CD36/SELE/CXCL10 | 8 |  |
| GO:0014808 | release of sequestered calcium ion into cytosol by sarcoplasmic reticulum | | | 3/107 | | 34/17653 | | | 0.001130024 | 0.027982213 | 0.024057611 | | CASQ2/RYR2/PLN | 3 |  |
| GO:0006501 | C-terminal protein lipidation | | | 4/107 | | 77/17653 | | | 0.001229227 | 0.029369991 | 0.025250748 | | CNTN4/NEGR1/CNTN3/CD52 | 4 |  |
| GO:0051279 | regulation of release of sequestered calcium ion into cytosol | | | 4/107 | | 77/17653 | | | 0.001229227 | 0.029369991 | 0.025250748 | | CASQ2/RYR2/PLN/CXCL10 | 4 |  |
| GO:1903514 | release of sequestered calcium ion into cytosol by endoplasmic reticulum | | | 3/107 | | 35/17653 | | | 0.001230545 | 0.029369991 | 0.025250748 | | CASQ2/RYR2/PLN | 3 |  |
| GO:0006936 | muscle contraction | | | 8/107 | | 352/17653 | | | 0.001356442 | 0.03195228 | 0.027470862 | | CNN1/CASQ2/MYOCD/NPNT/RYR2/MYOM1/PLN/ACTC1 | 8 |  |
| GO:0042113 | B cell activation | | | 7/107 | | 273/17653 | | | 0.001370996 | 0.03195228 | 0.027470862 | | VAV3/PRDM1/IGHV3-74/IGKC/IGHV3-43/IGHV3-73/CD180 | 7 |  |
| GO:0046849 | bone remodeling | | | 4/107 | | 82/17653 | | | 0.001552419 | 0.034925919 | 0.030027438 | | DCSTAMP/TPH1/ACP5/CARTPT | 4 |  |
| GO:0070296 | sarcoplasmic reticulum calcium ion transport | | | 3/107 | | 38/17653 | | | 0.001565319 | 0.034925919 | 0.030027438 | | CASQ2/RYR2/PLN | 3 |  |
| GO:0071675 | regulation of mononuclear cell migration | | | 3/107 | | 38/17653 | | | 0.001565319 | 0.034925919 | 0.030027438 | | CCR1/PLA2G7/CXCL10 | 3 |  |
| GO:0030240 | skeletal muscle thin filament assembly | | | 2/107 | | 10/17653 | | | 0.00158674 | 0.034925919 | 0.030027438 | | MYOM1/ACTC1 | 2 |  |
| GO:0072531 | pyrimidine-containing compound transmembrane transport | | | 2/107 | | 10/17653 | | | 0.00158674 | 0.034925919 | 0.030027438 | | SLC28A3/AQP9 | 2 |  |
| GO:0050867 | positive regulation of cell activation | | | 8/107 | | 363/17653 | | | 0.001646992 | 0.035711104 | 0.030702498 | | DPP4/VAV3/PRDM1/PLEK/IGHV3-74/IGKC/IGHV3-43/IGHV3-73 | 8 |  |
| GO:0003012 | muscle system process | | | 9/107 | | 450/17653 | | | 0.001658466 | 0.035711104 | 0.030702498 | | CNN1/CASQ2/MYOCD/HMOX1/NPNT/RYR2/MYOM1/PLN/ACTC1 | 9 |  |
| GO:0033002 | muscle cell proliferation | | | 6/107 | | 211/17653 | | | 0.001802778 | 0.038401116 | 0.033015227 | | CNN1/MYOCD/NPR3/HMOX1/NPR1/MMP9 | 6 |  |
| GO:0018410 | C-terminal protein amino acid modification | | | 4/107 | | 86/17653 | | | 0.001850292 | 0.038993925 | 0.033524892 | | CNTN4/NEGR1/CNTN3/CD52 | 4 |  |
| GO:0002377 | immunoglobulin production | | | 5/107 | | 146/17653 | | | 0.001953472 | 0.040735029 | 0.0350218 | | IGKC/IGKV3D-11/IGKV1D-43/IGKV3D-20/IGKV2D-26 | 5 |  |
| GO:0009187 | cyclic nucleotide metabolic process | | | 5/107 | | 147/17653 | | | 0.002012656 | 0.041531993 | 0.035706988 | | NPR3/PDE8B/MRAP2/NPR1/CXCL10 | 5 |  |
| GO:0007204 | positive regulation of cytosolic calcium ion concentration | | | 7/107 | | 295/17653 | | | 0.002129294 | 0.043485881 | 0.037386836 | | CASQ2/CCR1/RYR2/PLN/CD52/CD36/CXCL10 | 7 |  |
| GO:0048738 | cardiac muscle tissue development | | | 6/107 | | 221/17653 | | | 0.002274897 | 0.045738691 | 0.039323681 | | MYOCD/NEXN/RYR2/MYOM1/PLN/ACTC1 | 6 |  |
| GO:0014866 | skeletal myofibril assembly | | | 2/107 | | 12/17653 | | | 0.002308869 | 0.045738691 | 0.039323681 | | MYOM1/ACTC1 | 2 |  |
| GO:0033631 | cell-cell adhesion mediated by integrin | | | 2/107 | | 12/17653 | | | 0.002308869 | 0.045738691 | 0.039323681 | | DPP4/NPNT | 2 |  |
| ID | Description | | | GeneRatio | | BgRatio | | | pvalue | p.adjust | qvalue | | geneID | Count |  |
| GO:0009897 | external side of plasma membrane | | | 12/112 | | 287/18698 | | | 1.53E-07 | 3.05E-05 | 2.40E-05 | | IL31RA/CD163/CCR1/NLGN1/IGHV3-74/IGKC/IGHV3-43/ANPEP/IGHV3-73/CD36/CXCL10/GRIA2 | 12 |  |
| GO:0072562 | blood microparticle | | | 9/112 | | 183/18698 | | | 1.56E-06 | 0.000155631 | 0.000122661 | | IGKV1D-33/IGHV3-74/IGKC/IGHV3-7/IGHV3-43/IGKV3D-11/IGHV3-73/IGKV2D-28/ACTC1 | 9 | |
| GO:0031012 | extracellular matrix | | | 11/112 | | 479/18698 | | | 0.000142293 | 0.007710242 | 0.006076837 | IBSP/LRRN1/NPNT/SBSPON/MMRN1/FREM1/MMP9/MMP7/MMP8/MMP12/CHI3L1 | | 11 |  |
| GO:0031674 | I band | | | 6/112 | | 138/18698 | | | 0.000182647 | 0.007710242 | 0.006076837 | FHL5/CASQ2/NEXN/RYR2/MYOM1/ACTC1 | | 6 |  |
| GO:0042571 | immunoglobulin complex, circulating | | | 4/112 | | 48/18698 | | | 0.000193725 | 0.007710242 | 0.006076837 | IGHV3-74/IGKC/IGHV3-43/IGHV3-73 | | 4 |  |
| GO:0019814 | immunoglobulin complex | | | 4/112 | | 51/18698 | | | 0.000245391 | 0.008138815 | 0.006414617 | IGHV3-74/IGKC/IGHV3-43/IGHV3-73 | | 4 |  |
| GO:0044449 | contractile fiber part | | | 7/112 | | 214/18698 | | | 0.000305847 | 0.008694804 | 0.006852821 | FHL5/CASQ2/NEXN/NPNT/RYR2/MYOM1/ACTC1 | | 7 |  |
| GO:0031225 | anchored component of membrane | | | 6/112 | | 159/18698 | | | 0.000391811 | 0.009389386 | 0.007400257 | CNTN4/NEGR1/CNTN1/CNTN3/CD52/ITLN1 | | 6 |  |
| GO:0005578 | proteinaceous extracellular matrix | | | 9/112 | | 375/18698 | | | 0.000426233 | 0.009389386 | 0.007400257 | LRRN1/NPNT/SBSPON/FREM1/MMP9/MMP7/MMP8/MMP12/CHI3L1 | | 9 |  |
| GO:0043292 | contractile fiber | | | 7/112 | | 230/18698 | | | 0.000471828 | 0.009389386 | 0.007400257 | | FHL5/CASQ2/NEXN/NPNT/RYR2/MYOM1/ACTC1 | 7 |  |
| GO:0030018 | Z disc | | | 5/112 | | 125/18698 | | | 0.000934338 | 0.016903023 | 0.01332214 | | FHL5/CASQ2/NEXN/RYR2/MYOM1 | 5 |  |
| GO:0030017 | sarcomere | | | 6/112 | | 197/18698 | | | 0.001202407 | 0.019939921 | 0.015715674 | | FHL5/CASQ2/NEXN/RYR2/MYOM1/ACTC1 | 6 |  |
| GO:0033017 | sarcoplasmic reticulum membrane | | | 3/112 | | | | 39/18698 | 0.001634197 | 0.025015788 | 0.019716225 | | CASQ2/RYR2/PLN | 3 |  |
| GO:0043235 | receptor complex | | | 8/112 | | | | 374/18698 | 0.001856594 | 0.026390162 | 0.02079944 | | NPNT/NLGN1/NPR1/ITGAX/CD36/GRIA1/GRIA2/ITLN1 | 8 |  |
| GO:0030016 | myofibril | | | 6/112 | | 219/18698 | | | 0.002057678 | 0.027298534 | 0.021515375 | | FHL5/CASQ2/NEXN/RYR2/MYOM1/ACTC1 | 6 |  |
| GO:0030666 | endocytic vesicle membrane | | | 5/112 | | 161/18698 | | | 0.002846576 | 0.03540429 | 0.027903936 | | CD163/CD36/GRIA1/ATP6V0D2/GRIA2 | 5 |  |
| GO:0008328 | ionotropic glutamate receptor complex | | | 3/112 | | 49/18698 | | | 0.003154682 | 0.036928337 | 0.029105116 | | NLGN1/GRIA1/GRIA2 | 3 |  |
| GO:0098878 | neurotransmitter receptor complex | | | 3/112 | | 51/18698 | | | 0.003535053 | 0.039081974 | 0.030802508 | | NLGN1/GRIA1/GRIA2 | 3 |  |
| GO:0098802 | plasma membrane receptor complex | | | 5/112 | | 179/18698 | | | 0.004471866 | 0.046836914 | 0.036914574 | | NPNT/NLGN1/ITGAX/GRIA1/GRIA2 | 5 |  |
| ID | | Description | GeneRatio | | BgRatio | | pvalue | | | p.adjust | qvalue | | geneID | Count |  |
| GO:0003823 | | antigen binding | 14/102 | | 195/17548 | | 6.92E-12 | | | 2.10E-09 | 1.85E-09 | | IGKV1D-33/IGHV3-74/IGKV1-13/IGKC/IGHV3-7/IGHV3-43/IGHV4-59/IGKV3D-11/IGKV1D-43/IGHV3-73/IGKV3D-20/IGKV2D-26/IGKV2D-28/IGHV1OR15-1 | 14 |  |
| GO:0004252 | | serine-type endopeptidase activity | | 14/102 | | 248/17548 | | | 1.73E-10 | 2.62E-08 | 2.31E-08 | | DPP4/IGKV1D-33/IGKC/C2/IGHV3-7/IGHV4-59/IGKV3D-11/IGKV3D-20/MMP9/IGKV2D-28/MMP7/IGHV1OR15-1/MMP8/MMP12 | 14 |  |
| GO:0008236 | serine-type peptidase activity | | | 14/102 | | 276/17548 | | | 7.05E-10 | 6.74E-08 | 5.95E-08 | | DPP4/IGKV1D-33/IGKC/C2/IGHV3-7/IGHV4-59/IGKV3D-11/IGKV3D-20/MMP9/IGKV2D-28/MMP7/IGHV1OR15-1/MMP8/MMP12 | 14 |  |
| GO:0017171 | serine hydrolase activity | | | 14/102 | | 281/17548 | | | 8.90E-10 | 6.74E-08 | 5.95E-08 | | DPP4/IGKV1D-33/IGKC/C2/IGHV3-7/IGHV4-59/IGKV3D-11/IGKV3D-20/MMP9/IGKV2D-28/MMP7/IGHV1OR15-1/MMP8/MMP12 | 14 |  |
| GO:0004175 | endopeptidase activity | | | 16/102 | | 474/17548 | | | 1.40E-08 | 8.49E-07 | 7.49E-07 | | DPP4/IGKV1D-33/IGKC/C2/IGHV3-7/MME/IGHV4-59/IGKV3D-11/IGKV3D-20/MMP9/IGKV2D-28/MMP7/IGHV1OR15-1/MMP8/ADAMDEC1/MMP12 | 16 |  |
| GO:0004222 | metalloendopeptidase activity | | | 6/102 | | 112/17548 | | | 4.84E-05 | 0.002442482 | 0.002155256 | | MME/MMP9/MMP7/MMP8/ADAMDEC1/MMP12 | 6 |  |
| GO:0008237 | metallopeptidase activity | | | 7/102 | | 185/17548 | | | 0.000102659 | 0.004443665 | 0.003921108 | | MME/ANPEP/MMP9/MMP7/MMP8/ADAMDEC1/MMP12 | 7 |  |
| GO:0042277 | peptide binding | | | 8/102 | | 273/17548 | | | 0.000192178 | 0.00727873 | 0.00642278 | | NPR3/NLGN1/MME/ANPEP/NPR1/CD36/GRIA1/GRIA2 | 8 |  |
| GO:0034987 | immunoglobulin receptor binding | | | 4/102 | | 52/17548 | | | 0.000235111 | 0.007915392 | 0.006984574 | | IGHV3-74/IGKC/IGHV3-43/IGHV3-73 | 4 |  |
| GO:0001540 | amyloid-beta binding | | | 4/102 | | 57/17548 | | | 0.000335504 | 0.01016576 | 0.008970307 | | NLGN1/CD36/GRIA1/GRIA2 | 4 |  |
| GO:0033218 | amide binding | | | 8/102 | | 308/17548 | | | 0.000432249 | 0.011906508 | 0.010506351 | | NPR3/NLGN1/MME/ANPEP/NPR1/CD36/GRIA1/GRIA2 | 8 |  |
| GO:0030246 | carbohydrate binding | | | 7/102 | | 266/17548 | | | 0.00092221 | 0.023285799 | 0.020547483 | | CNTN1/SBSPON/ATRNL1/FREM1/SELE/CHI3L1/ITLN1 | 7 |  |
| GO:0008081 | phosphoric diester hydrolase activity | | | 4/102 | | 91/17548 | | | 0.00195245 | 0.045507112 | 0.040155659 | | PLCB4/CCR1/HMOX1/PDE8B | 4 |  |
| ID | Description | | | GeneRatio | | BgRatio | | | pvalue | p.adjust | qvalue | | geneID | Count |  |
| hsa03320 | PPAR signaling pathway | | | 5/51 | | 76/8106 | | | 0.000106434 | 0.010173772 | 0.00899301 | | 2167/33/123/2171/948 | 5 |  |
| hsa00380 | Tryptophan metabolism | | | 4/51 | | 42/8106 | | | 0.000130433 | 0.010173772 | 0.00899301 | | 7166/6999/8564/8942 | 4 |  |
| hsa04024 | cAMP signaling pathway | | | 7/51 | | 216/8106 | | | 0.000369712 | 0.019224999 | 0.016993758 | | 10451/6262/4886/4881/5350/2890/2891 | 7 |  |
| hsa04720 | Long-term potentiation | | | 4/51 | | 67/8106 | | | 0.000795585 | 0.031027822 | 0.027426752 | | 27330/5332/2890/2891 | 4 |  |
